# Characterizing the Human Mycobiota: A Comparison of Small Subunit rRNA, ITS1, ITS2, and Large Subunit rRNA Genomic Targets

**DOI:** 10.3389/fmicb.2018.02208

**Published:** 2018-09-19

**Authors:** Michael Hoggard, Anna Vesty, Giselle Wong, Johanna M. Montgomery, Chantelle Fourie, Richard G. Douglas, Kristi Biswas, Michael W. Taylor

**Affiliations:** ^1^School of Biological Sciences, The University of Auckland, Auckland, New Zealand; ^2^School of Medicine, The University of Auckland, Auckland, New Zealand; ^3^Microbiology Laboratory, LabPLUS, Auckland City Hospital, Auckland, New Zealand; ^4^Department of Physiology, Centre for Brain Research, The University of Auckland, Auckland, New Zealand; ^5^Maurice Wilkins Centre for Molecular Biodiscovery, The University of Auckland, Auckland, New Zealand

**Keywords:** fungi, human microbiome, microbiota, mycobiota, internal transcribed spacer, next-generation sequencing

## Abstract

Interest in the human microbiome has increased dramatically in the last decade. However, much of this research has focused on bacteria, while the composition and roles of their fungal counterparts remain less understood. Furthermore, a variety of methodological approaches have been applied, and the comparability between studies is unclear. This study compared four primer pairs targeting the small subunit (SSU) rRNA (18S), ITS1, ITS2, and large subunit (LSU) rRNA (26S) genomic regions for their ability to accurately characterize fungal communities typical of the human mycobiota. All four target regions of 21 individual fungal mock community taxa were capable of being amplified adequately and sequenced. Mixed mock community analyses revealed marked variability in the ability of each primer pair to accurately characterize a complex community. ITS target regions outperformed LSU and SSU. Of the ITS regions, ITS1 failed to generate sequences for *Yarrowia lipolytica* and all three *Malassezia* species when in a mixed community. These findings were further supported in studies of human sinonasal and mouse fecal samples. Based on these analyses, previous studies using ITS1, SSU, or LSU markers may omit key taxa that are identified by the ITS2 marker. Of methods commonly used in human mycobiota studies to date, we recommend selection of the ITS2 marker. Further investigation of more recently developed fungal primer options will be essential to ultimately determine the optimal methodological approach by which future human mycobiota studies ought to be standardized.

## Introduction

Interest in the human microbiome has increased dramatically in the last decade. The advent of modern molecular sequencing-based tools has rapidly expanded our understanding of microbes as essential players in the immune development, ongoing health, and the establishment of disease of many non-microbial members of the tree of life, including humans. Our resident microbiota is now thought to play important roles in immune priming and development, metabolism, and chronic inflammatory disease, with potential involvement in conditions as diverse as type 2 diabetes, autism spectrum disorder, depression, and inflammatory bowel, cardiac, and respiratory diseases (Cho and Blaser, 2012; Cryan and Dinan, 2012; Nicholson et al., 2012; Hoggard et al., 2018). However, much of the research to date has focused on the bacterial members of our microbial communities, while the composition and roles of their fungal, viral, and archaeal counterparts remain comparably less well understood.

Fungi are involved in a range of human diseases, and invasive forms of fungal infection can have significant associated mortality (Brown et al., 2012; Cui et al., 2013; Halwachs et al., 2017). Immunocompromised individuals are particularly at risk, but subtler roles in immunomodulation and influencing inflammatory disease are also of increasing interest (Rizzetto et al., 2014; Underhill and Iliev, 2014; Nguyen et al., 2015; Zhang et al., 2017). As with recent findings in bacterial microbiota studies (Hajishengallis and Lambris, 2012; Vujkovic-Cvijin et al., 2013; Nylund et al., 2015; Cope et al., 2017; Hoggard et al., 2017a,b), dysbiosis of the fungal proportion of microbiota (the mycobiota) may also influence disease pathogenesis, as well as the interconnected dynamics of their bacterial, viral, and archaeal counterparts (Oever and Netea, 2014; Saxena and Sitaraman, 2014; Underhill and Iliev, 2014; Nguyen et al., 2015). For example, effects on co-colonization patterns and fungal morphological development have been observed (Oever and Netea, 2014), and antibacterials have been shown to influence associated fungal communities, likely due to the shifted dynamics with their affected bacterial counterparts. In some cases, this can result in fungal blooms, mucosal invasion, and life-threatening illness (Oever and Netea, 2014; Rizzetto et al., 2014; Underhill and Iliev, 2014).

**FIGURE 1:**
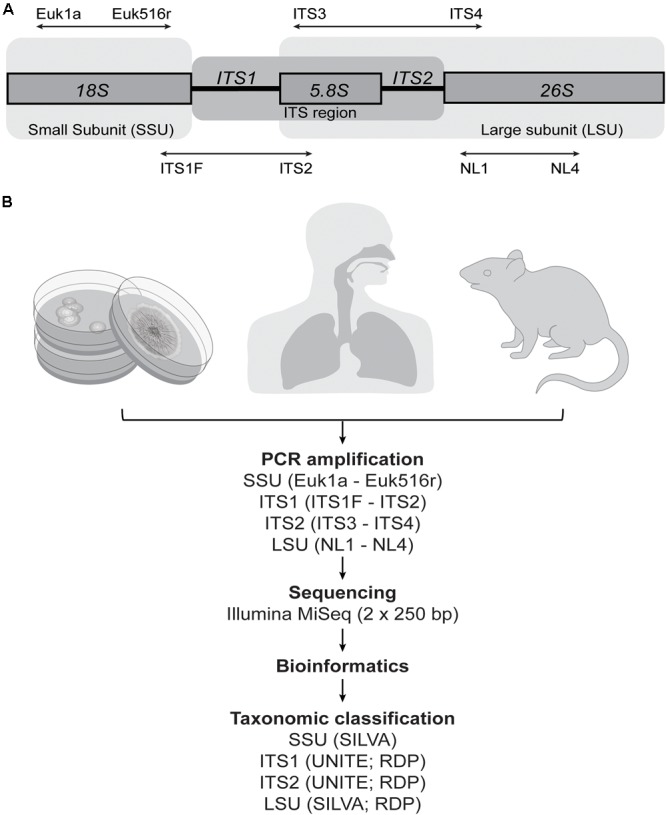
Methodological overview. **(A)** The fungal ribosomal cistron, together with the primers and target regions assessed in this study. ITS, internal transcribed spacer; SSU, small subunit; LSU, large subunit. **(B)** Samples were collected from 21 fungal isolates, human upper-respiratory tract (sinonasal swab) (*n* = 10), and mouse gut (fecal) material (*n* = 10). All samples were amplified using primer pairs for four target genomic regions and sequenced on the Illumina MiSeq platform to compare the ability of each to accurately characterize the fungal communities in the samples. Readily available reference databases were also compared to assess the accuracy of taxonomic assignments of each of the sequence data sets.

The lack of a robust and standardized methodology that accurately characterizes the full fungal diversity of complex communities remains a limitation of the field. In bacteria, the 16S rRNA gene, with its interspersed conserved and hyper-variable regions, allows for effective targeting of most of the known bacterial domain, as well as reasonable differentiation among bacterial taxa. A comparable target for fungi has yet to be consistently applied across studies. The main candidate fungal genomic targets are regions from the eukaryote ribosomal cistron, including the small subunit (SSU) (18S) and large subunit (LSU) (26S) rRNA genes, which are separated by the internal transcribed spacer (ITS) region (the 5.8S rRNA gene flanked by two ITS regions: ITS1 and ITS2) (**Figure 1A**). Each target has limitations, and it remains unclear how these biases specifically affect efforts to characterize the taxonomic diversity typical of the human mycobiota. *In silico* analyses as well as comparative studies from environmental samples have highlighted the various biases that primer selection can introduce (Bellemain et al., 2010; Mello et al., 2011; Ihrmark et al., 2012; Bazzicalupo et al., 2013; Blaalid et al., 2013; Tedersoo et al., 2015; Cottier et al., 2018), with one study identifying that primer pair selection explained more of the variation in the data (38%) than any environmental signal assessed (Tedersoo et al., 2015). Schoch et al. (2012) made a formal recommendation for the adoption of ITS as the optimal primary fungal barcode marker due to increased species resolution, probability of correct identification, and amplification success rate. This was further supported in a later study by Tedersoo et al. (2015), highlighting ITS2 in particular as the strongest candidate. Nonetheless, an array of marker targets have been used in human microbiota studies, including both ITS regions (Ghannoum et al., 2010; Charlson et al., 2012; Delhaes et al., 2012; Findley et al., 2013; Bittinger et al., 2014; Dupuy et al., 2014; Wang X. et al., 2014; Willger et al., 2014; Hoarau et al., 2016; Liguori et al., 2016; Strati et al., 2016; Botschuijver et al., 2017; Gao et al., 2017; Hamad et al., 2017; Huseyin et al., 2017; Motooka et al., 2017; Schei et al., 2017; Zhao et al., 2017) as well as SSU (Aurora et al., 2013; van Woerden et al., 2013; Cleland et al., 2014; Li et al., 2014) and LSU (Zhang et al., 2011; Park et al., 2012). The use of different methodological approaches makes meaningful comparisons between studies difficult, and it is unclear whether observed differences are genuine (e.g., due to geographical influences affecting the fungal exposure of different populations) or simply biases associated with different methods.

It is vital to assess the ability of each of these different target regions to accurately characterize mixed fungal communities in order to determine the degree of comparability between studies to date, and to establish a single methodological approach going forward which is adequately specific to the types of fungal communities typical of the human mycobiota. The aim of this study was to systematically compare common representative primer pairs used in human mycobiota studies for each of the SSU, ITS1, ITS2, and LSU target genomic regions. We examined the ability of each to accurately characterize a known mock community, which was constructed for this study, and which comprised 21 fungal taxa that have previously been established as characteristic members of mammalian-associated mycobiota. To further examine and present implications of methodological biases in the context of both respiratory- and gastrointestinal-associated fungi, each approach was also applied to pilot studies of 10 human sinonasal swab samples and 10 samples from mouse fecal material (**Figure 1B**).

## Materials and Methods

### Human Mycobiota Mock Community

Fungal species candidates were selected based on taxa previously identified in a broad range of published studies, including of the lung (Agbetile et al., 2012; Charlson et al., 2012; Delhaes et al., 2012; Huffnagle and Noverr, 2013; Liu et al., 2013; van Woerden et al., 2013; Bittinger et al., 2014; Chotirmall and McElvaney, 2014; Pashley, 2014; Pourfathollah et al., 2014; Willger et al., 2014; Eickmeier et al., 2015; Nguyen et al., 2015; Sharpe et al., 2015), sinonasal cavity (Aurora et al., 2013; Cleland et al., 2014; Jung et al., 2015; Comacle et al., 2016; Gelber et al., 2016; Zhao et al., 2017), oral cavity (Ghannoum et al., 2010; Huffnagle and Noverr, 2013; Dupuy et al., 2014; Underhill and Iliev, 2014; Vesty et al., 2017), gastrointestinal tract (Huffnagle and Noverr, 2013; Kim et al., 2014; Li et al., 2014; Underhill and Iliev, 2014; Wang Z.K. et al., 2014; Gerard et al., 2015; Tang et al., 2015; Hoarau et al., 2016; Liguori et al., 2016; Strati et al., 2016; Hallen-Adams and Suhr, 2017; Nash et al., 2017), vagina (Underhill and Iliev, 2014), and skin (Zhang et al., 2011; Park et al., 2012; Findley et al., 2013; Huffnagle and Noverr, 2013; Underhill and Iliev, 2014). Findings from both culture studies and molecular-based approaches with a range of marker gene targets were considered. To ensure that findings were appropriately representative across the range of studies and experimental (animal) models of the human microbiota, fungi previously identified among mammalian-associated mycobiota (with a particular focus on murine and rabbit fungal studies) were also considered.

**FIGURE 2:**
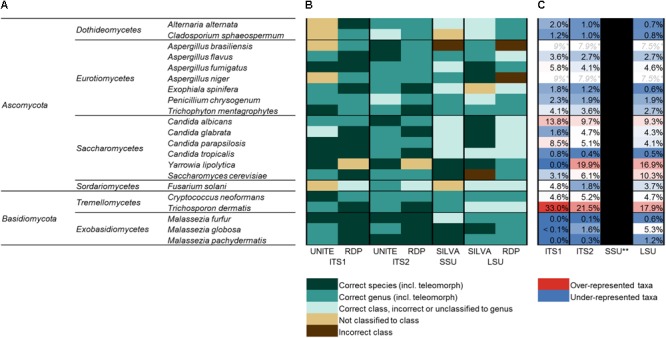
The human mycobiota mock community. **(A)** Full list of species included in the human mycobiota mock community assembled for this study (phylum; class; genus; and species). All taxa were sequenced individually and as a mixed mock community. **(B)** Accuracy of taxonomic assignments for each of the 21 taxa (sequenced individually) and four target regions based on UNITE (ITS), RDP (ITS, LSU), and SILVA (SSU, LSU) reference databases. **(C)** Relative proportion of sequences for each species when amplified and sequenced as a mixed mock community, color-coded by whether sequences for each were over-represented (red) or under-represented (blue) compared to the expected (even) distribution (expected = 4.7% relative abundance for each species). ^∗^*A. brasiliensis* and *A. niger* shared the same ZOTU for each of ITS1, ITS2, and LSU (i.e., had identical sequences), and *C. albicans* and *C. parapsilosis* shared the same ZOTU for SSU. ^∗∗^*A. brasiliensis* was split across numerous ZOTUs for SSU sequences and had mixed classification, making relative abundance estimates not possible for SSU.

In order to also assess the ability of the different target regions to differentiate species within some genera of interest, multiple representatives were included for *Aspergillus* (four species), *Candida* (four species), and *Malassezia* (three species). In all, 21 species spanning 13 fungal genera were selected. Fungal isolates were obtained from commercially available type-strain collections [American Type Culture Collection (ATCC) (Manassas, VA, United States), and the New Zealand Reference Culture Collection (NZRM) (Wellington, New Zealand)], as well as locally isolated human-associated wild strains (LabPLUS, Auckland, New Zealand). A full list of species included is given in **Figure 2A**, with further detail provided in **Supplementary Figure 1**.

### Human Sinonasal and Mouse Fecal Samples

To further investigate methodological biases in the context of actual mammalian mycobiome samples, upper respiratory (human sinonasal cavity) and gut (mouse fecal material) samples were also assessed. For sinonasal samples, pairs of sterile rayon-tipped swabs (Copan Diagnostics, Inc., Murrieta, CA, United States) were used to sample the mucosa of the left and right middle meatus in 10 healthy subjects. Swab pairs were placed in sterile 1.5 mL tubes together with RNA*later* (Life Technologies, Auckland, New Zealand), transferred to the laboratory, and stored at -20°C until DNA extraction. Mouse fecal pellets [C57BL/6J mice (Jackson Labs)] were collected using sterilized forceps and stored in sterile 1.5 mL tubes at -20°C.

### Processing of Cultures and DNA Extraction

Fungal isolates were grown from stock cultures on Sabouraud Dextrose Agar with antibiotics (Fort Richard Laboratories Ltd., Auckland, New Zealand) at 27–30°C. Fungal colonies were removed from agar plates and placed in sterile 1.5 mL tubes together with RNA*later* (Life Technologies, Auckland, New Zealand), transferred to the laboratory, and stored at -20°C.

DNA extractions were conducted using AllPrep DNA/RNA Isolation Kits (Qiagen, Hilden, Germany). Fungal isolates, sinonasal swab pairs, and mouse fecal material were each first placed in Lysing Matrix E bead tubes (MP Biomedicals, Sydney, NSW, Australia) together with 600 μL of RLT Plus lysis buffer, and ruptured at 25 m/s for 2 × 40 s using a Tissue Lyser II (Qiagen, Hilden, Germany). Following centrifugation (5 min at 1500 *g*), the supernatant was transferred to DNA collection columns and processed as per manufacturer’s instructions. DNA was quantified using Qubit double-stranded DNA (high sensitivity) Assay kit (Life Technologies, Auckland, New Zealand).

### PCR Amplification and Sequencing

Primers were selected based on representative primer pairs for each of the four target genomic regions that have been commonly used in published mammalian mycobiota studies: Euk1a – Euk516r (SSU) (Sogin and Gunderson, 1987; Amann et al., 1990; van Woerden et al., 2013); ITS1F – ITS2 (ITS1) (White et al., 1990; Gardes and Bruns, 1993; Ghannoum et al., 2010; Charlson et al., 2012; Bittinger et al., 2014; Dupuy et al., 2014; Wang X. et al., 2014; Hoarau et al., 2016; Botschuijver et al., 2017; Hamad et al., 2017; Huseyin et al., 2017; Motooka et al., 2017; Schei et al., 2017; Zhao et al., 2017); ITS3 – ITS4 (ITS2) (White et al., 1990; Wang X. et al., 2014; Strati et al., 2016; Gao et al., 2017; Hamad et al., 2017); NL1 – NL4 (LSU) (O’Donnell, 1992; Zhang et al., 2011; Park et al., 2012; **Table 1**). Target regions were amplified and sequenced for each of the 21 mock community taxa individually, and as a mixed community which was constructed by pooling equal concentrations of DNA to generate an even 21 species mock community.

**Table 1:** Primers targeting fungal SSU, ITS1, ITS2, and LSU.

Target	Primer name	Nextera adapters (5′–3′)	Primer sequence (5′–3′)	Reference
18S	Euk1a	TCGTCGGCAGCGTCAGATGTGTATAAGAGACAG	CTGGTTGATCCTGCCAG	Sogin and Gunderson, 1987
	Euk516r	GTCTCGTGGGCTCGGAGATGTGTATAAGAGACAG	ACCAGACTTGCCCTCC	Amann et al., 1990
ITS1	ITS1F	TCGTCGGCAGCGTCAGATGTGTATAAGAGACAG	CTTGGTCATTTAGAGGAAGTAA	Gardes and Bruns, 1993
	ITS2	GTCTCGTGGGCTCGGAGATGTGTATAAGAGACAG	GCTGCGTTCTTCATCGATGC	White et al., 1990
ITS2	ITS3	TCGTCGGCAGCGTCAGATGTGTATAAGAGACAG	GCATCGATGAAGAACGCAGC	White et al., 1990
	ITS4	GTCTCGTGGGCTCGGAGATGTGTATAAGAGACAG	TCCTCCGCTTATTGATATGC	White et al., 1990
26S	NL-1	TCGTCGGCAGCGTCAGATGTGTATAAGAGACAG	GCATATCAATAAGCGGAGGAAAAG	O’Donnell, 1992
	NL-4	GTCTCGTGGGCTCGGAGATGTGTATAAGAGACAG	GGTCCGTGTTTCAAGACGG	O’Donnell, 1992

Target regions for all samples were amplified in triplicate under the following PCR conditions: initial enzyme activation and denaturing for 15 min at 95°C, 30 cycles of 95°C for 30 s, 52°C (ITS1 and ITS2) or 57°C (SSU and LSU) for 30 s, and 70°C for 60 s, and a final extension step of 70°C (7 min). For sinonasal and fecal samples, these PCR conditions were increased to 35 cycles to generate sufficient PCR product for sequencing. Each reaction mix contained template DNA [0.1 ng for mock community samples, ∼100 ng (∼50 ng from each of the left and right sides) for sinonasal samples, and 10 ng for mouse fecal samples], 0.5 U HotStar DNA polymerase and HotStar PCR buffer (×1) (Qiagen, Hilden, Germany), equimolar concentrations (0.2 μM) of each primer, 0.2 mM deoxynucleotides (dNTPs), 2 mM MgCl_2_, and PCR-grade water to a final volume of 25 μL. Triplicate PCRs of the mixed mock community were purified and sequenced separately in order to also examine the variability between PCR replicates. For all other samples, triplicate PCRs were pooled for purification. PCR products were purified using Agencourt AMPure beads (Beckman–Coulter, Brea, CA, United States) as per the manufacturer’s instructions, and submitted to the sequencing provider (New Zealand Genomics Ltd.) who performed library preparation and sequencing on the Illumina MiSeq platform (2 × 250 bp paired-end reads). Raw sequences have been deposited onto the SRA-NCBI database (accession SRP132544).

### Bioinformatics

Fungal amplicon sequence data present issues that are not adequately addressed by standard bioinformatics pipelines developed for bacterial 16S rRNA gene sequencing (Diaz et al., 2017; Halwachs et al., 2017). For example, fungi ITS sequences are highly variable in length and, when merging paired-end Illumina reads, in some cases may be longer than the possible merged read length and will be discarded. A preliminary comparison of the data using merged paired-end reads vs. analyzing only forward reads identified that 3/21 of the mock community taxa would be effectively omitted from the merged-reads data for each of the ITS markers, and all taxa would be excluded from SSU and LSU data (SSU and LSU amplicons for all taxa were >550 bp), while forward reads alone generated data for all 21 taxa across all four target markers (**Supplementary Table 1**). To ensure taxa were not excluded on this basis, merging of paired reads was omitted from the pipeline and only forward reads were retained. Additionally, any ITS sequences shorter than the read length will generate erroneous sections of sequence after the reverse primer-binding region has been reached (which would normally be trimmed during paired-end merging). For this study, a customized bioinformatics pipeline was developed incorporating usearch (v10) (Edgar, 2010) and custom scripts to address these issues (bioinformatics pipeline^1^).

Forward reads were trimmed to a maximum read length of 230 bp (reverse reads were discarded) to remove the ends of reads that contain higher expected error rates, and then further trimmed to remove forward and reverse primer binding regions (together with any sequence either side), filtered based on maximum expected error rate of 1 and minimum length of 50 bp, and singletons removed (Edgar and Flyvbjerg, 2015). While a specific sequence similarity (such as 97%) for generating operational taxonomic units (OTUs) is often accepted as differentiating species or genera (depending on the sequence length) in bacteria, this is more variable in fungi for different lineages, thus making acceptable genus or species level cut-off equivalents difficult (Diaz et al., 2017; Halwachs et al., 2017). Instead, zero-radius OTUs (ZOTUs), representing 100% sequence similarity for each ZOTU [analogous to amplicon sequence variants (ASV)], were generated using the –unoise3 algorithm (Edgar, 2016b) in usearch with the default settings. Taxonomic assignments were generated via the sintax classifier in usearch (Edgar, 2016a), with multiple reference databases compared. For the two ITS regions, UNITE (UNITE_22.08.2016) (UNITE Community, 2017) and Ribosomal Database Project (RDP) (rdp_its_v2: Warcup training set v2) (Cole et al., 2014) reference databases were available for download from the usearch website correctly formatted for use with usearch. SILVA databases were downloaded for LSU (SILVA_128_LSURef_tax_trunc) and SSU (SILVA_128_SSURef_Nr99_tax_silva_trunc) (Quast et al., 2013), and manually curated and customized to remove taxonomic levels not accepted by usearch/sintax (e.g., sub-phyla), and to meet general usearch formatting requirements. Finally, an LSU database (current_Fungi_unaligned) was also downloaded from RDP (Cole et al., 2014), which contained the correct taxonomic levels for use with usearch and was customized to meet the formatting requirements for usearch’s sintax algorithm. All ZOTUs classified as “unassigned,” “fungi (unassigned),” and “plantae (unassigned)” were manually checked using NCBI blastn online, and those identified as non-target organisms (such as human- or mouse-associated sequences) were discarded. ZOTU tables were generated, and then filtered for cross-talk (Edgar, 2017) to a minimum ZOTU frequency of 0.001. All mock community data were subsampled to an even sequencing depth of 2000 reads per sample, and all sinonasal and fecal data to 1000 reads per sample. Pairwise sequence alignments were generated in usearch via the -allpairs_global command for mock community taxa that generated more than one unique ZOTU. Further examination of ZOTU tables and the generation of taxonomic summary figures and Venn diagrams were completed in Excel.

*In silico* analyses of reference genomes for each of the mock community taxa were conducted to assess primer specificity and marker region length. Reference genomes were downloaded from NCBI RefSeq and GenBank databases^2^. Primer targets and their position within genome contig sequences were identified via the -search_oligodb command in usearch (allowing for up to two mismatches).

### Data Processing and Analyses

The discriminatory power of each method to amplify the target species and also classification accuracy for each ZOTU was first checked for all 21 taxa individually. This also enabled the inference of the relative abundance attributable to each taxon within the mixed mock community, based on which ZOTU was present in each of the individually sequenced taxon samples.

Zero-radius OTUs generated are incomparable between each of the target regions [given that the sequences (and therefore the generated ZOTUs) are different for each]. Remaining analyses and figures [including non-metric multidimensional scaling (nMDS) and taxa plots] were based on ZOTU tables reduced to unique taxonomic assignments (down to genus level). Ordering of samples in the mock community taxa plot was arranged by average linkage hierarchical clustering calculated in R (v3.3) (R Core Team, 2015) using the function hclust. nMDS plots were generated from taxonomically assigned tables in R based on weighted Bray–Curtis dissimilarities using the vegdist command from the vegan package (Oksanen et al., 2015).

Ethical approval for the human sinonasal aspect of this study was obtained from the New Zealand Health and Disability Ethics Committee (NTX/08/12/126), and written informed consent was obtained for all participants. Ethical approval for the mouse aspect of this study was obtained from the University of Auckland’s Animal Ethics Committee (AEC#1299).

## Results

### Sequencing of Individual Mock Community Taxa

Gel images of the PCR-amplified amplicons suggested that the target regions of some taxa amplified more easily than others when processed independently as pure isolates, and approximate amplicon sizes (including primers and Nextera adapters) ranged between 250–600 bp (ITS1), 300–650 bp (ITS2), 580–650 bp (SSU), and 650–750 bp (LSU) (**Supplementary Figure 1**). Ultimately, all four primer pairs were capable of amplifying and sequencing the target regions for each of the selected mock community taxa, and all met the subsampling threshold of 2000 reads/sample.

The mock community taxa generally fell into their own unique ZOTUs (based on 100% sequence similarity) in each of the ITS1, ITS2, and LSU data sets, indicating that most isolates had a unique sequence that differentiated them from one another. Exceptions included *Aspergillus brasiliensis* and *A. niger*, which shared identical sequences for ITS1, ITS2, and LSU regions, and *Fusarium solani* (ITS2), *Exophiala spinifera* (LSU), and *Candida tropicalis* (LSU), which were split across multiple ZOTUs. In these cases, each of these ZOTUs were nonetheless identically classified for each of the given taxa. For SSU, *C. albicans* and *C. parapsilosis* shared identical sequences, and *A. brasiliensis* was split across 20 ZOTUs (including various ZOTUs associated with most of the other mock community taxa) and had mixed classification. Sequences and pairwise alignments for each of the isolates, which had more than one unique sequence for each marker are provided in the **Supplementary Material** (**Supplementary Data Sheet 1**).

### Taxonomic Assignments

Classification for each of the 21 mock community taxa was first checked via the individually sequenced isolates. The accuracy of classification for each is indicated in **Figure 2B**, and full taxonomic assignments for each of the four amplicon regions and classification databases are given in more detail in **Supplementary Figure 1**. Overall, classifications based on ITS1 and ITS2 analyses were considerably more accurate than SSU or LSU marker analyses. SSU (SILVA database) and LSU (SILVA and RDP databases) analyses included several instances of incorrect classification at the level of class and/or only returned higher level classification. ITS2 with both UNITE and RDP databases and ITS1 with the RDP database all successfully classified 19 of the 21 taxa to the level of genus, and in several cases correctly to the level of species. Overall, both ITS1 and ITS2 were relatively comparable with respect to the accuracy of classification of individual taxa.

### Sequencing of the Mixed Mock Community

While the target regions for all taxa were successfully amplified as individual isolates, sequencing of the mixed mock community revealed pronounced differences in the relative representation of taxa between the different target regions.

In the analyses of the individually sequenced taxa, it was identified that for ITS1, ITS2, and LSU, all taxa except *A. brasiliensis* and *A. niger* (which were identified as sharing identical sequences for these regions) fell into distinct ZOTUs. This enabled the comparison of expected relative proportions for each of the 21 members of the mock community (expected based on even DNA proportions = 4.76% per species) to the actual relative sequence abundance of each taxon’s ZOTU when amplified and sequenced as a mixed community (**Figure 2C**). Estimations were not possible for SSU data due to *A. brasiliensis* containing multiple sequence variants identical to a number of other taxa from the mock community. *Trichosporon dermatis* and *C. albicans* were over-represented compared to the expected range by all three targets (*T. dermatis* ITS1 = 33% relative abundance, ITS2 = 22%, LSU = 18%; *C. albicans* ITS1 = 14%, ITS2 = 10%, LSU = 9%). *Yarrowia lipolytica* was over-represented in ITS2 (20%) and LSU (17%) data, but was absent from ITS1 data (0%). All three *Malassezia* species were markedly under-represented in both ITS1 and ITS2 data, and *M. furfur* and *M. pachydermatis* were also under-represented in LSU. Among the three mock community replicates for ITS1, only one sequence read in total was assigned to *Malassezia*. *Malassezia* spp. were also considerably under-represented by ITS2 (0.1–1.6% relative sequence abundance, compared to the expected 4.7% for each species), but to a lesser extent than ITS1.

**FIGURE 3:**
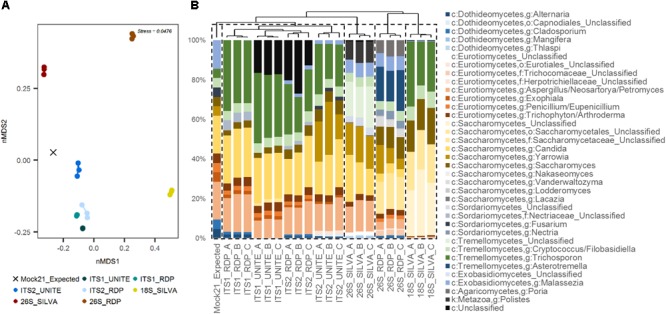
Human mycobiota mock community sequence data summary. **(A)** nMDS plot of replicates for each of the four target genomic regions [ITS1, ITS2, SSU (18S), LSU (26S)] and taxonomic reference databases (UNITE, RDP, SILVA) compared to the expected community structure (even distribution: “Mock21_Expected”), based on weighted Bray–Curtis dissimilarity. **(B)** Taxonomic summary plot for each of the replicates compared with the expected community structure, with samples ordered by average-linkage hierarchical clustering (represented by the dendrogram above). Dashed line boxes indicate partitioning of the samples into five clusters based on hierarchical clustering.

When hierarchically clustered mock community data were differentiated into five cluster groups, replicates for ITS1 and ITS2 (using either classification database) clustered together, and were more closely branching with the expected mock community than the SSU or LSU replicates (**Figure 3B**). LSU using the SILVA database was the next closest branching group, and replicates for LSU using RDP and SSU using SILVA each formed their own distantly branching clusters. These patterns were similarly reflected in nMDS analysis, where ITS replicates were more similar to the expected community composition than were SSU or LSU replicates (**Figure 3A**).

### *In silico* Analyses of the Mock Community Taxa

*In silico* analyses of reference genomes identified single- or multiple-base primer mismatches for several taxa. Two mismatches were identified between ITS1F and *Y. lipolytica*, as well as one mismatch with the primers ITS2 and ITS3. All three *Malassezia* spp. had one mismatch with the ITS2/ITS3 primers, but were perfect matches for ITS1F and ITS4 (with the exception of *M. furfur*, which also contained a single base mismatch with ITS4). *C. albicans* and *C. tropicalis* were perfect matches for each of the primers assessed. A complete list of primer target identification and mismatches is provided as a supplementary data file (**Supplementary Table 2**).

Marker lengths were largely comparable to PCR amplicon sizes noted above (taking into account additional amplicon length due to sequencing adapters). Markers ranged between 515–560 bp (SSU), 175–519 bp (ITS1) (with an additional outlier of 2177 bp for one ITS1 marker variant in *F. solani*), 239–556 bp (ITS2), and 547–643 bp (LSU). *Y. lipolytica* contained the shortest ITS markers (176 and 239 bp for ITS1 and ITS2, respectively). ITS2 markers for each of the *Malassezia* representatives were up to 50% larger (∼200 bp longer) than the other taxa assessed.

For some taxa, primer target sequences were not identified in *in silico* analyses, indicating that they either contained more than two mismatches with the primer sequences, or that currently available reference genomes were insufficiently complete and/or excluded the ribosomal cistron.

### Real-World Application: Human Upper Respiratory and Mouse Fecal Samples

Once filtering of non-target sequences was completed (which was particularly an issue for SSU data, which included a large proportion of non-target mouse or human sequences), very few usable sequences were retained for SSU (nasal median = 9 reads/sample; gut median = 15 reads/sample) and LSU (nasal median = 16858 reads/sample; gut median = 12 reads/sample). Those reads that remained gave generally poor resolution for classification. Due to this limitation, and in light of the findings from the mock community, the remaining analyses focused on the two ITS regions. For the purposes of these examples, RDP was chosen as the classification database for both ITS regions.

**FIGURE 4:**
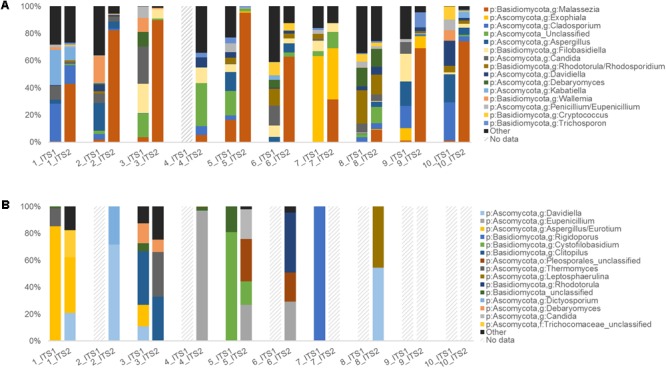
Human upper respiratory and mouse gut ITS data summaries. Taxonomic summary plots comparing ITS1 and ITS2 sequence data for **(A)** human sinonasal (*n* = 10) and **(B)** mouse fecal samples (*n* = 10), with taxonomic assignments conducted using the RDP ITS reference database. Dashed-line bars indicate samples that did not amplify sufficiently and/or meet the sequence subsampling threshold of 1000 reads per sample.

**FIGURE 5:**
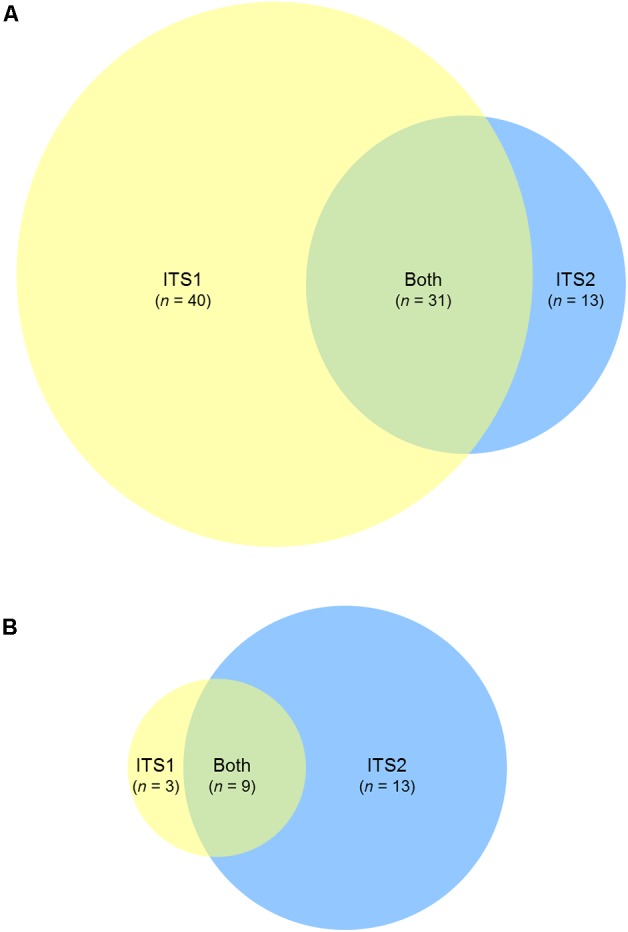
ITS1 and ITS2 fungal community coverage. Venn diagrams representing the number of unique taxonomic assignments identified by ITS1 only (yellow), ITS2 only (blue), or both (green), for **(A)** human sinonasal, and **(B)** mouse fecal samples. Taxonomic assignments were based on RDP ITS reference database. Full taxonomy lists used to generate the Venn diagrams are provided in **Supplementary Figure 2**.

Of the sinonasal samples, nine samples for ITS1 and all samples for ITS2 met the sequence subsampling threshold of 1000 reads per sample (**Figure 4A**). ITS2 was largely dominated by members of the genus *Malassezia* (median = 66% relative sequence abundance), however, this genus was in comparably low abundance in ITS1 data (median = 1%). The remainder of the dominant taxa was generally captured by both methods. Other main taxa identified included members of the genera *Filobasidiella, Rhodotorula, Candida, Debaryomyces, Aspergillus, Exophiala, Davidiella*, and *Cladosporium*. A greater range of taxa were identified solely by the ITS1 marker than by ITS2 exclusively or by both (**Figure 5A** and **Supplementary Figure 2A**). However, all 20 of the most abundant 20 taxa were identified by both target regions, albeit in markedly differing relative abundances.

For the mouse fecal samples, 4/10 ITS1 samples compared to 7/10 ITS2 samples met the sequence subsampling threshold. Overall, the 10 most abundant taxa identified in the mouse gut samples were *Davidiella, Eupenicillium, Aspergillus, Rigidoporus, Cystofilobasidium, Clitopilus*, unclassified members of the order *Pleosporales, Thermomyces, Leptosphaerulina*, and *Rhodotorula* (**Figure 4B**). Nine taxa were identified by both ITS1 and ITS2 markers, 13 were identified by ITS2 alone, and 3 were identified by ITS1 alone (**Figure 5B** and **Supplementary Figure 2B**).

## Discussion

Consistent selection of methodological approaches to characterize the human mycobiota is vital to enable more meaningful comparability between different studies. This will also ultimately allow for large retrospective meta-analyses, which have already proven invaluable for differentiating true patterns across bacterial microbiota studies from the inherent variability within individual studies comprising more limited sample sizes (Koren et al., 2013; Adams et al., 2015; Duvallet et al., 2017; Pammi et al., 2017; Wagner Mackenzie et al., 2017).

This study assessed four common genomic target regions used in human mycobiota studies for their ability to accurately characterize 21 fungal taxa previously identified as relevant members of mammalian mycobiota individually and as a mixed DNA community, as well as upper respiratory (human sinonasal swab) and gut (mouse fecal) samples.

### Mock Community Comparisons

Previous methodological comparisons have generally been centered on plant and soil-associated fungal communities (Mello et al., 2011; Ihrmark et al., 2012; Bazzicalupo et al., 2013; Blaalid et al., 2013; Tedersoo et al., 2015), and it remained unclear how these biases specifically affect efforts to characterize the taxonomic diversity typical of the human mycobiota. The results of this study further confirm the increased effectiveness of the ITS regions over the SSU and LSU targets for the characterization of complex human-associated fungal communities.

When each of the 21 mock community taxa were processed in isolation, all four target regions were capable of being amplified adequately and sequenced for all taxa. However, the accuracy of taxonomic classification for each of these regions was variable. SSU and LSU provided less resolution, as has been observed elsewhere (Schoch et al., 2012; Cui et al., 2013; Tedersoo et al., 2015; Halwachs et al., 2017; Nash et al., 2017), and also had a greater incidence of misclassification. Furthermore, sequencing analyses of the mixed mock community identified marked disparities between the target markers with respect to the relative representation of different taxa within the sequence data. Ultimately, ITS2 was determined as the optimal marker of those assessed here due to higher accuracy of taxonomic assignment of sequences and community structure representation.

Commonly used representative primer pairs were selected for each of the four target regions in order to assess the comparability and relative biases in human mycobiota studies thus far. However, this study is not exhaustive. Alternative primer sets are available for each region, including several refinements specifically designed to better capture a broader range of fungal taxa (Ihrmark et al., 2012; Tedersoo et al., 2015). The data here support the ITS2 marker as providing the most accurate representation. Future studies will identify whether alternative primer selections can further increase the accuracy of efforts to characterize the human mycobiota and pave the way for the establishment of a standardized methodology going forward.

### Community Structure Poorly Represented in All Cases

Relative sequence abundances in all cases poorly reflected the expected structure of the mock community. The accurate relative representation of fungal taxa within sequencing data may be influenced by a range of parameters, including primer sensitivity and binding imperfections, differences in amplicon length, differential PCR, or sequencing due to amplicon-specific inhibition, as well as marked differences in gene copy numbers and overall genome size. For example, two mismatches were identified between the ITS1F primer and *Y. lipolytica* in the *in silico* analyses of reference genomes, while the ITS2 marker was comparably shorter in length for this species than for other taxa. These mismatches may explain the under- and over-representation of this taxon in the ITS1 and ITS2 marker data, respectively. The comparably long ITS2 marker for all three *Malassezia* spp. may similarly explain the under-representation of these taxa in ITS2 data, due to preferential amplification and/or sequencing of taxa with shorter amplicons. In contrast, primer mismatches and amplicon length do not appear to explain the exclusion of *Malassezia* spp. from ITS1 marker data. Other factors, such as amplicon secondary structure, may be interfering with the amplification and ultimate detection of this taxa via the ITS1 marker. Furthermore, the ribosomal cistron can be repeated anywhere up to 100–200 times in a fungal genome, and this figure is highly variable for different taxa (Herrera et al., 2009; Lindahl et al., 2013; Tang et al., 2015; Diaz et al., 2017). Similarly, genome size can range anywhere from 2.2 to 3625 mb (Gregory et al., 2007; Xu et al., 2007; Mohanta and Bae, 2015; Egertová and Sochor, 2017). As such, the estimated relative abundances of the mixed mock community taxa (based on equal proportions of template DNA) are limited to the extent that different taxa may have markedly different ribosomal cistron copy numbers despite equal DNA concentrations. Findings based on divergence from the expected community structure here are to be viewed in light of this caveat. Nonetheless, the ITS2 marker clearly remains the strongest of the four candidate markers, given the poor resolution provided by the SSU and LSU markers and the omitted taxa in the ITS1 marker analyses.

As more complete genomes become available, better estimates of gene copy numbers for different fungal taxa should be possible. This will clarify the extent to which this aspect skews the structural representations of the fungal community, and will also better inform estimations of the true mock community structure. Furthermore, additional studies assessing the relative effect that differently staggered mock community structures (rather than even distribution) have on the overall community representation can provide further insight into dynamic changes observed within and between mycobiota communities. In the meantime, relative sequence abundances are unlikely to fully represent the true state of the fungal community, and should be treated with caution.

### Bioinformatics and Taxonomy Databases

Taxonomic databases for fungi have long been identified as a limitation of mycobiota studies (Cui et al., 2013; Tang et al., 2015; Diaz et al., 2017; Halwachs et al., 2017). However, improvements have been made more recently with the release of highly curated databases. This study compared several available databases for each of the target regions. The UNITE and RDP ITS databases were generally comparable (with the caveat that in some cases RDP returned teleomorph naming where UNITE returned the anamorph), and both were more optimal than the LSU- and SSU-based methods. Furthermore, both are already available in the correct format for use in usearch from the usearch website, whereas SILVA and RDP databases for LSU and SSU required considerable manual formatting and curation for the purposes of this study (at the time of writing, a formatted SSU database has also recently been made available on the usearch website). In future, updates to the UNITE and RDP ITS databases may ultimately enable both ITS1- and ITS2-based approaches to correctly classify a greater number of human-associated fungi to the level of genus (and in some cases, potentially to the level of species).

Finally, while the focus of this study has been to assess the selection of target ribosomal markers in human mycobiota studies, as recently highlighted (Diaz et al., 2017; Halwachs et al., 2017), downstream bioinformatics processing pipelines also require further attention. Standard 16S rRNA gene processing pipelines applied as-is can generate erroneous results, and for this study customized scripts were developed to handle some aspects of these processing requirements that are not yet available in usearch. Comparative assessment of the available pipelines and further development in this area is warranted. For example, merged paired-end sequence reads offer a greater potential for reduced error due to confirmatory reads in the overlap, and increased taxonomic resolution due to longer read length. To ensure that no taxa were omitted due to the potential for ITS regions to be longer than the possible merged paired-end sequencing length, only forward reads were processed in this study. Notably, accurate genus-level (and in some cases species-level) identification was still achieved for the majority of the mock community taxa, despite limiting the analysis to single-ended 250 bp reads. Furthermore, a preliminary comparison processing the data via merging paired reads (in lieu of the single-read-specific trimming steps) identified that several taxa would be excluded from the mock community data due to incompatibilities with merging (such as amplicons longer than the maximum merge length). However, further characterization of the possible range of marker region lengths, the use of longer read lengths [such as the MiSeq 2 × 300 bp (600 cycle) kit], and the development of primer pairs that target a region known to be shorter than the possible sequence read overlap in all or most taxa would enable increased confidence in the standard use of merged paired-end reads in mycobiota studies. Importantly, acquiring sequence data that most accurately reflect the real-world community is the first step in the chain. Understanding the biases in methods used to date and establishing consistency in this respect will enable the re-analysis and comparison of sequence data should further developments in bioinformatics processing warrant it.

### Respiratory and Gut Samples

Results from the limited studies of the mycobiota of the upper respiratory tract thus far have been conflicting. While some have identified *Malassezia* as a dominant taxon (Cleland et al., 2014; Gelber et al., 2016), other studies have identified others (such as *Cryptococcus* spp.) as dominant, and *Malassezia* in much lower abundance (Aurora et al., 2013). Here, ITS2 results highlighted a very dominant proportion of *Malassezia* spp. in most subjects. Furthermore, the mock community analyses identified that both ITS markers resulted in a marked under-representation of *Malassezia* spp. (particularly for ITS1). This would suggest that the dominance of *Malassezia* spp. within these sinonasal communities is even more pronounced than identified here. These results confirm the likely importance and dominance of *Malassezia* species in these fungal communities and indicate that the choice of methodological approach is likely a major determinant of the conflicting results in studies to date. Overall, given the importance of *Malassezia* in these communities, these data suggest that targeting the ITS2 marker is the most appropriate of the four methods tested here for these types of communities.

In the case of the gut data, targeting of the ITS2 marker successfully obtained data for 7/10 samples compared to 4/10 for ITS1, and the majority of taxa were identified by the ITS2 marker, further supporting its use. These observations are hindered by the limited data and uneven sample numbers available for analysis for each group, however. Additional optimization of DNA extraction and/or amplification conditions may be required to characterize a greater proportion of the fungal communities of fecal samples. Ultimately, while the ITS2 marker was deemed optimal in this study of a mock community, upper respiratory and gut samples, biases were observed in all approaches. Further pilot studies may therefore be warranted to determine whether this ITS2 approach is similarly optimal for other body environments and taxa of interest, and the extent to which more recent primer alternatives further advance the accuracy of our understanding of the human mycobiota.

## Summary and Concluding Remarks

In isolation, all four target regions for each of the 21 fungal mock community taxa were capable of being amplified adequately and sequenced, however, ITS1 and ITS2 outperformed LSU and SSU with respect to taxonomic classifications. Mixed mock community analyses revealed marked variability in the ability of each method to accurately characterize a mixed community. ITS regions again outperformed LSU and SSU data. Of the ITS regions, ITS1 failed to generate sequences for *Y. lipolytica* and all three *Malassezia* species when in a mixed community. These findings were further supported in pilot studies of upper-respiratory (human sinonasal swab) and gut (mouse fecal) samples. Based on these analyses, previous studies using ITS1, SSU, or LSU markers may omit key taxa that are identified by the ITS2 marker. Of methods commonly used in human mycobiota studies to date, we recommend the selection of the ITS2 target marker assessed in this study. Further investigation of more recently developed fungal primer options will be essential to ultimately determine the optimal methodological approach by which future human mycobiota studies ought to be standardized.

## Author Contributions

MH was involved in study design, sample processing of mock community and sinonasal samples, data analyses, and writing of the manuscript. MT contributed to study design and editing of the manuscript, and provided laboratory space and materials. AV contributed to acquiring and culturing mock community isolates. JM and CF provided mouse fecal samples. GW processed and prepared fecal samples for sequencing. RD collected sinonasal swab samples. KB contributed to editing of the manuscript. All authors read and approved the final manuscript.

## Conflict of Interest Statement

The authors declare that the research was conducted in the absence of any commercial or financial relationships that could be construed as a potential conflict of interest.

## Footnotes

1 https://github.com/mhog025/Microbiota-amplicon-bioinformatics

2 ftp://ftp.ncbi.nih.gov/genomes/

## References

[B1] Adams R. I. Bateman A. C. Bik H. M. Meadow J. F. (2015). Microbiota of the indoor environment: a meta-analysis. *Microbiome* 3 1–18. 10.1186/s40168-015-0108-3 26459172 PMC4604073

[B2] Agbetile J. Fairs A. Desai D. Hargadon B. Bourne M. Mutalithas K. (2012). Isolation of filamentous fungi from sputum in asthma is associated with reduced post-bronchodilator FEV1. *Clin. Exp. Allergy* 42 782–791. 10.1111/j.1365-2222.2012.03987.x 22515394 PMC3509218

[B3] Amann R. I. Binder B. J. Olson R. J. Chisholm S. W. Devereux R. Stahl D. A. (1990). Combination of 16S rRNA-Targeted oligonucleotide probes with flow cytometry for analyzing mixed microbial populations. *Appl. Environ. Microbiol.* 56 1919–1925. 2200342 10.1128/aem.56.6.1919-1925.1990 PMC184531

[B4] Aurora R. Chatterjee D. Hentzleman J. Prasad G. Sindwani R. Sanford T. (2013). Contrasting the microbiomes from healthy volunteers and patients with chronic rhinosinusitis. *JAMA Otolaryngol. Head Neck Surg.* 139 1328–1338. 10.1001/jamaoto.2013.5465 24177790

[B5] Bazzicalupo A. L. Bálint M. Schmitt I. (2013). Comparison of ITS1 and ITS2 rDNA in 454 sequencing of hyperdiverse fungal communities. *Fungal Ecol.* 6 102–109. 10.1016/j.funeco.2012.09.003

[B6] Bellemain E. Carlsen T. Brochmann C. Coissac E. Taberlet P. Kauserud H. (2010). ITS as an environmental DNA barcode for fungi: an in silico approach reveals potential PCR biases. *BMC Microbiol.* 10:189. 10.1186/1471-2180-10-189 20618939 PMC2909996

[B7] Bittinger K. Charlson E. S. Loy E. Shirley D. J. Haas A. R. Laughlin A. (2014). Improved characterization of medically relevant fungi in the human respiratory tract using next-generation sequencing. *Genome Biol.* 15 1–14. 10.1186/s13059-014-0487-y 25344286 PMC4232682

[B8] Blaalid R. Kumar S. Nilsson R. H. Abarenkov K. Kirk P. M. Kauserud H. (2013). ITS1 versus ITS2 as DNA metabarcodes for fungi. *Mol. Ecol. Resour.* 13 218–224. 10.1111/1755-0998.12065 23350562

[B9] Botschuijver S. Roeselers G. Levin E. Jonkers D. M. Welting O. Heinsbroek S. E. M. (2017). Intestinal fungal dysbiosis is associated with visceral hypersensitivity in patients with irritable bowel syndrome and rats. *Gastroenterology* 153 1026–1039. 10.1053/j.gastro.2017.06.004 28624575

[B10] Brown G. D. Denning D. W. Gow N. A. R. Levitz S. M. Netea M. G. White T. C. (2012). Hidden killers: human fungal infections. *Sci. Transl. Med.* 4:165rv13. 10.1126/scitranslmed.3004404 23253612

[B11] Charlson E. S. Diamond J. M. Bittinger K. Fitzgerald A. S. Yadav A. Haas A. R. (2012). Lung-enriched organisms and aberrant bacterial and fungal respiratory microbiota after lung transplant. *Am. J. Respir. Crit. Care Med.* 186 536–545. 10.1164/rccm.201204-0693OC 22798321 PMC3480531

[B12] Cho I. Blaser M. J. (2012). The human microbiome: at the interface of health and disease. *Nat. Rev. Genet.* 13 260–270. 10.1038/nrg3182 22411464 PMC3418802

[B13] Chotirmall S. H. McElvaney N. G. (2014). Fungi in the cystic fibrosis lung: bystanders or pathogens? *Int. J. Biochem. Cell Biol.* 52 161–173. 10.1016/j.biocel.2014.03.001 24625547

[B14] Cleland E. J. Bassioni A. Boase S. Dowd S. Vreugde S. Wormald P. J. (2014). The fungal microbiome in chronic rhinosinusitis: richness, diversity, postoperative changes and patient outcomes. *Int. Forum Allergy Rhinol.* 4 259–265. 10.1002/alr.21297 24500871

[B15] Cole J. R. Wang Q. Fish J. A. Chai B. McGarrell D. M. Sun Y. (2014). Ribosomal database project: data and tools for high throughput rRNA analysis. *Nucleic Acids Res.* 42 D633–D642. 10.1093/nar/gkt1244 24288368 PMC3965039

[B16] Comacle P. Belaz S. Jegoux F. Ruaux C. Le Gall F. Gangneux J. P. (2016). Contribution of molecular tools for the diagnosis and epidemiology of fungal chronic rhinosinusitis. *Med. Mycol.* 54 794–800. 10.1093/mmy/myw041 27335058

[B17] Cope E. K. Goldberg A. N. Pletcher S. D. Lynch S. V. (2017). Compositionally and functionally distinct sinus microbiota in chronic rhinosinusitis patients have immunological and clinically divergent consequences. *Microbiome* 5:53. 10.1186/s40168-017-0266-6 28494786 PMC5427582

[B18] Cottier F. Srinivasan K. G. Yurieva M. Liao W. Poidinger M. Zolezzi F. (2018). Advantages of meta-total RNA sequencing (MeTRS) over shotgun metagenomics and amplicon-based sequencing in the profiling of complex microbial communities. *NPJ Biofilms Microbiomes* 4:2. 10.1038/s41522-017-0046-x 29367879 PMC5773663

[B19] Cryan J. F. Dinan T. G. (2012). Mind-altering microorganisms: the impact of the gut microbiota on brain and behaviour. *Nat. Rev. Neurosci.* 13 701–712. 10.1038/nrn3346 22968153

[B20] Cui L. Morris A. Ghedin E. (2013). The human mycobiome in health and disease. *Genome Med.* 5 1–12. 10.1186/gm467 23899327 PMC3978422

[B21] Delhaes L. Monchy S. Fréalle E. Hubans C. Salleron J. Leroy S. (2012). The airway microbiota in cystic fibrosis: a complex fungal and bacterial community – Implications for therapeutic management. *PLoS One* 7:e36313. 10.1371/journal.pone.0036313 22558432 PMC3338676

[B22] Diaz P. I. Hong B.-Y. Dupuy A. K. Strausbaugh L. D. (2017). Mining the oral mycobiome: methods, components, and meaning. *Virulence* 8 313–323. 10.1080/21505594.2016.1252015 27791473 PMC5411240

[B23] Dupuy A. K. David M. S. Li L. Heider T. N. Peterson J. D. Montano E. A. (2014). Redefining the human oral mycobiome with improved practices in amplicon-based taxonomy: discovery of *Malassezia* as a prominent commensal. *PLoS One* 9:e90899. 10.1371/journal.pone.0090899 24614173 PMC3948697

[B24] Duvallet C. Gibbons S. M. Gurry T. Irizarry R. A. Alm E. J. (2017). Meta-analysis of gut microbiome studies identifies disease-specific and shared responses. *Nat. Commun.* 8 1–10. 10.1038/s41467-017-01973-8 29209090 PMC5716994

[B25] Edgar R. C. (2010). Search and clustering orders of magnitude faster than BLAST. *Bioinformatics* 26 2460–2461. 10.1093/bioinformatics/btq461 20709691

[B26] Edgar R. C. (2016a). SINTAX: a simple non-Bayesian taxonomy classifier for 16S and ITS sequences. *bioRxiv* [Preprint] 10.1101/074161

[B27] Edgar R. C. (2016b). UNOISE2: improved error-correction for Illumina 16S and ITS amplicon sequencing. *bioRxiv* [Preprint] 10.1101/081257

[B28] Edgar R. C. (2017). Accuracy of microbial community diversity estimated by closed- and open-reference OTUs. *Peer J.* 5:e3889. 10.7717/peerj.3889 29018622 PMC5631090

[B29] Edgar R. C. Flyvbjerg H. (2015). Error filtering, pair assembly and error correction for next-generation sequencing reads. *Bioinformatics* 31 3476–3482. 10.1093/bioinformatics/btv401 26139637

[B30] Egertová Z. Sochor M. (2017). The largest fungal genome discovered in Jafnea semitosta. *Plant Syst. Evol.* 303 981–986. 10.1007/s00606-017-1424-9

[B31] Eickmeier O. Hector A. Singh A. Chotirmall S. H. Hartl D. (2015). Fungi in cystic fibrosis: recent findings and unresolved questions. *Curr. Fungal Infect. Rep.* 9 1–5. 10.1007/s12281-014-0211-0

[B32] Findley K. Oh J. Yang J. Conlan S. Deming C. Meyer J. A. (2013). Topographic diversity of fungal and bacterial communities in human skin. *Nature* 498 367–370. 10.1038/nature12171 23698366 PMC3711185

[B33] Gao R. Kong C. Li H. Huang L. Qu X. Qin N. (2017). Dysbiosis signature of mycobiota in colon polyp and colorectal cancer. *Eur. J. Clin. Microbiol. Infect. Dis.* 36 2457–2468. 10.1007/s10096-017-3085-6 28821976

[B34] Gardes M. Bruns T. D. (1993). ITS primers with enhanced specificity for basidiomycetes – Application to the identification of mycorrhizae and rusts. *Mol. Ecol.* 2 113–118. 10.1111/j.1365-294X.1993.tb00005.x 8180733

[B35] Gelber J. T. Cope E. K. Goldberg A. N. Pletcher S. D. (2016). Evaluation of malassezia and common fungal pathogens in subtypes of chronic rhinosinusitis. *Int. Forum Allergy Rhinol.* 6 950–955. 10.1002/alr.21777 27153455

[B36] Gerard R. Sendid B. Colombel J. F. Poulain D. Jouault T. (2015). An immunological link between *Candida albicans* colonization and Crohn’s disease. *Crit. Rev. Microbiol.* 41 135–139. 10.3109/1040841X.2013.810587 23855357

[B37] Ghannoum M. A. Jurevic R. J. Mukherjee P. K. Cui F. Sikaroodi M. Naqvi A. (2010). Characterization of the oral fungal microbiome (mycobiome) in healthy individuals. *PLoS Pathog.* 6:e1000713. 10.1371/journal.ppat.1000713 20072605 PMC2795202

[B38] Gregory T. R. Nicol J. A. Tamm H. Kullman B. Kullman K. Leitch I. J. (2007). Eukaryotic genome size databases. *Nucleic Acids Res.* 35 D332–D338. 10.1093/nar/gkl828 17090588 PMC1669731

[B39] Hajishengallis G. Lambris J. D. (2012). Complement and dysbiosis in periodontal disease. *Immunobiology* 217 1111–1116. 10.1016/j.imbio.2012.07.007 22964237 PMC3439646

[B40] Hallen-Adams H. E. Suhr M. J. (2017). Fungi in the healthy human gastrointestinal tract. *Virulence* 8 352–358. 10.1080/21505594.2016.1247140 27736307 PMC5411236

[B41] Halwachs B. Madhusudhan N. Krause R. Nilsson R. H. Moissl-Eichinger C. Högenauer C. (2017). Critical issues in mycobiota analysis. *Front. Microbiol.* 8:180. 10.3389/fmicb.2017.00180 28261162 PMC5306204

[B42] Hamad I. Ranque S. Azhar E. I. Yasir M. Jiman-Fatani A. A. Tissot-Dupont H. (2017). Culturomics and amplicon-based metagenomic approaches for the study of fungal population in human gut microbiota. *Sci. Rep.* 7 1–8. 10.1038/s41598-017-17132-4 29196717 PMC5711903

[B43] Herrera M. L. Vallor A. C. Gelfond J. A. Patterson T. F. Wickes B. L. (2009). Strain-dependent variation in 18S ribosomal DNA copy numbers in *Aspergillus fumigatus*. *J. Clin. Microbiol.* 47 1325–1332. 19261786 10.1128/JCM.02073-08 PMC2681831

[B44] Hoarau G. Mukherjee P. K. Gower-Rousseau C. Hager C. Chandra J. Retuerto M. A. (2016). Bacteriome and mycobiome interactions underscore microbial dysbiosis in familial Crohn’s disease. *MBio* 7:e1250-16. 10.1128/mBio.01250-16 27651359 PMC5030358

[B45] Hoggard M. Biswas K. Zoing M. Wagner Mackenzie B. Taylor M. W. Douglas R. G. (2017a). Evidence of microbiota dysbiosis in chronic rhinosinusitis. *Int. Forum Allergy Rhinol.* 7 230–239. 10.1002/alr.21871 27879060

[B46] Hoggard M. Wagner Mackenzie B. Jain R. Taylor M. W. Biswas K. Douglas R. G. (2017b). Chronic rhinosinusitis and the evolving understanding of microbial ecology in chronic inflammatory mucosal disease. *Clin. Microbiol. Rev.* 30 321–348. 10.1128/CMR.00060-16 27903594 PMC5217796

[B47] Hoggard M. Nocera A. Biswas K. Taylor M. W. Douglas R. G. Bleier B. S. (2018). The sinonasal microbiota, neural signaling, and depression in chronic rhinosinusitis. *Int. Forum Allergy Rhinol.* 8 394–405. 10.1002/alr.22074 29278464

[B48] Huffnagle G. B. Noverr M. C. (2013). The emerging world of the fungal microbiome. *Trends Microbiol.* 21 334–341. 10.1016/j.tim.2013.04.002 23685069 PMC3708484

[B49] Huseyin C. E. Rubio R. C. O’Sullivan O. Cotter P. D. Scanlan P. D. (2017). The fungal frontier: a comparative analysis of methods used in the study of the human gut mycobiome. *Front. Microbiol.* 8:1432. 10.3389/fmicb.2017.01432 28824566 PMC5534473

[B50] Ihrmark K. Bödeker I. T. M. Cruz-Martinez K. Friberg H. Kubartova A. Schenck J. (2012). New primers to amplify the fungal ITS2 region – Evaluation by 454-sequencing of artificial and natural communities. *FEMS Microbiol. Ecol.* 82 666–677. 10.1111/j.1574-6941.2012.01437.x 22738186

[B51] Jung W. H. Croll D. Cho J. H. Kim Y. R. Lee Y. W. (2015). Analysis of the nasal vestibule mycobiome in patients with allergic rhinitis. *Mycoses* 58 167–172. 10.1111/myc.12296 25675851

[B52] Kim Y.-G. Udayanga K. G. S. Totsuka N. Weinberg J. B. Núñez G. Shibuya A. (2014). Gut dysbiosis promotes M2 macrophage polarization and allergic airway inflammation via fungi-induced PGE2. *Cell Host Microbe* 15 95–102. 10.1016/j.chom.2013.12.010 24439901 PMC3957200

[B53] Koren O. Knights D. Gonzalez A. Waldron L. Segata N. Knight R. (2013). A guide to enterotypes across the human body: meta-analysis of microbial community structures in human microbiome datasets. *PLoS Comput. Biol.* 9:e1002863. 10.1371/journal.pcbi.1002863 23326225 PMC3542080

[B54] Li Q. Wang C. Tang C. He Q. Li N. Li J. (2014). Dysbiosis of gut fungal microbiota is associated with mucosal inflammation in crohn’s disease. *J. Clin. Gastroenterol.* 48 513–523. 10.1097/MCG.0000000000000035 24275714 PMC4059552

[B55] Liguori G. Lamas B. Richard M. L. Brandi G. da Costa G. Hoffmann T. W. (2016). Fungal dysbiosis in mucosa-associated microbiota of Crohn’s disease patients. *J. Crohn’s Colitis* 10 296–305. 10.1093/ecco-jcc/jjv209 26574491 PMC4957473

[B56] Lindahl B. D. Nilsson R. H. Tedersoo L. Abarenkov K. Carlsen T. Kjøller R. (2013). Fungal community analysis by high-throughput sequencing of amplified markers – A user’s guide. *New Phytol.* 199 288–299. 10.1111/nph.12243 23534863 PMC3712477

[B57] Liu J. C. Modha D. E. Gaillard E. A. (2013). What is the clinical significance of filamentous fungi positive sputum cultures in patients with cystic fibrosis? *J. Cyst. Fibros.* 12 187–193. 10.1016/j.jcf.2013.02.003 23491855

[B58] Mello A. Napoli C. Murat C. Morin E. Marceddu G. Bonfante P. (2011). ITS-1 versus ITS-2 pyrosequencing: a comparison of fungal populations in truffle grounds. *Mycologia* 103 1184–1193. 10.3852/11-027 21700633

[B59] Mohanta T. K. Bae H. (2015). The diversity of fungal genome. *Biol. Proced. Online* 17 1–9. 10.1186/s12575-015-0020-z 25866485 PMC4392786

[B60] Motooka D. Fujimoto K. Tanaka R. Yaguchi T. Gotoh K. Maeda Y. (2017). Fungal ITS1 deep-sequencing strategies to reconstruct the composition of a 26-species community and evaluation of the gut mycobiota of healthy Japanese individuals. *Front. Microbiol.* 8:238. 10.3389/fmicb.2017.00238 28261190 PMC5309391

[B61] Nash A. K. Auchtung T. A. Wong M. C. Smith D. P. Gesell J. R. Ross M. C. (2017). The gut mycobiome of the human microbiome project healthy cohort. *Microbiome* 5 1–13. 10.1186/s40168-017-0373-4 29178920 PMC5702186

[B62] Nguyen L. D. N. Viscogliosi E. Delhaes L. (2015). The lung mycobiome: an emerging field of the human respiratory microbiome. *Front. Microbiol.* 6:89. 10.3389/fmicb.2015.00089 25762987 PMC4327734

[B63] Nicholson J. K. Holmes E. Kinross J. Burcelin R. Gibson G. Jia W. (2012). Host-gut microbiota metabolic interactions. *Science* 336 1262–1267. 10.1126/science.1223813 22674330

[B64] Nylund L. Nermes M. Isolauri E. Salminen S. de Vos W. M. Satokari R. (2015). Severity of atopic disease inversely correlates with intestinal microbiota diversity and butyrate-producing bacteria. *Allergy Eur. J. Allergy Clin. Immunol.* 70 241–244. 10.1111/all.12549 25413686

[B65] O’Donnell K. (1992). Ribosomal DNA internal transcribed spacers are highly divergent in the phytopathogenic ascomycete *Fusarium sambucinum* (*Gibberella pulicaris*). *Curr. Genet.* 22 213–220. 10.1007/BF00351728 1525873

[B66] Oever J. T. Netea M. G. (2014). The bacteriome-mycobiome interaction and antifungal host defense. *Eur. J. Immunol.* 44 3182–3191. 10.1002/eji.201344405 25256886

[B67] Oksanen J. Blanchet F. G. Kindt R. Legendre P. Minchin P. R. O’Hara R. B. (2015). *Vegan Community Ecology Package. R Package Version 2.3.2.* Available at: https://cran.r-project.org; https://github.com/vegandevs/vegan

[B68] Pammi M. Cope J. Tarr P. I. Warner B. B. Morrow A. L. Mai V. (2017). Intestinal dysbiosis in preterm infants preceding necrotizing enterocolitis: a systematic review and meta-analysis. *Microbiome* 5 1–15. 10.1186/s40168-017-0248-8 28274256 PMC5343300

[B69] Park H. K. Ha M. H. Park S. G. Kim M. N. Kim B. J. Kim W. (2012). Characterization of the fungal microbiota (mycobiome) in healthy and dandruff-afflicted human scalps. *PLoS One* 7:e32847. 10.1371/journal.pone.0032847 22393454 PMC3290624

[B70] Pashley C. H. (2014). Fungal culture and sensitisation in asthma, cystic fibrosis and chronic obstructive pulmonary disorder: what does it tell us? *Mycopathologia* 178 457–463. 10.1007/s11046-014-9804-y 25151366

[B71] Pourfathollah A. A. Beyzayi F. Khodadadi A. Athari S. S. (2014). General overview of fungal allergic asthma. *J. Mycol. Res.* 1 35–41.

[B72] Quast C. Pruesse E. Yilmaz P. Gerken J. Schweer T. Yarza P. (2013). The SILVA ribosomal RNA gene database project: improved data processing and web-based tools. *Nucleic Acids Res.* 41 D590–D596. 10.1093/nar/gks1219 23193283 PMC3531112

[B73] R Core Team (2015). *R: A Language and Environment for Statistical Computing.* Geneva: R Foundation for Statistical Computing.

[B74] Rizzetto L. De Filippo C. Cavalieri D. (2014). Richness and diversity of mammalian fungal communities shape innate and adaptive immunity in health and disease. *Eur. J. Immunol.* 44 3166–3181. 10.1002/eji.201344403 25257052

[B75] Saxena A. Sitaraman R. (2014). Osmoregulation and the human mycobiome. *Front. Microbiol.* 5:167. 10.3389/fmicb.2014.00167 24860554 PMC4028996

[B76] Schei K. Avershina E. Øien T. Rudi K. Follestad T. Salamati S. (2017). Early gut mycobiota and mother-offspring transfer. *Microbiome* 5 1–12. 10.1186/s40168-017-0319-x 28837002 PMC5571498

[B77] Schoch C. L. Seifert K. A. Huhndorf S. Robert V. Spouge J. L. Levesque C. A. (2012). Nuclear ribosomal internal transcribed spacer (ITS) region as a universal DNA barcode marker for Fungi. *Proc. Natl. Acad. Sci.* *U.S.A.* 109 6241–6246. 10.1073/pnas.1117018109 22454494 PMC3341068

[B78] Sharpe R. A. Bearman N. Thornton C. R. Husk K. Osborne N. J. (2015). Indoor fungal diversity and asthma: a meta-analysis and systematic review of risk factors. *J. Allergy Clin. Immunol.* 135 110–122. 10.1016/j.jaci.2014.07.002 25159468

[B79] Sogin M. L. Gunderson J. H. (1987). Structural diversity of eukaryotic small subunit ribosomal RNAs: evolutionary implications. *Ann. N. Y. Acad. Sci.* 503 125–139. 10.1111/j.1749-6632.1987.tb40603.x 3304074

[B80] Strati F. Di Paola M. Stefanini I. Albanese D. Rizzetto L. Lionetti P. (2016). Age and gender affect the composition of fungal population of the human gastrointestinal tract. *Front. Microbiol.* 7:1227 10.3389/fmicb.2016.01227 PMC4971113 27536299

[B81] Tang J. Iliev I. D. Brown J. Underhill D. M. Funari V. A. (2015). Mycobiome: approaches to analysis of intestinal fungi. *J. Immunol. Methods* 421 112–121. 10.1016/j.jim.2015.04.004 25891793 PMC4451377

[B82] Tedersoo L. Anslan S. Bahram M. Põlme S. Riit T. Liiv I. (2015). Shotgun metagenomes and multiple primer pair-barcode combinations of amplicons reveal biases in metabarcoding analyses of fungi. *MycoKeys* 10 1–43. 10.3897/mycokeys.10.4852

[B83] Underhill D. M. Iliev I. D. (2014). The mycobiota: interactions between commensal fungi and the host immune system. *Nat. Rev. Immunol.* 14 405–416. 10.1038/nri3684 24854590 PMC4332855

[B84] UNITE Community (2017). *UNITE USEARCH/UTAX Release.* London: UNITE Community 10.15156/BIO/587476

[B85] van Woerden H. C. Gregory C. Brown R. Marchesi J. R. Hoogendoorn B. Matthews I. P. (2013). Differences in fungi present in induced sputum samples from asthma patients and non-atopic controls: a community based case control study. *BMC Infect. Dis.* 13:69. 10.1186/1471-2334-13-69 23384395 PMC3570489

[B86] Vesty A. Biswas K. Taylor M. W. Gear K. Douglas R. G. (2017). Evaluating the impact of DNA extraction method on the representation of human oral bacterial and fungal communities. *PLoS One* 12:e0169877. 10.1371/journal.pone.0169877 28099455 PMC5242530

[B87] Vujkovic-Cvijin I. Dunham R. M. Iwai S. Maher M. C. Albright R. G. Broadhurst M. J. (2013). Dysbiosis of the gut microbiota is associated with HIV disease progression and tryptophan catabolism. *Sci. Transl. Med.* 5:193ra91. 10.1126/scitranslmed.3006438 23843452 PMC4094294

[B88] Wagner Mackenzie B. Waite D. W. Hoggard M. Douglas R. G. Taylor M. W. Biswas K. (2017). Bacterial community collapse: a meta-analysis of the sinonasal microbiota in chronic rhinosinusitis. *Environ. Microbiol.* 19 381–392. 10.1111/1462-2920.13632 27902866

[B89] Wang X. Fu Y. F. Wang R. Y. Li L. Cao Y. H. Chen Y. Q. (2014). Identification of clinically relevant fungi and *Prototheca* species by rRNA gene sequencing and multilocus PCR coupled with electrospray ionization mass spectrometry. *PLoS One* 9:e98110. 10.1371/journal.pone.0098110 24835205 PMC4024029

[B90] Wang Z. K. Yang Y. S. Stefka A. T. Sun G. Peng L. H. (2014). Review article: fungal microbiota and digestive diseases. *Aliment. Pharmacol. Ther.* 39 751–766. 10.1111/apt.12665 24612332

[B91] White T. J. Bruns T. D. Lee S. Taylor J. W. (1990). “Amplification and direct sequencing of fungal ribosomal RNA genes for phylogenetics,” in *PCR Protocols: A Guide to Methods and Applications* eds Innis M. A. Gelfand D. H. Sninsky J. J. White T. J. (New York, NY: Academic) 315–322.

[B92] Willger S. D. Grim S. L. Dolben E. L. Shipunova A. Hampton T. H. Morrison H. G. (2014). Characterization and quantification of the fungal microbiome in serial samples from individuals with cystic fibrosis. *Microbiome* 2 1–15. 10.1186/2049-2618-2-40 25408892 PMC4236224

[B93] Xu J. Saunders C. W. Hu P. Grant R. A. Boekhout T. Kuramae E. E. (2007). Dandruff-associated *Malassezia* genomes reveal convergent and divergent virulence traits shared with plant and human fungal pathogens. *Proc. Natl. Acad. Sci. U.S.A.* 104 18730–18735. 10.1073/pnas.0706756104 18000048 PMC2141845

[B94] Zhang E. Tanaka T. Tajima M. Tsuboi R. Nishikawa A. Sugita T. (2011). Characterization of the skin fungal microbiota in patients with atopic dermatitis and in healthy subjects. *Microbiol. Immunol.* 55 625–632. 10.1111/j.1348-0421.2011.00364.x 21699559

[B95] Zhang I. Pletcher S. D. Goldberg A. N. Barker B. M. Cope E. K. (2017). Fungal microbiota in chronic airway inflammatory disease and emerging relationships with the host immune response. *Front. Microbiol.* 8:2477. 10.3389/fmicb.2017.02477 29312187 PMC5733051

[B96] Zhao Y. C. Bassiouni A. Tanjararak K. Vreugde S. Wormald P.-J. Psaltis A. J. (2017). Role of fungi in chronic rhinosinusitis through ITS sequencing. *Laryngoscope* 128 16–22. 10.1002/lary.26702 28675446

