# Supplementary material for: Characterizing the Human Mycobiota: A Comparison of Small Subunit rRNA, ITS1, ITS2, and Large Subunit rRNA Genomic Targets
Source: Front Microbiol. 2018 Sep 19;9:2208. doi: 10.3389/fmicb.2018.02208 (PMC6157398; doi:10.3389/fmicb.2018.02208)
Supplement: Supplementary file 1 [file Table_1.pdf]

Supplementary Table 1. Retained sequence reads for individual mock community isolates based on merged paired-end reads processing vs. the bioinformatics pipeline used in this study (forward reads only)*

			Sequence reads (merged pipeline)**				Sequence reads (forward reads only)**			
			ITS1	ITS2	SSU	LSU	ITS1	ITS2	SSU	LSU
Ascomycota	Dothideomycetes	*Alternaria alternata*	89341	114073	9	7	265551	205917	41053	15088
		Cladosporium sphaeospermum	54967	21013	3	9	90956	83206	26587	31306
	Eurotiomycetes	*Aspergillus brasiliensis*	2378	8384	10	95	124246	118078	27452	32185
		Aspergillus flavus	3230	28976	32	9	122954	119969	43221	20589
		Aspergillus fumigatus	23	15445	24	5	79575	70330	49896	21851
		Aspergillus niger	2809	13602	9	8	126611	58681	48695	18224
		Exophiala spinifera	18529	60048	7	33	41316	105446	42554	21076
		Penicillium chrysogenum	1671	52857	5	10	235040	136974	20113	37680
		Trichophyton mentagrophytes	31	41651	4	30	177433	124798	19897	19945
	Saccharomycetes	*Candida albicans*	184891	31017	20	22	218982	48410	47027	28812
		Candida glabrata	58	18510	8	21	39969	26062	37351	11366
		Candida parapsilosis	139502	76934	16	5	167202	106998	38019	34513
		Candida tropicalis	89415	71195	9	7	107748	101414	26691	34239
		Yarrowia lipolytica	141177	155283	24	26	5308	173674	23499	23947
		Saccharomyces cerevisiae	58104	52629	1	35	96085	65569	14141	34961
	Sordariomycetes	*Fusarium solani*	16579	14	3	2	71390	148863	34640	27017
Basidiomycota	Tremellomycetes	*Cryptococcus neoformans*	74547	36599	16	7	85571	53422	33871	22269
		Trichosporon dermatis	150989	65057	4	21	150269	83533	25508	31947
	Exobasidiomycetes	*Malassezia furfur*	9401	9	9	17	16395	30689	41570	28938
		Malassezia globosa	31529	17893	6	24	46780	36685	31304	21620
		Malassezia pachydermatis	16674	7	4	8	27286	43115	22967	35618

*Merged reads processing was via the same bioinformatics pipeline as that used in this study, with the following alterations: primer binding regions were trimmed and forward and reverse reads were merged (via -fastq_mergepairs in usearch), and all single-read-specific processing was omitted (incl. trimming read tails, and trimming any retained reverse primer binding regions and associated junk sequence) .

**Sequence read counts are after all filtering, immediately prior to even depth subsampling
