# Supplementary material for: Characterizing the Human Mycobiota: A Comparison of Small Subunit rRNA, ITS1, ITS2, and Large Subunit rRNA Genomic Targets
Source: Front Microbiol. 2018 Sep 19;9:2208. doi: 10.3389/fmicb.2018.02208 (PMC6157398; doi:10.3389/fmicb.2018.02208)
Supplement: Supplementary file 3 [file Data_Sheet_1.docx]

**Supplementary Data**. Sequences and pairwise alignments of ZOTUs for mock community isolates which returned more than one unique sequence for each marker region.

#============================================================================================

# 1. ITS1

#============================================================================================

1.1 AALT (2 zOTUs)

Full sequences:

(n = sequence reads/2000 sequences after subsampling to even depth)

>Otu20 (n = 61)

AAGTCGTAACAAGGTCTCCGTAGGTGAACCTGCGGAGGGATCATTACACAACTATGCAGGCGGGCTGGAACCTCTCGGTG

TTACAGCCTTGCTGAATTATTCACCCTTGTCTTTTGCGTACTTCTTGTTTCCTTGGTGGGTTCGCCCACCACTAGGACAA

ACATAAACCTTTTGTAATTGCAATCAGCGTCAGTAACAAATTAATAAT

>Otu3 (n = 1938)

AAGTCGTAACAAGGTCTCCGTAGGTGAACCTGCGGAGGGATCATTACACAAATATGAAGGCGGGCTGGAACCTCTCGGGG

TTACAGCCTTGCTGAATTATTCACCCTTGTCTTTTGCGTACTTCTTGTTTCCTTGGTGGGTTCGCCCACCACTAGGACAA

ACATAAACCTTTTGTAATTGCAATCAGCGTCAGTAACAAATTAATAAT

---------------------------

Pairwise global alignments:

Query >Otu20

%Id TLen Target

99% 208 Otu3

Query 208nt >Otu20

Target 208nt >Otu3

Qry 1 + AAGTCGTAACAAGGTCTCCGTAGGTGAACCTGCGGAGGGATCATTACACAACTATGCAGGCGGGCTGGAACCTCTCGGTG 80

||||||||||||||||||||||||||||||||||||||||||||||||||| |||| ||||||||||||||||||||| |

Tgt 1 + AAGTCGTAACAAGGTCTCCGTAGGTGAACCTGCGGAGGGATCATTACACAAATATGAAGGCGGGCTGGAACCTCTCGGGG 80

Qry 81 + TTACAGCCTTGCTGAATTATTCACCCTTGTCTTTTGCGTACTTCTTGTTTCCTTGGTGGGTTCGCCCACCACTAGGACAA 160

||||||||||||||||||||||||||||||||||||||||||||||||||||||||||||||||||||||||||||||||

Tgt 81 + TTACAGCCTTGCTGAATTATTCACCCTTGTCTTTTGCGTACTTCTTGTTTCCTTGGTGGGTTCGCCCACCACTAGGACAA 160

Qry 161 + ACATAAACCTTTTGTAATTGCAATCAGCGTCAGTAACAAATTAATAAT 208

||||||||||||||||||||||||||||||||||||||||||||||||

Tgt 161 + ACATAAACCTTTTGTAATTGCAATCAGCGTCAGTAACAAATTAATAAT 208

208 cols, 205 ids (98.6%), 0 gaps (0.0%)

#============================================================================================

1.2 YLIP (5 zOTUs)

Full sequences:

(n = sequence reads/2000 sequences after subsampling to even depth)

>Otu21 (n = 1927)

AAGTCGTAACAAGGTTTCCGTAGGTGAACCTGCGGAAGGATCATTATTGATTTTATCTATTTCTGTGGATTTCTATTCTA

TTACAGCGTCATTTTATCTCAATTATAACTATCAACAACGGATCTCTTGGCTCTC

>Otu11 (n = 8)

AAGTCGTAACAAGGTTTCCGTAGGTGAACCTGCGGAAGGATCATTACCGAGTGCGGGTCCTTTGGGCCCAACCTCCCATC

CGTGTCTATTGTACCCTGTTGCTTCGGCGGGCCCGCCGCTTGTCGGCCGCCGGGGGGGCGCCTCTGCCCCCCGGGCCCGT

GCCCGCCGGAGACCCCAACACGAACCCTGTCTGAAAGCGTGCAGTCTG

>Otu12 (n = 12)

AAGTCGTAACAAGGTTTCCGTAGGTGAACCTGCGGAAGGATCATTACCGAGTGAGGGCCCTCTGGGTCCAACCTCCCACC

CGTGTCTATCGTACCTTGTTGCTTCGGCGGGCCCGCCGTTTCGACGGCCGCCGGGGAGGCCTTGCGCCCCCGGGCCCGCG

CCCGCCGAAGACCCCAACATGAACGCTGTTCTGAAAGTATGCAGTCTG

>Otu18 (n = 8)

AAGTCGTAACAAGGTCTCCGTTGGTGAACCAGCGGAGGGATCATTACCGAGTTATACAACTCATCAACCCTGTGAACATA

CCTAAAACGTTGCTTCGGCGGGAACAGACGGCCCCGTAACACGGGCCGCCCCCGCCAGAGGACCCCCTAACTCTGTTTCT

ATTATGTTTCTTCTGAGTAAAACAAGCAAATAAATTAAAACTTTCAAC

>Otu1 (n = 25)

AAGTCGTAACAAGGTTTCCGTAGGTGAACCTGCGGAAGGATCATTAGTGAATTGCTCTTTGAGCGTTAAACTATATCCAT

CTACACCTGTGAACTGTTGATTGACTTCGGTCAATTACTTTTACAAACATTGTGTAATGAACGTCATGTTATTATAACAA

AAATAACTTTCAACAACGGATCTCTTGGCTCTCGCATCGATGAAGAAC

---------------------------

Pairwise global alignments:

Query >Otu21

%Id TLen Target

57% 208 Otu11

57% 208 Otu1

55% 208 Otu18

55% 208 Otu12

Query 135nt >Otu21

Target 208nt >Otu11

Qry 1 + AAGTCGTAACAAGGTTTCCGTAGGTGAACCTGCGGAAGGATCATTATTGATTTTATCTATTTCTGTGGATTTCTATTCTA 80

|||||||||||||||||||||||||||||||||||||||||||||| || ||| || ||

Tgt 1 + AAGTCGTAACAAGGTTTCCGTAGGTGAACCTGCGGAAGGATCATTACCGA--------------GTGCGGGTCCTTTGGG 66

Qry 81 + TTACAGCGTCATTTTATCTCAATTATAACTATCAACAACGGATCTCTTGGCTCTC 135

| | | | | || ||| || | | ||| | | | ||

Tgt 67 + CCCAACCTCCCATCCGTGTCTATTGTACCCTGTTGCTTCGGCGGGCCCGCCGCTT 121

135 cols, 77 ids (57.0%), 14 gaps (10.4%)

Query 135nt >Otu21

Target 208nt >Otu1

Qry 1 + AAGTCGTAACAAGGTTTCCGTAGGTGAACCTGCGGAAGGATCATTATTGATTTTATCT---------------------- 58

|||||||||||||||||||||||||||||||||||||||||||||| ||| || |||

Tgt 1 + AAGTCGTAACAAGGTTTCCGTAGGTGAACCTGCGGAAGGATCATTAGTGAATTGCTCTTTGAGCGTTAAACTATATCCAT 80

Qry 59 + -----------------------ATTTCTGTGGATTTCTATTCTA-------------TTACAGCGTCATTTTATCTCAA 102

| ||| || ||| || || | || | | | |||| |||

Tgt 81 + CTACACCTGTGAACTGTTGATTGACTTCGGTCAATTACTTTTACAAACATTGTGTAATGAACGTCATGTTATTATAACAA 160

Qry 103 + TTATAACTATCAACAACGGATCTCTTGGCTCTC 135

|||||| ||||||||||||||||||||||||

Tgt 161 + AAATAACTTTCAACAACGGATCTCTTGGCTCTC 193

193 cols, 110 ids (57.0%), 58 gaps (30.1%)

Query 135nt >Otu21

Target 208nt >Otu18

Qry 1 + AAGTCGTAACAAGGTTTCCGTAGGTGAACCTGCGGAAGGATCATTATTGATTTTATCTATTTCTGTGGATTTCTATTCTA 80

||||||||||||||| ||||| |||||||| ||||| ||||||||| || || | | | | | | ||

Tgt 1 + AAGTCGTAACAAGGTCTCCGTTGGTGAACCAGCGGAGGGATCATTACCGAGTTATACAACTCATCAACCCTGTGAACATA 80

Qry 81 + TTACAGCGTCATTTTATCTCAATTATAACTATCAACAACGGATCTCTTGGCTCTC 135

| | || || ||| | | || | | | | | | |

Tgt 81 + CCTAA----AACGTTGCTTCGGCGGGAACAGACGGCCCCGTAACACGGGCCGCCC 131

135 cols, 74 ids (54.8%), 4 gaps (3.0%)

Query 135nt >Otu21

Target 208nt >Otu12

Qry 1 + AAGTCGTAACAAGGTTTCCGTAGGTGAACCTGCGGAAGGATCATTATTGATTTTATCTATTTCTGTGGATTTCTATTCTA 80

|||||||||||||||||||||||||||||||||||||||||||||| || ||| | |

Tgt 1 + AAGTCGTAACAAGGTTTCCGTAGGTGAACCTGCGGAAGGATCATTACCGA--------------GTGAGGGCCCTCTGGG 66

Qry 81 + TTACAGCGTCATTTTATCTCAATTATAACTATCAACAACGGATCTCTTGGCTCTC 135

| | | | | || || || || | ||| | | | |

Tgt 67 + TCCAACCTCCCACCCGTGTCTATCGTACCTTGTTGCTTCGGCGGGCCCGCCGTTT 121

135 cols, 74 ids (54.8%), 14 gaps (10.4%)

Query >Otu11

%Id TLen Target

89% 208 Otu12

54% 208 Otu18

51% 208 Otu1

Query 208nt >Otu11

Target 208nt >Otu12

Qry 1 + AAGTCGTAACAAGGTTTCCGTAGGTGAACCTGCGGAAGGATCATTACCGAGTGCGGGTCCTTTGGGCCCAACCTCCCATC 80

||||||||||||||||||||||||||||||||||||||||||||||||||||| ||| ||| |||| ||||||||||| |

Tgt 1 + AAGTCGTAACAAGGTTTCCGTAGGTGAACCTGCGGAAGGATCATTACCGAGTGAGGGCCCTCTGGGTCCAACCTCCCACC 80

Qry 81 + CGTGTCTATTGTACCCTGTTGCTTCGGCGGGCCCGCCGCTT-GTCGGCCGCCGGGGGGGCGCCTCTGCCCCCCGGGCCCG 159

||||||||| ||||| |||||||||||||||||||||| || | |||||||||||| ||| || ||||||||||||

Tgt 81 + CGTGTCTATCGTACCTTGTTGCTTCGGCGGGCCCGCCGTTTCGACGGCCGCCGGGGAGGC--CTTGCGCCCCCGGGCCCG 158

Qry 160 + TGCCCGCCGGAGACCCCAACACGAACCCTGT-CTGAAAGCGTGCAGTCTG 208

|||||||| ||||||||||| |||| |||| ||||||| |||||||||

Tgt 159 + CGCCCGCCGAAGACCCCAACATGAACGCTGTTCTGAAAGTATGCAGTCTG 208

210 cols, 186 ids (88.6%), 4 gaps (1.9%)

Query 208nt >Otu11

Target 208nt >Otu18

Qry 1 + AAGTCGTAACAAGGTTTCCGTAGGTGAACCTGCGGAAGGATCATTACCGAGTGCGGGTCCTTTGGGCCCAACCTCCCATC 80

||||||||||||||| ||||| |||||||| ||||| ||||||||||||||| |||| || |

Tgt 1 + AAGTCGTAACAAGGTCTCCGTTGGTGAACCAGCGGAGGGATCATTACCGAGT------------TATACAACTCATCAAC 68

Qry 81 + CGTGT---CTATTGTACCCTGTTGCTTCGGCGGGCCCGCCGCTTGTCGGCCGCCGGGGGGGCGCCTCTGCCCCCCGGGCC 157

| ||| | || |||||||||||||| | | ||||| | | ||| | || || | |

Tgt 69 + CCTGTGAACATACCTAAAACGTTGCTTCGGCGGGAAC------AGACGGCCCCGTAACACGGGCCGCCCCCGCCAGAGGA 142

Qry 158 + CGTGCCCGCCGGAGACCCCAACACGAACCCTGTCTG-AAAGCGTGCAGTCTG 208

| || | | | | | | | | | | ||| | |||

Tgt 143 + C-CCCCTAACTCTGTTTCTATTATGTTTCTTCTGAGTAAAACAAGCAAATAA 193

212 cols, 115 ids (54.2%), 23 gaps (10.8%)

Query 208nt >Otu11

Target 208nt >Otu1

Qry 1 + AAGTCGTAACAAGGTTTCCGTAGGTGAACCTGCGGAAGGATCATTACCGAGTGCGGGTCCTTTGGGCCCAACCTCCCATC 80

|||||||||||||||||||||||||||||||||||||||||||||| || | | ||||| || | |||

Tgt 1 + AAGTCGTAACAAGGTTTCCGTAGGTGAACCTGCGGAAGGATCATTAGTGAAT---TGCTCTTTGAGCGTTAAACTATATC 77

Qry 81 + CGTGT-CTATTGTACCCTGTTG-----CTTCGG-------CGGGCCCGCCGCTTGTCGGCCGCCGGGGGGGCGCCTCTGC 147

| | | | ||| |||||| |||||| | | |||| | | | | |

Tgt 78 + CATCTACACCTGTGAACTGTTGATTGACTTCGGTCAATTACTTTTACAAACATTGTGTAATGAACGTCATGTTATTATAA 157

Qry 148 + CCCCCGGGCCCGTGCCCGCCGGAGACCCCAACACGAACCCTGTCTGAAAGC 198

| | | | |||| | | | | | | | |

Tgt 158 + CAAAAATAACTTTCAACAACGGATCTCTTGGCTCTCGCATCGATGAAGAAC 208

211 cols, 107 ids (50.7%), 16 gaps (7.6%)

Query >Otu12

%Id TLen Target

55% 208 Otu18

49% 208 Otu1

Query 208nt >Otu12

Target 208nt >Otu18

Qry 1 + AAGTCGTAACAAGGTTTCCGTAGGTGAACCTGCGGAAGGATCATTACCGAGTGAGGGCCCTCTGGGTCCAACCTCCCACC 80

||||||||||||||| ||||| |||||||| ||||| ||||||||||||||| | | |||| || |

Tgt 1 + AAGTCGTAACAAGGTCTCCGTTGGTGAACCAGCGGAGGGATCATTACCGAGTTA------------TACAACTCATCAAC 68

Qry 81 + CGTGT---CTATCGTACCTTGTTGCTTCGGCGGGCCCGCCGTTTCGACGGCCGCCGGGGA-GGCCTTGCGCCCCCGGGCC 156

| ||| | | || |||||||||||||| | ||||||| | | || | | || || |

Tgt 69 + CCTGTGAACATACCTAAAACGTTGCTTCGGCGGGAAC-------AGACGGCCCCGTAACACGGGCCGCCCCCGCCAGAGG 141

Qry 157 + CGCGCCCGCCGAAGACCCCAACATGAACGCTGTTCTGAAAGTATGCAGTCTG 208

| || | | | | ||| | | ||| | |||

Tgt 142 + ACCCCCTAACTCTGTTTCTATTATGTTTCTTCTGAGTAAAACAAGCAAATAA 193

212 cols, 117 ids (55.2%), 23 gaps (10.8%)

Query 208nt >Otu12

Target 208nt >Otu1

Qry 1 + AAGTCGTAACAAGGTTTCCGTAGGTGAACCTGCGGAAGGATCATTACCGAGTGAGGGCCCTCTGGG--------TCCAAC 72

|||||||||||||||||||||||||||||||||||||||||||||| || | || || || | | | |

Tgt 1 + AAGTCGTAACAAGGTTTCCGTAGGTGAACCTGCGGAAGGATCATTAGTGA---ATTGCTCTTTGAGCGTTAAACTATATC 77

Qry 73 + CTCCCACCCGTGTCTATCGTACCTTGTTGCTTCGGCGGGCCCGCCGTTTCGACGGCCGCCGGGGAGGCCTTGCGCCCCCG 152

| | || | ||| | || ||| |||||| | || | | | | | | | |

Tgt 78 + CATCTACACCTGTGAACTGTTGATTG--ACTTCGG-------TCAATTACTTTTACAAACATTGTGTAATGAACGTCATG 148

Qry 153 + GGCCCGCGCCCGCCGAAGACCCCAACATGAACGCTGTT-----CTGAAAGTATGCAGTCT 207

| | ||||| || || | | ||| ||

Tgt 149 + TTATTATAACAAAAATAACTTTCAACAACGGATCTCTTGGCTCTCGCATCGATGAAGAAC 208

220 cols, 108 ids (49.1%), 25 gaps (11.4%)

Query >Otu18

%Id TLen Target

51% 208 Otu1

Query 208nt >Otu18

Target 208nt >Otu1

Qry 1 + AAGTCGTAACAAGGTCTCCGTTGGTGAACCAGCGGAGGGATCATTACCGA---GTTATACAACTCATCAACCCTGTGAAC 77

||||||||||||||| ||||| |||||||| ||||| ||||||||| || | | | | | ||| | | |

Tgt 1 + AAGTCGTAACAAGGTTTCCGTAGGTGAACCTGCGGAAGGATCATTAGTGAATTGCTCTTTGAGCGTTAAACTATATCCAT 80

Qry 78 + ATAC----CTAAAACGTTGCTT--CGGCGGGAACAGACGGCCCCGTAACACGGGCCGCCCCCGCCA---GAGGACCCCCT 148

||| | || |||| || | ||| | || | | | | || || | | |

Tgt 81 + CTACACCTGTGAACTGTTGATTGACTTCGGTCAATTACTTTTACAAACATTGTGTAATGAACGTCATGTTATTATAACAA 160

Qry 149 + AACTCTGTTTCTATTATGTTTCTTCTGAGTAAAACAAGCAAATAAATT 196

|| | |||| | | | ||| || | || | | |

Tgt 161 + AAATAACTTTCAACAACGGATCTCTTGGCTCTCGCATCGATGAAGAAC 208

208 cols, 106 ids (51.0%), 12 gaps (5.8%)

#============================================================================================

# 2. ITS2

#============================================================================================

2.1 FSOL

Full sequences:

(n = sequence reads/2000 sequences after subsampling to even depth)

>Otu21 (n = 525)

GAAATGCGATAAGTAATGTGAATTGCAGAATTCAGTGAATCATCGAATCTTTGAACGCACATTGCGCCCGCCAGTATTCT

GGCGGGCATGCCTGTTCGAGCGTCATTACAACCCTCAGGCCCCCGGGCCTGGCGTTGGGGATCGGCGAGGCGCCCCCTGC

GGGCACACGCCGTCCCCCAAATACAGTGGCGGTCCCGCCGCCGCCTCCCT

>Otu22 (n = 365)

GAAATGCGATAAGTAATGTGAATTGCAGAATTCAGTGAATCATCGAATCTTTGAACGCACATTGCGCCCGCCAGTATTCT

GGCGGGCATGCCTGTTCGAGCGTCATTACAACCCTCAGGCCCCCGGGCCTGGCGTTGGGGATCGGCGAGGCGCCCCCTGC

GGGCACACGCCGTCCCCCAAATACAGTGGCGGTCCCGCCGCAGCTTCCCT

>Otu20 (n = 309)

GAAATGCGATAAGTAATGTGAATTGCAGAATTCAGTGAATCATCGAATCTTTGAACGCACATTGCGCCCGCCAGTATTCT

GGCGGGCATGCCTGTTCGAGCGTCATTACAACCCTCAGGCCCCCGGGCCTGGCGTTGGGGATCGGCGAGGCGCCCCCTGC

GGGCACACGCCGTCCCCCAAATACAGTGGCGGTCCCGCCGCCGCTTCCCT

>Otu23 (n = 290)

GAAATGCGATAAGTAATGTGAATTGCAGAATTCAGTGAATCATCGAATCTTTGAACGCACATTGCGCCCGCCAGTATTCT

GGCGGGCATGCCTGTTCGAGCGTCATTACAACCCTCAGGCCCCCGGGCCTGGCGTTGGGGATCGGCGAGGCGCCCCCTGC

GGGCACACGCCGTCCCCCAAATACAGTGGCGGTCCCCCCGCCGCTTCCCT

>Otu26 (n = 119)

GAAATGCGATAAGTAATGTGAATTGCAGAATTCAGTGAATCATCGAATCTTTGAACGCACATTGCGCCCGCCAGTATTCT

GGCGGGCATGCCTGTTCGAGCGTCATTACAACCCTCAGGCCCCCGGGCCTGGCGTTGGGGATCGGCGAGGCGCCCCCTGC

GGGCACACGCCGTCCCCCAAATACAGTGGCGGGCCCGCCGCCGCTTCCCT

>Otu25 (n = 98)

GAAATGCGATAAGTAATGTGAATTGCAGAATTCAGTGAATCATCGAATCTTTGAACGCACATTGCGCCCGCCAGTATTCT

GGCGGGCATGCCTGTTCGAGCGTCATTACAACCCTCAGGCCCCCGGGCCTGGCGTTGGGGATCGGCGAGGCGCCCCCTGC

GGGCACACGCCGTCCCCCAAATACAGTGGCGGTCCCGCCGCAGCCTCCCT

>Otu31 (n = 88)

GAAATGCGATAAGTAATGTGAATTGCAGAATTCAGTGAATCATCGAATCTTTGAACGCACATTGCGCCCGCCAGTATTCT

GGCGGGCATGCCTGTTCGAGCGTCATTACAACCCTCAGGCCCCCGGGCCTGGCGTTGGGGATCGGCGAGGCGCCCCCTGC

GGGCACACGCCGTCCCCCAAATACAGTGGCGGGCCCGCCGCCGCCTCCCT

>Otu24 (n = 86)

GAAATGCGATAAGTAATGTGAATTGCAGAATTCAGTGAATCATCGAATCTTTGAACGCACATTGCGCCCGCCAGTATTCT

GGCGGGCATGCCTGTTCGAGCGTCATTACAACCCTCAGGCCCCCGGGCCTGGCGTTGGGGATCGGCGAGGCGCCCCCTGC

GGGCACACGCCGTCCCCCAAATACAGTGGCGGTCCCCCCGCCGCCTCCCT

>Otu33 (n = 73)

GAAATGCGATAAGTAATGTGAATTGCAGAATTCAGTGAATCATCGAATCTTTGAACGCACATTGCGCCCGCCAGTATTCT

GGCGGGCATGCCTGTTCGAGCGTCATTACAACCCTCAGGCCCCCGGGCCTGGCGTTGGGGATCGGCGAGGCGCCCCCTGC

GGGCACACGCCGTCCCCCAAATACAGTGGCGGGCCCGCCGCAGCTTCCCT

>Otu40 (n = 45)

GAAATGCGATAAGTAATGTGAATTGCAGAATTCAGTGAATCATCGAATCTTTGAACGCACATTGCGCCCGCCAGTATTCT

GGCGGGCATGCCTGTTCGAGCGTCATTACAACCCTCAGGCCCCCGGGCCTGGCGTTGGGGATCGGCGAGGCGCCCCCTGC

GGGCACACGCCGTCCCCCAAATACAGTGGCGGGCCCGCCGCAGCCTCCCT

---------------------------

Pairwise global alignments:

Query >Otu21

%Id TLen Target

100% 210 Otu25

100% 210 Otu31

100% 210 Otu24

100% 210 Otu20

99% 210 Otu22

99% 210 Otu40

99% 210 Otu23

99% 210 Otu26

99% 210 Otu33

Query 210nt >Otu21

Target 210nt >Otu25

Qry 1 + GAAATGCGATAAGTAATGTGAATTGCAGAATTCAGTGAATCATCGAATCTTTGAACGCACATTGCGCCCGCCAGTATTCT 80

||||||||||||||||||||||||||||||||||||||||||||||||||||||||||||||||||||||||||||||||

Tgt 1 + GAAATGCGATAAGTAATGTGAATTGCAGAATTCAGTGAATCATCGAATCTTTGAACGCACATTGCGCCCGCCAGTATTCT 80

Qry 81 + GGCGGGCATGCCTGTTCGAGCGTCATTACAACCCTCAGGCCCCCGGGCCTGGCGTTGGGGATCGGCGAGGCGCCCCCTGC 160

||||||||||||||||||||||||||||||||||||||||||||||||||||||||||||||||||||||||||||||||

Tgt 81 + GGCGGGCATGCCTGTTCGAGCGTCATTACAACCCTCAGGCCCCCGGGCCTGGCGTTGGGGATCGGCGAGGCGCCCCCTGC 160

Qry 161 + GGGCACACGCCGTCCCCCAAATACAGTGGCGGTCCCGCCGCCGCCTCCCT 210

||||||||||||||||||||||||||||||||||||||||| ||||||||

Tgt 161 + GGGCACACGCCGTCCCCCAAATACAGTGGCGGTCCCGCCGCAGCCTCCCT 210

210 cols, 209 ids (99.5%), 0 gaps (0.0%)

Query 210nt >Otu21

Target 210nt >Otu31

Qry 1 + GAAATGCGATAAGTAATGTGAATTGCAGAATTCAGTGAATCATCGAATCTTTGAACGCACATTGCGCCCGCCAGTATTCT 80

||||||||||||||||||||||||||||||||||||||||||||||||||||||||||||||||||||||||||||||||

Tgt 1 + GAAATGCGATAAGTAATGTGAATTGCAGAATTCAGTGAATCATCGAATCTTTGAACGCACATTGCGCCCGCCAGTATTCT 80

Qry 81 + GGCGGGCATGCCTGTTCGAGCGTCATTACAACCCTCAGGCCCCCGGGCCTGGCGTTGGGGATCGGCGAGGCGCCCCCTGC 160

||||||||||||||||||||||||||||||||||||||||||||||||||||||||||||||||||||||||||||||||

Tgt 81 + GGCGGGCATGCCTGTTCGAGCGTCATTACAACCCTCAGGCCCCCGGGCCTGGCGTTGGGGATCGGCGAGGCGCCCCCTGC 160

Qry 161 + GGGCACACGCCGTCCCCCAAATACAGTGGCGGTCCCGCCGCCGCCTCCCT 210

|||||||||||||||||||||||||||||||| |||||||||||||||||

Tgt 161 + GGGCACACGCCGTCCCCCAAATACAGTGGCGGGCCCGCCGCCGCCTCCCT 210

210 cols, 209 ids (99.5%), 0 gaps (0.0%)

Query 210nt >Otu21

Target 210nt >Otu24

Qry 1 + GAAATGCGATAAGTAATGTGAATTGCAGAATTCAGTGAATCATCGAATCTTTGAACGCACATTGCGCCCGCCAGTATTCT 80

||||||||||||||||||||||||||||||||||||||||||||||||||||||||||||||||||||||||||||||||

Tgt 1 + GAAATGCGATAAGTAATGTGAATTGCAGAATTCAGTGAATCATCGAATCTTTGAACGCACATTGCGCCCGCCAGTATTCT 80

Qry 81 + GGCGGGCATGCCTGTTCGAGCGTCATTACAACCCTCAGGCCCCCGGGCCTGGCGTTGGGGATCGGCGAGGCGCCCCCTGC 160

||||||||||||||||||||||||||||||||||||||||||||||||||||||||||||||||||||||||||||||||

Tgt 81 + GGCGGGCATGCCTGTTCGAGCGTCATTACAACCCTCAGGCCCCCGGGCCTGGCGTTGGGGATCGGCGAGGCGCCCCCTGC 160

Qry 161 + GGGCACACGCCGTCCCCCAAATACAGTGGCGGTCCCGCCGCCGCCTCCCT 210

|||||||||||||||||||||||||||||||||||| |||||||||||||

Tgt 161 + GGGCACACGCCGTCCCCCAAATACAGTGGCGGTCCCCCCGCCGCCTCCCT 210

210 cols, 209 ids (99.5%), 0 gaps (0.0%)

Query 210nt >Otu21

Target 210nt >Otu20

Qry 1 + GAAATGCGATAAGTAATGTGAATTGCAGAATTCAGTGAATCATCGAATCTTTGAACGCACATTGCGCCCGCCAGTATTCT 80

||||||||||||||||||||||||||||||||||||||||||||||||||||||||||||||||||||||||||||||||

Tgt 1 + GAAATGCGATAAGTAATGTGAATTGCAGAATTCAGTGAATCATCGAATCTTTGAACGCACATTGCGCCCGCCAGTATTCT 80

Qry 81 + GGCGGGCATGCCTGTTCGAGCGTCATTACAACCCTCAGGCCCCCGGGCCTGGCGTTGGGGATCGGCGAGGCGCCCCCTGC 160

||||||||||||||||||||||||||||||||||||||||||||||||||||||||||||||||||||||||||||||||

Tgt 81 + GGCGGGCATGCCTGTTCGAGCGTCATTACAACCCTCAGGCCCCCGGGCCTGGCGTTGGGGATCGGCGAGGCGCCCCCTGC 160

Qry 161 + GGGCACACGCCGTCCCCCAAATACAGTGGCGGTCCCGCCGCCGCCTCCCT 210

|||||||||||||||||||||||||||||||||||||||||||| |||||

Tgt 161 + GGGCACACGCCGTCCCCCAAATACAGTGGCGGTCCCGCCGCCGCTTCCCT 210

210 cols, 209 ids (99.5%), 0 gaps (0.0%)

Query 210nt >Otu21

Target 210nt >Otu22

Qry 1 + GAAATGCGATAAGTAATGTGAATTGCAGAATTCAGTGAATCATCGAATCTTTGAACGCACATTGCGCCCGCCAGTATTCT 80

||||||||||||||||||||||||||||||||||||||||||||||||||||||||||||||||||||||||||||||||

Tgt 1 + GAAATGCGATAAGTAATGTGAATTGCAGAATTCAGTGAATCATCGAATCTTTGAACGCACATTGCGCCCGCCAGTATTCT 80

Qry 81 + GGCGGGCATGCCTGTTCGAGCGTCATTACAACCCTCAGGCCCCCGGGCCTGGCGTTGGGGATCGGCGAGGCGCCCCCTGC 160

||||||||||||||||||||||||||||||||||||||||||||||||||||||||||||||||||||||||||||||||

Tgt 81 + GGCGGGCATGCCTGTTCGAGCGTCATTACAACCCTCAGGCCCCCGGGCCTGGCGTTGGGGATCGGCGAGGCGCCCCCTGC 160

Qry 161 + GGGCACACGCCGTCCCCCAAATACAGTGGCGGTCCCGCCGCCGCCTCCCT 210

||||||||||||||||||||||||||||||||||||||||| || |||||

Tgt 161 + GGGCACACGCCGTCCCCCAAATACAGTGGCGGTCCCGCCGCAGCTTCCCT 210

210 cols, 208 ids (99.0%), 0 gaps (0.0%)

Query 210nt >Otu21

Target 210nt >Otu40

Qry 1 + GAAATGCGATAAGTAATGTGAATTGCAGAATTCAGTGAATCATCGAATCTTTGAACGCACATTGCGCCCGCCAGTATTCT 80

||||||||||||||||||||||||||||||||||||||||||||||||||||||||||||||||||||||||||||||||

Tgt 1 + GAAATGCGATAAGTAATGTGAATTGCAGAATTCAGTGAATCATCGAATCTTTGAACGCACATTGCGCCCGCCAGTATTCT 80

Qry 81 + GGCGGGCATGCCTGTTCGAGCGTCATTACAACCCTCAGGCCCCCGGGCCTGGCGTTGGGGATCGGCGAGGCGCCCCCTGC 160

||||||||||||||||||||||||||||||||||||||||||||||||||||||||||||||||||||||||||||||||

Tgt 81 + GGCGGGCATGCCTGTTCGAGCGTCATTACAACCCTCAGGCCCCCGGGCCTGGCGTTGGGGATCGGCGAGGCGCCCCCTGC 160

Qry 161 + GGGCACACGCCGTCCCCCAAATACAGTGGCGGTCCCGCCGCCGCCTCCCT 210

|||||||||||||||||||||||||||||||| |||||||| ||||||||

Tgt 161 + GGGCACACGCCGTCCCCCAAATACAGTGGCGGGCCCGCCGCAGCCTCCCT 210

210 cols, 208 ids (99.0%), 0 gaps (0.0%)

Query 210nt >Otu21

Target 210nt >Otu23

Qry 1 + GAAATGCGATAAGTAATGTGAATTGCAGAATTCAGTGAATCATCGAATCTTTGAACGCACATTGCGCCCGCCAGTATTCT 80

||||||||||||||||||||||||||||||||||||||||||||||||||||||||||||||||||||||||||||||||

Tgt 1 + GAAATGCGATAAGTAATGTGAATTGCAGAATTCAGTGAATCATCGAATCTTTGAACGCACATTGCGCCCGCCAGTATTCT 80

Qry 81 + GGCGGGCATGCCTGTTCGAGCGTCATTACAACCCTCAGGCCCCCGGGCCTGGCGTTGGGGATCGGCGAGGCGCCCCCTGC 160

||||||||||||||||||||||||||||||||||||||||||||||||||||||||||||||||||||||||||||||||

Tgt 81 + GGCGGGCATGCCTGTTCGAGCGTCATTACAACCCTCAGGCCCCCGGGCCTGGCGTTGGGGATCGGCGAGGCGCCCCCTGC 160

Qry 161 + GGGCACACGCCGTCCCCCAAATACAGTGGCGGTCCCGCCGCCGCCTCCCT 210

|||||||||||||||||||||||||||||||||||| ||||||| |||||

Tgt 161 + GGGCACACGCCGTCCCCCAAATACAGTGGCGGTCCCCCCGCCGCTTCCCT 210

210 cols, 208 ids (99.0%), 0 gaps (0.0%)

Query 210nt >Otu21

Target 210nt >Otu26

Qry 1 + GAAATGCGATAAGTAATGTGAATTGCAGAATTCAGTGAATCATCGAATCTTTGAACGCACATTGCGCCCGCCAGTATTCT 80

||||||||||||||||||||||||||||||||||||||||||||||||||||||||||||||||||||||||||||||||

Tgt 1 + GAAATGCGATAAGTAATGTGAATTGCAGAATTCAGTGAATCATCGAATCTTTGAACGCACATTGCGCCCGCCAGTATTCT 80

Qry 81 + GGCGGGCATGCCTGTTCGAGCGTCATTACAACCCTCAGGCCCCCGGGCCTGGCGTTGGGGATCGGCGAGGCGCCCCCTGC 160

||||||||||||||||||||||||||||||||||||||||||||||||||||||||||||||||||||||||||||||||

Tgt 81 + GGCGGGCATGCCTGTTCGAGCGTCATTACAACCCTCAGGCCCCCGGGCCTGGCGTTGGGGATCGGCGAGGCGCCCCCTGC 160

Qry 161 + GGGCACACGCCGTCCCCCAAATACAGTGGCGGTCCCGCCGCCGCCTCCCT 210

|||||||||||||||||||||||||||||||| ||||||||||| |||||

Tgt 161 + GGGCACACGCCGTCCCCCAAATACAGTGGCGGGCCCGCCGCCGCTTCCCT 210

210 cols, 208 ids (99.0%), 0 gaps (0.0%)

Query 210nt >Otu21

Target 210nt >Otu33

Qry 1 + GAAATGCGATAAGTAATGTGAATTGCAGAATTCAGTGAATCATCGAATCTTTGAACGCACATTGCGCCCGCCAGTATTCT 80

||||||||||||||||||||||||||||||||||||||||||||||||||||||||||||||||||||||||||||||||

Tgt 1 + GAAATGCGATAAGTAATGTGAATTGCAGAATTCAGTGAATCATCGAATCTTTGAACGCACATTGCGCCCGCCAGTATTCT 80

Qry 81 + GGCGGGCATGCCTGTTCGAGCGTCATTACAACCCTCAGGCCCCCGGGCCTGGCGTTGGGGATCGGCGAGGCGCCCCCTGC 160

||||||||||||||||||||||||||||||||||||||||||||||||||||||||||||||||||||||||||||||||

Tgt 81 + GGCGGGCATGCCTGTTCGAGCGTCATTACAACCCTCAGGCCCCCGGGCCTGGCGTTGGGGATCGGCGAGGCGCCCCCTGC 160

Qry 161 + GGGCACACGCCGTCCCCCAAATACAGTGGCGGTCCCGCCGCCGCCTCCCT 210

|||||||||||||||||||||||||||||||| |||||||| || |||||

Tgt 161 + GGGCACACGCCGTCCCCCAAATACAGTGGCGGGCCCGCCGCAGCTTCCCT 210

210 cols, 207 ids (98.6%), 0 gaps (0.0%)

Query >Otu22

%Id TLen Target

100% 210 Otu25

100% 210 Otu33

100% 210 Otu20

99% 210 Otu40

99% 210 Otu26

99% 210 Otu23

99% 210 Otu31

99% 210 Otu24

Query 210nt >Otu22

Target 210nt >Otu25

Qry 1 + GAAATGCGATAAGTAATGTGAATTGCAGAATTCAGTGAATCATCGAATCTTTGAACGCACATTGCGCCCGCCAGTATTCT 80

||||||||||||||||||||||||||||||||||||||||||||||||||||||||||||||||||||||||||||||||

Tgt 1 + GAAATGCGATAAGTAATGTGAATTGCAGAATTCAGTGAATCATCGAATCTTTGAACGCACATTGCGCCCGCCAGTATTCT 80

Qry 81 + GGCGGGCATGCCTGTTCGAGCGTCATTACAACCCTCAGGCCCCCGGGCCTGGCGTTGGGGATCGGCGAGGCGCCCCCTGC 160

||||||||||||||||||||||||||||||||||||||||||||||||||||||||||||||||||||||||||||||||

Tgt 81 + GGCGGGCATGCCTGTTCGAGCGTCATTACAACCCTCAGGCCCCCGGGCCTGGCGTTGGGGATCGGCGAGGCGCCCCCTGC 160

Qry 161 + GGGCACACGCCGTCCCCCAAATACAGTGGCGGTCCCGCCGCAGCTTCCCT 210

|||||||||||||||||||||||||||||||||||||||||||| |||||

Tgt 161 + GGGCACACGCCGTCCCCCAAATACAGTGGCGGTCCCGCCGCAGCCTCCCT 210

210 cols, 209 ids (99.5%), 0 gaps (0.0%)

Query 210nt >Otu22

Target 210nt >Otu33

Qry 1 + GAAATGCGATAAGTAATGTGAATTGCAGAATTCAGTGAATCATCGAATCTTTGAACGCACATTGCGCCCGCCAGTATTCT 80

||||||||||||||||||||||||||||||||||||||||||||||||||||||||||||||||||||||||||||||||

Tgt 1 + GAAATGCGATAAGTAATGTGAATTGCAGAATTCAGTGAATCATCGAATCTTTGAACGCACATTGCGCCCGCCAGTATTCT 80

Qry 81 + GGCGGGCATGCCTGTTCGAGCGTCATTACAACCCTCAGGCCCCCGGGCCTGGCGTTGGGGATCGGCGAGGCGCCCCCTGC 160

||||||||||||||||||||||||||||||||||||||||||||||||||||||||||||||||||||||||||||||||

Tgt 81 + GGCGGGCATGCCTGTTCGAGCGTCATTACAACCCTCAGGCCCCCGGGCCTGGCGTTGGGGATCGGCGAGGCGCCCCCTGC 160

Qry 161 + GGGCACACGCCGTCCCCCAAATACAGTGGCGGTCCCGCCGCAGCTTCCCT 210

|||||||||||||||||||||||||||||||| |||||||||||||||||

Tgt 161 + GGGCACACGCCGTCCCCCAAATACAGTGGCGGGCCCGCCGCAGCTTCCCT 210

210 cols, 209 ids (99.5%), 0 gaps (0.0%)

Query 210nt >Otu22

Target 210nt >Otu20

Qry 1 + GAAATGCGATAAGTAATGTGAATTGCAGAATTCAGTGAATCATCGAATCTTTGAACGCACATTGCGCCCGCCAGTATTCT 80

||||||||||||||||||||||||||||||||||||||||||||||||||||||||||||||||||||||||||||||||

Tgt 1 + GAAATGCGATAAGTAATGTGAATTGCAGAATTCAGTGAATCATCGAATCTTTGAACGCACATTGCGCCCGCCAGTATTCT 80

Qry 81 + GGCGGGCATGCCTGTTCGAGCGTCATTACAACCCTCAGGCCCCCGGGCCTGGCGTTGGGGATCGGCGAGGCGCCCCCTGC 160

||||||||||||||||||||||||||||||||||||||||||||||||||||||||||||||||||||||||||||||||

Tgt 81 + GGCGGGCATGCCTGTTCGAGCGTCATTACAACCCTCAGGCCCCCGGGCCTGGCGTTGGGGATCGGCGAGGCGCCCCCTGC 160

Qry 161 + GGGCACACGCCGTCCCCCAAATACAGTGGCGGTCCCGCCGCAGCTTCCCT 210

||||||||||||||||||||||||||||||||||||||||| ||||||||

Tgt 161 + GGGCACACGCCGTCCCCCAAATACAGTGGCGGTCCCGCCGCCGCTTCCCT 210

210 cols, 209 ids (99.5%), 0 gaps (0.0%)

Query 210nt >Otu22

Target 210nt >Otu40

Qry 1 + GAAATGCGATAAGTAATGTGAATTGCAGAATTCAGTGAATCATCGAATCTTTGAACGCACATTGCGCCCGCCAGTATTCT 80

||||||||||||||||||||||||||||||||||||||||||||||||||||||||||||||||||||||||||||||||

Tgt 1 + GAAATGCGATAAGTAATGTGAATTGCAGAATTCAGTGAATCATCGAATCTTTGAACGCACATTGCGCCCGCCAGTATTCT 80

Qry 81 + GGCGGGCATGCCTGTTCGAGCGTCATTACAACCCTCAGGCCCCCGGGCCTGGCGTTGGGGATCGGCGAGGCGCCCCCTGC 160

||||||||||||||||||||||||||||||||||||||||||||||||||||||||||||||||||||||||||||||||

Tgt 81 + GGCGGGCATGCCTGTTCGAGCGTCATTACAACCCTCAGGCCCCCGGGCCTGGCGTTGGGGATCGGCGAGGCGCCCCCTGC 160

Qry 161 + GGGCACACGCCGTCCCCCAAATACAGTGGCGGTCCCGCCGCAGCTTCCCT 210

|||||||||||||||||||||||||||||||| ||||||||||| |||||

Tgt 161 + GGGCACACGCCGTCCCCCAAATACAGTGGCGGGCCCGCCGCAGCCTCCCT 210

210 cols, 208 ids (99.0%), 0 gaps (0.0%)

Query 210nt >Otu22

Target 210nt >Otu26

Qry 1 + GAAATGCGATAAGTAATGTGAATTGCAGAATTCAGTGAATCATCGAATCTTTGAACGCACATTGCGCCCGCCAGTATTCT 80

||||||||||||||||||||||||||||||||||||||||||||||||||||||||||||||||||||||||||||||||

Tgt 1 + GAAATGCGATAAGTAATGTGAATTGCAGAATTCAGTGAATCATCGAATCTTTGAACGCACATTGCGCCCGCCAGTATTCT 80

Qry 81 + GGCGGGCATGCCTGTTCGAGCGTCATTACAACCCTCAGGCCCCCGGGCCTGGCGTTGGGGATCGGCGAGGCGCCCCCTGC 160

||||||||||||||||||||||||||||||||||||||||||||||||||||||||||||||||||||||||||||||||

Tgt 81 + GGCGGGCATGCCTGTTCGAGCGTCATTACAACCCTCAGGCCCCCGGGCCTGGCGTTGGGGATCGGCGAGGCGCCCCCTGC 160

Qry 161 + GGGCACACGCCGTCCCCCAAATACAGTGGCGGTCCCGCCGCAGCTTCCCT 210

|||||||||||||||||||||||||||||||| |||||||| ||||||||

Tgt 161 + GGGCACACGCCGTCCCCCAAATACAGTGGCGGGCCCGCCGCCGCTTCCCT 210

210 cols, 208 ids (99.0%), 0 gaps (0.0%)

Query 210nt >Otu22

Target 210nt >Otu23

Qry 1 + GAAATGCGATAAGTAATGTGAATTGCAGAATTCAGTGAATCATCGAATCTTTGAACGCACATTGCGCCCGCCAGTATTCT 80

||||||||||||||||||||||||||||||||||||||||||||||||||||||||||||||||||||||||||||||||

Tgt 1 + GAAATGCGATAAGTAATGTGAATTGCAGAATTCAGTGAATCATCGAATCTTTGAACGCACATTGCGCCCGCCAGTATTCT 80

Qry 81 + GGCGGGCATGCCTGTTCGAGCGTCATTACAACCCTCAGGCCCCCGGGCCTGGCGTTGGGGATCGGCGAGGCGCCCCCTGC 160

||||||||||||||||||||||||||||||||||||||||||||||||||||||||||||||||||||||||||||||||

Tgt 81 + GGCGGGCATGCCTGTTCGAGCGTCATTACAACCCTCAGGCCCCCGGGCCTGGCGTTGGGGATCGGCGAGGCGCCCCCTGC 160

Qry 161 + GGGCACACGCCGTCCCCCAAATACAGTGGCGGTCCCGCCGCAGCTTCCCT 210

|||||||||||||||||||||||||||||||||||| |||| ||||||||

Tgt 161 + GGGCACACGCCGTCCCCCAAATACAGTGGCGGTCCCCCCGCCGCTTCCCT 210

210 cols, 208 ids (99.0%), 0 gaps (0.0%)

Query 210nt >Otu22

Target 210nt >Otu31

Qry 1 + GAAATGCGATAAGTAATGTGAATTGCAGAATTCAGTGAATCATCGAATCTTTGAACGCACATTGCGCCCGCCAGTATTCT 80

||||||||||||||||||||||||||||||||||||||||||||||||||||||||||||||||||||||||||||||||

Tgt 1 + GAAATGCGATAAGTAATGTGAATTGCAGAATTCAGTGAATCATCGAATCTTTGAACGCACATTGCGCCCGCCAGTATTCT 80

Qry 81 + GGCGGGCATGCCTGTTCGAGCGTCATTACAACCCTCAGGCCCCCGGGCCTGGCGTTGGGGATCGGCGAGGCGCCCCCTGC 160

||||||||||||||||||||||||||||||||||||||||||||||||||||||||||||||||||||||||||||||||

Tgt 81 + GGCGGGCATGCCTGTTCGAGCGTCATTACAACCCTCAGGCCCCCGGGCCTGGCGTTGGGGATCGGCGAGGCGCCCCCTGC 160

Qry 161 + GGGCACACGCCGTCCCCCAAATACAGTGGCGGTCCCGCCGCAGCTTCCCT 210

|||||||||||||||||||||||||||||||| |||||||| || |||||

Tgt 161 + GGGCACACGCCGTCCCCCAAATACAGTGGCGGGCCCGCCGCCGCCTCCCT 210

210 cols, 207 ids (98.6%), 0 gaps (0.0%)

Query 210nt >Otu22

Target 210nt >Otu24

Qry 1 + GAAATGCGATAAGTAATGTGAATTGCAGAATTCAGTGAATCATCGAATCTTTGAACGCACATTGCGCCCGCCAGTATTCT 80

||||||||||||||||||||||||||||||||||||||||||||||||||||||||||||||||||||||||||||||||

Tgt 1 + GAAATGCGATAAGTAATGTGAATTGCAGAATTCAGTGAATCATCGAATCTTTGAACGCACATTGCGCCCGCCAGTATTCT 80

Qry 81 + GGCGGGCATGCCTGTTCGAGCGTCATTACAACCCTCAGGCCCCCGGGCCTGGCGTTGGGGATCGGCGAGGCGCCCCCTGC 160

||||||||||||||||||||||||||||||||||||||||||||||||||||||||||||||||||||||||||||||||

Tgt 81 + GGCGGGCATGCCTGTTCGAGCGTCATTACAACCCTCAGGCCCCCGGGCCTGGCGTTGGGGATCGGCGAGGCGCCCCCTGC 160

Qry 161 + GGGCACACGCCGTCCCCCAAATACAGTGGCGGTCCCGCCGCAGCTTCCCT 210

|||||||||||||||||||||||||||||||||||| |||| || |||||

Tgt 161 + GGGCACACGCCGTCCCCCAAATACAGTGGCGGTCCCCCCGCCGCCTCCCT 210

210 cols, 207 ids (98.6%), 0 gaps (0.0%)

Query >Otu20

%Id TLen Target

100% 210 Otu26

100% 210 Otu23

99% 210 Otu24

99% 210 Otu33

99% 210 Otu25

99% 210 Otu31

99% 210 Otu40

Query 210nt >Otu20

Target 210nt >Otu26

Qry 1 + GAAATGCGATAAGTAATGTGAATTGCAGAATTCAGTGAATCATCGAATCTTTGAACGCACATTGCGCCCGCCAGTATTCT 80

||||||||||||||||||||||||||||||||||||||||||||||||||||||||||||||||||||||||||||||||

Tgt 1 + GAAATGCGATAAGTAATGTGAATTGCAGAATTCAGTGAATCATCGAATCTTTGAACGCACATTGCGCCCGCCAGTATTCT 80

Qry 81 + GGCGGGCATGCCTGTTCGAGCGTCATTACAACCCTCAGGCCCCCGGGCCTGGCGTTGGGGATCGGCGAGGCGCCCCCTGC 160

||||||||||||||||||||||||||||||||||||||||||||||||||||||||||||||||||||||||||||||||

Tgt 81 + GGCGGGCATGCCTGTTCGAGCGTCATTACAACCCTCAGGCCCCCGGGCCTGGCGTTGGGGATCGGCGAGGCGCCCCCTGC 160

Qry 161 + GGGCACACGCCGTCCCCCAAATACAGTGGCGGTCCCGCCGCCGCTTCCCT 210

|||||||||||||||||||||||||||||||| |||||||||||||||||

Tgt 161 + GGGCACACGCCGTCCCCCAAATACAGTGGCGGGCCCGCCGCCGCTTCCCT 210

210 cols, 209 ids (99.5%), 0 gaps (0.0%)

Query 210nt >Otu20

Target 210nt >Otu23

Qry 1 + GAAATGCGATAAGTAATGTGAATTGCAGAATTCAGTGAATCATCGAATCTTTGAACGCACATTGCGCCCGCCAGTATTCT 80

||||||||||||||||||||||||||||||||||||||||||||||||||||||||||||||||||||||||||||||||

Tgt 1 + GAAATGCGATAAGTAATGTGAATTGCAGAATTCAGTGAATCATCGAATCTTTGAACGCACATTGCGCCCGCCAGTATTCT 80

Qry 81 + GGCGGGCATGCCTGTTCGAGCGTCATTACAACCCTCAGGCCCCCGGGCCTGGCGTTGGGGATCGGCGAGGCGCCCCCTGC 160

||||||||||||||||||||||||||||||||||||||||||||||||||||||||||||||||||||||||||||||||

Tgt 81 + GGCGGGCATGCCTGTTCGAGCGTCATTACAACCCTCAGGCCCCCGGGCCTGGCGTTGGGGATCGGCGAGGCGCCCCCTGC 160

Qry 161 + GGGCACACGCCGTCCCCCAAATACAGTGGCGGTCCCGCCGCCGCTTCCCT 210

|||||||||||||||||||||||||||||||||||| |||||||||||||

Tgt 161 + GGGCACACGCCGTCCCCCAAATACAGTGGCGGTCCCCCCGCCGCTTCCCT 210

210 cols, 209 ids (99.5%), 0 gaps (0.0%)

Query 210nt >Otu20

Target 210nt >Otu24

Qry 1 + GAAATGCGATAAGTAATGTGAATTGCAGAATTCAGTGAATCATCGAATCTTTGAACGCACATTGCGCCCGCCAGTATTCT 80

||||||||||||||||||||||||||||||||||||||||||||||||||||||||||||||||||||||||||||||||

Tgt 1 + GAAATGCGATAAGTAATGTGAATTGCAGAATTCAGTGAATCATCGAATCTTTGAACGCACATTGCGCCCGCCAGTATTCT 80

Qry 81 + GGCGGGCATGCCTGTTCGAGCGTCATTACAACCCTCAGGCCCCCGGGCCTGGCGTTGGGGATCGGCGAGGCGCCCCCTGC 160

||||||||||||||||||||||||||||||||||||||||||||||||||||||||||||||||||||||||||||||||

Tgt 81 + GGCGGGCATGCCTGTTCGAGCGTCATTACAACCCTCAGGCCCCCGGGCCTGGCGTTGGGGATCGGCGAGGCGCCCCCTGC 160

Qry 161 + GGGCACACGCCGTCCCCCAAATACAGTGGCGGTCCCGCCGCCGCTTCCCT 210

|||||||||||||||||||||||||||||||||||| ||||||| |||||

Tgt 161 + GGGCACACGCCGTCCCCCAAATACAGTGGCGGTCCCCCCGCCGCCTCCCT 210

210 cols, 208 ids (99.0%), 0 gaps (0.0%)

Query 210nt >Otu20

Target 210nt >Otu33

Qry 1 + GAAATGCGATAAGTAATGTGAATTGCAGAATTCAGTGAATCATCGAATCTTTGAACGCACATTGCGCCCGCCAGTATTCT 80

||||||||||||||||||||||||||||||||||||||||||||||||||||||||||||||||||||||||||||||||

Tgt 1 + GAAATGCGATAAGTAATGTGAATTGCAGAATTCAGTGAATCATCGAATCTTTGAACGCACATTGCGCCCGCCAGTATTCT 80

Qry 81 + GGCGGGCATGCCTGTTCGAGCGTCATTACAACCCTCAGGCCCCCGGGCCTGGCGTTGGGGATCGGCGAGGCGCCCCCTGC 160

||||||||||||||||||||||||||||||||||||||||||||||||||||||||||||||||||||||||||||||||

Tgt 81 + GGCGGGCATGCCTGTTCGAGCGTCATTACAACCCTCAGGCCCCCGGGCCTGGCGTTGGGGATCGGCGAGGCGCCCCCTGC 160

Qry 161 + GGGCACACGCCGTCCCCCAAATACAGTGGCGGTCCCGCCGCCGCTTCCCT 210

|||||||||||||||||||||||||||||||| |||||||| ||||||||

Tgt 161 + GGGCACACGCCGTCCCCCAAATACAGTGGCGGGCCCGCCGCAGCTTCCCT 210

210 cols, 208 ids (99.0%), 0 gaps (0.0%)

Query 210nt >Otu20

Target 210nt >Otu25

Qry 1 + GAAATGCGATAAGTAATGTGAATTGCAGAATTCAGTGAATCATCGAATCTTTGAACGCACATTGCGCCCGCCAGTATTCT 80

||||||||||||||||||||||||||||||||||||||||||||||||||||||||||||||||||||||||||||||||

Tgt 1 + GAAATGCGATAAGTAATGTGAATTGCAGAATTCAGTGAATCATCGAATCTTTGAACGCACATTGCGCCCGCCAGTATTCT 80

Qry 81 + GGCGGGCATGCCTGTTCGAGCGTCATTACAACCCTCAGGCCCCCGGGCCTGGCGTTGGGGATCGGCGAGGCGCCCCCTGC 160

||||||||||||||||||||||||||||||||||||||||||||||||||||||||||||||||||||||||||||||||

Tgt 81 + GGCGGGCATGCCTGTTCGAGCGTCATTACAACCCTCAGGCCCCCGGGCCTGGCGTTGGGGATCGGCGAGGCGCCCCCTGC 160

Qry 161 + GGGCACACGCCGTCCCCCAAATACAGTGGCGGTCCCGCCGCCGCTTCCCT 210

||||||||||||||||||||||||||||||||||||||||| || |||||

Tgt 161 + GGGCACACGCCGTCCCCCAAATACAGTGGCGGTCCCGCCGCAGCCTCCCT 210

210 cols, 208 ids (99.0%), 0 gaps (0.0%)

Query 210nt >Otu20

Target 210nt >Otu31

Qry 1 + GAAATGCGATAAGTAATGTGAATTGCAGAATTCAGTGAATCATCGAATCTTTGAACGCACATTGCGCCCGCCAGTATTCT 80

||||||||||||||||||||||||||||||||||||||||||||||||||||||||||||||||||||||||||||||||

Tgt 1 + GAAATGCGATAAGTAATGTGAATTGCAGAATTCAGTGAATCATCGAATCTTTGAACGCACATTGCGCCCGCCAGTATTCT 80

Qry 81 + GGCGGGCATGCCTGTTCGAGCGTCATTACAACCCTCAGGCCCCCGGGCCTGGCGTTGGGGATCGGCGAGGCGCCCCCTGC 160

||||||||||||||||||||||||||||||||||||||||||||||||||||||||||||||||||||||||||||||||

Tgt 81 + GGCGGGCATGCCTGTTCGAGCGTCATTACAACCCTCAGGCCCCCGGGCCTGGCGTTGGGGATCGGCGAGGCGCCCCCTGC 160

Qry 161 + GGGCACACGCCGTCCCCCAAATACAGTGGCGGTCCCGCCGCCGCTTCCCT 210

|||||||||||||||||||||||||||||||| ||||||||||| |||||

Tgt 161 + GGGCACACGCCGTCCCCCAAATACAGTGGCGGGCCCGCCGCCGCCTCCCT 210

210 cols, 208 ids (99.0%), 0 gaps (0.0%)

Query 210nt >Otu20

Target 210nt >Otu40

Qry 1 + GAAATGCGATAAGTAATGTGAATTGCAGAATTCAGTGAATCATCGAATCTTTGAACGCACATTGCGCCCGCCAGTATTCT 80

||||||||||||||||||||||||||||||||||||||||||||||||||||||||||||||||||||||||||||||||

Tgt 1 + GAAATGCGATAAGTAATGTGAATTGCAGAATTCAGTGAATCATCGAATCTTTGAACGCACATTGCGCCCGCCAGTATTCT 80

Qry 81 + GGCGGGCATGCCTGTTCGAGCGTCATTACAACCCTCAGGCCCCCGGGCCTGGCGTTGGGGATCGGCGAGGCGCCCCCTGC 160

||||||||||||||||||||||||||||||||||||||||||||||||||||||||||||||||||||||||||||||||

Tgt 81 + GGCGGGCATGCCTGTTCGAGCGTCATTACAACCCTCAGGCCCCCGGGCCTGGCGTTGGGGATCGGCGAGGCGCCCCCTGC 160

Qry 161 + GGGCACACGCCGTCCCCCAAATACAGTGGCGGTCCCGCCGCCGCTTCCCT 210

|||||||||||||||||||||||||||||||| |||||||| || |||||

Tgt 161 + GGGCACACGCCGTCCCCCAAATACAGTGGCGGGCCCGCCGCAGCCTCCCT 210

210 cols, 207 ids (98.6%), 0 gaps (0.0%)

Query >Otu23

%Id TLen Target

100% 210 Otu24

99% 210 Otu26

99% 210 Otu33

99% 210 Otu25

99% 210 Otu31

98% 210 Otu40

Query 210nt >Otu23

Target 210nt >Otu24

Qry 1 + GAAATGCGATAAGTAATGTGAATTGCAGAATTCAGTGAATCATCGAATCTTTGAACGCACATTGCGCCCGCCAGTATTCT 80

||||||||||||||||||||||||||||||||||||||||||||||||||||||||||||||||||||||||||||||||

Tgt 1 + GAAATGCGATAAGTAATGTGAATTGCAGAATTCAGTGAATCATCGAATCTTTGAACGCACATTGCGCCCGCCAGTATTCT 80

Qry 81 + GGCGGGCATGCCTGTTCGAGCGTCATTACAACCCTCAGGCCCCCGGGCCTGGCGTTGGGGATCGGCGAGGCGCCCCCTGC 160

||||||||||||||||||||||||||||||||||||||||||||||||||||||||||||||||||||||||||||||||

Tgt 81 + GGCGGGCATGCCTGTTCGAGCGTCATTACAACCCTCAGGCCCCCGGGCCTGGCGTTGGGGATCGGCGAGGCGCCCCCTGC 160

Qry 161 + GGGCACACGCCGTCCCCCAAATACAGTGGCGGTCCCCCCGCCGCTTCCCT 210

|||||||||||||||||||||||||||||||||||||||||||| |||||

Tgt 161 + GGGCACACGCCGTCCCCCAAATACAGTGGCGGTCCCCCCGCCGCCTCCCT 210

210 cols, 209 ids (99.5%), 0 gaps (0.0%)

Query 210nt >Otu23

Target 210nt >Otu26

Qry 1 + GAAATGCGATAAGTAATGTGAATTGCAGAATTCAGTGAATCATCGAATCTTTGAACGCACATTGCGCCCGCCAGTATTCT 80

||||||||||||||||||||||||||||||||||||||||||||||||||||||||||||||||||||||||||||||||

Tgt 1 + GAAATGCGATAAGTAATGTGAATTGCAGAATTCAGTGAATCATCGAATCTTTGAACGCACATTGCGCCCGCCAGTATTCT 80

Qry 81 + GGCGGGCATGCCTGTTCGAGCGTCATTACAACCCTCAGGCCCCCGGGCCTGGCGTTGGGGATCGGCGAGGCGCCCCCTGC 160

||||||||||||||||||||||||||||||||||||||||||||||||||||||||||||||||||||||||||||||||

Tgt 81 + GGCGGGCATGCCTGTTCGAGCGTCATTACAACCCTCAGGCCCCCGGGCCTGGCGTTGGGGATCGGCGAGGCGCCCCCTGC 160

Qry 161 + GGGCACACGCCGTCCCCCAAATACAGTGGCGGTCCCCCCGCCGCTTCCCT 210

|||||||||||||||||||||||||||||||| ||| |||||||||||||

Tgt 161 + GGGCACACGCCGTCCCCCAAATACAGTGGCGGGCCCGCCGCCGCTTCCCT 210

210 cols, 208 ids (99.0%), 0 gaps (0.0%)

Query 210nt >Otu23

Target 210nt >Otu33

Qry 1 + GAAATGCGATAAGTAATGTGAATTGCAGAATTCAGTGAATCATCGAATCTTTGAACGCACATTGCGCCCGCCAGTATTCT 80

||||||||||||||||||||||||||||||||||||||||||||||||||||||||||||||||||||||||||||||||

Tgt 1 + GAAATGCGATAAGTAATGTGAATTGCAGAATTCAGTGAATCATCGAATCTTTGAACGCACATTGCGCCCGCCAGTATTCT 80

Qry 81 + GGCGGGCATGCCTGTTCGAGCGTCATTACAACCCTCAGGCCCCCGGGCCTGGCGTTGGGGATCGGCGAGGCGCCCCCTGC 160

||||||||||||||||||||||||||||||||||||||||||||||||||||||||||||||||||||||||||||||||

Tgt 81 + GGCGGGCATGCCTGTTCGAGCGTCATTACAACCCTCAGGCCCCCGGGCCTGGCGTTGGGGATCGGCGAGGCGCCCCCTGC 160

Qry 161 + GGGCACACGCCGTCCCCCAAATACAGTGGCGGTCCCCCCGCCGCTTCCCT 210

|||||||||||||||||||||||||||||||| ||| |||| ||||||||

Tgt 161 + GGGCACACGCCGTCCCCCAAATACAGTGGCGGGCCCGCCGCAGCTTCCCT 210

210 cols, 207 ids (98.6%), 0 gaps (0.0%)

Query 210nt >Otu23

Target 210nt >Otu25

Qry 1 + GAAATGCGATAAGTAATGTGAATTGCAGAATTCAGTGAATCATCGAATCTTTGAACGCACATTGCGCCCGCCAGTATTCT 80

||||||||||||||||||||||||||||||||||||||||||||||||||||||||||||||||||||||||||||||||

Tgt 1 + GAAATGCGATAAGTAATGTGAATTGCAGAATTCAGTGAATCATCGAATCTTTGAACGCACATTGCGCCCGCCAGTATTCT 80

Qry 81 + GGCGGGCATGCCTGTTCGAGCGTCATTACAACCCTCAGGCCCCCGGGCCTGGCGTTGGGGATCGGCGAGGCGCCCCCTGC 160

||||||||||||||||||||||||||||||||||||||||||||||||||||||||||||||||||||||||||||||||

Tgt 81 + GGCGGGCATGCCTGTTCGAGCGTCATTACAACCCTCAGGCCCCCGGGCCTGGCGTTGGGGATCGGCGAGGCGCCCCCTGC 160

Qry 161 + GGGCACACGCCGTCCCCCAAATACAGTGGCGGTCCCCCCGCCGCTTCCCT 210

|||||||||||||||||||||||||||||||||||| |||| || |||||

Tgt 161 + GGGCACACGCCGTCCCCCAAATACAGTGGCGGTCCCGCCGCAGCCTCCCT 210

210 cols, 207 ids (98.6%), 0 gaps (0.0%)

Query 210nt >Otu23

Target 210nt >Otu31

Qry 1 + GAAATGCGATAAGTAATGTGAATTGCAGAATTCAGTGAATCATCGAATCTTTGAACGCACATTGCGCCCGCCAGTATTCT 80

||||||||||||||||||||||||||||||||||||||||||||||||||||||||||||||||||||||||||||||||

Tgt 1 + GAAATGCGATAAGTAATGTGAATTGCAGAATTCAGTGAATCATCGAATCTTTGAACGCACATTGCGCCCGCCAGTATTCT 80

Qry 81 + GGCGGGCATGCCTGTTCGAGCGTCATTACAACCCTCAGGCCCCCGGGCCTGGCGTTGGGGATCGGCGAGGCGCCCCCTGC 160

||||||||||||||||||||||||||||||||||||||||||||||||||||||||||||||||||||||||||||||||

Tgt 81 + GGCGGGCATGCCTGTTCGAGCGTCATTACAACCCTCAGGCCCCCGGGCCTGGCGTTGGGGATCGGCGAGGCGCCCCCTGC 160

Qry 161 + GGGCACACGCCGTCCCCCAAATACAGTGGCGGTCCCCCCGCCGCTTCCCT 210

|||||||||||||||||||||||||||||||| ||| ||||||| |||||

Tgt 161 + GGGCACACGCCGTCCCCCAAATACAGTGGCGGGCCCGCCGCCGCCTCCCT 210

210 cols, 207 ids (98.6%), 0 gaps (0.0%)

Query 210nt >Otu23

Target 210nt >Otu40

Qry 1 + GAAATGCGATAAGTAATGTGAATTGCAGAATTCAGTGAATCATCGAATCTTTGAACGCACATTGCGCCCGCCAGTATTCT 80

||||||||||||||||||||||||||||||||||||||||||||||||||||||||||||||||||||||||||||||||

Tgt 1 + GAAATGCGATAAGTAATGTGAATTGCAGAATTCAGTGAATCATCGAATCTTTGAACGCACATTGCGCCCGCCAGTATTCT 80

Qry 81 + GGCGGGCATGCCTGTTCGAGCGTCATTACAACCCTCAGGCCCCCGGGCCTGGCGTTGGGGATCGGCGAGGCGCCCCCTGC 160

||||||||||||||||||||||||||||||||||||||||||||||||||||||||||||||||||||||||||||||||

Tgt 81 + GGCGGGCATGCCTGTTCGAGCGTCATTACAACCCTCAGGCCCCCGGGCCTGGCGTTGGGGATCGGCGAGGCGCCCCCTGC 160

Qry 161 + GGGCACACGCCGTCCCCCAAATACAGTGGCGGTCCCCCCGCCGCTTCCCT 210

|||||||||||||||||||||||||||||||| ||| |||| || |||||

Tgt 161 + GGGCACACGCCGTCCCCCAAATACAGTGGCGGGCCCGCCGCAGCCTCCCT 210

210 cols, 206 ids (98.1%), 0 gaps (0.0%)

Query >Otu26

%Id TLen Target

100% 210 Otu33

100% 210 Otu31

99% 210 Otu40

99% 210 Otu25

99% 210 Otu24

Query 210nt >Otu26

Target 210nt >Otu33

Qry 1 + GAAATGCGATAAGTAATGTGAATTGCAGAATTCAGTGAATCATCGAATCTTTGAACGCACATTGCGCCCGCCAGTATTCT 80

||||||||||||||||||||||||||||||||||||||||||||||||||||||||||||||||||||||||||||||||

Tgt 1 + GAAATGCGATAAGTAATGTGAATTGCAGAATTCAGTGAATCATCGAATCTTTGAACGCACATTGCGCCCGCCAGTATTCT 80

Qry 81 + GGCGGGCATGCCTGTTCGAGCGTCATTACAACCCTCAGGCCCCCGGGCCTGGCGTTGGGGATCGGCGAGGCGCCCCCTGC 160

||||||||||||||||||||||||||||||||||||||||||||||||||||||||||||||||||||||||||||||||

Tgt 81 + GGCGGGCATGCCTGTTCGAGCGTCATTACAACCCTCAGGCCCCCGGGCCTGGCGTTGGGGATCGGCGAGGCGCCCCCTGC 160

Qry 161 + GGGCACACGCCGTCCCCCAAATACAGTGGCGGGCCCGCCGCCGCTTCCCT 210

||||||||||||||||||||||||||||||||||||||||| ||||||||

Tgt 161 + GGGCACACGCCGTCCCCCAAATACAGTGGCGGGCCCGCCGCAGCTTCCCT 210

210 cols, 209 ids (99.5%), 0 gaps (0.0%)

Query 210nt >Otu26

Target 210nt >Otu31

Qry 1 + GAAATGCGATAAGTAATGTGAATTGCAGAATTCAGTGAATCATCGAATCTTTGAACGCACATTGCGCCCGCCAGTATTCT 80

||||||||||||||||||||||||||||||||||||||||||||||||||||||||||||||||||||||||||||||||

Tgt 1 + GAAATGCGATAAGTAATGTGAATTGCAGAATTCAGTGAATCATCGAATCTTTGAACGCACATTGCGCCCGCCAGTATTCT 80

Qry 81 + GGCGGGCATGCCTGTTCGAGCGTCATTACAACCCTCAGGCCCCCGGGCCTGGCGTTGGGGATCGGCGAGGCGCCCCCTGC 160

||||||||||||||||||||||||||||||||||||||||||||||||||||||||||||||||||||||||||||||||

Tgt 81 + GGCGGGCATGCCTGTTCGAGCGTCATTACAACCCTCAGGCCCCCGGGCCTGGCGTTGGGGATCGGCGAGGCGCCCCCTGC 160

Qry 161 + GGGCACACGCCGTCCCCCAAATACAGTGGCGGGCCCGCCGCCGCTTCCCT 210

|||||||||||||||||||||||||||||||||||||||||||| |||||

Tgt 161 + GGGCACACGCCGTCCCCCAAATACAGTGGCGGGCCCGCCGCCGCCTCCCT 210

210 cols, 209 ids (99.5%), 0 gaps (0.0%)

Query 210nt >Otu26

Target 210nt >Otu40

Qry 1 + GAAATGCGATAAGTAATGTGAATTGCAGAATTCAGTGAATCATCGAATCTTTGAACGCACATTGCGCCCGCCAGTATTCT 80

||||||||||||||||||||||||||||||||||||||||||||||||||||||||||||||||||||||||||||||||

Tgt 1 + GAAATGCGATAAGTAATGTGAATTGCAGAATTCAGTGAATCATCGAATCTTTGAACGCACATTGCGCCCGCCAGTATTCT 80

Qry 81 + GGCGGGCATGCCTGTTCGAGCGTCATTACAACCCTCAGGCCCCCGGGCCTGGCGTTGGGGATCGGCGAGGCGCCCCCTGC 160

||||||||||||||||||||||||||||||||||||||||||||||||||||||||||||||||||||||||||||||||

Tgt 81 + GGCGGGCATGCCTGTTCGAGCGTCATTACAACCCTCAGGCCCCCGGGCCTGGCGTTGGGGATCGGCGAGGCGCCCCCTGC 160

Qry 161 + GGGCACACGCCGTCCCCCAAATACAGTGGCGGGCCCGCCGCCGCTTCCCT 210

||||||||||||||||||||||||||||||||||||||||| || |||||

Tgt 161 + GGGCACACGCCGTCCCCCAAATACAGTGGCGGGCCCGCCGCAGCCTCCCT 210

210 cols, 208 ids (99.0%), 0 gaps (0.0%)

Query 210nt >Otu26

Target 210nt >Otu25

Qry 1 + GAAATGCGATAAGTAATGTGAATTGCAGAATTCAGTGAATCATCGAATCTTTGAACGCACATTGCGCCCGCCAGTATTCT 80

||||||||||||||||||||||||||||||||||||||||||||||||||||||||||||||||||||||||||||||||

Tgt 1 + GAAATGCGATAAGTAATGTGAATTGCAGAATTCAGTGAATCATCGAATCTTTGAACGCACATTGCGCCCGCCAGTATTCT 80

Qry 81 + GGCGGGCATGCCTGTTCGAGCGTCATTACAACCCTCAGGCCCCCGGGCCTGGCGTTGGGGATCGGCGAGGCGCCCCCTGC 160

||||||||||||||||||||||||||||||||||||||||||||||||||||||||||||||||||||||||||||||||

Tgt 81 + GGCGGGCATGCCTGTTCGAGCGTCATTACAACCCTCAGGCCCCCGGGCCTGGCGTTGGGGATCGGCGAGGCGCCCCCTGC 160

Qry 161 + GGGCACACGCCGTCCCCCAAATACAGTGGCGGGCCCGCCGCCGCTTCCCT 210

|||||||||||||||||||||||||||||||| |||||||| || |||||

Tgt 161 + GGGCACACGCCGTCCCCCAAATACAGTGGCGGTCCCGCCGCAGCCTCCCT 210

210 cols, 207 ids (98.6%), 0 gaps (0.0%)

Query 210nt >Otu26

Target 210nt >Otu24

Qry 1 + GAAATGCGATAAGTAATGTGAATTGCAGAATTCAGTGAATCATCGAATCTTTGAACGCACATTGCGCCCGCCAGTATTCT 80

||||||||||||||||||||||||||||||||||||||||||||||||||||||||||||||||||||||||||||||||

Tgt 1 + GAAATGCGATAAGTAATGTGAATTGCAGAATTCAGTGAATCATCGAATCTTTGAACGCACATTGCGCCCGCCAGTATTCT 80

Qry 81 + GGCGGGCATGCCTGTTCGAGCGTCATTACAACCCTCAGGCCCCCGGGCCTGGCGTTGGGGATCGGCGAGGCGCCCCCTGC 160

||||||||||||||||||||||||||||||||||||||||||||||||||||||||||||||||||||||||||||||||

Tgt 81 + GGCGGGCATGCCTGTTCGAGCGTCATTACAACCCTCAGGCCCCCGGGCCTGGCGTTGGGGATCGGCGAGGCGCCCCCTGC 160

Qry 161 + GGGCACACGCCGTCCCCCAAATACAGTGGCGGGCCCGCCGCCGCTTCCCT 210

|||||||||||||||||||||||||||||||| ||| ||||||| |||||

Tgt 161 + GGGCACACGCCGTCCCCCAAATACAGTGGCGGTCCCCCCGCCGCCTCCCT 210

210 cols, 207 ids (98.6%), 0 gaps (0.0%)

Query >Otu25

%Id TLen Target

100% 210 Otu40

99% 210 Otu33

99% 210 Otu31

99% 210 Otu24

Query 210nt >Otu25

Target 210nt >Otu40

Qry 1 + GAAATGCGATAAGTAATGTGAATTGCAGAATTCAGTGAATCATCGAATCTTTGAACGCACATTGCGCCCGCCAGTATTCT 80

||||||||||||||||||||||||||||||||||||||||||||||||||||||||||||||||||||||||||||||||

Tgt 1 + GAAATGCGATAAGTAATGTGAATTGCAGAATTCAGTGAATCATCGAATCTTTGAACGCACATTGCGCCCGCCAGTATTCT 80

Qry 81 + GGCGGGCATGCCTGTTCGAGCGTCATTACAACCCTCAGGCCCCCGGGCCTGGCGTTGGGGATCGGCGAGGCGCCCCCTGC 160

||||||||||||||||||||||||||||||||||||||||||||||||||||||||||||||||||||||||||||||||

Tgt 81 + GGCGGGCATGCCTGTTCGAGCGTCATTACAACCCTCAGGCCCCCGGGCCTGGCGTTGGGGATCGGCGAGGCGCCCCCTGC 160

Qry 161 + GGGCACACGCCGTCCCCCAAATACAGTGGCGGTCCCGCCGCAGCCTCCCT 210

|||||||||||||||||||||||||||||||| |||||||||||||||||

Tgt 161 + GGGCACACGCCGTCCCCCAAATACAGTGGCGGGCCCGCCGCAGCCTCCCT 210

210 cols, 209 ids (99.5%), 0 gaps (0.0%)

Query 210nt >Otu25

Target 210nt >Otu33

Qry 1 + GAAATGCGATAAGTAATGTGAATTGCAGAATTCAGTGAATCATCGAATCTTTGAACGCACATTGCGCCCGCCAGTATTCT 80

||||||||||||||||||||||||||||||||||||||||||||||||||||||||||||||||||||||||||||||||

Tgt 1 + GAAATGCGATAAGTAATGTGAATTGCAGAATTCAGTGAATCATCGAATCTTTGAACGCACATTGCGCCCGCCAGTATTCT 80

Qry 81 + GGCGGGCATGCCTGTTCGAGCGTCATTACAACCCTCAGGCCCCCGGGCCTGGCGTTGGGGATCGGCGAGGCGCCCCCTGC 160

||||||||||||||||||||||||||||||||||||||||||||||||||||||||||||||||||||||||||||||||

Tgt 81 + GGCGGGCATGCCTGTTCGAGCGTCATTACAACCCTCAGGCCCCCGGGCCTGGCGTTGGGGATCGGCGAGGCGCCCCCTGC 160

Qry 161 + GGGCACACGCCGTCCCCCAAATACAGTGGCGGTCCCGCCGCAGCCTCCCT 210

|||||||||||||||||||||||||||||||| ||||||||||| |||||

Tgt 161 + GGGCACACGCCGTCCCCCAAATACAGTGGCGGGCCCGCCGCAGCTTCCCT 210

210 cols, 208 ids (99.0%), 0 gaps (0.0%)

Query 210nt >Otu25

Target 210nt >Otu31

Qry 1 + GAAATGCGATAAGTAATGTGAATTGCAGAATTCAGTGAATCATCGAATCTTTGAACGCACATTGCGCCCGCCAGTATTCT 80

||||||||||||||||||||||||||||||||||||||||||||||||||||||||||||||||||||||||||||||||

Tgt 1 + GAAATGCGATAAGTAATGTGAATTGCAGAATTCAGTGAATCATCGAATCTTTGAACGCACATTGCGCCCGCCAGTATTCT 80

Qry 81 + GGCGGGCATGCCTGTTCGAGCGTCATTACAACCCTCAGGCCCCCGGGCCTGGCGTTGGGGATCGGCGAGGCGCCCCCTGC 160

||||||||||||||||||||||||||||||||||||||||||||||||||||||||||||||||||||||||||||||||

Tgt 81 + GGCGGGCATGCCTGTTCGAGCGTCATTACAACCCTCAGGCCCCCGGGCCTGGCGTTGGGGATCGGCGAGGCGCCCCCTGC 160

Qry 161 + GGGCACACGCCGTCCCCCAAATACAGTGGCGGTCCCGCCGCAGCCTCCCT 210

|||||||||||||||||||||||||||||||| |||||||| ||||||||

Tgt 161 + GGGCACACGCCGTCCCCCAAATACAGTGGCGGGCCCGCCGCCGCCTCCCT 210

210 cols, 208 ids (99.0%), 0 gaps (0.0%)

Query 210nt >Otu25

Target 210nt >Otu24

Qry 1 + GAAATGCGATAAGTAATGTGAATTGCAGAATTCAGTGAATCATCGAATCTTTGAACGCACATTGCGCCCGCCAGTATTCT 80

||||||||||||||||||||||||||||||||||||||||||||||||||||||||||||||||||||||||||||||||

Tgt 1 + GAAATGCGATAAGTAATGTGAATTGCAGAATTCAGTGAATCATCGAATCTTTGAACGCACATTGCGCCCGCCAGTATTCT 80

Qry 81 + GGCGGGCATGCCTGTTCGAGCGTCATTACAACCCTCAGGCCCCCGGGCCTGGCGTTGGGGATCGGCGAGGCGCCCCCTGC 160

||||||||||||||||||||||||||||||||||||||||||||||||||||||||||||||||||||||||||||||||

Tgt 81 + GGCGGGCATGCCTGTTCGAGCGTCATTACAACCCTCAGGCCCCCGGGCCTGGCGTTGGGGATCGGCGAGGCGCCCCCTGC 160

Qry 161 + GGGCACACGCCGTCCCCCAAATACAGTGGCGGTCCCGCCGCAGCCTCCCT 210

|||||||||||||||||||||||||||||||||||| |||| ||||||||

Tgt 161 + GGGCACACGCCGTCCCCCAAATACAGTGGCGGTCCCCCCGCCGCCTCCCT 210

210 cols, 208 ids (99.0%), 0 gaps (0.0%)

Query >Otu31

%Id TLen Target

100% 210 Otu40

99% 210 Otu33

99% 210 Otu24

Query 210nt >Otu31

Target 210nt >Otu40

Qry 1 + GAAATGCGATAAGTAATGTGAATTGCAGAATTCAGTGAATCATCGAATCTTTGAACGCACATTGCGCCCGCCAGTATTCT 80

||||||||||||||||||||||||||||||||||||||||||||||||||||||||||||||||||||||||||||||||

Tgt 1 + GAAATGCGATAAGTAATGTGAATTGCAGAATTCAGTGAATCATCGAATCTTTGAACGCACATTGCGCCCGCCAGTATTCT 80

Qry 81 + GGCGGGCATGCCTGTTCGAGCGTCATTACAACCCTCAGGCCCCCGGGCCTGGCGTTGGGGATCGGCGAGGCGCCCCCTGC 160

||||||||||||||||||||||||||||||||||||||||||||||||||||||||||||||||||||||||||||||||

Tgt 81 + GGCGGGCATGCCTGTTCGAGCGTCATTACAACCCTCAGGCCCCCGGGCCTGGCGTTGGGGATCGGCGAGGCGCCCCCTGC 160

Qry 161 + GGGCACACGCCGTCCCCCAAATACAGTGGCGGGCCCGCCGCCGCCTCCCT 210

||||||||||||||||||||||||||||||||||||||||| ||||||||

Tgt 161 + GGGCACACGCCGTCCCCCAAATACAGTGGCGGGCCCGCCGCAGCCTCCCT 210

210 cols, 209 ids (99.5%), 0 gaps (0.0%)

Query 210nt >Otu31

Target 210nt >Otu33

Qry 1 + GAAATGCGATAAGTAATGTGAATTGCAGAATTCAGTGAATCATCGAATCTTTGAACGCACATTGCGCCCGCCAGTATTCT 80

||||||||||||||||||||||||||||||||||||||||||||||||||||||||||||||||||||||||||||||||

Tgt 1 + GAAATGCGATAAGTAATGTGAATTGCAGAATTCAGTGAATCATCGAATCTTTGAACGCACATTGCGCCCGCCAGTATTCT 80

Qry 81 + GGCGGGCATGCCTGTTCGAGCGTCATTACAACCCTCAGGCCCCCGGGCCTGGCGTTGGGGATCGGCGAGGCGCCCCCTGC 160

||||||||||||||||||||||||||||||||||||||||||||||||||||||||||||||||||||||||||||||||

Tgt 81 + GGCGGGCATGCCTGTTCGAGCGTCATTACAACCCTCAGGCCCCCGGGCCTGGCGTTGGGGATCGGCGAGGCGCCCCCTGC 160

Qry 161 + GGGCACACGCCGTCCCCCAAATACAGTGGCGGGCCCGCCGCCGCCTCCCT 210

||||||||||||||||||||||||||||||||||||||||| || |||||

Tgt 161 + GGGCACACGCCGTCCCCCAAATACAGTGGCGGGCCCGCCGCAGCTTCCCT 210

210 cols, 208 ids (99.0%), 0 gaps (0.0%)

Query 210nt >Otu31

Target 210nt >Otu24

Qry 1 + GAAATGCGATAAGTAATGTGAATTGCAGAATTCAGTGAATCATCGAATCTTTGAACGCACATTGCGCCCGCCAGTATTCT 80

||||||||||||||||||||||||||||||||||||||||||||||||||||||||||||||||||||||||||||||||

Tgt 1 + GAAATGCGATAAGTAATGTGAATTGCAGAATTCAGTGAATCATCGAATCTTTGAACGCACATTGCGCCCGCCAGTATTCT 80

Qry 81 + GGCGGGCATGCCTGTTCGAGCGTCATTACAACCCTCAGGCCCCCGGGCCTGGCGTTGGGGATCGGCGAGGCGCCCCCTGC 160

||||||||||||||||||||||||||||||||||||||||||||||||||||||||||||||||||||||||||||||||

Tgt 81 + GGCGGGCATGCCTGTTCGAGCGTCATTACAACCCTCAGGCCCCCGGGCCTGGCGTTGGGGATCGGCGAGGCGCCCCCTGC 160

Qry 161 + GGGCACACGCCGTCCCCCAAATACAGTGGCGGGCCCGCCGCCGCCTCCCT 210

|||||||||||||||||||||||||||||||| ||| |||||||||||||

Tgt 161 + GGGCACACGCCGTCCCCCAAATACAGTGGCGGTCCCCCCGCCGCCTCCCT 210

210 cols, 208 ids (99.0%), 0 gaps (0.0%)

Query >Otu24

%Id TLen Target

99% 210 Otu40

98% 210 Otu33

Query 210nt >Otu24

Target 210nt >Otu40

Qry 1 + GAAATGCGATAAGTAATGTGAATTGCAGAATTCAGTGAATCATCGAATCTTTGAACGCACATTGCGCCCGCCAGTATTCT 80

||||||||||||||||||||||||||||||||||||||||||||||||||||||||||||||||||||||||||||||||

Tgt 1 + GAAATGCGATAAGTAATGTGAATTGCAGAATTCAGTGAATCATCGAATCTTTGAACGCACATTGCGCCCGCCAGTATTCT 80

Qry 81 + GGCGGGCATGCCTGTTCGAGCGTCATTACAACCCTCAGGCCCCCGGGCCTGGCGTTGGGGATCGGCGAGGCGCCCCCTGC 160

||||||||||||||||||||||||||||||||||||||||||||||||||||||||||||||||||||||||||||||||

Tgt 81 + GGCGGGCATGCCTGTTCGAGCGTCATTACAACCCTCAGGCCCCCGGGCCTGGCGTTGGGGATCGGCGAGGCGCCCCCTGC 160

Qry 161 + GGGCACACGCCGTCCCCCAAATACAGTGGCGGTCCCCCCGCCGCCTCCCT 210

|||||||||||||||||||||||||||||||| ||| |||| ||||||||

Tgt 161 + GGGCACACGCCGTCCCCCAAATACAGTGGCGGGCCCGCCGCAGCCTCCCT 210

210 cols, 207 ids (98.6%), 0 gaps (0.0%)

Query 210nt >Otu24

Target 210nt >Otu33

Qry 1 + GAAATGCGATAAGTAATGTGAATTGCAGAATTCAGTGAATCATCGAATCTTTGAACGCACATTGCGCCCGCCAGTATTCT 80

||||||||||||||||||||||||||||||||||||||||||||||||||||||||||||||||||||||||||||||||

Tgt 1 + GAAATGCGATAAGTAATGTGAATTGCAGAATTCAGTGAATCATCGAATCTTTGAACGCACATTGCGCCCGCCAGTATTCT 80

Qry 81 + GGCGGGCATGCCTGTTCGAGCGTCATTACAACCCTCAGGCCCCCGGGCCTGGCGTTGGGGATCGGCGAGGCGCCCCCTGC 160

||||||||||||||||||||||||||||||||||||||||||||||||||||||||||||||||||||||||||||||||

Tgt 81 + GGCGGGCATGCCTGTTCGAGCGTCATTACAACCCTCAGGCCCCCGGGCCTGGCGTTGGGGATCGGCGAGGCGCCCCCTGC 160

Qry 161 + GGGCACACGCCGTCCCCCAAATACAGTGGCGGTCCCCCCGCCGCCTCCCT 210

|||||||||||||||||||||||||||||||| ||| |||| || |||||

Tgt 161 + GGGCACACGCCGTCCCCCAAATACAGTGGCGGGCCCGCCGCAGCTTCCCT 210

210 cols, 206 ids (98.1%), 0 gaps (0.0%)

Query >Otu33

%Id TLen Target

100% 210 Otu40

Query 210nt >Otu33

Target 210nt >Otu40

Qry 1 + GAAATGCGATAAGTAATGTGAATTGCAGAATTCAGTGAATCATCGAATCTTTGAACGCACATTGCGCCCGCCAGTATTCT 80

||||||||||||||||||||||||||||||||||||||||||||||||||||||||||||||||||||||||||||||||

Tgt 1 + GAAATGCGATAAGTAATGTGAATTGCAGAATTCAGTGAATCATCGAATCTTTGAACGCACATTGCGCCCGCCAGTATTCT 80

Qry 81 + GGCGGGCATGCCTGTTCGAGCGTCATTACAACCCTCAGGCCCCCGGGCCTGGCGTTGGGGATCGGCGAGGCGCCCCCTGC 160

||||||||||||||||||||||||||||||||||||||||||||||||||||||||||||||||||||||||||||||||

Tgt 81 + GGCGGGCATGCCTGTTCGAGCGTCATTACAACCCTCAGGCCCCCGGGCCTGGCGTTGGGGATCGGCGAGGCGCCCCCTGC 160

Qry 161 + GGGCACACGCCGTCCCCCAAATACAGTGGCGGGCCCGCCGCAGCTTCCCT 210

|||||||||||||||||||||||||||||||||||||||||||| |||||

Tgt 161 + GGGCACACGCCGTCCCCCAAATACAGTGGCGGGCCCGCCGCAGCCTCCCT 210

210 cols, 209 ids (99.5%), 0 gaps (0.0%)

#============================================================================================

# 3. SSU

#============================================================================================

3.1 AALT (2 zOTUs)

Full sequences:

(n = sequence reads/2000 sequences after subsampling to even depth)

>Otu10 (n = 1761)

TAGTCATATGCTTGTCTCAAAGATTAAGCCATGCATGTCTAAGTATAAGCAATTATACCGTGAAACTGCGAATGGCTCAT

TAAATCAGTTATCGTTTATTTGATAATACCTTACTACTTGGATAACCGTGGTAATTCTAGAGCTAATACATGCTGAAAAT

CCCGACTTCGGAAGGGATGTGTTTATTAGATAAAAAACCAATGCCCTTCGGGG

>Otu20 (n = 237)

CAGTCATATGCTTGTCTCAAAGATTAAGCCATGCATGTCTAAGTATAAGCAATTATACCGTGAAACTGCGAATGGCTCAT

TAAATCAGTTATCGTTTATTTGATAATACCTTACTACTTGGATAACCGTGGTAATTCTAGAGCTAATACATGCTGAAAAT

CCCGACTTCGGAAGGGATGTGTTTATTAGATAAAAAACCAATGCCCTTCGGGG

---------------------------

Pairwise global alignments:

Query >Otu10

%Id TLen Target

100% 213 Otu20

Query 213nt >Otu10

Target 213nt >Otu20

Qry 1 + TAGTCATATGCTTGTCTCAAAGATTAAGCCATGCATGTCTAAGTATAAGCAATTATACCGTGAAACTGCGAATGGCTCAT 80

|||||||||||||||||||||||||||||||||||||||||||||||||||||||||||||||||||||||||||||||

Tgt 1 + CAGTCATATGCTTGTCTCAAAGATTAAGCCATGCATGTCTAAGTATAAGCAATTATACCGTGAAACTGCGAATGGCTCAT 80

Qry 81 + TAAATCAGTTATCGTTTATTTGATAATACCTTACTACTTGGATAACCGTGGTAATTCTAGAGCTAATACATGCTGAAAAT 160

||||||||||||||||||||||||||||||||||||||||||||||||||||||||||||||||||||||||||||||||

Tgt 81 + TAAATCAGTTATCGTTTATTTGATAATACCTTACTACTTGGATAACCGTGGTAATTCTAGAGCTAATACATGCTGAAAAT 160

Qry 161 + CCCGACTTCGGAAGGGATGTGTTTATTAGATAAAAAACCAATGCCCTTCGGGG 213

|||||||||||||||||||||||||||||||||||||||||||||||||||||

Tgt 161 + CCCGACTTCGGAAGGGATGTGTTTATTAGATAAAAAACCAATGCCCTTCGGGG 213

213 cols, 212 ids (99.5%), 0 gaps (0.0%)

#============================================================================================

3.2 ABRA (15 zOTUs)

Full sequences:

(n = sequence reads/2000 sequences after subsampling to even depth)

>Otu1 (n = 28)

TAGTCATATGCTTGTCTCAAAGATTAAGCCATGCATGTCTAAGTATAAACAAATTTATACCGTGAAACTGCGAATGGCTC

ATTAAATCAGTTATAGTTTATTTGATGGTATCTTGCTACATGGATAACTGTGGTAATTCTAGAGCTAATACATGCTTAAA

AGCCCCGACTTCTGGAAGGGGTGTATTTATTAGATAAAAAACCAATGACTTCG

>Otu3 (n = 456)

TAGTCATATGCTTGTCTCAAAGACTAAGCCATGCATGTCTAAGTATAAACAATTATACAGTGAAACTGCGAACGGCTCAT

TAAATCAGTTATCGTTTATTTGATAGTTTTCTACATGGATAACCGTGATAACTTCAGAACTAATACATGACAGCCTTCTG

GCGTATATATTAGATACAAACCAACAGTATGGTGATTCATAATATCTTGTCGA

>Otu4 (n = 41)

TAGTCATATGCTTGTCTCAAAGATTAAGCCATGCATGTCTAAGTATAAGCAATTTATACAGTGAAACTGCGAATGGCTCA

TTAAATCAGTTATCGTTTATTTGATAGTTCCTTTACTACATGGTATAACTGTGGTAATTCTAGAGCTAATACATGCTTAA

AATCTCGACCTCTTGGAAGAGATGTATTTATTAGATAAAAAATCAATGTCTTC

>Otu6 (n = 107)

TAGTCATATGCTTGTCTCAAAGATTAAGCCATGCATGTCTAAGTATAAACGAATTCATACTGTGAAACTGCGAATGGCTC

ATTAAATCAGTTATAGTTTATTTGATGGTATCTTGCTACATGGATAACTGTGGTAATTCTAGAGCTAATACATGCTGAAA

AGCCCCGACTTCTGGAAGGGGTGTATTTATTAGATAAAAAACCAATGGGTTTC

>Otu7 (n = 615)

TAGTCATATGCTTGTCTCAAAGATTAAGCCATGCATGTCTAAGTATAAGCACTTTATACTGTGAAACTGCGAATGGCTCA

TTAAATCAGTTATCGTTTATTTGATAGTACCTTACTACATGGATACCTGTGGTAATTCTAGAGCTAATACATGCTGAAAA

CCTCGACTTCGGAAGGGGTGTATTTATTAGATAAAAAACCAATGCCCTTCGGG

>Otu16 (n = 189)

TAGTCATATGCTTGTCTCAAAGATTAAGCCATGCATGTCTAAGTATAAGCAAATTGTACGGTGAAACTGCGAATGGCTCA

TTAAATCAGTTATCGTTTATTTGATAGTACCTTACTACATGGATACCTGTGGTAATTCTAGAGCTAATACATGCTAAAAA

CCTCGACTTCGGAAGGGGTGTATTTATTAGATAAAAAACCAATGCCCTTCGGG

>Otu14 (n = 42)

TAGTCATATGCTTGTCTCAAAGATTAAGCCATGCATGTCTAAGTATAAGCAATTTATACAGTGAAACTGCGAATGGCTCA

TTAAATCAGTTATCGTTTATTTGATAGTACCTTACTACTTGGATAACCGTGGTAATTCTAGAGCTAATACATGCTTAAAA

TCCCGACTGTTTGGAAGGGATGTATTTATTAGATAAAAAATCAATGTCTTCGG

>Otu18 (n = 52)

TAGTCATATGCTTGTCTCAAAGATTAAGCCATGCATGTCTAAGTATAAACAACTTTGTACTGTGAAACTGCGAATGGCTC

ATTAAATCAGTTATAGTTTATTTGATGGTTCTTGCTACATGGATAACTGTGGTAATTCTAGAGCTAATACATGCGTATAA

TCCCCGACTTCTGGAAGGGGTGTATTTATTAGATAAAAACCATCCCCTTTGGG

>Otu8 (n = 23)

TAGTCATATGCTTGTCTCAAAGATTAAGCCATGCATGTCTAAGTATAAGCAATTTATACGGTGAAACTGCGAATGGCTCA

TTAAATCAGTTATCGTTTATTTGATAGTACCTTACTACATGGATACCTGTGGTAATTCTAGAGCTAATACATGCTAAAAA

CCTCGACTTCGGAAGGGGTGTATTTATTAGATAAAAAACCAATGCCCTTCGGG

>Otu10 (n = 285)

TAGTCATATGCTTGTCTCAAAGATTAAGCCATGCATGTCTAAGTATAAGCAATTATACCGTGAAACTGCGAATGGCTCAT

TAAATCAGTTATCGTTTATTTGATAATACCTTACTACTTGGATAACCGTGGTAATTCTAGAGCTAATACATGCTGAAAAT

CCCGACTTCGGAAGGGATGTGTTTATTAGATAAAAAACCAATGCCCTTCGGGG

>Otu15 (n = 17)

TAGTCATATGCTTGTCTCAAAGATTAAGCCATGCATGTCTAAGTATAAACACGTTATACTGTGAAACTGCGAATGGCTCA

TTAAATCAGTTATAGTTTATTTGATGGTTTTTGCTACATGGATAACTGTGGTAATTCTAGAGCTAATACATGCGTATAAG

CCCCGACTTCTGGAAGGGGTGTATTTATTAGATAAAAATCAACACTGTTGGTG

>Otu12 (n = 35)

TAGTCATATGCTTGTCTCAAAGATTAAGCCATGCATGTCTAAGTATAAGCAATCATACTGTGAAACTGCGAATGGCTCAT

TAAATCAGTTATCGTTTATTTGATAGTACCTTTACCTGGATAACCGTGGTAATTCTAGAGCTAATACATGCTAACAACCC

CGACTTCGGGAGGGGTGTATTTATTAGATAAAAAACCAATGCCCTTCGGGGCT

>Otu9 (n = 23)

TAGTCATATGCTTGTCTCAAAGATTAAGCCATGCATGTCTAAGTATAAGCACTTTATACTGTGAAACTGCGAATGGCTCA

TTAAATCAGTTATCGTTTATTTGATAGTACCTTACTACATGGATACCTGTGGTAATTCTAGAGCTAATACATGCTAAAAA

CCTCGACTTCGGAAGGGGTGTATTTATTAGATAAAAAACCAATGCCCTTCGGG

>Otu17 (n = 15)

TAGTCATATGCTTGTCTCAAAGATTAAGCCATGCATGTCTAAGTATAAGCAACTATACGGTGAAACTGCGAATGGCTCAT

TAAATCAGTTATCGTTTATTTGATAGTACCTTACTACATGGATAACCGTGGTAATTCTAGAGCTAATACATGCTAAAAAC

CCCGACTTCGGAAGGGGTGTATTTATTAGATAAAAAACCAATGCCCTTCGGGG

>Otu20 (n = 68)

CAGTCATATGCTTGTCTCAAAGATTAAGCCATGCATGTCTAAGTATAAGCAATTATACCGTGAAACTGCGAATGGCTCAT

TAAATCAGTTATCGTTTATTTGATAATACCTTACTACTTGGATAACCGTGGTAATTCTAGAGCTAATACATGCTGAAAAT

CCCGACTTCGGAAGGGATGTGTTTATTAGATAAAAAACCAATGCCCTTCGGGG

---------------------------

Pairwise global alignments:

Query >Otu1

%Id TLen Target

96% 213 Otu6

94% 213 Otu18

92% 213 Otu8

92% 213 Otu15

92% 213 Otu7

92% 213 Otu9

92% 213 Otu16

92% 213 Otu17

91% 213 Otu14

91% 213 Otu10

90% 213 Otu4

90% 213 Otu20

90% 213 Otu12

78% 213 Otu3

Query 213nt >Otu1

Target 213nt >Otu6

Qry 1 + TAGTCATATGCTTGTCTCAAAGATTAAGCCATGCATGTCTAAGTATAAACAAATTTATACCGTGAAACTGCGAATGGCTC 80

|||||||||||||||||||||||||||||||||||||||||||||||||| |||| |||| |||||||||||||||||||

Tgt 1 + TAGTCATATGCTTGTCTCAAAGATTAAGCCATGCATGTCTAAGTATAAACGAATTCATACTGTGAAACTGCGAATGGCTC 80

Qry 81 + ATTAAATCAGTTATAGTTTATTTGATGGTATCTTGCTACATGGATAACTGTGGTAATTCTAGAGCTAATACATGCTTAAA 160

|||||||||||||||||||||||||||||||||||||||||||||||||||||||||||||||||||||||||||| |||

Tgt 81 + ATTAAATCAGTTATAGTTTATTTGATGGTATCTTGCTACATGGATAACTGTGGTAATTCTAGAGCTAATACATGCTGAAA 160

Qry 161 + AGCCCCGACTTCTGGAAGGGGTGTATTTATTAGATAAAAAACCAATGACTTCG 213

||||||||||||||||||||||||||||||||||||||||||||||| ||

Tgt 161 + AGCCCCGACTTCTGGAAGGGGTGTATTTATTAGATAAAAAACCAATGGGTTTC 213

213 cols, 205 ids (96.2%), 0 gaps (0.0%)

Query 213nt >Otu1

Target 213nt >Otu18

Qry 1 + TAGTCATATGCTTGTCTCAAAGATTAAGCCATGCATGTCTAAGTATAAACAAATTTATACCGTGAAACTGCGAATGGCTC 80

|||||||||||||||||||||||||||||||||||||||||||||||||||| ||| ||| |||||||||||||||||||

Tgt 1 + TAGTCATATGCTTGTCTCAAAGATTAAGCCATGCATGTCTAAGTATAAACAACTTTGTACTGTGAAACTGCGAATGGCTC 80

Qry 81 + ATTAAATCAGTTATAGTTTATTTGATGGTATCTTGCTACATGGATAACTGTGGTAATTCTAGAGCTAATACATGCTTAAA 160

||||||||||||||||||||||||||||| ||||||||||||||||||||||||||||||||||||||||||||| || |

Tgt 81 + ATTAAATCAGTTATAGTTTATTTGATGGT-TCTTGCTACATGGATAACTGTGGTAATTCTAGAGCTAATACATGCGTATA 159

Qry 161 + AGCCCCGACTTCTGGAAGGGGTGTATTTATTAGATAAAAAACCAATGACTTCG 213

| |||||||||||||||||||||||||||||||||||||| ||| ||| |

Tgt 160 + ATCCCCGACTTCTGGAAGGGGTGTATTTATTAGATAAAAA-CCATCCCCTTTG 211

213 cols, 200 ids (93.9%), 2 gaps (0.9%)

Query 213nt >Otu1

Target 213nt >Otu8

Qry 1 + TAGTCATATGCTTGTCTCAAAGATTAAGCCATGCATGTCTAAGTATAAACAAATTTATACCGTGAAACTGCGAATGGCTC 80

|||||||||||||||||||||||||||||||||||||||||||||||| | ||||||||| |||||||||||||||||||

Tgt 1 + TAGTCATATGCTTGTCTCAAAGATTAAGCCATGCATGTCTAAGTATAAGC-AATTTATACGGTGAAACTGCGAATGGCTC 79

Qry 81 + ATTAAATCAGTTATAGTTTATTTGATGGTATCTTGCTACATGGATAACTGTGGTAATTCTAGAGCTAATACATGCTTAAA 160

|||||||||||||| ||||||||||| ||| ||| ||||||||||| ||||||||||||||||||||||||||||| |||

Tgt 80 + ATTAAATCAGTTATCGTTTATTTGATAGTACCTTACTACATGGATACCTGTGGTAATTCTAGAGCTAATACATGCTAAAA 159

Qry 161 + AGCCCCGACTTCTGGAAGGGGTGTATTTATTAGATAAAAAACCAATGACTTCG 213

| || ||||||| |||||||||||||||||||||||||||||||||| | |

Tgt 160 + A-CCTCGACTTC-GGAAGGGGTGTATTTATTAGATAAAAAACCAATGCCCTTC 210

213 cols, 197 ids (92.5%), 3 gaps (1.4%)

Query 213nt >Otu1

Target 213nt >Otu15

Qry 1 + TAGTCATATGCTTGTCTCAAAGATTAAGCCATGCATGTCTAAGTATAAACAAATTTATACCGTGAAACTGCGAATGGCTC 80

||||||||||||||||||||||||||||||||||||||||||||||||||| |||||| |||||||||||||||||||

Tgt 1 + TAGTCATATGCTTGTCTCAAAGATTAAGCCATGCATGTCTAAGTATAAACA-CGTTATACTGTGAAACTGCGAATGGCTC 79

Qry 81 + ATTAAATCAGTTATAGTTTATTTGATGGTATCTTGCTACATGGATAACTGTGGTAATTCTAGAGCTAATACATGCTTAAA 160

||||||||||||||||||||||||||||| | ||||||||||||||||||||||||||||||||||||||||||| || |

Tgt 80 + ATTAAATCAGTTATAGTTTATTTGATGGT-TTTTGCTACATGGATAACTGTGGTAATTCTAGAGCTAATACATGCGTATA 158

Qry 161 + AGCCCCGACTTCTGGAAGGGGTGTATTTATTAGATAAAAAACCAATGACTTCG 213

|||||||||||||||||||||||||||||||||||||||| | | || |

Tgt 159 + AGCCCCGACTTCTGGAAGGGGTGTATTTATTAGATAAAAATCAACACTGTTGG 211

213 cols, 197 ids (92.5%), 2 gaps (0.9%)

Query 213nt >Otu1

Target 213nt >Otu7

Qry 1 + TAGTCATATGCTTGTCTCAAAGATTAAGCCATGCATGTCTAAGTATAAACAAATTTATACCGTGAAACTGCGAATGGCTC 80

|||||||||||||||||||||||||||||||||||||||||||||||| | | ||||||| |||||||||||||||||||

Tgt 1 + TAGTCATATGCTTGTCTCAAAGATTAAGCCATGCATGTCTAAGTATAAGC-ACTTTATACTGTGAAACTGCGAATGGCTC 79

Qry 81 + ATTAAATCAGTTATAGTTTATTTGATGGTATCTTGCTACATGGATAACTGTGGTAATTCTAGAGCTAATACATGCTTAAA 160

|||||||||||||| ||||||||||| ||| ||| ||||||||||| ||||||||||||||||||||||||||||| |||

Tgt 80 + ATTAAATCAGTTATCGTTTATTTGATAGTACCTTACTACATGGATACCTGTGGTAATTCTAGAGCTAATACATGCTGAAA 159

Qry 161 + AGCCCCGACTTCTGGAAGGGGTGTATTTATTAGATAAAAAACCAATGACTTCG 213

| || ||||||| |||||||||||||||||||||||||||||||||| | |

Tgt 160 + A-CCTCGACTTC-GGAAGGGGTGTATTTATTAGATAAAAAACCAATGCCCTTC 210

213 cols, 196 ids (92.0%), 3 gaps (1.4%)

Query 213nt >Otu1

Target 213nt >Otu9

Qry 1 + TAGTCATATGCTTGTCTCAAAGATTAAGCCATGCATGTCTAAGTATAAACAAATTTATACCGTGAAACTGCGAATGGCTC 80

|||||||||||||||||||||||||||||||||||||||||||||||| | | ||||||| |||||||||||||||||||

Tgt 1 + TAGTCATATGCTTGTCTCAAAGATTAAGCCATGCATGTCTAAGTATAAGC-ACTTTATACTGTGAAACTGCGAATGGCTC 79

Qry 81 + ATTAAATCAGTTATAGTTTATTTGATGGTATCTTGCTACATGGATAACTGTGGTAATTCTAGAGCTAATACATGCTTAAA 160

|||||||||||||| ||||||||||| ||| ||| ||||||||||| ||||||||||||||||||||||||||||| |||

Tgt 80 + ATTAAATCAGTTATCGTTTATTTGATAGTACCTTACTACATGGATACCTGTGGTAATTCTAGAGCTAATACATGCTAAAA 159

Qry 161 + AGCCCCGACTTCTGGAAGGGGTGTATTTATTAGATAAAAAACCAATGACTTCG 213

| || ||||||| |||||||||||||||||||||||||||||||||| | |

Tgt 160 + A-CCTCGACTTC-GGAAGGGGTGTATTTATTAGATAAAAAACCAATGCCCTTC 210

213 cols, 196 ids (92.0%), 3 gaps (1.4%)

Query 213nt >Otu1

Target 213nt >Otu16

Qry 1 + TAGTCATATGCTTGTCTCAAAGATTAAGCCATGCATGTCTAAGTATAAACAAATTTATACCGTGAAACTGCGAATGGCTC 80

|||||||||||||||||||||||||||||||||||||||||||||||| |||||| ||| |||||||||||||||||||

Tgt 1 + TAGTCATATGCTTGTCTCAAAGATTAAGCCATGCATGTCTAAGTATAAGCAAATT-GTACGGTGAAACTGCGAATGGCTC 79

Qry 81 + ATTAAATCAGTTATAGTTTATTTGATGGTATCTTGCTACATGGATAACTGTGGTAATTCTAGAGCTAATACATGCTTAAA 160

|||||||||||||| ||||||||||| ||| ||| ||||||||||| ||||||||||||||||||||||||||||| |||

Tgt 80 + ATTAAATCAGTTATCGTTTATTTGATAGTACCTTACTACATGGATACCTGTGGTAATTCTAGAGCTAATACATGCTAAAA 159

Qry 161 + AGCCCCGACTTCTGGAAGGGGTGTATTTATTAGATAAAAAACCAATGACTTCG 213

| || ||||||| |||||||||||||||||||||||||||||||||| | |

Tgt 160 + A-CCTCGACTTC-GGAAGGGGTGTATTTATTAGATAAAAAACCAATGCCCTTC 210

213 cols, 196 ids (92.0%), 3 gaps (1.4%)

Query 213nt >Otu1

Target 213nt >Otu17

Qry 1 + TAGTCATATGCTTGTCTCAAAGATTAAGCCATGCATGTCTAAGTATAAACAAATTTATACCGTGAAACTGCGAATGGCTC 80

|||||||||||||||||||||||||||||||||||||||||||||||| ||| ||||| |||||||||||||||||||

Tgt 1 + TAGTCATATGCTTGTCTCAAAGATTAAGCCATGCATGTCTAAGTATAAGCAA--CTATACGGTGAAACTGCGAATGGCTC 78

Qry 81 + ATTAAATCAGTTATAGTTTATTTGATGGTATCTTGCTACATGGATAACTGTGGTAATTCTAGAGCTAATACATGCTTAAA 160

|||||||||||||| ||||||||||| ||| ||| ||||||||||||| ||||||||||||||||||||||||||| |||

Tgt 79 + ATTAAATCAGTTATCGTTTATTTGATAGTACCTTACTACATGGATAACCGTGGTAATTCTAGAGCTAATACATGCTAAAA 158

Qry 161 + AGCCCCGACTTCTGGAAGGGGTGTATTTATTAGATAAAAAACCAATGACTTCG 213

| ||| |||||| |||||||||||||||||||||||||||||||||| | |

Tgt 159 + ACCCC-GACTTC-GGAAGGGGTGTATTTATTAGATAAAAAACCAATGCCCTTC 209

213 cols, 195 ids (91.5%), 4 gaps (1.9%)

Query 213nt >Otu1

Target 213nt >Otu14

Qry 1 + TAGTCATATGCTTGTCTCAAAGATTAAGCCATGCATGTCTAAGTATAAACAAATTTATACCGTGAAACTGCGAATGGCTC 80

|||||||||||||||||||||||||||||||||||||||||||||||| | ||||||||| |||||||||||||||||||

Tgt 1 + TAGTCATATGCTTGTCTCAAAGATTAAGCCATGCATGTCTAAGTATAAGC-AATTTATACAGTGAAACTGCGAATGGCTC 79

Qry 81 + ATTAAATCAGTTATAGTTTATTTGATGGTATCTTGCTACATGGATAACTGTGGTAATTCTAGAGCTAATACATGCTTAAA 160

|||||||||||||| ||||||||||| ||| ||| |||| |||||||| |||||||||||||||||||||||||||||||

Tgt 80 + ATTAAATCAGTTATCGTTTATTTGATAGTACCTTACTACTTGGATAACCGTGGTAATTCTAGAGCTAATACATGCTTAAA 159

Qry 161 + AGCCCCGACTTCTGGAAGGGGTGTATTTATTAGATAAAAAACCAATGACTTCG 213

| ||| | |||||||| |||||||||||||||||||| ||||| |||||

Tgt 160 + ATCCCGACTGTTTGGAAGGGATGTATTTATTAGATAAAAAATCAATGTCTTCG 212

213 cols, 194 ids (91.1%), 1 gaps (0.5%)

Query 213nt >Otu1

Target 213nt >Otu10

Qry 1 + TAGTCATATGCTTGTCTCAAAGATTAAGCCATGCATGTCTAAGTATAAACAAATTTATACCGTGAAACTGCGAATGGCTC 80

|||||||||||||||||||||||||||||||||||||||||||||||| ||| ||||||||||||||||||||||||||

Tgt 1 + TAGTCATATGCTTGTCTCAAAGATTAAGCCATGCATGTCTAAGTATAAGCAA--TTATACCGTGAAACTGCGAATGGCTC 78

Qry 81 + ATTAAATCAGTTATAGTTTATTTGATGGTATCTTGCTACATGGATAACTGTGGTAATTCTAGAGCTAATACATGCTTAAA 160

|||||||||||||| ||||||||||| || ||| |||| |||||||| ||||||||||||||||||||||||||| |||

Tgt 79 + ATTAAATCAGTTATCGTTTATTTGATAATACCTTACTACTTGGATAACCGTGGTAATTCTAGAGCTAATACATGCTGAAA 158

Qry 161 + AGCCCCGACTTCTGGAAGGGGTGTATTTATTAGATAAAAAACCAATGACTTCG 213

| ||| |||||| ||||||| ||| |||||||||||||||||||||| | |

Tgt 159 + ATCCC-GACTTC-GGAAGGGATGTGTTTATTAGATAAAAAACCAATGCCCTTC 209

213 cols, 193 ids (90.6%), 4 gaps (1.9%)

Query 213nt >Otu1

Target 213nt >Otu4

Qry 1 + TAGTCATATGCTTGTCTCAAAGATTAAGCCATGCATGTCTAAGTATAAACAAATTTATACCGTGAAACTGCGAATGGCTC 80

|||||||||||||||||||||||||||||||||||||||||||||||| ||| ||||||| |||||||||||||||||||

Tgt 1 + TAGTCATATGCTTGTCTCAAAGATTAAGCCATGCATGTCTAAGTATAAGCAA-TTTATACAGTGAAACTGCGAATGGCTC 79

Qry 81 + ATTAAATCAGTTATAGTTTATTTGATGGTATC-TTGCTACATGG-ATAACTGTGGTAATTCTAGAGCTAATACATGCTTA 158

|||||||||||||| ||||||||||| || | || |||||||| |||||||||||||||||||||||||||||||||||

Tgt 80 + ATTAAATCAGTTATCGTTTATTTGATAGTTCCTTTACTACATGGTATAACTGTGGTAATTCTAGAGCTAATACATGCTTA 159

Qry 159 + AAAGCCCCGACTTCTGGAAGGGGTGTATTTATTAGATAAAAAACCAATGACTTC 212

||| | | || |||||| | |||||||||||||||||||| ||||| ||||

Tgt 160 + AAATCTCGACCTCTTGGAAGAGATGTATTTATTAGATAAAAAATCAATGTCTTC 213

214 cols, 193 ids (90.2%), 3 gaps (1.4%)

Query 213nt >Otu1

Target 213nt >Otu20

Qry 1 + TAGTCATATGCTTGTCTCAAAGATTAAGCCATGCATGTCTAAGTATAAACAAATTTATACCGTGAAACTGCGAATGGCTC 80

||||||||||||||||||||||||||||||||||||||||||||||| ||| ||||||||||||||||||||||||||

Tgt 1 + CAGTCATATGCTTGTCTCAAAGATTAAGCCATGCATGTCTAAGTATAAGCAA--TTATACCGTGAAACTGCGAATGGCTC 78

Qry 81 + ATTAAATCAGTTATAGTTTATTTGATGGTATCTTGCTACATGGATAACTGTGGTAATTCTAGAGCTAATACATGCTTAAA 160

|||||||||||||| ||||||||||| || ||| |||| |||||||| ||||||||||||||||||||||||||| |||

Tgt 79 + ATTAAATCAGTTATCGTTTATTTGATAATACCTTACTACTTGGATAACCGTGGTAATTCTAGAGCTAATACATGCTGAAA 158

Qry 161 + AGCCCCGACTTCTGGAAGGGGTGTATTTATTAGATAAAAAACCAATGACTTCG 213

| ||| |||||| ||||||| ||| |||||||||||||||||||||| | |

Tgt 159 + ATCCC-GACTTC-GGAAGGGATGTGTTTATTAGATAAAAAACCAATGCCCTTC 209

213 cols, 192 ids (90.1%), 4 gaps (1.9%)

Query 213nt >Otu1

Target 213nt >Otu12

Qry 1 + TAGTCATATGCTTGTCTCAAAGATTAAGCCATGCATGTCTAAGTATAAACAAATTTATACCGTGAAACTGCGAATGGCTC 80

|||||||||||||||||||||||||||||||||||||||||||||||| ||| | |||| |||||||||||||||||||

Tgt 1 + TAGTCATATGCTTGTCTCAAAGATTAAGCCATGCATGTCTAAGTATAAGCAA--TCATACTGTGAAACTGCGAATGGCTC 78

Qry 81 + ATTAAATCAGTTATAGTTTATTTGATGGTATCTTGCTACATGGATAACTGTGGTAATTCTAGAGCTAATACATGCTTAAA 160

|||||||||||||| ||||||||||| ||| ||| ||| |||||||| ||||||||||||||||||||||||||| ||

Tgt 79 + ATTAAATCAGTTATCGTTTATTTGATAGTACCTT--TACCTGGATAACCGTGGTAATTCTAGAGCTAATACATGCT-AAC 155

Qry 161 + AGCCCCGACTTCTGGAAGGGGTGTATTTATTAGATAAAAAACCAATGACTTCG 213

| |||||||||| || ||||||||||||||||||||||||||||||| | |

Tgt 156 + AACCCCGACTTC-GGGAGGGGTGTATTTATTAGATAAAAAACCAATGCCCTTC 207

213 cols, 192 ids (90.1%), 6 gaps (2.8%)

Query 213nt >Otu1

Target 213nt >Otu3

Qry 1 + TAGTCATATGCTTGTCTCAAAGATTAAGCCATGCATGTCTAAGTATAAACAAATTTATACCGTGAAACTGCGAATGGCTC 80

||||||||||||||||||||||| |||||||||||||||||||||||||||| |||||| ||||||||||||| |||||

Tgt 1 + TAGTCATATGCTTGTCTCAAAGACTAAGCCATGCATGTCTAAGTATAAACAA--TTATACAGTGAAACTGCGAACGGCTC 78

Qry 81 + ATTAAATCAGTTATAGTTTATTTGATGGTATCTTGCTACATGGATAACTGTGGTAATTCTAGAGCTAATACATGCTTAAA 160

|||||||||||||| ||||||||||| || || ||||||||||||| ||| ||| | ||| ||||||||||

Tgt 79 + ATTAAATCAGTTATCGTTTATTTGATAGT---TTTCTACATGGATAACCGTGATAACTTCAGAACTAATACATG------ 149

Qry 161 + AGCCCCGACTTCTGGAAGGGGTGTATTTATTAGATAAAAAACCAATGACTTCG 213

| | ||||| || |||| ||||||||| ||| | | | |

Tgt 150 + ---ACAGCCTTCT------GGCGTATATATTAGATACAAACCAACAGTATGGT 193

213 cols, 166 ids (77.9%), 20 gaps (9.4%)

Query >Otu3

%Id TLen Target

81% 213 Otu15

79% 213 Otu17

79% 213 Otu18

78% 213 Otu12

78% 213 Otu10

77% 213 Otu20

77% 213 Otu8

77% 213 Otu6

77% 213 Otu9

77% 213 Otu16

77% 213 Otu4

77% 213 Otu14

77% 213 Otu7

Query 213nt >Otu3

Target 213nt >Otu15

Qry 1 + TAGTCATATGCTTGTCTCAAAGACTAAGCCATGCATGTCTAAGTATAAACA-ATTATACAGTGAAACTGCGAACGGCTCA 79

||||||||||||||||||||||| ||||||||||||||||||||||||||| |||||| ||||||||||||| ||||||

Tgt 1 + TAGTCATATGCTTGTCTCAAAGATTAAGCCATGCATGTCTAAGTATAAACACGTTATACTGTGAAACTGCGAATGGCTCA 80

Qry 80 + TTAAATCAGTTATCGTTTATTTGATAGTTTT--CTACATGGATAACCGTGATAACTTCAGAACTAATACATG-------- 149

||||||||||||| ||||||||||| ||||| ||||||||||||| ||| ||| | ||| ||||||||||

Tgt 81 + TTAAATCAGTTATAGTTTATTTGATGGTTTTTGCTACATGGATAACTGTGGTAATTCTAGAGCTAATACATGCGTATAAG 160

Qry 150 + -ACAGCCTTCT------GGCGTATATATTAGATACAAACCAACAGT-ATGGTG 194

| | ||||| || |||| ||||||||| ||| ||||| | |||||

Tgt 161 + CCCCGACTTCTGGAAGGGGTGTATTTATTAGATAAAAATCAACACTGTTGGTG 213

213 cols, 173 ids (81.2%), 19 gaps (8.9%)

Query 213nt >Otu3

Target 213nt >Otu17

Qry 1 + TAGTCATATGCTTGTCTCAAAGACTAAGCCATGCATGTCTAAGTATAAACAATTATACAGTGAAACTGCGAACGGCTCAT 80

||||||||||||||||||||||| |||||||||||||||||||||||| ||| ||||| ||||||||||||| |||||||

Tgt 1 + TAGTCATATGCTTGTCTCAAAGATTAAGCCATGCATGTCTAAGTATAAGCAACTATACGGTGAAACTGCGAATGGCTCAT 80

Qry 81 + TAAATCAGTTATCGTTTATTTGATAGT---TTTCTACATGGATAACCGTGATAACTTCAGAACTAATACATG-------- 149

||||||||||||||||||||||||||| || ||||||||||||||||| ||| | ||| ||||||||||

Tgt 81 + TAAATCAGTTATCGTTTATTTGATAGTACCTTACTACATGGATAACCGTGGTAATTCTAGAGCTAATACATGCTAAAAAC 160

Qry 150 + ACAGCCTTC-----TGGCGTATATATTAGATACAAACCAA---CAGTATGGTG 194

| | |||| || |||| ||||||||| ||| | | | | || |

Tgt 161 + CCCGACTTCGGAAGGGGTGTATTTATTAGATAAAAAACCAATGCCCTTCGGGG 213

213 cols, 169 ids (79.3%), 19 gaps (8.9%)

Query 213nt >Otu3

Target 213nt >Otu18

Qry 1 + TAGTCATATGCTTGTCTCAAAGACTAAGCCATGCATGTCTAAGTATAAACAA--TTATACAGTGAAACTGCGAACGGCTC 78

||||||||||||||||||||||| |||||||||||||||||||||||||||| || ||| ||||||||||||| |||||

Tgt 1 + TAGTCATATGCTTGTCTCAAAGATTAAGCCATGCATGTCTAAGTATAAACAACTTTGTACTGTGAAACTGCGAATGGCTC 80

Qry 79 + ATTAAATCAGTTATCGTTTATTTGATAGTT--TTCTACATGGATAACCGTGATAACTTCAGAACTAATACATG------- 149

|||||||||||||| ||||||||||| ||| | ||||||||||||| ||| ||| | ||| ||||||||||

Tgt 81 + ATTAAATCAGTTATAGTTTATTTGATGGTTCTTGCTACATGGATAACTGTGGTAATTCTAGAGCTAATACATGCGTATAA 160

Qry 150 + --ACAGCCTTCT------GGCGTATATATTAGATACAAACCAACAGTATGGTG 194

| | ||||| || |||| ||||||||| |||||| | | | |

Tgt 161 + TCCCCGACTTCTGGAAGGGGTGTATTTATTAGATAAAAACCATCCCCTTTGGG 213

213 cols, 168 ids (78.9%), 19 gaps (8.9%)

Query 213nt >Otu3

Target 213nt >Otu12

Qry 1 + TAGTCATATGCTTGTCTCAAAGACTAAGCCATGCATGTCTAAGTATAAACAATTATACAGTGAAACTGCGAACGGCTCAT 80

||||||||||||||||||||||| |||||||||||||||||||||||| |||| |||| ||||||||||||| |||||||

Tgt 1 + TAGTCATATGCTTGTCTCAAAGATTAAGCCATGCATGTCTAAGTATAAGCAATCATACTGTGAAACTGCGAATGGCTCAT 80

Qry 81 + TAAATCAGTTATCGTTTATTTGATAGT-TTTCTACATGGATAACCGTGATAACTTCAGAACTAATACATG--------AC 151

||||||||||||||||||||||||||| | ||| |||||||||||| ||| | ||| |||||||||| |

Tgt 81 + TAAATCAGTTATCGTTTATTTGATAGTACCTTTACCTGGATAACCGTGGTAATTCTAGAGCTAATACATGCTAACAACCC 160

Qry 152 + AGCCTTC-----TGGCGTATATATTAGATACAAACCAA---CAGTATGGTGAT 196

| |||| || |||| ||||||||| ||| | | | | || | |

Tgt 161 + CGACTTCGGGAGGGGTGTATTTATTAGATAAAAAACCAATGCCCTTCGGGGCT 213

213 cols, 167 ids (78.4%), 17 gaps (8.0%)

Query 213nt >Otu3

Target 213nt >Otu10

Qry 1 + TAGTCATATGCTTGTCTCAAAGACTAAGCCATGCATGTCTAAGTATAAACAATTATACAGTGAAACTGCGAACGGCTCAT 80

||||||||||||||||||||||| |||||||||||||||||||||||| ||||||||| ||||||||||||| |||||||

Tgt 1 + TAGTCATATGCTTGTCTCAAAGATTAAGCCATGCATGTCTAAGTATAAGCAATTATACCGTGAAACTGCGAATGGCTCAT 80

Qry 81 + TAAATCAGTTATCGTTTATTTGATAGT---TTTCTACATGGATAACCGTGATAACTTCAGAACTAATACATG-------- 149

||||||||||||||||||||||||| | || |||| |||||||||||| ||| | ||| ||||||||||

Tgt 81 + TAAATCAGTTATCGTTTATTTGATAATACCTTACTACTTGGATAACCGTGGTAATTCTAGAGCTAATACATGCTGAAAAT 160

Qry 150 + ACAGCCTTC-----TGGCGTATATATTAGATACAAACCAA---CAGTATGGTG 194

| | |||| | || | ||||||||| ||| | | | | || |

Tgt 161 + CCCGACTTCGGAAGGGATGTGTTTATTAGATAAAAAACCAATGCCCTTCGGGG 213

213 cols, 166 ids (77.9%), 19 gaps (8.9%)

Query 213nt >Otu3

Target 213nt >Otu20

Qry 1 + TAGTCATATGCTTGTCTCAAAGACTAAGCCATGCATGTCTAAGTATAAACAATTATACAGTGAAACTGCGAACGGCTCAT 80

|||||||||||||||||||||| |||||||||||||||||||||||| ||||||||| ||||||||||||| |||||||

Tgt 1 + CAGTCATATGCTTGTCTCAAAGATTAAGCCATGCATGTCTAAGTATAAGCAATTATACCGTGAAACTGCGAATGGCTCAT 80

Qry 81 + TAAATCAGTTATCGTTTATTTGATAGT---TTTCTACATGGATAACCGTGATAACTTCAGAACTAATACATG-------- 149

||||||||||||||||||||||||| | || |||| |||||||||||| ||| | ||| ||||||||||

Tgt 81 + TAAATCAGTTATCGTTTATTTGATAATACCTTACTACTTGGATAACCGTGGTAATTCTAGAGCTAATACATGCTGAAAAT 160

Qry 150 + ACAGCCTTC-----TGGCGTATATATTAGATACAAACCAA---CAGTATGGTG 194

| | |||| | || | ||||||||| ||| | | | | || |

Tgt 161 + CCCGACTTCGGAAGGGATGTGTTTATTAGATAAAAAACCAATGCCCTTCGGGG 213

213 cols, 165 ids (77.5%), 19 gaps (8.9%)

Query 213nt >Otu3

Target 213nt >Otu8

Qry 1 + TAGTCATATGCTTGTCTCAAAGACTAAGCCATGCATGTCTAAGTATAAACAA-TTATACAGTGAAACTGCGAACGGCTCA 79

||||||||||||||||||||||| |||||||||||||||||||||||| ||| |||||| ||||||||||||| ||||||

Tgt 1 + TAGTCATATGCTTGTCTCAAAGATTAAGCCATGCATGTCTAAGTATAAGCAATTTATACGGTGAAACTGCGAATGGCTCA 80

Qry 80 + TTAAATCAGTTATCGTTTATTTGATAGT---TTTCTACATGGATAACCGTGATAACTTCAGAACTAATACATGACA---- 152

|||||||||||||||||||||||||||| || ||||||||||| | ||| ||| | ||| |||||||||| |

Tgt 81 + TTAAATCAGTTATCGTTTATTTGATAGTACCTTACTACATGGATACCTGTGGTAATTCTAGAGCTAATACATGCTAAAAA 160

Qry 153 + ----GCCTTC-----TGGCGTATATATTAGATACAAACCAACAGTATGGTGAT 196

| |||| || |||| ||||||||| ||| | | | |

Tgt 161 + CCTCGACTTCGGAAGGGGTGTATTTATTAGATAAAAAACCAATGCCCTTCGGG 213

213 cols, 165 ids (77.5%), 17 gaps (8.0%)

Query 213nt >Otu3

Target 213nt >Otu6

Qry 1 + TAGTCATATGCTTGTCTCAAAGACTAAGCCATGCATGTCTAAGTATAAAC--AATTATACAGTGAAACTGCGAACGGCTC 78

||||||||||||||||||||||| |||||||||||||||||||||||||| | | |||| ||||||||||||| |||||

Tgt 1 + TAGTCATATGCTTGTCTCAAAGATTAAGCCATGCATGTCTAAGTATAAACGAATTCATACTGTGAAACTGCGAATGGCTC 80

Qry 79 + ATTAAATCAGTTATCGTTTATTTGATAGT---TTTCTACATGGATAACCGTGATAACTTCAGAACTAATACATG------ 149

|||||||||||||| ||||||||||| || || ||||||||||||| ||| ||| | ||| ||||||||||

Tgt 81 + ATTAAATCAGTTATAGTTTATTTGATGGTATCTTGCTACATGGATAACTGTGGTAATTCTAGAGCTAATACATGCTGAAA 160

Qry 150 + ---ACAGCCTTCT------GGCGTATATATTAGATACAAACCAACAGTATGGT 193

| | ||||| || |||| ||||||||| ||| | | | |

Tgt 161 + AGCCCCGACTTCTGGAAGGGGTGTATTTATTAGATAAAAAACCAATGGGTTTC 213

213 cols, 164 ids (77.0%), 20 gaps (9.4%)

Query 213nt >Otu3

Target 213nt >Otu9

Qry 1 + TAGTCATATGCTTGTCTCAAAGACTAAGCCATGCATGTCTAAGTATAAACA-ATTATACAGTGAAACTGCGAACGGCTCA 79

||||||||||||||||||||||| |||||||||||||||||||||||| || |||||| ||||||||||||| ||||||

Tgt 1 + TAGTCATATGCTTGTCTCAAAGATTAAGCCATGCATGTCTAAGTATAAGCACTTTATACTGTGAAACTGCGAATGGCTCA 80

Qry 80 + TTAAATCAGTTATCGTTTATTTGATAGT---TTTCTACATGGATAACCGTGATAACTTCAGAACTAATACATGACA---- 152

|||||||||||||||||||||||||||| || ||||||||||| | ||| ||| | ||| |||||||||| |

Tgt 81 + TTAAATCAGTTATCGTTTATTTGATAGTACCTTACTACATGGATACCTGTGGTAATTCTAGAGCTAATACATGCTAAAAA 160

Qry 153 + ----GCCTTC-----TGGCGTATATATTAGATACAAACCAACAGTATGGTGAT 196

| |||| || |||| ||||||||| ||| | | | |

Tgt 161 + CCTCGACTTCGGAAGGGGTGTATTTATTAGATAAAAAACCAATGCCCTTCGGG 213

213 cols, 164 ids (77.0%), 17 gaps (8.0%)

Query 213nt >Otu3

Target 213nt >Otu16

Qry 1 + TAGTCATATGCTTGTCTCAAAGACTAAGCCATGCATGTCTAAGTATAAAC-AATTATACAGTGAAACTGCGAACGGCTCA 79

||||||||||||||||||||||| |||||||||||||||||||||||| | |||| ||| ||||||||||||| ||||||

Tgt 1 + TAGTCATATGCTTGTCTCAAAGATTAAGCCATGCATGTCTAAGTATAAGCAAATTGTACGGTGAAACTGCGAATGGCTCA 80

Qry 80 + TTAAATCAGTTATCGTTTATTTGATAGT---TTTCTACATGGATAACCGTGATAACTTCAGAACTAATACATGACA---- 152

|||||||||||||||||||||||||||| || ||||||||||| | ||| ||| | ||| |||||||||| |

Tgt 81 + TTAAATCAGTTATCGTTTATTTGATAGTACCTTACTACATGGATACCTGTGGTAATTCTAGAGCTAATACATGCTAAAAA 160

Qry 153 + ----GCCTTC-----TGGCGTATATATTAGATACAAACCAACAGTATGGTGAT 196

| |||| || |||| ||||||||| ||| | | | |

Tgt 161 + CCTCGACTTCGGAAGGGGTGTATTTATTAGATAAAAAACCAATGCCCTTCGGG 213

213 cols, 164 ids (77.0%), 17 gaps (8.0%)

Query 213nt >Otu3

Target 213nt >Otu4

Qry 1 + TAGTCATATGCTTGTCTCAAAGACTAAGCCATGCATGTCTAAGTATAAACAA-TTATACAGTGAAACTGCGAACGGCTCA 79

||||||||||||||||||||||| |||||||||||||||||||||||| ||| |||||||||||||||||||| ||||||

Tgt 1 + TAGTCATATGCTTGTCTCAAAGATTAAGCCATGCATGTCTAAGTATAAGCAATTTATACAGTGAAACTGCGAATGGCTCA 80

Qry 80 + TTAAATCAGTTATCGTTTATTTGATAGTT----TTCTACATGG-ATAACCGTGATAACTTCAGAACTAATACATGACAGC 154

||||||||||||||||||||||||||||| | |||||||| ||||| ||| ||| | ||| ||||||||||

Tgt 81 + TTAAATCAGTTATCGTTTATTTGATAGTTCCTTTACTACATGGTATAACTGTGGTAATTCTAGAGCTAATACATGCTTAA 160

Qry 155 + CTTCT---------------GGCGTATATATTAGATACAAACCAACAGTATGG 192

||| | |||| ||||||||| ||| | || |

Tgt 161 + AATCTCGACCTCTTGGAAGAGATGTATTTATTAGATAAAAAATCAATGTCTTC 213

213 cols, 163 ids (76.5%), 21 gaps (9.9%)

Query 213nt >Otu3

Target 213nt >Otu14

Qry 1 + TAGTCATATGCTTGTCTCAAAGACTAAGCCATGCATGTCTAAGTATAAACAA-TTATACAGTGAAACTGCGAACGGCTCA 79

||||||||||||||||||||||| |||||||||||||||||||||||| ||| |||||||||||||||||||| ||||||

Tgt 1 + TAGTCATATGCTTGTCTCAAAGATTAAGCCATGCATGTCTAAGTATAAGCAATTTATACAGTGAAACTGCGAATGGCTCA 80

Qry 80 + TTAAATCAGTTATCGTTTATTTGATAGT---TTTCTACATGGATAACCGTGATAACTTCAGAACTAATACATGACAGCCT 156

|||||||||||||||||||||||||||| || |||| |||||||||||| ||| | ||| ||||||||||

Tgt 81 + TTAAATCAGTTATCGTTTATTTGATAGTACCTTACTACTTGGATAACCGTGGTAATTCTAGAGCTAATACATGCTTAAAA 160

Qry 157 + TC---------------TGGCGTATATATTAGATACAAACCAACAGTATGGTG 194

|| | |||| ||||||||| ||| | || | |

Tgt 161 + TCCCGACTGTTTGGAAGGGATGTATTTATTAGATAAAAAATCAATGTCTTCGG 213

213 cols, 163 ids (76.5%), 19 gaps (8.9%)

Query 213nt >Otu3

Target 213nt >Otu7

Qry 1 + TAGTCATATGCTTGTCTCAAAGACTAAGCCATGCATGTCTAAGTATAAACA-ATTATACAGTGAAACTGCGAACGGCTCA 79

||||||||||||||||||||||| |||||||||||||||||||||||| || |||||| ||||||||||||| ||||||

Tgt 1 + TAGTCATATGCTTGTCTCAAAGATTAAGCCATGCATGTCTAAGTATAAGCACTTTATACTGTGAAACTGCGAATGGCTCA 80

Qry 80 + TTAAATCAGTTATCGTTTATTTGATAGT---TTTCTACATGGATAACCGTGATAACTTCAGAACTAATACATG---ACAG 153

|||||||||||||||||||||||||||| || ||||||||||| | ||| ||| | ||| |||||||||| | |

Tgt 81 + TTAAATCAGTTATCGTTTATTTGATAGTACCTTACTACATGGATACCTGTGGTAATTCTAGAGCTAATACATGCTGAAAA 160

Qry 154 + CCT----------TCTGGCGTATATATTAGATACAAACCAACAGTATGGTGAT 196

||| || |||| ||||||||| ||| | | | |

Tgt 161 + CCTCGACTTCGGAAGGGGTGTATTTATTAGATAAAAAACCAATGCCCTTCGGG 213

213 cols, 163 ids (76.5%), 17 gaps (8.0%)

Query >Otu4

%Id TLen Target

95% 213 Otu14

92% 213 Otu8

92% 213 Otu9

92% 213 Otu7

92% 213 Otu16

91% 213 Otu17

91% 213 Otu10

91% 213 Otu20

88% 213 Otu6

88% 213 Otu12

87% 213 Otu18

86% 213 Otu15

Query 213nt >Otu4

Target 213nt >Otu14

Qry 1 + TAGTCATATGCTTGTCTCAAAGATTAAGCCATGCATGTCTAAGTATAAGCAATTTATACAGTGAAACTGCGAATGGCTCA 80

||||||||||||||||||||||||||||||||||||||||||||||||||||||||||||||||||||||||||||||||

Tgt 1 + TAGTCATATGCTTGTCTCAAAGATTAAGCCATGCATGTCTAAGTATAAGCAATTTATACAGTGAAACTGCGAATGGCTCA 80

Qry 81 + TTAAATCAGTTATCGTTTATTTGATAGTTCCTTTACTACATGGTATAACTGTGGTAATTCTAGAGCTAATACATGCTTAA 160

|||||||||||||||||||||||||||| |||| ||||| ||| ||||| ||||||||||||||||||||||||||||||

Tgt 81 + TTAAATCAGTTATCGTTTATTTGATAGTACCTT-ACTACTTGG-ATAACCGTGGTAATTCTAGAGCTAATACATGCTTAA 158

Qry 161 + AATCTCGACCTCTTGGAAGAGATGTATTTATTAGATAAAAAATCAATGTCTTC 213

|||| |||| ||||||| |||||||||||||||||||||||||||||||||

Tgt 159 + AATCCCGACTGTTTGGAAGGGATGTATTTATTAGATAAAAAATCAATGTCTTC 211

213 cols, 203 ids (95.3%), 2 gaps (0.9%)

Query 213nt >Otu4

Target 213nt >Otu8

Qry 1 + TAGTCATATGCTTGTCTCAAAGATTAAGCCATGCATGTCTAAGTATAAGCAATTTATACAGTGAAACTGCGAATGGCTCA 80

||||||||||||||||||||||||||||||||||||||||||||||||||||||||||| ||||||||||||||||||||

Tgt 1 + TAGTCATATGCTTGTCTCAAAGATTAAGCCATGCATGTCTAAGTATAAGCAATTTATACGGTGAAACTGCGAATGGCTCA 80

Qry 81 + TTAAATCAGTTATCGTTTATTTGATAGTTCCTTTACTACATGGTATAACTGTGGTAATTCTAGAGCTAATACATGCTTAA 160

|||||||||||||||||||||||||||| |||| ||||||||| ||| ||||||||||||||||||||||||||||| ||

Tgt 81 + TTAAATCAGTTATCGTTTATTTGATAGTACCTT-ACTACATGG-ATACCTGTGGTAATTCTAGAGCTAATACATGCTAAA 158

Qry 161 + AATCTCGACCTCTTGGAAGAGATGTATTTATTAGATAAAAAATCAATGTCTTC 213

|| |||||| || ||||| | |||||||||||||||||||| ||||| | |

Tgt 159 + AACCTCGACTTC--GGAAGGGGTGTATTTATTAGATAAAAAACCAATGCCCTT 209

213 cols, 197 ids (92.5%), 4 gaps (1.9%)

Query 213nt >Otu4

Target 213nt >Otu9

Qry 1 + TAGTCATATGCTTGTCTCAAAGATTAAGCCATGCATGTCTAAGTATAAGCAATTTATACAGTGAAACTGCGAATGGCTCA 80

||||||||||||||||||||||||||||||||||||||||||||||||||| ||||||| ||||||||||||||||||||

Tgt 1 + TAGTCATATGCTTGTCTCAAAGATTAAGCCATGCATGTCTAAGTATAAGCACTTTATACTGTGAAACTGCGAATGGCTCA 80

Qry 81 + TTAAATCAGTTATCGTTTATTTGATAGTTCCTTTACTACATGGTATAACTGTGGTAATTCTAGAGCTAATACATGCTTAA 160

|||||||||||||||||||||||||||| |||| ||||||||| ||| ||||||||||||||||||||||||||||| ||

Tgt 81 + TTAAATCAGTTATCGTTTATTTGATAGTACCTT-ACTACATGG-ATACCTGTGGTAATTCTAGAGCTAATACATGCTAAA 158

Qry 161 + AATCTCGACCTCTTGGAAGAGATGTATTTATTAGATAAAAAATCAATGTCTTC 213

|| |||||| || ||||| | |||||||||||||||||||| ||||| | |

Tgt 159 + AACCTCGACTTC--GGAAGGGGTGTATTTATTAGATAAAAAACCAATGCCCTT 209

213 cols, 196 ids (92.0%), 4 gaps (1.9%)

Query 213nt >Otu4

Target 213nt >Otu7

Qry 1 + TAGTCATATGCTTGTCTCAAAGATTAAGCCATGCATGTCTAAGTATAAGCAATTTATACAGTGAAACTGCGAATGGCTCA 80

||||||||||||||||||||||||||||||||||||||||||||||||||| ||||||| ||||||||||||||||||||

Tgt 1 + TAGTCATATGCTTGTCTCAAAGATTAAGCCATGCATGTCTAAGTATAAGCACTTTATACTGTGAAACTGCGAATGGCTCA 80

Qry 81 + TTAAATCAGTTATCGTTTATTTGATAGTTCCTTTACTACATGGTATAACTGTGGTAATTCTAGAGCTAATACATGCTTAA 160

|||||||||||||||||||||||||||| |||| ||||||||| ||| ||||||||||||||||||||||||||||| ||

Tgt 81 + TTAAATCAGTTATCGTTTATTTGATAGTACCTT-ACTACATGG-ATACCTGTGGTAATTCTAGAGCTAATACATGCTGAA 158

Qry 161 + AATCTCGACCTCTTGGAAGAGATGTATTTATTAGATAAAAAATCAATGTCTTC 213

|| |||||| || ||||| | |||||||||||||||||||| ||||| | |

Tgt 159 + AACCTCGACTTC--GGAAGGGGTGTATTTATTAGATAAAAAACCAATGCCCTT 209

213 cols, 196 ids (92.0%), 4 gaps (1.9%)

Query 213nt >Otu4

Target 213nt >Otu16

Qry 1 + TAGTCATATGCTTGTCTCAAAGATTAAGCCATGCATGTCTAAGTATAAGCAATTTATACAGTGAAACTGCGAATGGCTCA 80

|||||||||||||||||||||||||||||||||||||||||||||||||||| || ||| ||||||||||||||||||||

Tgt 1 + TAGTCATATGCTTGTCTCAAAGATTAAGCCATGCATGTCTAAGTATAAGCAAATTGTACGGTGAAACTGCGAATGGCTCA 80

Qry 81 + TTAAATCAGTTATCGTTTATTTGATAGTTCCTTTACTACATGGTATAACTGTGGTAATTCTAGAGCTAATACATGCTTAA 160

|||||||||||||||||||||||||||| |||| ||||||||| ||| ||||||||||||||||||||||||||||| ||

Tgt 81 + TTAAATCAGTTATCGTTTATTTGATAGTACCTT-ACTACATGG-ATACCTGTGGTAATTCTAGAGCTAATACATGCTAAA 158

Qry 161 + AATCTCGACCTCTTGGAAGAGATGTATTTATTAGATAAAAAATCAATGTCTTC 213

|| |||||| || ||||| | |||||||||||||||||||| ||||| | |

Tgt 159 + AACCTCGACTTC--GGAAGGGGTGTATTTATTAGATAAAAAACCAATGCCCTT 209

213 cols, 195 ids (91.5%), 4 gaps (1.9%)

Query 213nt >Otu4

Target 213nt >Otu17

Qry 1 + TAGTCATATGCTTGTCTCAAAGATTAAGCCATGCATGTCTAAGTATAAGCAATTTATACAGTGAAACTGCGAATGGCTCA 80

|||||||||||||||||||||||||||||||||||||||||||||||||||| ||||| ||||||||||||||||||||

Tgt 1 + TAGTCATATGCTTGTCTCAAAGATTAAGCCATGCATGTCTAAGTATAAGCAA-CTATACGGTGAAACTGCGAATGGCTCA 79

Qry 81 + TTAAATCAGTTATCGTTTATTTGATAGTTCCTTTACTACATGGTATAACTGTGGTAATTCTAGAGCTAATACATGCTTAA 160

|||||||||||||||||||||||||||| |||| ||||||||| ||||| ||||||||||||||||||||||||||| ||

Tgt 80 + TTAAATCAGTTATCGTTTATTTGATAGTACCTT-ACTACATGG-ATAACCGTGGTAATTCTAGAGCTAATACATGCTAAA 157

Qry 161 + AATCTCGACCTCTTGGAAGAGATGTATTTATTAGATAAAAAATCAATGTCTTC 213

|| | |||| || ||||| | |||||||||||||||||||| ||||| | |

Tgt 158 + AACCCCGACTTC--GGAAGGGGTGTATTTATTAGATAAAAAACCAATGCCCTT 208

213 cols, 194 ids (91.1%), 5 gaps (2.3%)

Query 213nt >Otu4

Target 213nt >Otu10

Qry 1 + TAGTCATATGCTTGTCTCAAAGATTAAGCCATGCATGTCTAAGTATAAGCAATTTATACAGTGAAACTGCGAATGGCTCA 80

|||||||||||||||||||||||||||||||||||||||||||||||||||||| |||| ||||||||||||||||||||

Tgt 1 + TAGTCATATGCTTGTCTCAAAGATTAAGCCATGCATGTCTAAGTATAAGCAATT-ATACCGTGAAACTGCGAATGGCTCA 79

Qry 81 + TTAAATCAGTTATCGTTTATTTGATAGTTCCTTTACTACATGGTATAACTGTGGTAATTCTAGAGCTAATACATGCTTAA 160

|||||||||||||||||||||||||| | || ||||||| ||| ||||| ||||||||||||||||||||||||||| ||

Tgt 80 + TTAAATCAGTTATCGTTTATTTGATAATACC-TTACTACTTGG-ATAACCGTGGTAATTCTAGAGCTAATACATGCTGAA 157

Qry 161 + AATCTCGACCTCTTGGAAGAGATGTATTTATTAGATAAAAAATCAATGTCTTC 213

|||| |||| || ||||| ||||| |||||||||||||||| ||||| | |

Tgt 158 + AATCCCGACTTC--GGAAGGGATGTGTTTATTAGATAAAAAACCAATGCCCTT 208

213 cols, 194 ids (91.1%), 5 gaps (2.3%)

Query 213nt >Otu4

Target 213nt >Otu20

Qry 1 + TAGTCATATGCTTGTCTCAAAGATTAAGCCATGCATGTCTAAGTATAAGCAATTTATACAGTGAAACTGCGAATGGCTCA 80

||||||||||||||||||||||||||||||||||||||||||||||||||||| |||| ||||||||||||||||||||

Tgt 1 + CAGTCATATGCTTGTCTCAAAGATTAAGCCATGCATGTCTAAGTATAAGCAATT-ATACCGTGAAACTGCGAATGGCTCA 79

Qry 81 + TTAAATCAGTTATCGTTTATTTGATAGTTCCTTTACTACATGGTATAACTGTGGTAATTCTAGAGCTAATACATGCTTAA 160

|||||||||||||||||||||||||| | || ||||||| ||| ||||| ||||||||||||||||||||||||||| ||

Tgt 80 + TTAAATCAGTTATCGTTTATTTGATAATACC-TTACTACTTGG-ATAACCGTGGTAATTCTAGAGCTAATACATGCTGAA 157

Qry 161 + AATCTCGACCTCTTGGAAGAGATGTATTTATTAGATAAAAAATCAATGTCTTC 213

|||| |||| || ||||| ||||| |||||||||||||||| ||||| | |

Tgt 158 + AATCCCGACTTC--GGAAGGGATGTGTTTATTAGATAAAAAACCAATGCCCTT 208

213 cols, 193 ids (90.6%), 5 gaps (2.3%)

Query 213nt >Otu4

Target 213nt >Otu6

Qry 1 + TAGTCATATGCTTGTCTCAAAGATTAAGCCATGCATGTCTAAGTATAAGC-AATTTATACAGTGAAACTGCGAATGGCTC 79

|||||||||||||||||||||||||||||||||||||||||||||||| | |||| |||| |||||||||||||||||||

Tgt 1 + TAGTCATATGCTTGTCTCAAAGATTAAGCCATGCATGTCTAAGTATAAACGAATTCATACTGTGAAACTGCGAATGGCTC 80

Qry 80 + ATTAAATCAGTTATCGTTTATTTGATAGTTCCTTTACTACATGGTATAACTGTGGTAATTCTAGAGCTAATACATGCTTA 159

|||||||||||||| ||||||||||| || | || |||||||| ||||||||||||||||||||||||||||||||| |

Tgt 81 + ATTAAATCAGTTATAGTTTATTTGATGGTATC-TTGCTACATGG-ATAACTGTGGTAATTCTAGAGCTAATACATGCTGA 158

Qry 160 + AAATCTCGACCTCTTGGAAGAGATGTATTTATTAGATAAAAAATCAATGTCTTC 213

||| | | || |||||| | |||||||||||||||||||| ||||| ||

Tgt 159 + AAAGCCCCGACTTCTGGAAGGGGTGTATTTATTAGATAAAAAACCAATGGGTTT 212

214 cols, 189 ids (88.3%), 3 gaps (1.4%)

Query 213nt >Otu4

Target 213nt >Otu12

Qry 1 + TAGTCATATGCTTGTCTCAAAGATTAAGCCATGCATGTCTAAGTATAAGCAATTTATACAGTGAAACTGCGAATGGCTCA 80

||||||||||||||||||||||||||||||||||||||||||||||||||||| |||| ||||||||||||||||||||

Tgt 1 + TAGTCATATGCTTGTCTCAAAGATTAAGCCATGCATGTCTAAGTATAAGCAAT-CATACTGTGAAACTGCGAATGGCTCA 79

Qry 81 + TTAAATCAGTTATCGTTTATTTGATAGTTCCTTTACTACATGGTATAACTGTGGTAATTCTAGAGCTAATACATGCTTAA 160

|||||||||||||||||||||||||||| ||||||| | ||||| ||||||||||||||||||||||||||| |

Tgt 80 + TTAAATCAGTTATCGTTTATTTGATAGTACCTTTAC----CTGGATAACCGTGGTAATTCTAGAGCTAATACATGCTAAC 155

Qry 161 + AATCTCGACCTCTTGGAAGAGATGTATTTATTAGATAAAAAATCAATGTCTTC 213

|| | |||| || || || | |||||||||||||||||||| ||||| | |

Tgt 156 + AACCCCGACTTC--GGGAGGGGTGTATTTATTAGATAAAAAACCAATGCCCTT 206

213 cols, 187 ids (87.8%), 7 gaps (3.3%)

Query 213nt >Otu4

Target 213nt >Otu18

Qry 1 + TAGTCATATGCTTGTCTCAAAGATTAAGCCATGCATGTCTAAGTATAAGCAA-TTTATACAGTGAAACTGCGAATGGCTC 79

|||||||||||||||||||||||||||||||||||||||||||||||| ||| ||| ||| |||||||||||||||||||

Tgt 1 + TAGTCATATGCTTGTCTCAAAGATTAAGCCATGCATGTCTAAGTATAAACAACTTTGTACTGTGAAACTGCGAATGGCTC 80

Qry 80 + ATTAAATCAGTTATCGTTTATTTGATAGTTCCTTTACTACATGGTATAACTGTGGTAATTCTAGAGCTAATACATGCTTA 159

|||||||||||||| ||||||||||| |||| || |||||||| |||||||||||||||||||||||||||||||| ||

Tgt 81 + ATTAAATCAGTTATAGTTTATTTGATGGTTC--TTGCTACATGG-ATAACTGTGGTAATTCTAGAGCTAATACATGCGTA 157

Qry 160 + AAATCTCGACCTCTTGGAAGAGATGTATTTATTAGATAAAAAATCAATGTCTTC 213

|||| | || |||||| | ||||||||||||||||||| | || | |

Tgt 158 + TAATCCCCGACTTCTGGAAGGGGTGTATTTATTAGATAAAAA--CCATCCCCTT 209

214 cols, 187 ids (87.4%), 6 gaps (2.8%)

Query 213nt >Otu4

Target 213nt >Otu15

Qry 1 + TAGTCATATGCTTGTCTCAAAGATTAAGCCATGCATGTCTAAGTATAAGCAATTTATACAGTGAAACTGCGAATGGCTCA 80

|||||||||||||||||||||||||||||||||||||||||||||||| || |||||| ||||||||||||||||||||

Tgt 1 + TAGTCATATGCTTGTCTCAAAGATTAAGCCATGCATGTCTAAGTATAAACACGTTATACTGTGAAACTGCGAATGGCTCA 80

Qry 81 + TTAAATCAGTTATCGTTTATTTGATAGTTCCTTTACTACATGGTATAACTGTGGTAATTCTAGAGCTAATACATGCTTAA 160

||||||||||||| ||||||||||| ||| ||| |||||||| |||||||||||||||||||||||||||||||| ||

Tgt 81 + TTAAATCAGTTATAGTTTATTTGATGGTT--TTTGCTACATGG-ATAACTGTGGTAATTCTAGAGCTAATACATGCGTAT 157

Qry 161 + AATCTCGACCTCTTGGAAGAGATGTATTTATTAGATAAAAAATCAATGTCTTC 213

|| | | || |||||| | ||||||||||||||||||| | | ||

Tgt 158 + AAGCCCCGACTTCTGGAAGGGGTGTATTTATTAGATAAAAATCAACACTGTTG 210

213 cols, 184 ids (86.4%), 3 gaps (1.4%)

Query >Otu6

%Id TLen Target

93% 213 Otu7

92% 213 Otu8

92% 213 Otu9

92% 213 Otu18

92% 213 Otu15

92% 213 Otu16

92% 213 Otu17

91% 213 Otu12

90% 213 Otu10

90% 213 Otu20

89% 213 Otu14

Query 213nt >Otu6

Target 213nt >Otu7

Qry 1 + TAGTCATATGCTTGTCTCAAAGATTAAGCCATGCATGTCTAAGTATAAACGAATTCATACTGTGAAACTGCGAATGGCTC 80

|||||||||||||||||||||||||||||||||||||||||||||||| | | || ||||||||||||||||||||||||

Tgt 1 + TAGTCATATGCTTGTCTCAAAGATTAAGCCATGCATGTCTAAGTATAAGC-ACTTTATACTGTGAAACTGCGAATGGCTC 79

Qry 81 + ATTAAATCAGTTATAGTTTATTTGATGGTATCTTGCTACATGGATAACTGTGGTAATTCTAGAGCTAATACATGCTGAAA 160

|||||||||||||| ||||||||||| ||| ||| ||||||||||| |||||||||||||||||||||||||||||||||

Tgt 80 + ATTAAATCAGTTATCGTTTATTTGATAGTACCTTACTACATGGATACCTGTGGTAATTCTAGAGCTAATACATGCTGAAA 159

Qry 161 + AGCCCCGACTTCTGGAAGGGGTGTATTTATTAGATAAAAAACCAATGGGTTTC 213

| || ||||||| |||||||||||||||||||||||||||||||||| |||

Tgt 160 + A-CCTCGACTTC-GGAAGGGGTGTATTTATTAGATAAAAAACCAATGCCCTTC 210

213 cols, 198 ids (93.0%), 3 gaps (1.4%)

Query 213nt >Otu6

Target 213nt >Otu8

Qry 1 + TAGTCATATGCTTGTCTCAAAGATTAAGCCATGCATGTCTAAGTATAAACGAATTCATACTGTGAAACTGCGAATGGCTC 80

|||||||||||||||||||||||||||||||||||||||||||||||| | |||| |||| |||||||||||||||||||

Tgt 1 + TAGTCATATGCTTGTCTCAAAGATTAAGCCATGCATGTCTAAGTATAAGC-AATTTATACGGTGAAACTGCGAATGGCTC 79

Qry 81 + ATTAAATCAGTTATAGTTTATTTGATGGTATCTTGCTACATGGATAACTGTGGTAATTCTAGAGCTAATACATGCTGAAA 160

|||||||||||||| ||||||||||| ||| ||| ||||||||||| ||||||||||||||||||||||||||||| |||

Tgt 80 + ATTAAATCAGTTATCGTTTATTTGATAGTACCTTACTACATGGATACCTGTGGTAATTCTAGAGCTAATACATGCTAAAA 159

Qry 161 + AGCCCCGACTTCTGGAAGGGGTGTATTTATTAGATAAAAAACCAATGGGTTTC 213

| || ||||||| |||||||||||||||||||||||||||||||||| |||

Tgt 160 + A-CCTCGACTTC-GGAAGGGGTGTATTTATTAGATAAAAAACCAATGCCCTTC 210

213 cols, 197 ids (92.5%), 3 gaps (1.4%)

Query 213nt >Otu6

Target 213nt >Otu9

Qry 1 + TAGTCATATGCTTGTCTCAAAGATTAAGCCATGCATGTCTAAGTATAAACGAATTCATACTGTGAAACTGCGAATGGCTC 80

|||||||||||||||||||||||||||||||||||||||||||||||| | | || ||||||||||||||||||||||||

Tgt 1 + TAGTCATATGCTTGTCTCAAAGATTAAGCCATGCATGTCTAAGTATAAGC-ACTTTATACTGTGAAACTGCGAATGGCTC 79

Qry 81 + ATTAAATCAGTTATAGTTTATTTGATGGTATCTTGCTACATGGATAACTGTGGTAATTCTAGAGCTAATACATGCTGAAA 160

|||||||||||||| ||||||||||| ||| ||| ||||||||||| ||||||||||||||||||||||||||||| |||

Tgt 80 + ATTAAATCAGTTATCGTTTATTTGATAGTACCTTACTACATGGATACCTGTGGTAATTCTAGAGCTAATACATGCTAAAA 159

Qry 161 + AGCCCCGACTTCTGGAAGGGGTGTATTTATTAGATAAAAAACCAATGGGTTTC 213

| || ||||||| |||||||||||||||||||||||||||||||||| |||

Tgt 160 + A-CCTCGACTTC-GGAAGGGGTGTATTTATTAGATAAAAAACCAATGCCCTTC 210

213 cols, 197 ids (92.5%), 3 gaps (1.4%)

Query 213nt >Otu6

Target 213nt >Otu18

Qry 1 + TAGTCATATGCTTGTCTCAAAGATTAAGCCATGCATGTCTAAGTATAAACGAATTCATACTGTGAAACTGCGAATGGCTC 80

|||||||||||||||||||||||||||||||||||||||||||||||||| | || |||||||||||||||||||||||

Tgt 1 + TAGTCATATGCTTGTCTCAAAGATTAAGCCATGCATGTCTAAGTATAAACAACTTTGTACTGTGAAACTGCGAATGGCTC 80

Qry 81 + ATTAAATCAGTTATAGTTTATTTGATGGTATCTTGCTACATGGATAACTGTGGTAATTCTAGAGCTAATACATGCTGAAA 160

||||||||||||||||||||||||||||| ||||||||||||||||||||||||||||||||||||||||||||| | |

Tgt 81 + ATTAAATCAGTTATAGTTTATTTGATGGT-TCTTGCTACATGGATAACTGTGGTAATTCTAGAGCTAATACATGCGTATA 159

Qry 161 + AGCCCCGACTTCTGGAAGGGGTGTATTTATTAGATAAAAA-----ACCAATGGG 209

| |||||||||||||||||||||||||||||||||||||| || ||||

Tgt 160 + ATCCCCGACTTCTGGAAGGGGTGTATTTATTAGATAAAAACCATCCCCTTTGGG 213

214 cols, 197 ids (92.1%), 6 gaps (2.8%)

Query 213nt >Otu6

Target 213nt >Otu15

Qry 1 + TAGTCATATGCTTGTCTCAAAGATTAAGCCATGCATGTCTAAGTATAAACGAATTCATACTGTGAAACTGCGAATGGCTC 80

|||||||||||||||||||||||||||||||||||||||||||||||||| | | ||||||||||||||||||||||||

Tgt 1 + TAGTCATATGCTTGTCTCAAAGATTAAGCCATGCATGTCTAAGTATAAAC-ACGTTATACTGTGAAACTGCGAATGGCTC 79

Qry 81 + ATTAAATCAGTTATAGTTTATTTGATGGTATCTTGCTACATGGATAACTGTGGTAATTCTAGAGCTAATACATGCTGAAA 160

||||||||||||||||||||||||||||| | ||||||||||||||||||||||||||||||||||||||||||| | |

Tgt 80 + ATTAAATCAGTTATAGTTTATTTGATGGT-TTTTGCTACATGGATAACTGTGGTAATTCTAGAGCTAATACATGCGTATA 158

Qry 161 + AGCCCCGACTTCTGGAAGGGGTGTATTTATTAGATAAAAAACCAATGGGTTTC 213

|||||||||||||||||||||||||||||||||||||||| | | |||

Tgt 159 + AGCCCCGACTTCTGGAAGGGGTGTATTTATTAGATAAAAATCAACACTGTTGG 211

213 cols, 196 ids (92.0%), 2 gaps (0.9%)

Query 213nt >Otu6

Target 213nt >Otu16

Qry 1 + TAGTCATATGCTTGTCTCAAAGATTAAGCCATGCATGTCTAAGTATAAACGAATTCATACTGTGAAACTGCGAATGGCTC 80

|||||||||||||||||||||||||||||||||||||||||||||||| | |||| ||| |||||||||||||||||||

Tgt 1 + TAGTCATATGCTTGTCTCAAAGATTAAGCCATGCATGTCTAAGTATAAGCAAATT-GTACGGTGAAACTGCGAATGGCTC 79

Qry 81 + ATTAAATCAGTTATAGTTTATTTGATGGTATCTTGCTACATGGATAACTGTGGTAATTCTAGAGCTAATACATGCTGAAA 160

|||||||||||||| ||||||||||| ||| ||| ||||||||||| ||||||||||||||||||||||||||||| |||

Tgt 80 + ATTAAATCAGTTATCGTTTATTTGATAGTACCTTACTACATGGATACCTGTGGTAATTCTAGAGCTAATACATGCTAAAA 159

Qry 161 + AGCCCCGACTTCTGGAAGGGGTGTATTTATTAGATAAAAAACCAATGGGTTTC 213

| || ||||||| |||||||||||||||||||||||||||||||||| |||

Tgt 160 + A-CCTCGACTTC-GGAAGGGGTGTATTTATTAGATAAAAAACCAATGCCCTTC 210

213 cols, 196 ids (92.0%), 3 gaps (1.4%)

Query 213nt >Otu6

Target 213nt >Otu17

Qry 1 + TAGTCATATGCTTGTCTCAAAGATTAAGCCATGCATGTCTAAGTATAAACGAATTCATACTGTGAAACTGCGAATGGCTC 80

|||||||||||||||||||||||||||||||||||||||||||||||| | | | |||| |||||||||||||||||||

Tgt 1 + TAGTCATATGCTTGTCTCAAAGATTAAGCCATGCATGTCTAAGTATAAGCAACT--ATACGGTGAAACTGCGAATGGCTC 78

Qry 81 + ATTAAATCAGTTATAGTTTATTTGATGGTATCTTGCTACATGGATAACTGTGGTAATTCTAGAGCTAATACATGCTGAAA 160

|||||||||||||| ||||||||||| ||| ||| ||||||||||||| ||||||||||||||||||||||||||| |||

Tgt 79 + ATTAAATCAGTTATCGTTTATTTGATAGTACCTTACTACATGGATAACCGTGGTAATTCTAGAGCTAATACATGCTAAAA 158

Qry 161 + AGCCCCGACTTCTGGAAGGGGTGTATTTATTAGATAAAAAACCAATGGGTTTC 213

| ||| |||||| |||||||||||||||||||||||||||||||||| |||

Tgt 159 + ACCCC-GACTTC-GGAAGGGGTGTATTTATTAGATAAAAAACCAATGCCCTTC 209

213 cols, 195 ids (91.5%), 4 gaps (1.9%)

Query 213nt >Otu6

Target 213nt >Otu12

Qry 1 + TAGTCATATGCTTGTCTCAAAGATTAAGCCATGCATGTCTAAGTATAAACGAATTCATACTGTGAAACTGCGAATGGCTC 80

|||||||||||||||||||||||||||||||||||||||||||||||| | | ||||||||||||||||||||||||||

Tgt 1 + TAGTCATATGCTTGTCTCAAAGATTAAGCCATGCATGTCTAAGTATAA--GCAATCATACTGTGAAACTGCGAATGGCTC 78

Qry 81 + ATTAAATCAGTTATAGTTTATTTGATGGTATCTTGCTACATGGATAACTGTGGTAATTCTAGAGCTAATACATGCTGAAA 160

|||||||||||||| ||||||||||| ||| ||| ||| |||||||| ||||||||||||||||||||||||||| ||

Tgt 79 + ATTAAATCAGTTATCGTTTATTTGATAGTACCTT--TACCTGGATAACCGTGGTAATTCTAGAGCTAATACATGCT-AAC 155

Qry 161 + AGCCCCGACTTCTGGAAGGGGTGTATTTATTAGATAAAAAACCAATGGGTTTC 213

| |||||||||| || ||||||||||||||||||||||||||||||| |||

Tgt 156 + AACCCCGACTTC-GGGAGGGGTGTATTTATTAGATAAAAAACCAATGCCCTTC 207

213 cols, 194 ids (91.1%), 6 gaps (2.8%)

Query 213nt >Otu6

Target 213nt >Otu10

Qry 1 + TAGTCATATGCTTGTCTCAAAGATTAAGCCATGCATGTCTAAGTATAAACGAATTCATACTGTGAAACTGCGAATGGCTC 80

|||||||||||||||||||||||||||||||||||||||||||||||| | | | |||| |||||||||||||||||||

Tgt 1 + TAGTCATATGCTTGTCTCAAAGATTAAGCCATGCATGTCTAAGTATAA--GCAATTATACCGTGAAACTGCGAATGGCTC 78

Qry 81 + ATTAAATCAGTTATAGTTTATTTGATGGTATCTTGCTACATGGATAACTGTGGTAATTCTAGAGCTAATACATGCTGAAA 160

|||||||||||||| ||||||||||| || ||| |||| |||||||| |||||||||||||||||||||||||||||||

Tgt 79 + ATTAAATCAGTTATCGTTTATTTGATAATACCTTACTACTTGGATAACCGTGGTAATTCTAGAGCTAATACATGCTGAAA 158

Qry 161 + AGCCCCGACTTCTGGAAGGGGTGTATTTATTAGATAAAAAACCAATGGGTTTC 213

| ||| |||||| ||||||| ||| |||||||||||||||||||||| |||

Tgt 159 + ATCCC-GACTTC-GGAAGGGATGTGTTTATTAGATAAAAAACCAATGCCCTTC 209

213 cols, 192 ids (90.1%), 4 gaps (1.9%)

Query 213nt >Otu6

Target 213nt >Otu20

Qry 1 + TAGTCATATGCTTGTCTCAAAGATTAAGCCATGCATGTCTAAGTATAAACGAATTCATACTGTGAAACTGCGAATGGCTC 80

||||||||||||||||||||||||||||||||||||||||||||||| | | | |||| |||||||||||||||||||

Tgt 1 + CAGTCATATGCTTGTCTCAAAGATTAAGCCATGCATGTCTAAGTATAA--GCAATTATACCGTGAAACTGCGAATGGCTC 78

Qry 81 + ATTAAATCAGTTATAGTTTATTTGATGGTATCTTGCTACATGGATAACTGTGGTAATTCTAGAGCTAATACATGCTGAAA 160

|||||||||||||| ||||||||||| || ||| |||| |||||||| |||||||||||||||||||||||||||||||

Tgt 79 + ATTAAATCAGTTATCGTTTATTTGATAATACCTTACTACTTGGATAACCGTGGTAATTCTAGAGCTAATACATGCTGAAA 158

Qry 161 + AGCCCCGACTTCTGGAAGGGGTGTATTTATTAGATAAAAAACCAATGGGTTTC 213

| ||| |||||| ||||||| ||| |||||||||||||||||||||| |||

Tgt 159 + ATCCC-GACTTC-GGAAGGGATGTGTTTATTAGATAAAAAACCAATGCCCTTC 209

213 cols, 191 ids (89.7%), 4 gaps (1.9%)

Query 213nt >Otu6

Target 213nt >Otu14

Qry 1 + TAGTCATATGCTTGTCTCAAAGATTAAGCCATGCATGTCTAAGTATAAACGAATTCATACTGTGAAACTGCGAATGGCTC 80

|||||||||||||||||||||||||||||||||||||||||||||||| | |||| |||| |||||||||||||||||||

Tgt 1 + TAGTCATATGCTTGTCTCAAAGATTAAGCCATGCATGTCTAAGTATAAGC-AATTTATACAGTGAAACTGCGAATGGCTC 79

Qry 81 + ATTAAATCAGTTATAGTTTATTTGATGGTATCTTGCTACATGGATAACTGTGGTAATTCTAGAGCTAATACATGCTGAAA 160

|||||||||||||| ||||||||||| ||| ||| |||| |||||||| ||||||||||||||||||||||||||| |||

Tgt 80 + ATTAAATCAGTTATCGTTTATTTGATAGTACCTTACTACTTGGATAACCGTGGTAATTCTAGAGCTAATACATGCTTAAA 159

Qry 161 + AGCCCCGACTTCTGGAAGGGGTGTATTTATTAGATAAAAAACCAATGGGTTTC 213

| ||| | |||||||| |||||||||||||||||||| ||||| ||

Tgt 160 + ATCCCGACTGTTTGGAAGGGATGTATTTATTAGATAAAAAATCAATGTCTTCG 212

213 cols, 189 ids (88.7%), 1 gaps (0.5%)

Query >Otu7

%Id TLen Target

100% 213 Otu9

99% 213 Otu8

98% 213 Otu16

96% 213 Otu17

95% 213 Otu10

94% 213 Otu12

94% 213 Otu20

92% 213 Otu14

91% 213 Otu18

89% 213 Otu15

Query 213nt >Otu7

Target 213nt >Otu9

Qry 1 + TAGTCATATGCTTGTCTCAAAGATTAAGCCATGCATGTCTAAGTATAAGCACTTTATACTGTGAAACTGCGAATGGCTCA 80

||||||||||||||||||||||||||||||||||||||||||||||||||||||||||||||||||||||||||||||||

Tgt 1 + TAGTCATATGCTTGTCTCAAAGATTAAGCCATGCATGTCTAAGTATAAGCACTTTATACTGTGAAACTGCGAATGGCTCA 80

Qry 81 + TTAAATCAGTTATCGTTTATTTGATAGTACCTTACTACATGGATACCTGTGGTAATTCTAGAGCTAATACATGCTGAAAA 160

||||||||||||||||||||||||||||||||||||||||||||||||||||||||||||||||||||||||||| ||||

Tgt 81 + TTAAATCAGTTATCGTTTATTTGATAGTACCTTACTACATGGATACCTGTGGTAATTCTAGAGCTAATACATGCTAAAAA 160

Qry 161 + CCTCGACTTCGGAAGGGGTGTATTTATTAGATAAAAAACCAATGCCCTTCGGG 213

|||||||||||||||||||||||||||||||||||||||||||||||||||||

Tgt 161 + CCTCGACTTCGGAAGGGGTGTATTTATTAGATAAAAAACCAATGCCCTTCGGG 213

213 cols, 212 ids (99.5%), 0 gaps (0.0%)

Query 213nt >Otu7

Target 213nt >Otu8

Qry 1 + TAGTCATATGCTTGTCTCAAAGATTAAGCCATGCATGTCTAAGTATAAGCACTTTATACTGTGAAACTGCGAATGGCTCA 80

||||||||||||||||||||||||||||||||||||||||||||||||||| ||||||| ||||||||||||||||||||

Tgt 1 + TAGTCATATGCTTGTCTCAAAGATTAAGCCATGCATGTCTAAGTATAAGCAATTTATACGGTGAAACTGCGAATGGCTCA 80

Qry 81 + TTAAATCAGTTATCGTTTATTTGATAGTACCTTACTACATGGATACCTGTGGTAATTCTAGAGCTAATACATGCTGAAAA 160

||||||||||||||||||||||||||||||||||||||||||||||||||||||||||||||||||||||||||| ||||

Tgt 81 + TTAAATCAGTTATCGTTTATTTGATAGTACCTTACTACATGGATACCTGTGGTAATTCTAGAGCTAATACATGCTAAAAA 160

Qry 161 + CCTCGACTTCGGAAGGGGTGTATTTATTAGATAAAAAACCAATGCCCTTCGGG 213

|||||||||||||||||||||||||||||||||||||||||||||||||||||

Tgt 161 + CCTCGACTTCGGAAGGGGTGTATTTATTAGATAAAAAACCAATGCCCTTCGGG 213

213 cols, 210 ids (98.6%), 0 gaps (0.0%)

Query 213nt >Otu7

Target 213nt >Otu16

Qry 1 + TAGTCATATGCTTGTCTCAAAGATTAAGCCATGCATGTCTAAGTATAAGCACTTTATACTGTGAAACTGCGAATGGCTCA 80

||||||||||||||||||||||||||||||||||||||||||||||||||| || ||| ||||||||||||||||||||

Tgt 1 + TAGTCATATGCTTGTCTCAAAGATTAAGCCATGCATGTCTAAGTATAAGCAAATTGTACGGTGAAACTGCGAATGGCTCA 80

Qry 81 + TTAAATCAGTTATCGTTTATTTGATAGTACCTTACTACATGGATACCTGTGGTAATTCTAGAGCTAATACATGCTGAAAA 160

||||||||||||||||||||||||||||||||||||||||||||||||||||||||||||||||||||||||||| ||||

Tgt 81 + TTAAATCAGTTATCGTTTATTTGATAGTACCTTACTACATGGATACCTGTGGTAATTCTAGAGCTAATACATGCTAAAAA 160

Qry 161 + CCTCGACTTCGGAAGGGGTGTATTTATTAGATAAAAAACCAATGCCCTTCGGG 213

|||||||||||||||||||||||||||||||||||||||||||||||||||||

Tgt 161 + CCTCGACTTCGGAAGGGGTGTATTTATTAGATAAAAAACCAATGCCCTTCGGG 213

213 cols, 208 ids (97.7%), 0 gaps (0.0%)

Query 213nt >Otu7

Target 213nt >Otu17

Qry 1 + TAGTCATATGCTTGTCTCAAAGATTAAGCCATGCATGTCTAAGTATAAGCACTTTATACTGTGAAACTGCGAATGGCTCA 80

||||||||||||||||||||||||||||||||||||||||||||||||||| ||||| ||||||||||||||||||||

Tgt 1 + TAGTCATATGCTTGTCTCAAAGATTAAGCCATGCATGTCTAAGTATAAGCA-ACTATACGGTGAAACTGCGAATGGCTCA 79

Qry 81 + TTAAATCAGTTATCGTTTATTTGATAGTACCTTACTACATGGATACCTGTGGTAATTCTAGAGCTAATACATGCTGAAAA 160

||||||||||||||||||||||||||||||||||||||||||||| | ||||||||||||||||||||||||||| ||||

Tgt 80 + TTAAATCAGTTATCGTTTATTTGATAGTACCTTACTACATGGATAACCGTGGTAATTCTAGAGCTAATACATGCTAAAAA 159

Qry 161 + CCTCGACTTCGGAAGGGGTGTATTTATTAGATAAAAAACCAATGCCCTTCGGG 213

|| ||||||||||||||||||||||||||||||||||||||||||||||||||

Tgt 160 + CCCCGACTTCGGAAGGGGTGTATTTATTAGATAAAAAACCAATGCCCTTCGGG 212

213 cols, 205 ids (96.2%), 1 gaps (0.5%)

Query 213nt >Otu7

Target 213nt >Otu10

Qry 1 + TAGTCATATGCTTGTCTCAAAGATTAAGCCATGCATGTCTAAGTATAAGCACTTTATACTGTGAAACTGCGAATGGCTCA 80

||||||||||||||||||||||||||||||||||||||||||||||||||| |||||| ||||||||||||||||||||

Tgt 1 + TAGTCATATGCTTGTCTCAAAGATTAAGCCATGCATGTCTAAGTATAAGCA-ATTATACCGTGAAACTGCGAATGGCTCA 79

Qry 81 + TTAAATCAGTTATCGTTTATTTGATAGTACCTTACTACATGGATACCTGTGGTAATTCTAGAGCTAATACATGCTGAAAA 160

|||||||||||||||||||||||||| ||||||||||| |||||| | ||||||||||||||||||||||||||||||||

Tgt 80 + TTAAATCAGTTATCGTTTATTTGATAATACCTTACTACTTGGATAACCGTGGTAATTCTAGAGCTAATACATGCTGAAAA 159

Qry 161 + CCTCGACTTCGGAAGGGGTGTATTTATTAGATAAAAAACCAATGCCCTTCGGG 213

| |||||||||||||| ||| |||||||||||||||||||||||||||||||

Tgt 160 + TCCCGACTTCGGAAGGGATGTGTTTATTAGATAAAAAACCAATGCCCTTCGGG 212

213 cols, 202 ids (94.8%), 1 gaps (0.5%)

Query 213nt >Otu7

Target 213nt >Otu12

Qry 1 + TAGTCATATGCTTGTCTCAAAGATTAAGCCATGCATGTCTAAGTATAAGCACTTTATACTGTGAAACTGCGAATGGCTCA 80

||||||||||||||||||||||||||||||||||||||||||||||||||| | |||||||||||||||||||||||||

Tgt 1 + TAGTCATATGCTTGTCTCAAAGATTAAGCCATGCATGTCTAAGTATAAGCA-ATCATACTGTGAAACTGCGAATGGCTCA 79

Qry 81 + TTAAATCAGTTATCGTTTATTTGATAGTACCTTACTACATGGATACCTGTGGTAATTCTAGAGCTAATACATGCTGAAAA 160

||||||||||||||||||||||||||||||||| ||| |||||| | ||||||||||||||||||||||||||| | ||

Tgt 80 + TTAAATCAGTTATCGTTTATTTGATAGTACCTT--TACCTGGATAACCGTGGTAATTCTAGAGCTAATACATGCTAACAA 157

Qry 161 + CCTCGACTTCGGAAGGGGTGTATTTATTAGATAAAAAACCAATGCCCTTCGGG 213

|| ||||||||| ||||||||||||||||||||||||||||||||||||||||

Tgt 158 + CCCCGACTTCGGGAGGGGTGTATTTATTAGATAAAAAACCAATGCCCTTCGGG 210

213 cols, 201 ids (94.4%), 3 gaps (1.4%)

Query 213nt >Otu7

Target 213nt >Otu20

Qry 1 + TAGTCATATGCTTGTCTCAAAGATTAAGCCATGCATGTCTAAGTATAAGCACTTTATACTGTGAAACTGCGAATGGCTCA 80

|||||||||||||||||||||||||||||||||||||||||||||||||| |||||| ||||||||||||||||||||

Tgt 1 + CAGTCATATGCTTGTCTCAAAGATTAAGCCATGCATGTCTAAGTATAAGCA-ATTATACCGTGAAACTGCGAATGGCTCA 79

Qry 81 + TTAAATCAGTTATCGTTTATTTGATAGTACCTTACTACATGGATACCTGTGGTAATTCTAGAGCTAATACATGCTGAAAA 160

|||||||||||||||||||||||||| ||||||||||| |||||| | ||||||||||||||||||||||||||||||||

Tgt 80 + TTAAATCAGTTATCGTTTATTTGATAATACCTTACTACTTGGATAACCGTGGTAATTCTAGAGCTAATACATGCTGAAAA 159

Qry 161 + CCTCGACTTCGGAAGGGGTGTATTTATTAGATAAAAAACCAATGCCCTTCGGG 213

| |||||||||||||| ||| |||||||||||||||||||||||||||||||

Tgt 160 + TCCCGACTTCGGAAGGGATGTGTTTATTAGATAAAAAACCAATGCCCTTCGGG 212

213 cols, 201 ids (94.4%), 1 gaps (0.5%)

Query 213nt >Otu7

Target 213nt >Otu14

Qry 1 + TAGTCATATGCTTGTCTCAAAGATTAAGCCATGCATGTCTAAGTATAAGCACTTTATACTGTGAAACTGCGAATGGCTCA 80

||||||||||||||||||||||||||||||||||||||||||||||||||| ||||||| ||||||||||||||||||||

Tgt 1 + TAGTCATATGCTTGTCTCAAAGATTAAGCCATGCATGTCTAAGTATAAGCAATTTATACAGTGAAACTGCGAATGGCTCA 80

Qry 81 + TTAAATCAGTTATCGTTTATTTGATAGTACCTTACTACATGGATACCTGTGGTAATTCTAGAGCTAATACATGCTGAAAA 160

|||||||||||||||||||||||||||||||||||||| |||||| | ||||||||||||||||||||||||||| ||||

Tgt 81 + TTAAATCAGTTATCGTTTATTTGATAGTACCTTACTACTTGGATAACCGTGGTAATTCTAGAGCTAATACATGCTTAAAA 160

Qry 161 + CCTCGACT--TCGGAAGGGGTGTATTTATTAGATAAAAAACCAATGCCCTTCG 211

| ||||| | ||||||| |||||||||||||||||||| ||||| | | |

Tgt 161 + TCCCGACTGTTTGGAAGGGATGTATTTATTAGATAAAAAATCAATGTCTTCGG 213

213 cols, 196 ids (92.0%), 2 gaps (0.9%)

Query 213nt >Otu7

Target 213nt >Otu18

Qry 1 + TAGTCATATGCTTGTCTCAAAGATTAAGCCATGCATGTCTAAGTATAAGC-ACTTTATACTGTGAAACTGCGAATGGCTC 79

|||||||||||||||||||||||||||||||||||||||||||||||| | ||||| |||||||||||||||||||||||

Tgt 1 + TAGTCATATGCTTGTCTCAAAGATTAAGCCATGCATGTCTAAGTATAAACAACTTTGTACTGTGAAACTGCGAATGGCTC 80

Qry 80 + ATTAAATCAGTTATCGTTTATTTGATAGTACCTTACTACATGGATACCTGTGGTAATTCTAGAGCTAATACATGC-TGAA 158

|||||||||||||| ||||||||||| || ||| ||||||||||| |||||||||||||||||||||||||||| | |

Tgt 81 + ATTAAATCAGTTATAGTTTATTTGATGGT-TCTTGCTACATGGATAACTGTGGTAATTCTAGAGCTAATACATGCGTATA 159

Qry 159 + AACCTCGACTTC-GGAAGGGGTGTATTTATTAGATAAAAAACCAATGCCCTTCGGG 213

| || ||||||| ||||||||||||||||||||||||||| | || ||||| |||

Tgt 160 + ATCCCCGACTTCTGGAAGGGGTGTATTTATTAGATAAAAA--CCATCCCCTTTGGG 213

216 cols, 196 ids (90.7%), 6 gaps (2.8%)

Query 213nt >Otu7

Target 213nt >Otu15

Qry 1 + TAGTCATATGCTTGTCTCAAAGATTAAGCCATGCATGTCTAAGTATAAGCACTTTATACTGTGAAACTGCGAATGGCTCA 80

|||||||||||||||||||||||||||||||||||||||||||||||| ||| |||||||||||||||||||||||||||

Tgt 1 + TAGTCATATGCTTGTCTCAAAGATTAAGCCATGCATGTCTAAGTATAAACACGTTATACTGTGAAACTGCGAATGGCTCA 80

Qry 81 + TTAAATCAGTTATCGTTTATTTGATAGTACCTTACTACATGGATACCTGTGGTAATTCTAGAGCTAATACATGC-TGAAA 159

||||||||||||| ||||||||||| || || ||||||||||| |||||||||||||||||||||||||||| | ||

Tgt 81 + TTAAATCAGTTATAGTTTATTTGATGGT-TTTTGCTACATGGATAACTGTGGTAATTCTAGAGCTAATACATGCGTATAA 159

Qry 160 + ACCTCGACTTC-GGAAGGGGTGTATTTATTAGATAAAAAACCAATGCCCTTCGGG 213

|| ||||||| ||||||||||||||||||||||||||| ||| | || | |

Tgt 160 + GCCCCGACTTCTGGAAGGGGTGTATTTATTAGATAAAAA-TCAACACTGTTGGTG 213

215 cols, 192 ids (89.3%), 4 gaps (1.9%)

Query >Otu16

%Id TLen Target

99% 213 Otu8

98% 213 Otu9

97% 213 Otu17

94% 213 Otu12

94% 213 Otu10

94% 213 Otu20

92% 213 Otu14

91% 213 Otu18

88% 213 Otu15

Query 213nt >Otu16

Target 213nt >Otu8

Qry 1 + TAGTCATATGCTTGTCTCAAAGATTAAGCCATGCATGTCTAAGTATAAGCAAATTGTACGGTGAAACTGCGAATGGCTCA 80

|||||||||||||||||||||||||||||||||||||||||||||||||||| || ||||||||||||||||||||||||

Tgt 1 + TAGTCATATGCTTGTCTCAAAGATTAAGCCATGCATGTCTAAGTATAAGCAATTTATACGGTGAAACTGCGAATGGCTCA 80

Qry 81 + TTAAATCAGTTATCGTTTATTTGATAGTACCTTACTACATGGATACCTGTGGTAATTCTAGAGCTAATACATGCTAAAAA 160

||||||||||||||||||||||||||||||||||||||||||||||||||||||||||||||||||||||||||||||||

Tgt 81 + TTAAATCAGTTATCGTTTATTTGATAGTACCTTACTACATGGATACCTGTGGTAATTCTAGAGCTAATACATGCTAAAAA 160

Qry 161 + CCTCGACTTCGGAAGGGGTGTATTTATTAGATAAAAAACCAATGCCCTTCGGG 213

|||||||||||||||||||||||||||||||||||||||||||||||||||||

Tgt 161 + CCTCGACTTCGGAAGGGGTGTATTTATTAGATAAAAAACCAATGCCCTTCGGG 213

213 cols, 211 ids (99.1%), 0 gaps (0.0%)

Query 213nt >Otu16

Target 213nt >Otu9

Qry 1 + TAGTCATATGCTTGTCTCAAAGATTAAGCCATGCATGTCTAAGTATAAGCAAATTGTACGGTGAAACTGCGAATGGCTCA 80

||||||||||||||||||||||||||||||||||||||||||||||||||| || ||| ||||||||||||||||||||

Tgt 1 + TAGTCATATGCTTGTCTCAAAGATTAAGCCATGCATGTCTAAGTATAAGCACTTTATACTGTGAAACTGCGAATGGCTCA 80

Qry 81 + TTAAATCAGTTATCGTTTATTTGATAGTACCTTACTACATGGATACCTGTGGTAATTCTAGAGCTAATACATGCTAAAAA 160

||||||||||||||||||||||||||||||||||||||||||||||||||||||||||||||||||||||||||||||||

Tgt 81 + TTAAATCAGTTATCGTTTATTTGATAGTACCTTACTACATGGATACCTGTGGTAATTCTAGAGCTAATACATGCTAAAAA 160

Qry 161 + CCTCGACTTCGGAAGGGGTGTATTTATTAGATAAAAAACCAATGCCCTTCGGG 213

|||||||||||||||||||||||||||||||||||||||||||||||||||||

Tgt 161 + CCTCGACTTCGGAAGGGGTGTATTTATTAGATAAAAAACCAATGCCCTTCGGG 213

213 cols, 209 ids (98.1%), 0 gaps (0.0%)

Query 213nt >Otu16

Target 213nt >Otu17

Qry 1 + TAGTCATATGCTTGTCTCAAAGATTAAGCCATGCATGTCTAAGTATAAGCAAATTGTACGGTGAAACTGCGAATGGCTCA 80

|||||||||||||||||||||||||||||||||||||||||||||||||||| | ||||||||||||||||||||||||

Tgt 1 + TAGTCATATGCTTGTCTCAAAGATTAAGCCATGCATGTCTAAGTATAAGCAA-CTATACGGTGAAACTGCGAATGGCTCA 79

Qry 81 + TTAAATCAGTTATCGTTTATTTGATAGTACCTTACTACATGGATACCTGTGGTAATTCTAGAGCTAATACATGCTAAAAA 160

||||||||||||||||||||||||||||||||||||||||||||| | ||||||||||||||||||||||||||||||||

Tgt 80 + TTAAATCAGTTATCGTTTATTTGATAGTACCTTACTACATGGATAACCGTGGTAATTCTAGAGCTAATACATGCTAAAAA 159

Qry 161 + CCTCGACTTCGGAAGGGGTGTATTTATTAGATAAAAAACCAATGCCCTTCGGG 213

|| ||||||||||||||||||||||||||||||||||||||||||||||||||

Tgt 160 + CCCCGACTTCGGAAGGGGTGTATTTATTAGATAAAAAACCAATGCCCTTCGGG 212

213 cols, 207 ids (97.2%), 1 gaps (0.5%)

Query 213nt >Otu16

Target 213nt >Otu12

Qry 1 + TAGTCATATGCTTGTCTCAAAGATTAAGCCATGCATGTCTAAGTATAAGCAAATTGTACGGTGAAACTGCGAATGGCTCA 80

|||||||||||||||||||||||||||||||||||||||||||||||||||| | ||| ||||||||||||||||||||

Tgt 1 + TAGTCATATGCTTGTCTCAAAGATTAAGCCATGCATGTCTAAGTATAAGCAA-TCATACTGTGAAACTGCGAATGGCTCA 79

Qry 81 + TTAAATCAGTTATCGTTTATTTGATAGTACCTTACTACATGGATACCTGTGGTAATTCTAGAGCTAATACATGCTAAAAA 160

||||||||||||||||||||||||||||||||| ||| |||||| | ||||||||||||||||||||||||||||| ||

Tgt 80 + TTAAATCAGTTATCGTTTATTTGATAGTACCTT--TACCTGGATAACCGTGGTAATTCTAGAGCTAATACATGCTAACAA 157

Qry 161 + CCTCGACTTCGGAAGGGGTGTATTTATTAGATAAAAAACCAATGCCCTTCGGG 213

|| ||||||||| ||||||||||||||||||||||||||||||||||||||||

Tgt 158 + CCCCGACTTCGGGAGGGGTGTATTTATTAGATAAAAAACCAATGCCCTTCGGG 210

213 cols, 201 ids (94.4%), 3 gaps (1.4%)

Query 213nt >Otu16

Target 213nt >Otu10

Qry 1 + TAGTCATATGCTTGTCTCAAAGATTAAGCCATGCATGTCTAAGTATAAGCAAATTGTACGGTGAAACTGCGAATGGCTCA 80

|||||||||||||||||||||||||||||||||||||||||||||||||| |||| ||| ||||||||||||||||||||

Tgt 1 + TAGTCATATGCTTGTCTCAAAGATTAAGCCATGCATGTCTAAGTATAAGC-AATTATACCGTGAAACTGCGAATGGCTCA 79

Qry 81 + TTAAATCAGTTATCGTTTATTTGATAGTACCTTACTACATGGATACCTGTGGTAATTCTAGAGCTAATACATGCTAAAAA 160

|||||||||||||||||||||||||| ||||||||||| |||||| | ||||||||||||||||||||||||||| ||||

Tgt 80 + TTAAATCAGTTATCGTTTATTTGATAATACCTTACTACTTGGATAACCGTGGTAATTCTAGAGCTAATACATGCTGAAAA 159

Qry 161 + CCTCGACTTCGGAAGGGGTGTATTTATTAGATAAAAAACCAATGCCCTTCGGG 213

| |||||||||||||| ||| |||||||||||||||||||||||||||||||

Tgt 160 + TCCCGACTTCGGAAGGGATGTGTTTATTAGATAAAAAACCAATGCCCTTCGGG 212

213 cols, 201 ids (94.4%), 1 gaps (0.5%)

Query 213nt >Otu16

Target 213nt >Otu20

Qry 1 + TAGTCATATGCTTGTCTCAAAGATTAAGCCATGCATGTCTAAGTATAAGCAAATTGTACGGTGAAACTGCGAATGGCTCA 80

||||||||||||||||||||||||||||||||||||||||||||||||| |||| ||| ||||||||||||||||||||

Tgt 1 + CAGTCATATGCTTGTCTCAAAGATTAAGCCATGCATGTCTAAGTATAAGC-AATTATACCGTGAAACTGCGAATGGCTCA 79

Qry 81 + TTAAATCAGTTATCGTTTATTTGATAGTACCTTACTACATGGATACCTGTGGTAATTCTAGAGCTAATACATGCTAAAAA 160

|||||||||||||||||||||||||| ||||||||||| |||||| | ||||||||||||||||||||||||||| ||||

Tgt 80 + TTAAATCAGTTATCGTTTATTTGATAATACCTTACTACTTGGATAACCGTGGTAATTCTAGAGCTAATACATGCTGAAAA 159

Qry 161 + CCTCGACTTCGGAAGGGGTGTATTTATTAGATAAAAAACCAATGCCCTTCGGG 213

| |||||||||||||| ||| |||||||||||||||||||||||||||||||

Tgt 160 + TCCCGACTTCGGAAGGGATGTGTTTATTAGATAAAAAACCAATGCCCTTCGGG 212

213 cols, 200 ids (93.9%), 1 gaps (0.5%)

Query 213nt >Otu16

Target 213nt >Otu14

Qry 1 + TAGTCATATGCTTGTCTCAAAGATTAAGCCATGCATGTCTAAGTATAAGCAAATTGTACGGTGAAACTGCGAATGGCTCA 80

|||||||||||||||||||||||||||||||||||||||||||||||||||| || ||| ||||||||||||||||||||

Tgt 1 + TAGTCATATGCTTGTCTCAAAGATTAAGCCATGCATGTCTAAGTATAAGCAATTTATACAGTGAAACTGCGAATGGCTCA 80

Qry 81 + TTAAATCAGTTATCGTTTATTTGATAGTACCTTACTACATGGATACCTGTGGTAATTCTAGAGCTAATACATGCTAAAAA 160

|||||||||||||||||||||||||||||||||||||| |||||| | ||||||||||||||||||||||||||| ||||

Tgt 81 + TTAAATCAGTTATCGTTTATTTGATAGTACCTTACTACTTGGATAACCGTGGTAATTCTAGAGCTAATACATGCTTAAAA 160

Qry 161 + CCTCGACT--TCGGAAGGGGTGTATTTATTAGATAAAAAACCAATGCCCTTCG 211

| ||||| | ||||||| |||||||||||||||||||| ||||| | | |

Tgt 161 + TCCCGACTGTTTGGAAGGGATGTATTTATTAGATAAAAAATCAATGTCTTCGG 213

213 cols, 195 ids (91.5%), 2 gaps (0.9%)

Query 213nt >Otu16

Target 213nt >Otu18

Qry 1 + TAGTCATATGCTTGTCTCAAAGATTAAGCCATGCATGTCTAAGTATAAGCAA-ATTGTACGGTGAAACTGCGAATGGCTC 79

|||||||||||||||||||||||||||||||||||||||||||||||| ||| |||||| |||||||||||||||||||

Tgt 1 + TAGTCATATGCTTGTCTCAAAGATTAAGCCATGCATGTCTAAGTATAAACAACTTTGTACTGTGAAACTGCGAATGGCTC 80

Qry 80 + ATTAAATCAGTTATCGTTTATTTGATAGTACCTTACTACATGGATACCTGTGGTAATTCTAGAGCTAATACATGC-TAAA 158

|||||||||||||| ||||||||||| || ||| ||||||||||| |||||||||||||||||||||||||||| || |

Tgt 81 + ATTAAATCAGTTATAGTTTATTTGATGGT-TCTTGCTACATGGATAACTGTGGTAATTCTAGAGCTAATACATGCGTATA 159

Qry 159 + AACCTCGACTTC-GGAAGGGGTGTATTTATTAGATAAAAAACCAATGCCCTTCGGG 213

| || ||||||| ||||||||||||||||||||||||||| | || ||||| |||

Tgt 160 + ATCCCCGACTTCTGGAAGGGGTGTATTTATTAGATAAAAA--CCATCCCCTTTGGG 213

216 cols, 196 ids (90.7%), 6 gaps (2.8%)

Query 213nt >Otu16

Target 213nt >Otu15

Qry 1 + TAGTCATATGCTTGTCTCAAAGATTAAGCCATGCATGTCTAAGTATAAGCAAATTGTACGGTGAAACTGCGAATGGCTCA 80

|||||||||||||||||||||||||||||||||||||||||||||||| || || ||| ||||||||||||||||||||

Tgt 1 + TAGTCATATGCTTGTCTCAAAGATTAAGCCATGCATGTCTAAGTATAAACACGTTATACTGTGAAACTGCGAATGGCTCA 80

Qry 81 + TTAAATCAGTTATCGTTTATTTGATAGTACCTTACTACATGGATACCTGTGGTAATTCTAGAGCTAATACATGC-TAAAA 159

||||||||||||| ||||||||||| || || ||||||||||| |||||||||||||||||||||||||||| || ||

Tgt 81 + TTAAATCAGTTATAGTTTATTTGATGGT-TTTTGCTACATGGATAACTGTGGTAATTCTAGAGCTAATACATGCGTATAA 159

Qry 160 + ACCTCGACTTC-GGAAGGGGTGTATTTATTAGATAAAAAACCAATGCCCTTCGGG 213

|| ||||||| ||||||||||||||||||||||||||| ||| | || | |

Tgt 160 + GCCCCGACTTCTGGAAGGGGTGTATTTATTAGATAAAAA-TCAACACTGTTGGTG 213

215 cols, 190 ids (88.4%), 4 gaps (1.9%)

Query >Otu14

%Id TLen Target

94% 213 Otu10

93% 213 Otu20

93% 213 Otu17

92% 213 Otu8

92% 213 Otu9

91% 213 Otu12

87% 213 Otu18

85% 213 Otu15

Query 213nt >Otu14

Target 213nt >Otu10

Qry 1 + TAGTCATATGCTTGTCTCAAAGATTAAGCCATGCATGTCTAAGTATAAGCAATTTATACAGTGAAACTGCGAATGGCTCA 80

|||||||||||||||||||||||||||||||||||||||||||||||||||| |||||| ||||||||||||||||||||

Tgt 1 + TAGTCATATGCTTGTCTCAAAGATTAAGCCATGCATGTCTAAGTATAAGCAA-TTATACCGTGAAACTGCGAATGGCTCA 79

Qry 81 + TTAAATCAGTTATCGTTTATTTGATAGTACCTTACTACTTGGATAACCGTGGTAATTCTAGAGCTAATACATGCTTAAAA 160

|||||||||||||||||||||||||| |||||||||||||||||||||||||||||||||||||||||||||||| ||||

Tgt 80 + TTAAATCAGTTATCGTTTATTTGATAATACCTTACTACTTGGATAACCGTGGTAATTCTAGAGCTAATACATGCTGAAAA 159

Qry 161 + TCCCGACTGTTTGGAAGGGATGTATTTATTAGATAAAAAATCAATGTCTTCGG 213

|||||||| | ||||||||||| |||||||||||||||| ||||| | | |

Tgt 160 + TCCCGACT--TCGGAAGGGATGTGTTTATTAGATAAAAAACCAATGCCCTTCG 210

213 cols, 200 ids (93.9%), 3 gaps (1.4%)

Query 213nt >Otu14

Target 213nt >Otu20

Qry 1 + TAGTCATATGCTTGTCTCAAAGATTAAGCCATGCATGTCTAAGTATAAGCAATTTATACAGTGAAACTGCGAATGGCTCA 80

||||||||||||||||||||||||||||||||||||||||||||||||||| |||||| ||||||||||||||||||||

Tgt 1 + CAGTCATATGCTTGTCTCAAAGATTAAGCCATGCATGTCTAAGTATAAGCAA-TTATACCGTGAAACTGCGAATGGCTCA 79

Qry 81 + TTAAATCAGTTATCGTTTATTTGATAGTACCTTACTACTTGGATAACCGTGGTAATTCTAGAGCTAATACATGCTTAAAA 160

|||||||||||||||||||||||||| |||||||||||||||||||||||||||||||||||||||||||||||| ||||

Tgt 80 + TTAAATCAGTTATCGTTTATTTGATAATACCTTACTACTTGGATAACCGTGGTAATTCTAGAGCTAATACATGCTGAAAA 159

Qry 161 + TCCCGACTGTTTGGAAGGGATGTATTTATTAGATAAAAAATCAATGTCTTCGG 213

|||||||| | ||||||||||| |||||||||||||||| ||||| | | |

Tgt 160 + TCCCGACT--TCGGAAGGGATGTGTTTATTAGATAAAAAACCAATGCCCTTCG 210

213 cols, 199 ids (93.4%), 3 gaps (1.4%)

Query 213nt >Otu14

Target 213nt >Otu17

Qry 1 + TAGTCATATGCTTGTCTCAAAGATTAAGCCATGCATGTCTAAGTATAAGCAATTTATACAGTGAAACTGCGAATGGCTCA 80

|||||||||||||||||||||||||||||||||||||||||||||||||||| ||||| ||||||||||||||||||||

Tgt 1 + TAGTCATATGCTTGTCTCAAAGATTAAGCCATGCATGTCTAAGTATAAGCAA-CTATACGGTGAAACTGCGAATGGCTCA 79

Qry 81 + TTAAATCAGTTATCGTTTATTTGATAGTACCTTACTACTTGGATAACCGTGGTAATTCTAGAGCTAATACATGCTTAAAA 160

|||||||||||||||||||||||||||||||||||||| |||||||||||||||||||||||||||||||||||| ||||

Tgt 80 + TTAAATCAGTTATCGTTTATTTGATAGTACCTTACTACATGGATAACCGTGGTAATTCTAGAGCTAATACATGCTAAAAA 159

Qry 161 + TCCCGACTGTTTGGAAGGGATGTATTTATTAGATAAAAAATCAATGTCTTCGG 213

||||||| | ||||||| |||||||||||||||||||| ||||| | | |

Tgt 160 + CCCCGACT--TCGGAAGGGGTGTATTTATTAGATAAAAAACCAATGCCCTTCG 210

213 cols, 198 ids (93.0%), 3 gaps (1.4%)

Query 213nt >Otu14

Target 213nt >Otu8

Qry 1 + TAGTCATATGCTTGTCTCAAAGATTAAGCCATGCATGTCTAAGTATAAGCAATTTATACAGTGAAACTGCGAATGGCTCA 80

||||||||||||||||||||||||||||||||||||||||||||||||||||||||||| ||||||||||||||||||||

Tgt 1 + TAGTCATATGCTTGTCTCAAAGATTAAGCCATGCATGTCTAAGTATAAGCAATTTATACGGTGAAACTGCGAATGGCTCA 80

Qry 81 + TTAAATCAGTTATCGTTTATTTGATAGTACCTTACTACTTGGATAACCGTGGTAATTCTAGAGCTAATACATGCTTAAAA 160

|||||||||||||||||||||||||||||||||||||| |||||| | ||||||||||||||||||||||||||| ||||

Tgt 81 + TTAAATCAGTTATCGTTTATTTGATAGTACCTTACTACATGGATACCTGTGGTAATTCTAGAGCTAATACATGCTAAAAA 160

Qry 161 + TCCCGACTGTTTGGAAGGGATGTATTTATTAGATAAAAAATCAATGTCTTCGG 213

| ||||| | ||||||| |||||||||||||||||||| ||||| | | |

Tgt 161 + CCTCGACT--TCGGAAGGGGTGTATTTATTAGATAAAAAACCAATGCCCTTCG 211

213 cols, 197 ids (92.5%), 2 gaps (0.9%)

Query 213nt >Otu14

Target 213nt >Otu9

Qry 1 + TAGTCATATGCTTGTCTCAAAGATTAAGCCATGCATGTCTAAGTATAAGCAATTTATACAGTGAAACTGCGAATGGCTCA 80

||||||||||||||||||||||||||||||||||||||||||||||||||| ||||||| ||||||||||||||||||||

Tgt 1 + TAGTCATATGCTTGTCTCAAAGATTAAGCCATGCATGTCTAAGTATAAGCACTTTATACTGTGAAACTGCGAATGGCTCA 80

Qry 81 + TTAAATCAGTTATCGTTTATTTGATAGTACCTTACTACTTGGATAACCGTGGTAATTCTAGAGCTAATACATGCTTAAAA 160

|||||||||||||||||||||||||||||||||||||| |||||| | ||||||||||||||||||||||||||| ||||

Tgt 81 + TTAAATCAGTTATCGTTTATTTGATAGTACCTTACTACATGGATACCTGTGGTAATTCTAGAGCTAATACATGCTAAAAA 160

Qry 161 + TCCCGACTGTTTGGAAGGGATGTATTTATTAGATAAAAAATCAATGTCTTCGG 213

| ||||| | ||||||| |||||||||||||||||||| ||||| | | |

Tgt 161 + CCTCGACT--TCGGAAGGGGTGTATTTATTAGATAAAAAACCAATGCCCTTCG 211

213 cols, 196 ids (92.0%), 2 gaps (0.9%)

Query 213nt >Otu14

Target 213nt >Otu12

Qry 1 + TAGTCATATGCTTGTCTCAAAGATTAAGCCATGCATGTCTAAGTATAAGCAATTTATACAGTGAAACTGCGAATGGCTCA 80

||||||||||||||||||||||||||||||||||||||||||||||||||||| |||| ||||||||||||||||||||

Tgt 1 + TAGTCATATGCTTGTCTCAAAGATTAAGCCATGCATGTCTAAGTATAAGCAAT-CATACTGTGAAACTGCGAATGGCTCA 79

Qry 81 + TTAAATCAGTTATCGTTTATTTGATAGTACCTTACTACTTGGATAACCGTGGTAATTCTAGAGCTAATACATGCTTAAAA 160

||||||||||||||||||||||||||||||||| ||| |||||||||||||||||||||||||||||||||||| | ||

Tgt 80 + TTAAATCAGTTATCGTTTATTTGATAGTACCTT--TACCTGGATAACCGTGGTAATTCTAGAGCTAATACATGCTAACAA 157

Qry 161 + TCCCGACTGTTTGGAAGGGATGTATTTATTAGATAAAAAATCAATGTCTTCGG 213

||||||| | || |||| |||||||||||||||||||| ||||| | | |

Tgt 158 + CCCCGACT--TCGGGAGGGGTGTATTTATTAGATAAAAAACCAATGCCCTTCG 208

213 cols, 194 ids (91.1%), 5 gaps (2.3%)

Query 213nt >Otu14

Target 213nt >Otu18

Qry 1 + TAGTCATATGCTTGTCTCAAAGATTAAGCCATGCATGTCTAAGTATAAGCAA-TTTATACAGTGAAACTGCGAATGGCTC 79

|||||||||||||||||||||||||||||||||||||||||||||||| ||| ||| ||| |||||||||||||||||||

Tgt 1 + TAGTCATATGCTTGTCTCAAAGATTAAGCCATGCATGTCTAAGTATAAACAACTTTGTACTGTGAAACTGCGAATGGCTC 80

Qry 80 + ATTAAATCAGTTATCGTTTATTTGATAGTACCTTACTACTTGGATAACCGTGGTAATTCTAGAGCTAATACATGCTTAAA 159

|||||||||||||| ||||||||||| || ||| |||| |||||||| |||||||||||||||||||||||||| || |

Tgt 81 + ATTAAATCAGTTATAGTTTATTTGATGGT-TCTTGCTACATGGATAACTGTGGTAATTCTAGAGCTAATACATGCGTATA 159

Qry 160 + ATCCCGACTGTTTGGAAGGGATGTATTTATTAGATAAAAAATCAATGTCTTCGG 213

||||| | |||||||| ||||||||||||||||||| || ||| ||

Tgt 160 + ATCCCCGACTTCTGGAAGGGGTGTATTTATTAGATAAAAA-CCATCCCCTTTGG 212

214 cols, 187 ids (87.4%), 3 gaps (1.4%)

Query 213nt >Otu14

Target 213nt >Otu15

Qry 1 + TAGTCATATGCTTGTCTCAAAGATTAAGCCATGCATGTCTAAGTATAAGCAATTTATACAGTGAAACTGCGAATGGCTCA 80

|||||||||||||||||||||||||||||||||||||||||||||||| || |||||| ||||||||||||||||||||

Tgt 1 + TAGTCATATGCTTGTCTCAAAGATTAAGCCATGCATGTCTAAGTATAAACACGTTATACTGTGAAACTGCGAATGGCTCA 80

Qry 81 + TTAAATCAGTTATCGTTTATTTGATAGTACCTTACTACTTGGATAACCGTGGTAATTCTAGAGCTAATACATGCTTAAAA 160

||||||||||||| ||||||||||| || || |||| |||||||| |||||||||||||||||||||||||| || ||

Tgt 81 + TTAAATCAGTTATAGTTTATTTGATGGT-TTTTGCTACATGGATAACTGTGGTAATTCTAGAGCTAATACATGCGTATAA 159

Qry 161 + TCCCGACTGTTTGGAAGGGATGTATTTATTAGATAAAAAATCAATGTCTTCGG 213

||| | |||||||| ||||||||||||||||||| | | || |

Tgt 160 + GCCCCGACTTCTGGAAGGGGTGTATTTATTAGATAAAAATCAACACTGTTGGT 212

213 cols, 182 ids (85.4%), 1 gaps (0.5%)

Query >Otu18

%Id TLen Target

95% 213 Otu15

91% 213 Otu9

91% 213 Otu8

91% 213 Otu17

89% 213 Otu12

88% 213 Otu10

88% 213 Otu20

Query 213nt >Otu18

Target 213nt >Otu15

Qry 1 + TAGTCATATGCTTGTCTCAAAGATTAAGCCATGCATGTCTAAGTATAAACAACTTTGTACTGTGAAACTGCGAATGGCTC 80

||||||||||||||||||||||||||||||||||||||||||||||||||| | || |||||||||||||||||||||||

Tgt 1 + TAGTCATATGCTTGTCTCAAAGATTAAGCCATGCATGTCTAAGTATAAACA-CGTTATACTGTGAAACTGCGAATGGCTC 79

Qry 81 + ATTAAATCAGTTATAGTTTATTTGATGGTTCTTGCTACATGGATAACTGTGGTAATTCTAGAGCTAATACATGCGTATAA 160

|||||||||||||||||||||||||||||| |||||||||||||||||||||||||||||||||||||||||||||||||

Tgt 80 + ATTAAATCAGTTATAGTTTATTTGATGGTTTTTGCTACATGGATAACTGTGGTAATTCTAGAGCTAATACATGCGTATAA 159

Qry 161 + TCCCCGACTTCTGGAAGGGGTGTATTTATTAGATAAAAACCATCCCCTTTGGG 213

|||||||||||||||||||||||||||||||||||||| || | | ||||

Tgt 160 + GCCCCGACTTCTGGAAGGGGTGTATTTATTAGATAAAAATCAACACTGTTGGT 212

213 cols, 202 ids (94.8%), 1 gaps (0.5%)

Query 213nt >Otu18

Target 213nt >Otu9

Qry 1 + TAGTCATATGCTTGTCTCAAAGATTAAGCCATGCATGTCTAAGTATAAACAACTTTGTACTGTGAAACTGCGAATGGCTC 80

|||||||||||||||||||||||||||||||||||||||||||||||| | ||||| |||||||||||||||||||||||

Tgt 1 + TAGTCATATGCTTGTCTCAAAGATTAAGCCATGCATGTCTAAGTATAAGC-ACTTTATACTGTGAAACTGCGAATGGCTC 79

Qry 81 + ATTAAATCAGTTATAGTTTATTTGATGGT-TCTTGCTACATGGATAACTGTGGTAATTCTAGAGCTAATACATGCGTATA 159

|||||||||||||| ||||||||||| || ||| ||||||||||| |||||||||||||||||||||||||||| || |

Tgt 80 + ATTAAATCAGTTATCGTTTATTTGATAGTACCTTACTACATGGATACCTGTGGTAATTCTAGAGCTAATACATGC-TAAA 158

Qry 160 + ATCCCCGACTTCTGGAAGGGGTGTATTTATTAGATAAAAA--CCATCCCCTTTGGG 213

| || ||||||| ||||||||||||||||||||||||||| | || ||||| |||

Tgt 159 + AACCTCGACTTC-GGAAGGGGTGTATTTATTAGATAAAAAACCAATGCCCTTCGGG 213

216 cols, 197 ids (91.2%), 6 gaps (2.8%)

Query 213nt >Otu18

Target 213nt >Otu8

Qry 1 + TAGTCATATGCTTGTCTCAAAGATTAAGCCATGCATGTCTAAGTATAAACAACTTTGTACTGTGAAACTGCGAATGGCTC 80

|||||||||||||||||||||||||||||||||||||||||||||||| ||| ||| ||| |||||||||||||||||||

Tgt 1 + TAGTCATATGCTTGTCTCAAAGATTAAGCCATGCATGTCTAAGTATAAGCAA-TTTATACGGTGAAACTGCGAATGGCTC 79

Qry 81 + ATTAAATCAGTTATAGTTTATTTGATGGT-TCTTGCTACATGGATAACTGTGGTAATTCTAGAGCTAATACATGCGTATA 159

|||||||||||||| ||||||||||| || ||| ||||||||||| |||||||||||||||||||||||||||| || |

Tgt 80 + ATTAAATCAGTTATCGTTTATTTGATAGTACCTTACTACATGGATACCTGTGGTAATTCTAGAGCTAATACATGC-TAAA 158

Qry 160 + ATCCCCGACTTCTGGAAGGGGTGTATTTATTAGATAAAAA--CCATCCCCTTTGGG 213

| || ||||||| ||||||||||||||||||||||||||| | || ||||| |||

Tgt 159 + AACCTCGACTTC-GGAAGGGGTGTATTTATTAGATAAAAAACCAATGCCCTTCGGG 213

216 cols, 196 ids (90.7%), 6 gaps (2.8%)

Query 213nt >Otu18

Target 213nt >Otu17

Qry 1 + TAGTCATATGCTTGTCTCAAAGATTAAGCCATGCATGTCTAAGTATAAACAACTTTGTACTGTGAAACTGCGAATGGCTC 80

|||||||||||||||||||||||||||||||||||||||||||||||| ||||| ||| |||||||||||||||||||

Tgt 1 + TAGTCATATGCTTGTCTCAAAGATTAAGCCATGCATGTCTAAGTATAAGCAACT--ATACGGTGAAACTGCGAATGGCTC 78

Qry 81 + ATTAAATCAGTTATAGTTTATTTGATGGT-TCTTGCTACATGGATAACTGTGGTAATTCTAGAGCTAATACATGCGTATA 159

|||||||||||||| ||||||||||| || ||| ||||||||||||| |||||||||||||||||||||||||| || |

Tgt 79 + ATTAAATCAGTTATCGTTTATTTGATAGTACCTTACTACATGGATAACCGTGGTAATTCTAGAGCTAATACATGC-TAAA 157

Qry 160 + ATCCCCGACTTCTGGAAGGGGTGTATTTATTAGATAAAAA--CCATCCCCTTTGGG 213

| |||||||||| ||||||||||||||||||||||||||| | || ||||| |||

Tgt 158 + AACCCCGACTTC-GGAAGGGGTGTATTTATTAGATAAAAAACCAATGCCCTTCGGG 212

216 cols, 196 ids (90.7%), 7 gaps (3.2%)

Query 213nt >Otu18

Target 213nt >Otu12

Qry 1 + TAGTCATATGCTTGTCTCAAAGATTAAGCCATGCATGTCTAAGTATAAACAACTTTGTACTGTGAAACTGCGAATGGCTC 80

|||||||||||||||||||||||||||||||||||||||||||||||| ||| | |||||||||||||||||||||||

Tgt 1 + TAGTCATATGCTTGTCTCAAAGATTAAGCCATGCATGTCTAAGTATAAGCAA--TCATACTGTGAAACTGCGAATGGCTC 78

Qry 81 + ATTAAATCAGTTATAGTTTATTTGATGGTTCTTGCTACATGGATAACTGTGGTAATTCTAGAGCTAATACATGCGTATAA 160

|||||||||||||| ||||||||||| || | | ||| |||||||| |||||||||||||||||||||||||| || |

Tgt 79 + ATTAAATCAGTTATCGTTTATTTGATAGTACCT-TTACCTGGATAACCGTGGTAATTCTAGAGCTAATACATGC-TAACA 156

Qry 161 + TCCCCGACTTCTGGAAGGGGTGTATTTATTAGATAAAAA--CCATCCCCTTTGGG 213

|||||||||| || |||||||||||||||||||||||| | || ||||| |||

Tgt 157 + ACCCCGACTTC-GGGAGGGGTGTATTTATTAGATAAAAAACCAATGCCCTTCGGG 210

215 cols, 191 ids (88.8%), 7 gaps (3.3%)

Query 213nt >Otu18

Target 213nt >Otu10

Qry 1 + TAGTCATATGCTTGTCTCAAAGATTAAGCCATGCATGTCTAAGTATAAACAACTTTGTACTGTGAAACTGCGAATGGCTC 80

|||||||||||||||||||||||||||||||||||||||||||||||| ||| || ||| |||||||||||||||||||

Tgt 1 + TAGTCATATGCTTGTCTCAAAGATTAAGCCATGCATGTCTAAGTATAAGCAA--TTATACCGTGAAACTGCGAATGGCTC 78

Qry 81 + ATTAAATCAGTTATAGTTTATTTGATGGT-TCTTGCTACATGGATAACTGTGGTAATTCTAGAGCTAATACATGCGTATA 159

|||||||||||||| ||||||||||| | ||| |||| |||||||| |||||||||||||||||||||||||| | |

Tgt 79 + ATTAAATCAGTTATCGTTTATTTGATAATACCTTACTACTTGGATAACCGTGGTAATTCTAGAGCTAATACATGCTGAAA 158

Qry 160 + ATCCCCGACTTCTGGAAGGGGTGTATTTATTAGATAAAAA--CCATCCCCTTTGGG 213

||||| |||||| ||||||| ||| ||||||||||||||| | || ||||| |||

Tgt 159 + ATCCC-GACTTC-GGAAGGGATGTGTTTATTAGATAAAAAACCAATGCCCTTCGGG 212

216 cols, 191 ids (88.4%), 7 gaps (3.2%)

Query 213nt >Otu18

Target 213nt >Otu20

Qry 1 + TAGTCATATGCTTGTCTCAAAGATTAAGCCATGCATGTCTAAGTATAAACAACTTTGTACTGTGAAACTGCGAATGGCTC 80

||||||||||||||||||||||||||||||||||||||||||||||| ||| || ||| |||||||||||||||||||

Tgt 1 + CAGTCATATGCTTGTCTCAAAGATTAAGCCATGCATGTCTAAGTATAAGCAA--TTATACCGTGAAACTGCGAATGGCTC 78

Qry 81 + ATTAAATCAGTTATAGTTTATTTGATGGT-TCTTGCTACATGGATAACTGTGGTAATTCTAGAGCTAATACATGCGTATA 159

|||||||||||||| ||||||||||| | ||| |||| |||||||| |||||||||||||||||||||||||| | |

Tgt 79 + ATTAAATCAGTTATCGTTTATTTGATAATACCTTACTACTTGGATAACCGTGGTAATTCTAGAGCTAATACATGCTGAAA 158

Qry 160 + ATCCCCGACTTCTGGAAGGGGTGTATTTATTAGATAAAAA--CCATCCCCTTTGGG 213

||||| |||||| ||||||| ||| ||||||||||||||| | || ||||| |||

Tgt 159 + ATCCC-GACTTC-GGAAGGGATGTGTTTATTAGATAAAAAACCAATGCCCTTCGGG 212

216 cols, 190 ids (88.0%), 7 gaps (3.2%)

Query >Otu8

%Id TLen Target

99% 213 Otu9

98% 213 Otu17

95% 213 Otu12

95% 213 Otu10

94% 213 Otu20

89% 213 Otu15

Query 213nt >Otu8

Target 213nt >Otu9

Qry 1 + TAGTCATATGCTTGTCTCAAAGATTAAGCCATGCATGTCTAAGTATAAGCAATTTATACGGTGAAACTGCGAATGGCTCA 80

||||||||||||||||||||||||||||||||||||||||||||||||||| ||||||| ||||||||||||||||||||

Tgt 1 + TAGTCATATGCTTGTCTCAAAGATTAAGCCATGCATGTCTAAGTATAAGCACTTTATACTGTGAAACTGCGAATGGCTCA 80

Qry 81 + TTAAATCAGTTATCGTTTATTTGATAGTACCTTACTACATGGATACCTGTGGTAATTCTAGAGCTAATACATGCTAAAAA 160

||||||||||||||||||||||||||||||||||||||||||||||||||||||||||||||||||||||||||||||||

Tgt 81 + TTAAATCAGTTATCGTTTATTTGATAGTACCTTACTACATGGATACCTGTGGTAATTCTAGAGCTAATACATGCTAAAAA 160

Qry 161 + CCTCGACTTCGGAAGGGGTGTATTTATTAGATAAAAAACCAATGCCCTTCGGG 213

|||||||||||||||||||||||||||||||||||||||||||||||||||||

Tgt 161 + CCTCGACTTCGGAAGGGGTGTATTTATTAGATAAAAAACCAATGCCCTTCGGG 213

213 cols, 211 ids (99.1%), 0 gaps (0.0%)

Query 213nt >Otu8

Target 213nt >Otu17

Qry 1 + TAGTCATATGCTTGTCTCAAAGATTAAGCCATGCATGTCTAAGTATAAGCAATTTATACGGTGAAACTGCGAATGGCTCA 80

|||||||||||||||||||||||||||||||||||||||||||||||||||| ||||||||||||||||||||||||||

Tgt 1 + TAGTCATATGCTTGTCTCAAAGATTAAGCCATGCATGTCTAAGTATAAGCAA-CTATACGGTGAAACTGCGAATGGCTCA 79

Qry 81 + TTAAATCAGTTATCGTTTATTTGATAGTACCTTACTACATGGATACCTGTGGTAATTCTAGAGCTAATACATGCTAAAAA 160

||||||||||||||||||||||||||||||||||||||||||||| | ||||||||||||||||||||||||||||||||

Tgt 80 + TTAAATCAGTTATCGTTTATTTGATAGTACCTTACTACATGGATAACCGTGGTAATTCTAGAGCTAATACATGCTAAAAA 159

Qry 161 + CCTCGACTTCGGAAGGGGTGTATTTATTAGATAAAAAACCAATGCCCTTCGGG 213

|| ||||||||||||||||||||||||||||||||||||||||||||||||||

Tgt 160 + CCCCGACTTCGGAAGGGGTGTATTTATTAGATAAAAAACCAATGCCCTTCGGG 212

213 cols, 208 ids (97.7%), 1 gaps (0.5%)

Query 213nt >Otu8

Target 213nt >Otu12

Qry 1 + TAGTCATATGCTTGTCTCAAAGATTAAGCCATGCATGTCTAAGTATAAGCAATTTATACGGTGAAACTGCGAATGGCTCA 80

||||||||||||||||||||||||||||||||||||||||||||||||||||| |||| ||||||||||||||||||||

Tgt 1 + TAGTCATATGCTTGTCTCAAAGATTAAGCCATGCATGTCTAAGTATAAGCAAT-CATACTGTGAAACTGCGAATGGCTCA 79

Qry 81 + TTAAATCAGTTATCGTTTATTTGATAGTACCTTACTACATGGATACCTGTGGTAATTCTAGAGCTAATACATGCTAAAAA 160

||||||||||||||||||||||||||||||||| ||| |||||| | ||||||||||||||||||||||||||||| ||

Tgt 80 + TTAAATCAGTTATCGTTTATTTGATAGTACCTT--TACCTGGATAACCGTGGTAATTCTAGAGCTAATACATGCTAACAA 157

Qry 161 + CCTCGACTTCGGAAGGGGTGTATTTATTAGATAAAAAACCAATGCCCTTCGGG 213

|| ||||||||| ||||||||||||||||||||||||||||||||||||||||

Tgt 158 + CCCCGACTTCGGGAGGGGTGTATTTATTAGATAAAAAACCAATGCCCTTCGGG 210

213 cols, 202 ids (94.8%), 3 gaps (1.4%)

Query 213nt >Otu8

Target 213nt >Otu10

Qry 1 + TAGTCATATGCTTGTCTCAAAGATTAAGCCATGCATGTCTAAGTATAAGCAATTTATACGGTGAAACTGCGAATGGCTCA 80

|||||||||||||||||||||||||||||||||||||||||||||||||||| |||||| ||||||||||||||||||||

Tgt 1 + TAGTCATATGCTTGTCTCAAAGATTAAGCCATGCATGTCTAAGTATAAGCAA-TTATACCGTGAAACTGCGAATGGCTCA 79

Qry 81 + TTAAATCAGTTATCGTTTATTTGATAGTACCTTACTACATGGATACCTGTGGTAATTCTAGAGCTAATACATGCTAAAAA 160

|||||||||||||||||||||||||| ||||||||||| |||||| | ||||||||||||||||||||||||||| ||||

Tgt 80 + TTAAATCAGTTATCGTTTATTTGATAATACCTTACTACTTGGATAACCGTGGTAATTCTAGAGCTAATACATGCTGAAAA 159

Qry 161 + CCTCGACTTCGGAAGGGGTGTATTTATTAGATAAAAAACCAATGCCCTTCGGG 213

| |||||||||||||| ||| |||||||||||||||||||||||||||||||

Tgt 160 + TCCCGACTTCGGAAGGGATGTGTTTATTAGATAAAAAACCAATGCCCTTCGGG 212

213 cols, 202 ids (94.8%), 1 gaps (0.5%)

Query 213nt >Otu8

Target 213nt >Otu20

Qry 1 + TAGTCATATGCTTGTCTCAAAGATTAAGCCATGCATGTCTAAGTATAAGCAATTTATACGGTGAAACTGCGAATGGCTCA 80

||||||||||||||||||||||||||||||||||||||||||||||||||| |||||| ||||||||||||||||||||

Tgt 1 + CAGTCATATGCTTGTCTCAAAGATTAAGCCATGCATGTCTAAGTATAAGCAA-TTATACCGTGAAACTGCGAATGGCTCA 79

Qry 81 + TTAAATCAGTTATCGTTTATTTGATAGTACCTTACTACATGGATACCTGTGGTAATTCTAGAGCTAATACATGCTAAAAA 160

|||||||||||||||||||||||||| ||||||||||| |||||| | ||||||||||||||||||||||||||| ||||

Tgt 80 + TTAAATCAGTTATCGTTTATTTGATAATACCTTACTACTTGGATAACCGTGGTAATTCTAGAGCTAATACATGCTGAAAA 159

Qry 161 + CCTCGACTTCGGAAGGGGTGTATTTATTAGATAAAAAACCAATGCCCTTCGGG 213

| |||||||||||||| ||| |||||||||||||||||||||||||||||||

Tgt 160 + TCCCGACTTCGGAAGGGATGTGTTTATTAGATAAAAAACCAATGCCCTTCGGG 212

213 cols, 201 ids (94.4%), 1 gaps (0.5%)

Query 213nt >Otu8

Target 213nt >Otu15

Qry 1 + TAGTCATATGCTTGTCTCAAAGATTAAGCCATGCATGTCTAAGTATAAGCAATTTATACGGTGAAACTGCGAATGGCTCA 80

|||||||||||||||||||||||||||||||||||||||||||||||| || |||||| ||||||||||||||||||||

Tgt 1 + TAGTCATATGCTTGTCTCAAAGATTAAGCCATGCATGTCTAAGTATAAACACGTTATACTGTGAAACTGCGAATGGCTCA 80

Qry 81 + TTAAATCAGTTATCGTTTATTTGATAGTACCTTACTACATGGATACCTGTGGTAATTCTAGAGCTAATACATGC-TAAAA 159

||||||||||||| ||||||||||| || || ||||||||||| |||||||||||||||||||||||||||| || ||

Tgt 81 + TTAAATCAGTTATAGTTTATTTGATGGT-TTTTGCTACATGGATAACTGTGGTAATTCTAGAGCTAATACATGCGTATAA 159

Qry 160 + ACCTCGACTTC-GGAAGGGGTGTATTTATTAGATAAAAAACCAATGCCCTTCGGG 213

|| ||||||| ||||||||||||||||||||||||||| ||| | || | |

Tgt 160 + GCCCCGACTTCTGGAAGGGGTGTATTTATTAGATAAAAA-TCAACACTGTTGGTG 213

215 cols, 191 ids (88.8%), 4 gaps (1.9%)

Query >Otu10

%Id TLen Target

100% 213 Otu20

96% 213 Otu17

94% 213 Otu9

94% 213 Otu12

87% 213 Otu15

Query 213nt >Otu10

Target 213nt >Otu20

Qry 1 + TAGTCATATGCTTGTCTCAAAGATTAAGCCATGCATGTCTAAGTATAAGCAATTATACCGTGAAACTGCGAATGGCTCAT 80

|||||||||||||||||||||||||||||||||||||||||||||||||||||||||||||||||||||||||||||||

Tgt 1 + CAGTCATATGCTTGTCTCAAAGATTAAGCCATGCATGTCTAAGTATAAGCAATTATACCGTGAAACTGCGAATGGCTCAT 80

Qry 81 + TAAATCAGTTATCGTTTATTTGATAATACCTTACTACTTGGATAACCGTGGTAATTCTAGAGCTAATACATGCTGAAAAT 160

||||||||||||||||||||||||||||||||||||||||||||||||||||||||||||||||||||||||||||||||

Tgt 81 + TAAATCAGTTATCGTTTATTTGATAATACCTTACTACTTGGATAACCGTGGTAATTCTAGAGCTAATACATGCTGAAAAT 160

Qry 161 + CCCGACTTCGGAAGGGATGTGTTTATTAGATAAAAAACCAATGCCCTTCGGGG 213

|||||||||||||||||||||||||||||||||||||||||||||||||||||

Tgt 161 + CCCGACTTCGGAAGGGATGTGTTTATTAGATAAAAAACCAATGCCCTTCGGGG 213

213 cols, 212 ids (99.5%), 0 gaps (0.0%)

Query 213nt >Otu10

Target 213nt >Otu17

Qry 1 + TAGTCATATGCTTGTCTCAAAGATTAAGCCATGCATGTCTAAGTATAAGCAATTATACCGTGAAACTGCGAATGGCTCAT 80

|||||||||||||||||||||||||||||||||||||||||||||||||||| ||||| |||||||||||||||||||||

Tgt 1 + TAGTCATATGCTTGTCTCAAAGATTAAGCCATGCATGTCTAAGTATAAGCAACTATACGGTGAAACTGCGAATGGCTCAT 80

Qry 81 + TAAATCAGTTATCGTTTATTTGATAATACCTTACTACTTGGATAACCGTGGTAATTCTAGAGCTAATACATGCTGAAAAT 160

||||||||||||||||||||||||| ||||||||||| |||||||||||||||||||||||||||||||||||| ||||

Tgt 81 + TAAATCAGTTATCGTTTATTTGATAGTACCTTACTACATGGATAACCGTGGTAATTCTAGAGCTAATACATGCTAAAAAC 160

Qry 161 + CCCGACTTCGGAAGGGATGTGTTTATTAGATAAAAAACCAATGCCCTTCGGGG 213

|||||||||||||||| ||| ||||||||||||||||||||||||||||||||

Tgt 161 + CCCGACTTCGGAAGGGGTGTATTTATTAGATAAAAAACCAATGCCCTTCGGGG 213

213 cols, 205 ids (96.2%), 0 gaps (0.0%)

Query 213nt >Otu10

Target 213nt >Otu9

Qry 1 + TAGTCATATGCTTGTCTCAAAGATTAAGCCATGCATGTCTAAGTATAAGCA-ATTATACCGTGAAACTGCGAATGGCTCA 79

||||||||||||||||||||||||||||||||||||||||||||||||||| |||||| ||||||||||||||||||||

Tgt 1 + TAGTCATATGCTTGTCTCAAAGATTAAGCCATGCATGTCTAAGTATAAGCACTTTATACTGTGAAACTGCGAATGGCTCA 80

Qry 80 + TTAAATCAGTTATCGTTTATTTGATAATACCTTACTACTTGGATAACCGTGGTAATTCTAGAGCTAATACATGCTGAAAA 159

|||||||||||||||||||||||||| ||||||||||| |||||| | ||||||||||||||||||||||||||| ||||

Tgt 81 + TTAAATCAGTTATCGTTTATTTGATAGTACCTTACTACATGGATACCTGTGGTAATTCTAGAGCTAATACATGCTAAAAA 160

Qry 160 + TCCCGACTTCGGAAGGGATGTGTTTATTAGATAAAAAACCAATGCCCTTCGGG 212

| |||||||||||||| ||| |||||||||||||||||||||||||||||||

Tgt 161 + CCTCGACTTCGGAAGGGGTGTATTTATTAGATAAAAAACCAATGCCCTTCGGG 213

213 cols, 201 ids (94.4%), 1 gaps (0.5%)

Query 213nt >Otu10

Target 213nt >Otu12

Qry 1 + TAGTCATATGCTTGTCTCAAAGATTAAGCCATGCATGTCTAAGTATAAGCAATTATACCGTGAAACTGCGAATGGCTCAT 80

||||||||||||||||||||||||||||||||||||||||||||||||||||| |||| |||||||||||||||||||||

Tgt 1 + TAGTCATATGCTTGTCTCAAAGATTAAGCCATGCATGTCTAAGTATAAGCAATCATACTGTGAAACTGCGAATGGCTCAT 80

Qry 81 + TAAATCAGTTATCGTTTATTTGATAATACCTTACTACTTGGATAACCGTGGTAATTCTAGAGCTAATACATGCTGAAAAT 160

||||||||||||||||||||||||| |||||| ||| |||||||||||||||||||||||||||||||||||| | ||

Tgt 81 + TAAATCAGTTATCGTTTATTTGATAGTACCTT--TACCTGGATAACCGTGGTAATTCTAGAGCTAATACATGCTAACAAC 158

Qry 161 + CCCGACTTCGGAAGGGATGTGTTTATTAGATAAAAAACCAATGCCCTTCGGGG 213

||||||||||| |||| ||| ||||||||||||||||||||||||||||||||

Tgt 159 + CCCGACTTCGGGAGGGGTGTATTTATTAGATAAAAAACCAATGCCCTTCGGGG 211

213 cols, 201 ids (94.4%), 2 gaps (0.9%)

Query 213nt >Otu10

Target 213nt >Otu15

Qry 1 + TAGTCATATGCTTGTCTCAAAGATTAAGCCATGCATGTCTAAGTATAAGCA-ATTATACCGTGAAACTGCGAATGGCTCA 79

|||||||||||||||||||||||||||||||||||||||||||||||| || |||||| ||||||||||||||||||||

Tgt 1 + TAGTCATATGCTTGTCTCAAAGATTAAGCCATGCATGTCTAAGTATAAACACGTTATACTGTGAAACTGCGAATGGCTCA 80

Qry 80 + TTAAATCAGTTATCGTTTATTTGATAATACCTTACTACTTGGATAACCGTGGTAATTCTAGAGCTAATACATGC-TGAAA 158

||||||||||||| ||||||||||| | || |||| |||||||| |||||||||||||||||||||||||| | ||

Tgt 81 + TTAAATCAGTTATAGTTTATTTGATGGT-TTTTGCTACATGGATAACTGTGGTAATTCTAGAGCTAATACATGCGTATAA 159

Qry 159 + ATCCCGACTTC-GGAAGGGATGTGTTTATTAGATAAAAAACCAATGCCCTTCGGG 212

||||||||| ||||||| ||| ||||||||||||||| ||| | || | |

Tgt 160 + GCCCCGACTTCTGGAAGGGGTGTATTTATTAGATAAAAA-TCAACACTGTTGGTG 213

215 cols, 186 ids (86.5%), 5 gaps (2.3%)

Query >Otu15

%Id TLen Target

90% 213 Otu9

89% 213 Otu17

87% 213 Otu12

86% 213 Otu20

Query 213nt >Otu15

Target 213nt >Otu9

Qry 1 + TAGTCATATGCTTGTCTCAAAGATTAAGCCATGCATGTCTAAGTATAAACACGTTATACTGTGAAACTGCGAATGGCTCA 80

|||||||||||||||||||||||||||||||||||||||||||||||| ||| |||||||||||||||||||||||||||

Tgt 1 + TAGTCATATGCTTGTCTCAAAGATTAAGCCATGCATGTCTAAGTATAAGCACTTTATACTGTGAAACTGCGAATGGCTCA 80

Qry 81 + TTAAATCAGTTATAGTTTATTTGATGGT-TTTTGCTACATGGATAACTGTGGTAATTCTAGAGCTAATACATGCGTATAA 159

||||||||||||| ||||||||||| || || ||||||||||| |||||||||||||||||||||||||||| || ||

Tgt 81 + TTAAATCAGTTATCGTTTATTTGATAGTACCTTACTACATGGATACCTGTGGTAATTCTAGAGCTAATACATGC-TAAAA 159

Qry 160 + GCCCCGACTTCTGGAAGGGGTGTATTTATTAGATAAAAA-TCAACACTGTTGGTG 213

|| ||||||| ||||||||||||||||||||||||||| ||| | || | |

Tgt 160 + ACCTCGACTTC-GGAAGGGGTGTATTTATTAGATAAAAAACCAATGCCCTTCGGG 213

215 cols, 193 ids (89.8%), 4 gaps (1.9%)

Query 213nt >Otu15

Target 213nt >Otu17

Qry 1 + TAGTCATATGCTTGTCTCAAAGATTAAGCCATGCATGTCTAAGTATAAACACGTTATACTGTGAAACTGCGAATGGCTCA 80

|||||||||||||||||||||||||||||||||||||||||||||||| || ||||| ||||||||||||||||||||

Tgt 1 + TAGTCATATGCTTGTCTCAAAGATTAAGCCATGCATGTCTAAGTATAAGCA-ACTATACGGTGAAACTGCGAATGGCTCA 79

Qry 81 + TTAAATCAGTTATAGTTTATTTGATGGT-TTTTGCTACATGGATAACTGTGGTAATTCTAGAGCTAATACATGCGTATAA 159

||||||||||||| ||||||||||| || || ||||||||||||| |||||||||||||||||||||||||| || ||

Tgt 80 + TTAAATCAGTTATCGTTTATTTGATAGTACCTTACTACATGGATAACCGTGGTAATTCTAGAGCTAATACATGC-TAAAA 158

Qry 160 + GCCCCGACTTCTGGAAGGGGTGTATTTATTAGATAAAAA-TCAACACTGTTGGTG 213

|||||||||| ||||||||||||||||||||||||||| ||| | || | |

Tgt 159 + ACCCCGACTTC-GGAAGGGGTGTATTTATTAGATAAAAAACCAATGCCCTTCGGG 212

215 cols, 191 ids (88.8%), 5 gaps (2.3%)

Query 213nt >Otu15

Target 213nt >Otu12

Qry 1 + TAGTCATATGCTTGTCTCAAAGATTAAGCCATGCATGTCTAAGTATAAACACGTTATACTGTGAAACTGCGAATGGCTCA 80

|||||||||||||||||||||||||||||||||||||||||||||||| || | |||||||||||||||||||||||||

Tgt 1 + TAGTCATATGCTTGTCTCAAAGATTAAGCCATGCATGTCTAAGTATAAGCA-ATCATACTGTGAAACTGCGAATGGCTCA 79

Qry 81 + TTAAATCAGTTATAGTTTATTTGATGGTTTTTGCTACATGGATAACTGTGGTAATTCTAGAGCTAATACATGCGTATAAG 160

||||||||||||| ||||||||||| || | ||| |||||||| |||||||||||||||||||||||||| || |

Tgt 80 + TTAAATCAGTTATCGTTTATTTGATAGTACCT-TTACCTGGATAACCGTGGTAATTCTAGAGCTAATACATGC-TAACAA 157

Qry 161 + CCCCGACTTCTGGAAGGGGTGTATTTATTAGATAAAAA-TCAACACTGTTGGTG 213

|||||||||| || |||||||||||||||||||||||| ||| | || | |

Tgt 158 + CCCCGACTTC-GGGAGGGGTGTATTTATTAGATAAAAAACCAATGCCCTTCGGG 210

214 cols, 187 ids (87.4%), 5 gaps (2.3%)

Query 213nt >Otu15

Target 213nt >Otu20

Qry 1 + TAGTCATATGCTTGTCTCAAAGATTAAGCCATGCATGTCTAAGTATAAACACGTTATACTGTGAAACTGCGAATGGCTCA 80

||||||||||||||||||||||||||||||||||||||||||||||| || |||||| ||||||||||||||||||||

Tgt 1 + CAGTCATATGCTTGTCTCAAAGATTAAGCCATGCATGTCTAAGTATAAGCA-ATTATACCGTGAAACTGCGAATGGCTCA 79

Qry 81 + TTAAATCAGTTATAGTTTATTTGATGGT-TTTTGCTACATGGATAACTGTGGTAATTCTAGAGCTAATACATGCGTATAA 159

||||||||||||| ||||||||||| | || |||| |||||||| |||||||||||||||||||||||||| | ||

Tgt 80 + TTAAATCAGTTATCGTTTATTTGATAATACCTTACTACTTGGATAACCGTGGTAATTCTAGAGCTAATACATGC-TGAAA 158

Qry 160 + GCCCCGACTTCTGGAAGGGGTGTATTTATTAGATAAAAA-TCAACACTGTTGGTG 213

||||||||| ||||||| ||| ||||||||||||||| ||| | || | |

Tgt 159 + ATCCCGACTTC-GGAAGGGATGTGTTTATTAGATAAAAAACCAATGCCCTTCGGG 212

215 cols, 185 ids (86.0%), 5 gaps (2.3%)

Query >Otu12

%Id TLen Target

96% 213 Otu17

95% 213 Otu9

94% 213 Otu20

Query 213nt >Otu12

Target 213nt >Otu17

Qry 1 + TAGTCATATGCTTGTCTCAAAGATTAAGCCATGCATGTCTAAGTATAAGCAATCATACTGTGAAACTGCGAATGGCTCAT 80

|||||||||||||||||||||||||||||||||||||||||||||||||||| |||| |||||||||||||||||||||

Tgt 1 + TAGTCATATGCTTGTCTCAAAGATTAAGCCATGCATGTCTAAGTATAAGCAACTATACGGTGAAACTGCGAATGGCTCAT 80

Qry 81 + TAAATCAGTTATCGTTTATTTGATAGTACCTT--TACCTGGATAACCGTGGTAATTCTAGAGCTAATACATGCTAACAAC 158

|||||||||||||||||||||||||||||||| ||| |||||||||||||||||||||||||||||||||||||| |||

Tgt 81 + TAAATCAGTTATCGTTTATTTGATAGTACCTTACTACATGGATAACCGTGGTAATTCTAGAGCTAATACATGCTAAAAAC 160

Qry 159 + CCCGACTTCGGGAGGGGTGTATTTATTAGATAAAAAACCAATGCCCTTCGGGG 211

||||||||||| |||||||||||||||||||||||||||||||||||||||||

Tgt 161 + CCCGACTTCGGAAGGGGTGTATTTATTAGATAAAAAACCAATGCCCTTCGGGG 213

213 cols, 205 ids (96.2%), 2 gaps (0.9%)

Query 213nt >Otu12

Target 213nt >Otu9

Qry 1 + TAGTCATATGCTTGTCTCAAAGATTAAGCCATGCATGTCTAAGTATAAGCA-ATCATACTGTGAAACTGCGAATGGCTCA 79

||||||||||||||||||||||||||||||||||||||||||||||||||| | |||||||||||||||||||||||||

Tgt 1 + TAGTCATATGCTTGTCTCAAAGATTAAGCCATGCATGTCTAAGTATAAGCACTTTATACTGTGAAACTGCGAATGGCTCA 80

Qry 80 + TTAAATCAGTTATCGTTTATTTGATAGTACCTT--TACCTGGATAACCGTGGTAATTCTAGAGCTAATACATGCTAACAA 157

||||||||||||||||||||||||||||||||| ||| |||||| | ||||||||||||||||||||||||||||| ||

Tgt 81 + TTAAATCAGTTATCGTTTATTTGATAGTACCTTACTACATGGATACCTGTGGTAATTCTAGAGCTAATACATGCTAAAAA 160

Qry 158 + CCCCGACTTCGGGAGGGGTGTATTTATTAGATAAAAAACCAATGCCCTTCGGG 210

|| ||||||||| ||||||||||||||||||||||||||||||||||||||||

Tgt 161 + CCTCGACTTCGGAAGGGGTGTATTTATTAGATAAAAAACCAATGCCCTTCGGG 213

213 cols, 202 ids (94.8%), 3 gaps (1.4%)

Query 213nt >Otu12

Target 213nt >Otu20

Qry 1 + TAGTCATATGCTTGTCTCAAAGATTAAGCCATGCATGTCTAAGTATAAGCAATCATACTGTGAAACTGCGAATGGCTCAT 80

|||||||||||||||||||||||||||||||||||||||||||||||||||| |||| |||||||||||||||||||||

Tgt 1 + CAGTCATATGCTTGTCTCAAAGATTAAGCCATGCATGTCTAAGTATAAGCAATTATACCGTGAAACTGCGAATGGCTCAT 80

Qry 81 + TAAATCAGTTATCGTTTATTTGATAGTACCTT--TACCTGGATAACCGTGGTAATTCTAGAGCTAATACATGCTAACAAC 158

||||||||||||||||||||||||| |||||| ||| |||||||||||||||||||||||||||||||||||| | ||

Tgt 81 + TAAATCAGTTATCGTTTATTTGATAATACCTTACTACTTGGATAACCGTGGTAATTCTAGAGCTAATACATGCTGAAAAT 160

Qry 159 + CCCGACTTCGGGAGGGGTGTATTTATTAGATAAAAAACCAATGCCCTTCGGGG 211

||||||||||| |||| ||| ||||||||||||||||||||||||||||||||

Tgt 161 + CCCGACTTCGGAAGGGATGTGTTTATTAGATAAAAAACCAATGCCCTTCGGGG 213

213 cols, 200 ids (93.9%), 2 gaps (0.9%)

Query >Otu9

%Id TLen Target

97% 213 Otu17

94% 213 Otu20

Query 213nt >Otu9

Target 213nt >Otu17

Qry 1 + TAGTCATATGCTTGTCTCAAAGATTAAGCCATGCATGTCTAAGTATAAGCACTTTATACTGTGAAACTGCGAATGGCTCA 80

||||||||||||||||||||||||||||||||||||||||||||||||||| ||||| ||||||||||||||||||||

Tgt 1 + TAGTCATATGCTTGTCTCAAAGATTAAGCCATGCATGTCTAAGTATAAGCA-ACTATACGGTGAAACTGCGAATGGCTCA 79

Qry 81 + TTAAATCAGTTATCGTTTATTTGATAGTACCTTACTACATGGATACCTGTGGTAATTCTAGAGCTAATACATGCTAAAAA 160

||||||||||||||||||||||||||||||||||||||||||||| | ||||||||||||||||||||||||||||||||

Tgt 80 + TTAAATCAGTTATCGTTTATTTGATAGTACCTTACTACATGGATAACCGTGGTAATTCTAGAGCTAATACATGCTAAAAA 159

Qry 161 + CCTCGACTTCGGAAGGGGTGTATTTATTAGATAAAAAACCAATGCCCTTCGGG 213

|| ||||||||||||||||||||||||||||||||||||||||||||||||||

Tgt 160 + CCCCGACTTCGGAAGGGGTGTATTTATTAGATAAAAAACCAATGCCCTTCGGG 212

213 cols, 206 ids (96.7%), 1 gaps (0.5%)

Query 213nt >Otu9

Target 213nt >Otu20

Qry 1 + TAGTCATATGCTTGTCTCAAAGATTAAGCCATGCATGTCTAAGTATAAGCACTTTATACTGTGAAACTGCGAATGGCTCA 80

|||||||||||||||||||||||||||||||||||||||||||||||||| |||||| ||||||||||||||||||||

Tgt 1 + CAGTCATATGCTTGTCTCAAAGATTAAGCCATGCATGTCTAAGTATAAGCA-ATTATACCGTGAAACTGCGAATGGCTCA 79

Qry 81 + TTAAATCAGTTATCGTTTATTTGATAGTACCTTACTACATGGATACCTGTGGTAATTCTAGAGCTAATACATGCTAAAAA 160

|||||||||||||||||||||||||| ||||||||||| |||||| | ||||||||||||||||||||||||||| ||||

Tgt 80 + TTAAATCAGTTATCGTTTATTTGATAATACCTTACTACTTGGATAACCGTGGTAATTCTAGAGCTAATACATGCTGAAAA 159

Qry 161 + CCTCGACTTCGGAAGGGGTGTATTTATTAGATAAAAAACCAATGCCCTTCGGG 213

| |||||||||||||| ||| |||||||||||||||||||||||||||||||

Tgt 160 + TCCCGACTTCGGAAGGGATGTGTTTATTAGATAAAAAACCAATGCCCTTCGGG 212

213 cols, 200 ids (93.9%), 1 gaps (0.5%)

Query >Otu17

%Id TLen Target

96% 213 Otu20

Query 213nt >Otu17

Target 213nt >Otu20

Qry 1 + TAGTCATATGCTTGTCTCAAAGATTAAGCCATGCATGTCTAAGTATAAGCAACTATACGGTGAAACTGCGAATGGCTCAT 80

||||||||||||||||||||||||||||||||||||||||||||||||||| ||||| |||||||||||||||||||||

Tgt 1 + CAGTCATATGCTTGTCTCAAAGATTAAGCCATGCATGTCTAAGTATAAGCAATTATACCGTGAAACTGCGAATGGCTCAT 80

Qry 81 + TAAATCAGTTATCGTTTATTTGATAGTACCTTACTACATGGATAACCGTGGTAATTCTAGAGCTAATACATGCTAAAAAC 160

||||||||||||||||||||||||| ||||||||||| |||||||||||||||||||||||||||||||||||| ||||

Tgt 81 + TAAATCAGTTATCGTTTATTTGATAATACCTTACTACTTGGATAACCGTGGTAATTCTAGAGCTAATACATGCTGAAAAT 160

Qry 161 + CCCGACTTCGGAAGGGGTGTATTTATTAGATAAAAAACCAATGCCCTTCGGGG 213

|||||||||||||||| ||| ||||||||||||||||||||||||||||||||

Tgt 161 + CCCGACTTCGGAAGGGATGTGTTTATTAGATAAAAAACCAATGCCCTTCGGGG 213

213 cols, 204 ids (95.8%), 0 gaps (0.0%)

#============================================================================================

3.3 AFLA (2 zOTUs)

Full sequences:

(n = sequence reads/2000 sequences after subsampling to even depth)

>Otu7 (n = 9)

TAGTCATATGCTTGTCTCAAAGATTAAGCCATGCATGTCTAAGTATAAGCACTTTATACTGTGAAACTGCGAATGGCTCA

TTAAATCAGTTATCGTTTATTTGATAGTACCTTACTACATGGATACCTGTGGTAATTCTAGAGCTAATACATGCTGAAAA

CCTCGACTTCGGAAGGGGTGTATTTATTAGATAAAAAACCAATGCCCTTCGGG

>Otu9 (n = 1985)

TAGTCATATGCTTGTCTCAAAGATTAAGCCATGCATGTCTAAGTATAAGCACTTTATACTGTGAAACTGCGAATGGCTCA

TTAAATCAGTTATCGTTTATTTGATAGTACCTTACTACATGGATACCTGTGGTAATTCTAGAGCTAATACATGCTAAAAA

CCTCGACTTCGGAAGGGGTGTATTTATTAGATAAAAAACCAATGCCCTTCGGG

---------------------------

Pairwise global alignments:

Query >Otu7

%Id TLen Target

100% 213 Otu9

Query 213nt >Otu7

Target 213nt >Otu9

Qry 1 + TAGTCATATGCTTGTCTCAAAGATTAAGCCATGCATGTCTAAGTATAAGCACTTTATACTGTGAAACTGCGAATGGCTCA 80

||||||||||||||||||||||||||||||||||||||||||||||||||||||||||||||||||||||||||||||||

Tgt 1 + TAGTCATATGCTTGTCTCAAAGATTAAGCCATGCATGTCTAAGTATAAGCACTTTATACTGTGAAACTGCGAATGGCTCA 80

Qry 81 + TTAAATCAGTTATCGTTTATTTGATAGTACCTTACTACATGGATACCTGTGGTAATTCTAGAGCTAATACATGCTGAAAA 160

||||||||||||||||||||||||||||||||||||||||||||||||||||||||||||||||||||||||||| ||||

Tgt 81 + TTAAATCAGTTATCGTTTATTTGATAGTACCTTACTACATGGATACCTGTGGTAATTCTAGAGCTAATACATGCTAAAAA 160

Qry 161 + CCTCGACTTCGGAAGGGGTGTATTTATTAGATAAAAAACCAATGCCCTTCGGG 213

|||||||||||||||||||||||||||||||||||||||||||||||||||||

Tgt 161 + CCTCGACTTCGGAAGGGGTGTATTTATTAGATAAAAAACCAATGCCCTTCGGG 213

213 cols, 212 ids (99.5%), 0 gaps (0.0%)

#============================================================================================

3.4 CTRO (2 zOTUs)

Full sequences:

(n = sequence reads/2000 sequences after subsampling to even depth)

>Otu2 (n = 32)

TAGTCATATGCTTGTCTCAAAGATTAAGCCATGCATGTCTAAGTATAAGCAATTTATACAGTGAAACTGCGAATGGCTCA

TTAAATCAGTTATCGTTTATTTGATAGTACCTTACTACTTGGATAACCGTGGTAATTCTAGAGCTAATACATGCTTAAAA

TCCCGACTGTTTGGAAGGGATGTATTTATTAGATAAAAAATCAATGCCTTCGG

>Otu14 (n = 1966)

TAGTCATATGCTTGTCTCAAAGATTAAGCCATGCATGTCTAAGTATAAGCAATTTATACAGTGAAACTGCGAATGGCTCA

TTAAATCAGTTATCGTTTATTTGATAGTACCTTACTACTTGGATAACCGTGGTAATTCTAGAGCTAATACATGCTTAAAA

TCCCGACTGTTTGGAAGGGATGTATTTATTAGATAAAAAATCAATGTCTTCGG

---------------------------

Pairwise global alignments:

Query >Otu2

%Id TLen Target

100% 213 Otu14

Query 213nt >Otu2

Target 213nt >Otu14

Qry 1 + TAGTCATATGCTTGTCTCAAAGATTAAGCCATGCATGTCTAAGTATAAGCAATTTATACAGTGAAACTGCGAATGGCTCA 80

||||||||||||||||||||||||||||||||||||||||||||||||||||||||||||||||||||||||||||||||

Tgt 1 + TAGTCATATGCTTGTCTCAAAGATTAAGCCATGCATGTCTAAGTATAAGCAATTTATACAGTGAAACTGCGAATGGCTCA 80

Qry 81 + TTAAATCAGTTATCGTTTATTTGATAGTACCTTACTACTTGGATAACCGTGGTAATTCTAGAGCTAATACATGCTTAAAA 160

||||||||||||||||||||||||||||||||||||||||||||||||||||||||||||||||||||||||||||||||

Tgt 81 + TTAAATCAGTTATCGTTTATTTGATAGTACCTTACTACTTGGATAACCGTGGTAATTCTAGAGCTAATACATGCTTAAAA 160

Qry 161 + TCCCGACTGTTTGGAAGGGATGTATTTATTAGATAAAAAATCAATGCCTTCGG 213

|||||||||||||||||||||||||||||||||||||||||||||| ||||||

Tgt 161 + TCCCGACTGTTTGGAAGGGATGTATTTATTAGATAAAAAATCAATGTCTTCGG 213

213 cols, 212 ids (99.5%), 0 gaps (0.0%)

#============================================================================================

3.5 SCER (2 zOTUs)

Full sequences:

(n = sequence reads/2000 sequences after subsampling to even depth)

>Otu5 (n = 1988)

TAGTCATATGCTTGTCTCAAAGATTAAGCCATGCATGTCTAAGTATAAGCAATTTATACAGTGAAACTGCGAATGGCTCA

TTAAATCAGTTATCGTTTATTTGATAGTTCCTTTACTACATGGTATAACTGTGGTAATTCTAGAGCTAATACATGCTTAA

AATCTCGACCCTTTGGAAGAGATGTATTTATTAGATAAAAAATCAATGTCTTC

>Otu4 (n = 11)

TAGTCATATGCTTGTCTCAAAGATTAAGCCATGCATGTCTAAGTATAAGCAATTTATACAGTGAAACTGCGAATGGCTCA

TTAAATCAGTTATCGTTTATTTGATAGTTCCTTTACTACATGGTATAACTGTGGTAATTCTAGAGCTAATACATGCTTAA

AATCTCGACCTCTTGGAAGAGATGTATTTATTAGATAAAAAATCAATGTCTTC

---------------------------

Pairwise global alignments:

Query >Otu5

%Id TLen Target

99% 213 Otu4

Query 213nt >Otu5

Target 213nt >Otu4

Qry 1 + TAGTCATATGCTTGTCTCAAAGATTAAGCCATGCATGTCTAAGTATAAGCAATTTATACAGTGAAACTGCGAATGGCTCA 80

||||||||||||||||||||||||||||||||||||||||||||||||||||||||||||||||||||||||||||||||

Tgt 1 + TAGTCATATGCTTGTCTCAAAGATTAAGCCATGCATGTCTAAGTATAAGCAATTTATACAGTGAAACTGCGAATGGCTCA 80

Qry 81 + TTAAATCAGTTATCGTTTATTTGATAGTTCCTTTACTACATGGTATAACTGTGGTAATTCTAGAGCTAATACATGCTTAA 160

||||||||||||||||||||||||||||||||||||||||||||||||||||||||||||||||||||||||||||||||

Tgt 81 + TTAAATCAGTTATCGTTTATTTGATAGTTCCTTTACTACATGGTATAACTGTGGTAATTCTAGAGCTAATACATGCTTAA 160

Qry 161 + AATCTCGACCCTTTGGAAGAGATGTATTTATTAGATAAAAAATCAATGTCTTC 213

|||||||||| |||||||||||||||||||||||||||||||||||||||||

Tgt 161 + AATCTCGACCTCTTGGAAGAGATGTATTTATTAGATAAAAAATCAATGTCTTC 213

213 cols, 211 ids (99.1%), 0 gaps (0.0%)

#============================================================================================

3.6 TMEN (2 zOTUs)

Full sequences:

(n = sequence reads/2000 sequences after subsampling to even depth)

>Otu16 (n = 1992)

TAGTCATATGCTTGTCTCAAAGATTAAGCCATGCATGTCTAAGTATAAGCAAATTGTACGGTGAAACTGCGAATGGCTCA

TTAAATCAGTTATCGTTTATTTGATAGTACCTTACTACATGGATACCTGTGGTAATTCTAGAGCTAATACATGCTAAAAA

CCTCGACTTCGGAAGGGGTGTATTTATTAGATAAAAAACCAATGCCCTTCGGG

>Otu8 (n = 7)

TAGTCATATGCTTGTCTCAAAGATTAAGCCATGCATGTCTAAGTATAAGCAATTTATACGGTGAAACTGCGAATGGCTCA

TTAAATCAGTTATCGTTTATTTGATAGTACCTTACTACATGGATACCTGTGGTAATTCTAGAGCTAATACATGCTAAAAA

CCTCGACTTCGGAAGGGGTGTATTTATTAGATAAAAAACCAATGCCCTTCGGG

---------------------------

Pairwise global alignments:

Query >Otu16

%Id TLen Target

99% 213 Otu8

Query 213nt >Otu16

Target 213nt >Otu8

Qry 1 + TAGTCATATGCTTGTCTCAAAGATTAAGCCATGCATGTCTAAGTATAAGCAAATTGTACGGTGAAACTGCGAATGGCTCA 80

|||||||||||||||||||||||||||||||||||||||||||||||||||| || ||||||||||||||||||||||||

Tgt 1 + TAGTCATATGCTTGTCTCAAAGATTAAGCCATGCATGTCTAAGTATAAGCAATTTATACGGTGAAACTGCGAATGGCTCA 80

Qry 81 + TTAAATCAGTTATCGTTTATTTGATAGTACCTTACTACATGGATACCTGTGGTAATTCTAGAGCTAATACATGCTAAAAA 160

||||||||||||||||||||||||||||||||||||||||||||||||||||||||||||||||||||||||||||||||

Tgt 81 + TTAAATCAGTTATCGTTTATTTGATAGTACCTTACTACATGGATACCTGTGGTAATTCTAGAGCTAATACATGCTAAAAA 160

Qry 161 + CCTCGACTTCGGAAGGGGTGTATTTATTAGATAAAAAACCAATGCCCTTCGGG 213

|||||||||||||||||||||||||||||||||||||||||||||||||||||

Tgt 161 + CCTCGACTTCGGAAGGGGTGTATTTATTAGATAAAAAACCAATGCCCTTCGGG 213

213 cols, 211 ids (99.1%), 0 gaps (0.0%)

#============================================================================================

# 4. LSU

#============================================================================================

4.1 ABRA (8 zOTUs)

Full sequences:

(n = sequence reads/2000 sequences after subsampling to even depth)

>Otu3 (n = 1864)

AAACCAACCGGGATTGCCTCAGTAACGGCGAGTGAAGCGGCAAGAGCTCAAATTTGAAAGCTGGCTCCTTCGGAGTCCGC

ATTGTAATTTGCAGAGGATGCTTTGGGTGCGGCCCCCGTCTAAGTGCCCTGGAACGGGCCGTCAGAGAGGGTGAGAATCC

CGTCTTGGGCGGGGTGTCCGTGCCCGTGTAAAGCTCCTTCGACGAG

>Otu2 (n = 7)

AAACTAACTAGGATTCCCTTAGTAACGGCGAGTGAACCGGGAAAAGCTCAAATTTGTAATCTGGCTGTCTTCGATAGTCC

GAGTTGTAATCTATAGACGTGTTTTCCGTGCTGGACCGTATCTAAGTCCCTTGGAACAGGGTATCAAAGAGGGTGACAAT

CCCGTGCTTGATACGACCACCAGTGCTCTGTGATACACGCTCTACG

>Otu12 (n = 6)

AAACTAACAAGGATTCCCCTAGTAACGGCGAGCGAAGCGGGAAGAGCTCAAATTTGAAAGCTGGCACCTCCGGTGTCCGC

GTTGTAATCTCGAGACGTGTTTTCCGTGCGGCTCTATGGACAAGTCCCTTGGAATAGGGCATCGTAGAGGGTGAGAATCC

CGTACTTGCCATGGAAGAACCGTGCTTTGCGATACACGCTCTAAGA

>Otu10 (n = 10)

AAACTAACAAGGATTCCCTTAGTAACGGCGAGTGAACCGGGAAGAGCTCAAATTTGAAATCTGGCGTCCTCCGGGCGTCC

GAGTTGTAATCTACAGAAACGTTTTCCGTGCTGGACCGTGTCTAAGTCCCTTGGAATAGGGTATCAAAGAGGGTGACAAT

CCCGTACTTGACACGATCACCAGTGCTCTGTGATACGTTTTCTACG

>Otu14 (n = 7)

AAACCAACAGGGATTGCCCCAGTAACGGCGAGTGAAGCGGCAAGAGCTCAAATTTGAAATCTGGCCTCCCCCGGGGGCCC

GAGTTGTAATTTGCAGAGGATGCTTCGGGTGCGGCCGCCGTCTAAGTTCCTTGGAACAGGACGTCAGAGAGGGTGAGAAT

CCCGTCTTGGGCGGGCGGTCCGCGCCCGTGTGAAGCTCCTTCGACG

>Otu18 (n = 9)

AAACCAACCGGGATTGCCTCAGTAACGGCGAGTGAAGCGGCAAGAGCTCAAATTTGAAAGCTGGCTCCTTCGGGGTCCGC

ATTGTAATTTGCAGAGGATGCTTCGGGTGCGGCCCCTGTCTAAGTGCCCTGGAACGGGCCGTCAGAGAGGGTGAGAATCC

CGTCTGGGATGGGGTGTCCGCGCCCGTGTGAAGCTCCTTCGACGAG

>Otu8 (n = 26)

AAACTAACAAGGATTCCCCTAGTAACGGCGAGCGAAGCGGGAAGAGCTCAAATTTGAAAGCTGGCACCTCCGGTGTCCGC

GTTGTAATCTCGAGACGTGTTTTCCGTGTGGCGCTATGGACAAGTCCCTTGGAACAGGGCATCGTAGAGGGTGAAAATCC

CGTACTTGCCATGGATGTACCATGCTTTGTGATACACGCTCTAAGA

>Otu16 (n = 11)

AAACCAACAGGGATTGCCCTAGTAACGGCGAGTGAAGCGGCAACAGCTCAAATTTGAAATCTGGCTCTTTTAGAGTCCGA

GTTGTAATTTGCAGAGGGCGCTTTGGCTTTGGCAGCGGTCCAAGTTCCTTGGAACAGGACGTCACAGAGGGTGAGAATCC

CGTACGTGGTCGCTGGCTATTGCCGTGTAAAGCCCCTTCGACGAGT

---------------------------

Pairwise global alignments:

Query >Otu3

%Id TLen Target

96% 206 Otu18

89% 206 Otu14

82% 206 Otu16

72% 206 Otu8

71% 206 Otu12

69% 206 Otu10

67% 206 Otu2

Query 206nt >Otu3

Target 206nt >Otu18

Qry 1 + AAACCAACCGGGATTGCCTCAGTAACGGCGAGTGAAGCGGCAAGAGCTCAAATTTGAAAGCTGGCTCCTTCGGAGTCCGC 80

||||||||||||||||||||||||||||||||||||||||||||||||||||||||||||||||||||||||| ||||||

Tgt 1 + AAACCAACCGGGATTGCCTCAGTAACGGCGAGTGAAGCGGCAAGAGCTCAAATTTGAAAGCTGGCTCCTTCGGGGTCCGC 80

Qry 81 + ATTGTAATTTGCAGAGGATGCTTTGGGTGCGGCCCCCGTCTAAGTGCCCTGGAACGGGCCGTCAGAGAGGGTGAGAATCC 160

||||||||||||||||||||||| |||||||||||| |||||||||||||||||||||||||||||||||||||||||||

Tgt 81 + ATTGTAATTTGCAGAGGATGCTTCGGGTGCGGCCCCTGTCTAAGTGCCCTGGAACGGGCCGTCAGAGAGGGTGAGAATCC 160

Qry 161 + CGTCTTGGGCGGGGTGTCCGTGCCCGTGTAAAGCTCCTTCGACGAG 206

||||| || |||||||||| |||||||| ||||||||||||||||

Tgt 161 + CGTCTGGGATGGGGTGTCCGCGCCCGTGTGAAGCTCCTTCGACGAG 206

206 cols, 198 ids (96.1%), 0 gaps (0.0%)

Query 206nt >Otu3

Target 206nt >Otu14

Qry 1 + AAACCAACCGGGATTGCCTCAGTAACGGCGAGTGAAGCGGCAAGAGCTCAAATTTGAAAGCTGGC--TCCTTCGGAGTCC 78

|||||||| ||||||||| |||||||||||||||||||||||||||||||||||||||| ||||| || || | ||

Tgt 1 + AAACCAACAGGGATTGCCCCAGTAACGGCGAGTGAAGCGGCAAGAGCTCAAATTTGAAATCTGGCCTCCCCCGGGGGCCC 80

Qry 79 + GCATTGTAATTTGCAGAGGATGCTTTGGGTGCGGCCCCCGTCTAAGTGCCCTGGAACGGGCCGTCAGAGAGGGTGAGAAT 158

| |||||||||||||||||||||| |||||||||| |||||||||| || |||||| || |||||||||||||||||||

Tgt 81 + GAGTTGTAATTTGCAGAGGATGCTTCGGGTGCGGCCGCCGTCTAAGTTCCTTGGAACAGGACGTCAGAGAGGGTGAGAAT 160

Qry 159 + CCCGTCTTGGGCGGGGTGTCCGTGCCCGTGTAAAGCTCCTTCGACG 204

||||||||||||||| ||||| |||||||| ||||||||||||||

Tgt 161 + CCCGTCTTGGGCGGGCGGTCCGCGCCCGTGTGAAGCTCCTTCGACG 206

206 cols, 183 ids (88.8%), 2 gaps (1.0%)

Query 206nt >Otu3

Target 206nt >Otu16

Qry 1 + AAACCAACCGGGATTGCCTCAGTAACGGCGAGTGAAGCGGCAAGAGCTCAAATTTGAAAGCTGGCTCCTTCGGAGTCCGC 80

|||||||| ||||||||| ||||||||||||||||||||||| ||||||||||||||| ||||||| || |||||||

Tgt 1 + AAACCAACAGGGATTGCCCTAGTAACGGCGAGTGAAGCGGCAACAGCTCAAATTTGAAATCTGGCTCTTTTAGAGTCCGA 80

Qry 81 + ATTGTAATTTGCAGAGGATGCTTTGGGTGCGGCCCCCGTCTAAGTGCCCTGGAACGGGCCGTCAGAGAGGGTGAGAATCC 160

|||||||||||||||| ||||||| | ||| | ||| |||| || |||||| || ||||| |||||||||||||||

Tgt 81 + GTTGTAATTTGCAGAGGGCGCTTTGGCTTTGGCAGCGGTCCAAGTTCCTTGGAACAGGACGTCACAGAGGGTGAGAATCC 160

Qry 161 + CGTCTTGGGCGGGGTGTCCGTGCCCGTGTAAAGCTCCTTCGACGAG 206

||| | | | || | | ||||||||||| |||||||||||

Tgt 161 + CGT-ACGTGGTCGCTGGCTATTGCCGTGTAAAGCCCCTTCGACGAG 205

206 cols, 168 ids (81.6%), 1 gaps (0.5%)

Query 206nt >Otu3

Target 206nt >Otu8

Qry 1 + AAACCAACCGGGATTGCCTCAGTAACGGCGAGTGAAGCGGCAAGAGCTCAAATTTGAAAGCTGGCTCCTTCGGAGTCCGC 80

|||| ||| ||||| || |||||||||||| ||||||| |||||||||||||||||||||||| ||| ||| ||||||

Tgt 1 + AAACTAACAAGGATTCCCCTAGTAACGGCGAGCGAAGCGGGAAGAGCTCAAATTTGAAAGCTGGCACCTCCGGTGTCCGC 80

Qry 81 + ATTGTAATTTGCAGAGGATGCTTTGGGTGCGGC-CCCCGTCTAAGTGCCCTGGAACGGGCCGTCAGAGAGGGTGAGAATC 159

||||||| | ||| | || ||| ||| ||| | | |||| || |||||| || | || ||||||||| ||||

Tgt 81 + GTTGTAATCTCGAGACG-TGTTTTCCGTGTGGCGCTATGGACAAGTCCCTTGGAACAGGGCATCGTAGAGGGTGAAAATC 159

Qry 160 + CCGT-CTTGGGCGGGGTGTCCGTGCCCGTGTAAAGCTCCTTCGACGA 205

|||| |||| || ||| | | ||| | | | || | ||

Tgt 160 + CCGTACTTGCCATGGATGTACCATGCTTTGTGATACACGCTCTAAGA 206

207 cols, 150 ids (72.5%), 3 gaps (1.4%)

Query 206nt >Otu3

Target 206nt >Otu12

Qry 1 + AAACCAACCGGGATTGCCTCAGTAACGGCGAGTGAAGCGGCAAGAGCTCAAATTTGAAAGCTGGCTCCTTCGGAGTCCGC 80

|||| ||| ||||| || |||||||||||| ||||||| |||||||||||||||||||||||| ||| ||| ||||||

Tgt 1 + AAACTAACAAGGATTCCCCTAGTAACGGCGAGCGAAGCGGGAAGAGCTCAAATTTGAAAGCTGGCACCTCCGGTGTCCGC 80

Qry 81 + ATTGTAATTTGCAGAGGATGCTTTGGGTGCGGC-CCCCGTCTAAGTGCCCTGGAACGGGCCGTCAGAGAGGGTGAGAATC 159

||||||| | ||| | || ||| ||||||| | | |||| || ||||| || | || ||||||||||||||

Tgt 81 + GTTGTAATCTCGAGACG-TGTTTTCCGTGCGGCTCTATGGACAAGTCCCTTGGAATAGGGCATCGTAGAGGGTGAGAATC 159

Qry 160 + CCGT-CTTGGGCGGGGTGTCCGTGCCCGTGTAAAGCTCCTTCGACGA 205

|||| |||| || | | | || | | | || | ||

Tgt 160 + CCGTACTTGCCATGGAAGAACCGTGCTTTGCGATACACGCTCTAAGA 206

207 cols, 148 ids (71.5%), 3 gaps (1.4%)

Query 206nt >Otu3

Target 206nt >Otu10

Qry 1 + AAACCAACCGGGATTGCCTCAGTAACGGCGAGTGAAGCGGCAAGAGCTCAAATTTGAAAGCTGGCTCCTTC--GGAGTCC 78

|||| ||| ||||| ||| |||||||||||||||| ||| |||||||||||||||||| ||||| | || || ||||

Tgt 1 + AAACTAACAAGGATTCCCTTAGTAACGGCGAGTGAACCGGGAAGAGCTCAAATTTGAAATCTGGCGTCCTCCGGGCGTCC 80

Qry 79 + GCATTGTAATTTGCAGAGGATGCTTTGGGTGCGGCCCCCGTCTAAGTGCCCTGGAACGGGCCGTCAGAGAGGGTGAGAAT 158

| ||||||| | |||| || | || || |||||||| || ||||| || ||| ||||||||| |||

Tgt 81 + GAGTTGTAATCTACAGAAACGTTTTCCGTGCTGGACCGTGTCTAAGTCCCTTGGAATAGGGTATCAAAGAGGGTGACAAT 160

Qry 159 + CCCGTCTTGGGCGGGGTGTCCGTGCCCGTGTAAAGCTCCTTCGACG 204

||||| | | | | | || | ||| | | ||| |||

Tgt 161 + CCCGTACTTGACACGATCACCAGTGCTCTGTGATACGTTTTCTACG 206

206 cols, 142 ids (68.9%), 2 gaps (1.0%)

Query 206nt >Otu3

Target 206nt >Otu2

Qry 1 + AAACCAACCGGGATTGCCTCAGTAACGGCGAGTGAAGCGGCAAGAGCTCAAATTTGAAAGCTGGCT--CCTTCGGAGTCC 78

|||| ||| ||||| ||| |||||||||||||||| ||| || |||||||||||| || |||||| | | |||||

Tgt 1 + AAACTAACTAGGATTCCCTTAGTAACGGCGAGTGAACCGGGAAAAGCTCAAATTTGTAATCTGGCTGTCTTCGATAGTCC 80

Qry 79 + GCATTGTAATTTGCAGAGGATGCTTTGGGTGCGGCCCCCGTCTAAGTGCCCTGGAACGGGCCGTCAGAGAGGGTGAGAAT 158

| ||||||| | ||| | || | || || ||||||| || |||||| || ||| ||||||||| |||

Tgt 81 + GAGTTGTAATCTATAGACGTGTTTTCCGTGCTGGACCGTATCTAAGTCCCTTGGAACAGGGTATCAAAGAGGGTGACAAT 160

Qry 159 + CCCGTCTTGGGCGGGGTGTCCGTGCCCGTGTAAAGCTCCTTCGACG 204

||||| | | | || | ||| | | | || |||

Tgt 161 + CCCGTGCTTGATACGACCACCAGTGCTCTGTGATACACGCTCTACG 206

206 cols, 137 ids (66.5%), 2 gaps (1.0%)

Query >Otu2

%Id TLen Target

89% 206 Otu10

79% 206 Otu12

78% 206 Otu8

71% 206 Otu16

68% 206 Otu14

68% 206 Otu18

Query 206nt >Otu2

Target 206nt >Otu10

Qry 1 + AAACTAACTAGGATTCCCTTAGTAACGGCGAGTGAACCGGGAAAAGCTCAAATTTGTAATCTGGCTGTCTTCGATAGTCC 80

|||||||| |||||||||||||||||||||||||||||||||| |||||||||||| |||||||| || || ||||

Tgt 1 + AAACTAACAAGGATTCCCTTAGTAACGGCGAGTGAACCGGGAAGAGCTCAAATTTGAAATCTGGCGTCCTCCGGGCGTCC 80

Qry 81 + GAGTTGTAATCTATAGACGTGTTTTCCGTGCTGGACCGTATCTAAGTCCCTTGGAACAGGGTATCAAAGAGGGTGACAAT 160

||||||||||||| ||| ||||||||||||||||||| |||||||||||||||| |||||||||||||||||||||||

Tgt 81 + GAGTTGTAATCTACAGAAACGTTTTCCGTGCTGGACCGTGTCTAAGTCCCTTGGAATAGGGTATCAAAGAGGGTGACAAT 160

Qry 161 + CCCGTGCTTGATACGACCACCAGTGCTCTGTGATACACGCTCTACG 206

||||| ||||| |||| ||||||||||||||||||| ||||||

Tgt 161 + CCCGTACTTGACACGATCACCAGTGCTCTGTGATACGTTTTCTACG 206

206 cols, 183 ids (88.8%), 0 gaps (0.0%)

Query 206nt >Otu2

Target 206nt >Otu12

Qry 1 + AAACTAACTAGGATTCCCTTAGTAACGGCGAGTGAACCGGGAAAAGCTCAAATTTGTAATCTGGCTGTCTTCGATAGTCC 80

|||||||| ||||||||| ||||||||||||| ||| |||||| |||||||||||| || ||||| | || ||||

Tgt 1 + AAACTAACAAGGATTCCCCTAGTAACGGCGAGCGAAGCGGGAAGAGCTCAAATTTGAAAGCTGGC--ACCTCCGGTGTCC 78

Qry 81 + GAGTTGTAATCTATAGACGTGTTTTCCGTGCTGGACCGTATCTAAGTCCCTTGGAACAGGGTATCAAAGAGGGTGACAAT 160

| |||||||||| ||||||||||||||||| | | | ||||||||||||| |||| ||| ||||||||| |||

Tgt 79 + GCGTTGTAATCTCGAGACGTGTTTTCCGTGCGGCTCTATGGACAAGTCCCTTGGAATAGGGCATCGTAGAGGGTGAGAAT 158

Qry 161 + CCCGTGCTTGATA-CGACCACCAGTGCTCTGTGATACACGCTCTACG 206

||||| |||| | || | | ||||| || ||||||||||||| |

Tgt 159 + CCCGTACTTGCCATGGAAGAACCGTGCTTTGCGATACACGCTCTAAG 205

207 cols, 163 ids (78.7%), 3 gaps (1.4%)

Query 206nt >Otu2

Target 206nt >Otu8

Qry 1 + AAACTAACTAGGATTCCCTTAGTAACGGCGAGTGAACCGGGAAAAGCTCAAATTTGTAATCTGGCTGTCTTCGATAGTCC 80

|||||||| ||||||||| ||||||||||||| ||| |||||| |||||||||||| || ||||| | || ||||

Tgt 1 + AAACTAACAAGGATTCCCCTAGTAACGGCGAGCGAAGCGGGAAGAGCTCAAATTTGAAAGCTGGC--ACCTCCGGTGTCC 78

Qry 81 + GAGTTGTAATCTATAGACGTGTTTTCCGTGCTGGACCGTATCTAAGTCCCTTGGAACAGGGTATCAAAGAGGGTGACAAT 160

| |||||||||| |||||||||||||||| | | | |||||||||||||||||| ||| ||||||||| |||

Tgt 79 + GCGTTGTAATCTCGAGACGTGTTTTCCGTGTGGCGCTATGGACAAGTCCCTTGGAACAGGGCATCGTAGAGGGTGAAAAT 158

Qry 161 + CCCGTGCTTGATA-CGACCACCAGTGCTCTGTGATACACGCTCTACG 206

||||| |||| | || | |||| |||||||||||||||| |

Tgt 159 + CCCGTACTTGCCATGGATGTACCATGCTTTGTGATACACGCTCTAAG 205

207 cols, 162 ids (78.3%), 3 gaps (1.4%)

Query 206nt >Otu2

Target 206nt >Otu16

Qry 1 + AAACTAACTAGGATTCCCTTAGTAACGGCGAGTGAACCGGGAAAAGCTCAAATTTGTAATCTGGCTGTCTTCGATAGTCC 80

|||| ||| ||||| || ||||||||||||||||| ||| || |||||||||||| ||||||||| ||| | |||||

Tgt 1 + AAACCAACAGGGATTGCCCTAGTAACGGCGAGTGAAGCGGCAACAGCTCAAATTTGAAATCTGGCT--CTTTTAGAGTCC 78

Qry 81 + GAGTTGTAATCTATAGACGTGTTTTCCGTGCTGGACCGTATCTAAGTCCCTTGGAACAGGGTATCAAAGAGGGTGACAAT 160

|||||||||| | ||| | || | ||| || |||| |||||||||||| ||| ||||||||| |||

Tgt 79 + GAGTTGTAATTTGCAGAGGGCGCTTTGGCTTTGGCAGCGGTCCAAGTTCCTTGGAACAGGACGTCACAGAGGGTGAGAAT 158

Qry 161 + CCCGTGCTTGATACGACCACCAGTGCTCTGTGATACACGCTCTACG 206

||||| | || | || | | ||| ||| | | | || |||

Tgt 159 + CCCGTACGTGGT-CGCTGGCTATTGCCGTGTAAAGCCCCTTCGACG 203

206 cols, 147 ids (71.4%), 3 gaps (1.5%)

Query 206nt >Otu2

Target 206nt >Otu14

Qry 1 + AAACTAACTAGGATTCCCTTAGTAACGGCGAGTGAACCGGGAAAAGCTCAAATTTGTAATCTGGCTGTCTTCGATAGTCC 80

|||| ||| ||||| || |||||||||||||||| ||| || |||||||||||| |||||||| | || | ||

Tgt 1 + AAACCAACAGGGATTGCCCCAGTAACGGCGAGTGAAGCGGCAAGAGCTCAAATTTGAAATCTGGCCTCCCCCGGGGGCCC 80

Qry 81 + GAGTTGTAATCTATAGACGTGTTTTCCGTGCTGGACCGTATCTAAGTCCCTTGGAACAGGGTATCAAAGAGGGTGACAAT 160

|||||||||| | ||| | ||| | || | ||||||| |||||||||||| ||| ||||||||| |||

Tgt 81 + GAGTTGTAATTTGCAGAGGATGCTTCGGGTGCGGCCGCCGTCTAAGTTCCTTGGAACAGGACGTCAGAGAGGGTGAGAAT 160

Qry 161 + CCCGTGCTTGATACGACCACCAGTGCTCTGTGATACACGCTCTACG 206

||||| | | | || | ||||| | | || |||

Tgt 161 + CCCGTCTTGGGCGGGCGGTCCGCGCCCGTGTGAAGCTCCTTCGACG 206

206 cols, 140 ids (68.0%), 0 gaps (0.0%)

Query 206nt >Otu2

Target 206nt >Otu18

Qry 1 + AAACTAACTAGGATTCCCTTAGTAACGGCGAGTGAACCGGGAAAAGCTCAAATTTGTAATCTGGCTGTCTTCGATAGTCC 80

|||| ||| ||||| ||| |||||||||||||||| ||| || |||||||||||| || |||||| | | ||||

Tgt 1 + AAACCAACCGGGATTGCCTCAGTAACGGCGAGTGAAGCGGCAAGAGCTCAAATTTGAAAGCTGGCT--CCTTCGGGGTCC 78

Qry 81 + GAGTTGTAATCTATAGACGTGTTTTCCGTGCTGGACCGTATCTAAGTCCCTTGGAACAGGGTATCAAAGAGGGTGACAAT 160

| ||||||| | ||| | ||| | || || | ||||||| || |||||| || ||| ||||||||| |||

Tgt 79 + GCATTGTAATTTGCAGAGGATGCTTCGGGTGCGGCCCCTGTCTAAGTGCCCTGGAACGGGCCGTCAGAGAGGGTGAGAAT 158

Qry 161 + CCCGTGCTTGATACGACCACCAGTGCTCTGTGATACACGCTCTACG 206

||||| ||| | || | ||||| | | || |||

Tgt 159 + CCCGTCTGGGATGGGGTGTCCGCGCCCGTGTGAAGCTCCTTCGACG 204

206 cols, 140 ids (68.0%), 2 gaps (1.0%)

Query >Otu12

%Id TLen Target

96% 206 Otu8

79% 206 Otu10

69% 206 Otu18

69% 206 Otu14

68% 206 Otu16

Query 206nt >Otu12

Target 206nt >Otu8

Qry 1 + AAACTAACAAGGATTCCCCTAGTAACGGCGAGCGAAGCGGGAAGAGCTCAAATTTGAAAGCTGGCACCTCCGGTGTCCGC 80

||||||||||||||||||||||||||||||||||||||||||||||||||||||||||||||||||||||||||||||||

Tgt 1 + AAACTAACAAGGATTCCCCTAGTAACGGCGAGCGAAGCGGGAAGAGCTCAAATTTGAAAGCTGGCACCTCCGGTGTCCGC 80

Qry 81 + GTTGTAATCTCGAGACGTGTTTTCCGTGCGGCTCTATGGACAAGTCCCTTGGAATAGGGCATCGTAGAGGGTGAGAATCC 160

|||||||||||||||||||||||||||| ||| ||||||||||||||||||||| ||||||||||||||||||| |||||

Tgt 81 + GTTGTAATCTCGAGACGTGTTTTCCGTGTGGCGCTATGGACAAGTCCCTTGGAACAGGGCATCGTAGAGGGTGAAAATCC 160

Qry 161 + CGTACTTGCCATGGAAGAACCGTGCTTTGCGATACACGCTCTAAGA 206

||||||||||||||| | ||| ||||||| ||||||||||||||||

Tgt 161 + CGTACTTGCCATGGATGTACCATGCTTTGTGATACACGCTCTAAGA 206

206 cols, 198 ids (96.1%), 0 gaps (0.0%)

Query 206nt >Otu12

Target 206nt >Otu10

Qry 1 + AAACTAACAAGGATTCCCCTAGTAACGGCGAGCGAAGCGGGAAGAGCTCAAATTTGAAAGCTGGC--ACCTCCGGTGTCC 78

|||||||||||||||||| ||||||||||||| ||| |||||||||||||||||||||| ||||| | | || ||||

Tgt 1 + AAACTAACAAGGATTCCCTTAGTAACGGCGAGTGAACCGGGAAGAGCTCAAATTTGAAATCTGGCGTCCTCCGGGCGTCC 80

Qry 79 + GCGTTGTAATCTCGAGACGTGTTTTCCGTGCGGCTCTATGGACAAGTCCCTTGGAATAGGGCATCGTAGAGGGTGAGAAT 158

| |||||||||| ||| ||||||||||| | | || |||||||||||||||||| ||| ||||||||| |||

Tgt 81 + GAGTTGTAATCTACAGAAACGTTTTCCGTGCTGGACCGTGTCTAAGTCCCTTGGAATAGGGTATCAAAGAGGGTGACAAT 160

Qry 159 + CCCGTACTTGCCATGGAAGAACCGTGCTTTGCGATACACGCTCTAAG 205

|||||||||| || || | | ||||| || ||||| |||| |

Tgt 161 + CCCGTACTTGACA-CGATCACCAGTGCTCTGTGATACGTTTTCTACG 206

207 cols, 164 ids (79.2%), 3 gaps (1.4%)

Query 206nt >Otu12

Target 206nt >Otu18

Qry 1 + AAACTAACAAGGATTCCCCTAGTAACGGCGAGCGAAGCGGGAAGAGCTCAAATTTGAAAGCTGGCACCTCCGGTGTCCGC 80

|||| ||| ||||| || |||||||||||| ||||||| |||||||||||||||||||||||| ||| ||| ||||||

Tgt 1 + AAACCAACCGGGATTGCCTCAGTAACGGCGAGTGAAGCGGCAAGAGCTCAAATTTGAAAGCTGGCTCCTTCGGGGTCCGC 80

Qry 81 + GTTGTAATCTCGAGACGTGTTTTCCGTGCGGCTCTATGGACAAGTCCCTTGGAATAGGGCATCGTAGAGGGTGAGAATCC 160

||||||| | ||| | ||| | | | || |||| || ||||| || | || |||||||||||||||

Tgt 81 + ATTGTAATTTGCAGAGGATGCTTCGGGTGCGGCCCCTGTCTAAGTGCCCTGGAACGGGCCGTCAGAGAGGGTGAGAATCC 160

Qry 161 + CGTACTTGCCATGGAAGAACCGTGCTTTGCGATACACGCTCTAAGA 206

||| || | || | | | || || | | || | ||

Tgt 161 + CGT-CTGGGATGGGGTGTCCGCGCCCGTGTGAAGCTCCTTCGACGA 205

206 cols, 142 ids (68.9%), 1 gaps (0.5%)

Query 206nt >Otu12

Target 206nt >Otu14

Qry 1 + AAACTAACAAGGATTCCCCTAGTAACGGCGAGCGAAGCGGGAAGAGCTCAAATTTGAAAGCTGGC--ACCTCCGGTGTCC 78

|||| |||| ||||| ||| |||||||||||| ||||||| |||||||||||||||||| ||||| || | || | ||

Tgt 1 + AAACCAACAGGGATTGCCCCAGTAACGGCGAGTGAAGCGGCAAGAGCTCAAATTTGAAATCTGGCCTCCCCCGGGGGCCC 80

Qry 79 + GCGTTGTAATCTCGAGACGTGTTTTCCGTGCGGCTCTATGGACAAGTCCCTTGGAATAGGGCATCGTAGAGGGTGAGAAT 158

| |||||||| | ||| | ||| | | | | |||| |||||||| ||| | || |||||||||||||

Tgt 81 + GAGTTGTAATTTGCAGAGGATGCTTCGGGTGCGGCCGCCGTCTAAGTTCCTTGGAACAGGACGTCAGAGAGGGTGAGAAT 160

Qry 159 + CCCGTACTTGCCATGGAAGAACCGTGCTTTGCGATACACGCTCTAAG 205

||||| |||| || | | | || || | | || | |

Tgt 161 + CCCGT-CTTGGGCGGGCGGTCCGCGCCCGTGTGAAGCTCCTTCGACG 206

207 cols, 142 ids (68.6%), 3 gaps (1.4%)

Query 206nt >Otu12

Target 206nt >Otu16

Qry 1 + AAACTAACAAGGATTCCCCTAGTAACGGCGAGCGAAGCGGGAAGAGCTCAAATTTGAAAGCTGGCACCTCCGGTGTCCGC 80

|||| |||| ||||| |||||||||||||||| ||||||| || ||||||||||||||| ||||| | | | |||||

Tgt 1 + AAACCAACAGGGATTGCCCTAGTAACGGCGAGTGAAGCGGCAACAGCTCAAATTTGAAATCTGGCTCTTTTAGAGTCCGA 80

Qry 81 + GTTGTAATCTCGAGACGTGTTTTCCGTGCGGCTCTATGGACAAGTCCCTTGGAATAGGGCATCGTAGAGGGTGAGAATCC 160

|||||||| | ||| | || | | | ||||| |||||||| ||| | || |||||||||||||||

Tgt 81 + GTTGTAATTTGCAGAGGGCGCTTTGGCTTTGGCAGCGGTCCAAGTTCCTTGGAACAGGACGTCACAGAGGGTGAGAATCC 160

Qry 161 + CGTACTTGCCATGGAAGAACCGTGCTTTGCGATACACGCTCTAAGA 206

||||| || | | | ||| || | | | || | ||

Tgt 161 + CGTACGTG--GTCGCTGGCTATTGCCGTGTAAAGCCCCTTCGACGA 204

206 cols, 141 ids (68.4%), 2 gaps (1.0%)

Query >Otu10

%Id TLen Target

78% 206 Otu8

72% 206 Otu14

71% 206 Otu16

70% 206 Otu18

Query 206nt >Otu10

Target 206nt >Otu8

Qry 1 + AAACTAACAAGGATTCCCTTAGTAACGGCGAGTGAACCGGGAAGAGCTCAAATTTGAAATCTGGCGTCCTCCGGGCGTCC 80

|||||||||||||||||| ||||||||||||| ||| |||||||||||||||||||||| ||||| | | || ||||

Tgt 1 + AAACTAACAAGGATTCCCCTAGTAACGGCGAGCGAAGCGGGAAGAGCTCAAATTTGAAAGCTGGC--ACCTCCGGTGTCC 78

Qry 81 + GAGTTGTAATCTACAGAAACGTTTTCCGTGCTGGACCGTGTCTAAGTCCCTTGGAATAGGGTATCAAAGAGGGTGACAAT 160

| |||||||||| ||| |||||||||| | | || ||||||||||||| |||| ||| ||||||||| |||

Tgt 79 + GCGTTGTAATCTCGAGACGTGTTTTCCGTGTGGCGCTATGGACAAGTCCCTTGGAACAGGGCATCGTAGAGGGTGAAAAT 158

Qry 161 + CCCGTACTTGACA-CGATCACCAGTGCTCTGTGATACGTTTTCTACG 206

|||||||||| || ||| | |||| |||||||| |||| |

Tgt 159 + CCCGTACTTGCCATGGATGTACCATGCTTTGTGATACACGCTCTAAG 205

207 cols, 162 ids (78.3%), 3 gaps (1.4%)

Query 206nt >Otu10

Target 206nt >Otu14

Qry 1 + AAACTAACAAGGATTCCCTTAGTAACGGCGAGTGAACCGGGAAGAGCTCAAATTTGAAATCTGGCGTCCTCCGGGCGTCC 80

|||| |||| ||||| || |||||||||||||||| ||| |||||||||||||||||||||||| ||| ||||| | ||

Tgt 1 + AAACCAACAGGGATTGCCCCAGTAACGGCGAGTGAAGCGGCAAGAGCTCAAATTTGAAATCTGGCCTCCCCCGGGGGCCC 80

Qry 81 + GAGTTGTAATCTACAGAAACGTTTTCCGTGCTGGACCGTGTCTAAGTCCCTTGGAATAGGGTATCAAAGAGGGTGACAAT 160

|||||||||| | |||| ||| | || | |||||||| |||||||| ||| ||| ||||||||| |||

Tgt 81 + GAGTTGTAATTTGCAGAGGATGCTTCGGGTGCGGCCGCCGTCTAAGTTCCTTGGAACAGGACGTCAGAGAGGGTGAGAAT 160

Qry 161 + CCCGTACTTGACACGATCACCAGTGCTCTGTGATACGTTTTCTACG 206

||||| | | | | || | ||||| | ||| |||

Tgt 161 + CCCGTCTTGGGCGGGCGGTCCGCGCCCGTGTGAAGCTCCTTCGACG 206

206 cols, 149 ids (72.3%), 0 gaps (0.0%)

Query 206nt >Otu10

Target 206nt >Otu16

Qry 1 + AAACTAACAAGGATTCCCTTAGTAACGGCGAGTGAACCGGGAAGAGCTCAAATTTGAAATCTGGCGTCCTCCGGGCGTCC 80

|||| |||| ||||| || ||||||||||||||||| ||| || ||||||||||||||||||||| || | ||||

Tgt 1 + AAACCAACAGGGATTGCCCTAGTAACGGCGAGTGAAGCGGCAACAGCTCAAATTTGAAATCTGGC--TCTTTTAGAGTCC 78

Qry 81 + GAGTTGTAATCTACAGAAACGTTTTCCGTGCTGGACCGTGTCTAAGTCCCTTGGAATAGGGTATCAAAGAGGGTGACAAT 160

|||||||||| | |||| || | ||| ||| |||| |||||||| ||| ||| ||||||||| |||

Tgt 79 + GAGTTGTAATTTGCAGAGGGCGCTTTGGCTTTGGCAGCGGTCCAAGTTCCTTGGAACAGGACGTCACAGAGGGTGAGAAT 158

Qry 161 + CCCGTACTTGACACGATCACCAGTGCTCTGTGATACGTTTTCTACG 206

||||||| || || | | | ||| ||| | | ||| |||

Tgt 159 + CCCGTACGTG-GTCGCTGGCTATTGCCGTGTAAAGCCCCTTCGACG 203

206 cols, 147 ids (71.4%), 3 gaps (1.5%)

Query 206nt >Otu10

Target 206nt >Otu18

Qry 1 + AAACTAACAAGGATTCCCTTAGTAACGGCGAGTGAACCGGGAAGAGCTCAAATTTGAAATCTGGCGTCCTCCGGGCGTCC 80

|||| ||| ||||| ||| |||||||||||||||| ||| |||||||||||||||||| ||||| | || || ||||

Tgt 1 + AAACCAACCGGGATTGCCTCAGTAACGGCGAGTGAAGCGGCAAGAGCTCAAATTTGAAAGCTGGCTCCTTC--GGGGTCC 78

Qry 81 + GAGTTGTAATCTACAGAAACGTTTTCCGTGCTGGACCGTGTCTAAGTCCCTTGGAATAGGGTATCAAAGAGGGTGACAAT 160

| ||||||| | |||| ||| | || || ||||||||| || ||||| || ||| ||||||||| |||

Tgt 79 + GCATTGTAATTTGCAGAGGATGCTTCGGGTGCGGCCCCTGTCTAAGTGCCCTGGAACGGGCCGTCAGAGAGGGTGAGAAT 158

Qry 161 + CCCGTACTTGACACGATCACCAGTGCTCTGTGATACGTTTTCTACG 206

||||| || | | || | ||||| | ||| |||

Tgt 159 + CCCGTCTGGGATGGGGTGTCCGCGCCCGTGTGAAGCTCCTTCGACG 204

206 cols, 144 ids (69.9%), 2 gaps (1.0%)

Query >Otu14

%Id TLen Target

89% 206 Otu18

82% 206 Otu16

70% 206 Otu8

Query 206nt >Otu14

Target 206nt >Otu18

Qry 1 + AAACCAACAGGGATTGCCCCAGTAACGGCGAGTGAAGCGGCAAGAGCTCAAATTTGAAATCTGGCCTCCCCCGGGGGCCC 80

|||||||| ||||||||| |||||||||||||||||||||||||||||||||||||||| ||||| || |||| ||

Tgt 1 + AAACCAACCGGGATTGCCTCAGTAACGGCGAGTGAAGCGGCAAGAGCTCAAATTTGAAAGCTGGC--TCCTTCGGGGTCC 78

Qry 81 + GAGTTGTAATTTGCAGAGGATGCTTCGGGTGCGGCCGCCGTCTAAGTTCCTTGGAACAGGACGTCAGAGAGGGTGAGAAT 160

| ||||||||||||||||||||||||||||||||| | |||||||| || |||||| || |||||||||||||||||||

Tgt 79 + GCATTGTAATTTGCAGAGGATGCTTCGGGTGCGGCCCCTGTCTAAGTGCCCTGGAACGGGCCGTCAGAGAGGGTGAGAAT 158

Qry 161 + CCCGTCTTGGGCGGGCGGTCCGCGCCCGTGTGAAGCTCCTTCGACG 206

||||||| || ||| |||||||||||||||||||||||||||||

Tgt 159 + CCCGTCTGGGATGGGGTGTCCGCGCCCGTGTGAAGCTCCTTCGACG 204

206 cols, 183 ids (88.8%), 2 gaps (1.0%)

Query 206nt >Otu14

Target 206nt >Otu16

Qry 1 + AAACCAACAGGGATTGCCCCAGTAACGGCGAGTGAAGCGGCAAGAGCTCAAATTTGAAATCTGGCCTCCCCCGGGGGCCC 80

||||||||||||||||||| ||||||||||||||||||||||| ||||||||||||||||||||| | | | ||

Tgt 1 + AAACCAACAGGGATTGCCCTAGTAACGGCGAGTGAAGCGGCAACAGCTCAAATTTGAAATCTGGC--TCTTTTAGAGTCC 78

Qry 81 + GAGTTGTAATTTGCAGAGGATGCTTCGGGTGCGGCCGCCGTCTAAGTTCCTTGGAACAGGACGTCAGAGAGGGTGAGAAT 160

||||||||||||||||||| |||| || | ||| || ||| ||||||||||||||||||||||| |||||||||||||

Tgt 79 + GAGTTGTAATTTGCAGAGGGCGCTTTGGCTTTGGCAGCGGTCCAAGTTCCTTGGAACAGGACGTCACAGAGGGTGAGAAT 158

Qry 161 + CCCGTCTTGGGCGGGCGGTCCGCGCCCGTGTGAAGCTCCTTCGACG 206

||||| | | || | | |||||| |||| |||||||||

Tgt 159 + CCCGT-ACGTGGTCGCTGGCTATTGCCGTGTAAAGCCCCTTCGACG 203

206 cols, 168 ids (81.6%), 3 gaps (1.5%)

Query 206nt >Otu14

Target 206nt >Otu8

Qry 1 + AAACCAACAGGGATTGCCCCAGTAACGGCGAGTGAAGCGGCAAGAGCTCAAATTTGAAATCTGGCCTCCCCCGGGGGCCC 80

|||| |||| ||||| ||| |||||||||||| ||||||| |||||||||||||||||| ||||| || | || | ||

Tgt 1 + AAACTAACAAGGATTCCCCTAGTAACGGCGAGCGAAGCGGGAAGAGCTCAAATTTGAAAGCTGGC--ACCTCCGGTGTCC 78

Qry 81 + GAGTTGTAATTTGCAGAGGATGCTTCGGGTGCGGCCGCCGTCTAAGTTCCTTGGAACAGGACGTCAGAGAGGGTGAGAAT 160

| |||||||| | ||| | ||| | | | | |||| |||||||||||| | || ||||||||| |||

Tgt 79 + GCGTTGTAATCTCGAGACGTGTTTTCCGTGTGGCGCTATGGACAAGTCCCTTGGAACAGGGCATCGTAGAGGGTGAAAAT 158

Qry 161 + CCCGT-CTTGGGCGGGCGGTCCGCGCCCGTGTGAAGCTCCTTCGACG 206

||||| |||| || || | | ||||| | | || | |

Tgt 159 + CCCGTACTTGCCATGGATGTACCATGCTTTGTGATACACGCTCTAAG 205

207 cols, 144 ids (69.6%), 3 gaps (1.4%)

Query >Otu18

%Id TLen Target

80% 206 Otu16

70% 206 Otu8

Query 206nt >Otu18

Target 206nt >Otu16

Qry 1 + AAACCAACCGGGATTGCCTCAGTAACGGCGAGTGAAGCGGCAAGAGCTCAAATTTGAAAGCTGGCTCCTTCGGGGTCCGC 80

|||||||| ||||||||| ||||||||||||||||||||||| ||||||||||||||| ||||||| || | |||||

Tgt 1 + AAACCAACAGGGATTGCCCTAGTAACGGCGAGTGAAGCGGCAACAGCTCAAATTTGAAATCTGGCTCTTTTAGAGTCCGA 80

Qry 81 + ATTGTAATTTGCAGAGGATGCTTCGGGTGCGGCCCCTGTCTAAGTGCCCTGGAACGGGCCGTCAGAGAGGGTGAGAATCC 160

|||||||||||||||| |||| || | ||| | ||| |||| || |||||| || ||||| |||||||||||||||

Tgt 81 + GTTGTAATTTGCAGAGGGCGCTTTGGCTTTGGCAGCGGTCCAAGTTCCTTGGAACAGGACGTCACAGAGGGTGAGAATCC 160

Qry 161 + CGTCTGGGATGGGGTGTCCGCGCCCGTGTGAAGCTCCTTCGACGAG 206

||| | | | | || | |||||| |||| |||||||||||

Tgt 161 + CGTACGTGGT-CGCTGGCTATTGCCGTGTAAAGCCCCTTCGACGAG 205

206 cols, 165 ids (80.1%), 1 gaps (0.5%)

Query 206nt >Otu18

Target 206nt >Otu8

Qry 1 + AAACCAACCGGGATTGCCTCAGTAACGGCGAGTGAAGCGGCAAGAGCTCAAATTTGAAAGCTGGCTCCTTCGGGGTCCGC 80

|||| ||| ||||| || |||||||||||| ||||||| |||||||||||||||||||||||| ||| ||| ||||||

Tgt 1 + AAACTAACAAGGATTCCCCTAGTAACGGCGAGCGAAGCGGGAAGAGCTCAAATTTGAAAGCTGGCACCTCCGGTGTCCGC 80

Qry 81 + ATTGTAATTTGCAGAGGATGCTTCGGGTGCGGCCCCTGTCTAAGTGCCCTGGAACGGGCCGTCAGAGAGGGTGAGAATCC 160

||||||| | ||| | ||| | | | || |||| || |||||| || | || ||||||||| |||||

Tgt 81 + GTTGTAATCTCGAGACGTGTTTTCCGTGTGGCGCTATGGACAAGTCCCTTGGAACAGGGCATCGTAGAGGGTGAAAATCC 160

Qry 161 + CGT-CTGGGATGGGGTGTCCGCGCCCGTGTGAAGCTCCTTCGACGA 205

||| || | || ||| | | ||||| | | || | ||

Tgt 161 + CGTACTTGCCATGGATGTACCATGCTTTGTGATACACGCTCTAAGA 206

206 cols, 145 ids (70.4%), 1 gaps (0.5%)

Query >Otu8

%Id TLen Target

70% 206 Otu16

Query 206nt >Otu8

Target 206nt >Otu16

Qry 1 + AAACTAACAAGGATTCCCCTAGTAACGGCGAGCGAAGCGGGAAGAGCTCAAATTTGAAAGCTGGCACCTCCGGTGTCCGC 80

|||| |||| ||||| |||||||||||||||| ||||||| || ||||||||||||||| ||||| | | | |||||

Tgt 1 + AAACCAACAGGGATTGCCCTAGTAACGGCGAGTGAAGCGGCAACAGCTCAAATTTGAAATCTGGCTCTTTTAGAGTCCGA 80

Qry 81 + GTTGTAATCTCGAGACGTGTTTTCCGTGTGGCGCTATGGACAAGTCCCTTGGAACAGGGCATCGTAGAGGGTGAAAATCC 160

|||||||| | ||| | || | | | | ||||| |||||||||||| | || ||||||||| |||||

Tgt 81 + GTTGTAATTTGCAGAGGGCGCTTTGGCTTTGGCAGCGGTCCAAGTTCCTTGGAACAGGACGTCACAGAGGGTGAGAATCC 160

Qry 161 + CGTACTTGCCATGGATGTACCATGCTTTGTGATACACGCTCTAAGA 206

||||| || | | || ||| ||| | | | || | ||

Tgt 161 + CGTACGTG--GTCGCTGGCTATTGCCGTGTAAAGCCCCTTCGACGA 204

206 cols, 144 ids (69.9%), 2 gaps (1.0%)

#============================================================================================

4.2 CTRO (2 zOTUs)

Full sequences:

(n = sequence reads/2000 sequences after subsampling to even depth)

>Otu15 (n = 1237)

AAACCAACAGGGATTGCCTTAGTAGCGGCGAGTGAAGCGGCAAAAGCTCAAATTTGAAATCTGGCTCTTTCAGAGTCCGA

GTTGTAATTTGAAGAAGGTATCTTTGGGTCTGGCTCTTGTCTATGTTTCTTGGAACAGAACGTCACAGAGGGTGAGAATC

CCGTGCGATGAGATGATCCAGGCCTATGTAAAGTTCCTTCGAAGAG

>Otu20 (n = 763)

AAACCAACAGGGATTGCCTTAGTAGCGGCGAGTGAAGCGGCAAAAGCTCAAATTTGAAATCTGGCTCTTTCAGAGTCCGA

GTTGTAATTTGAAGAAGGTATCTTTGGGTCTGGCCCTTGTCTATGTTTCTTGGAACAGAACGTCACAGAGGGTGAGAATC

CCGTGCGATGAGATGATCCAGGCCTATGTAAAGTTCCTTCGAAGAG

---------------------------

Pairwise global alignments:

Query >Otu15

%Id TLen Target

100% 206 Otu20

Query 206nt >Otu15

Target 206nt >Otu20

Qry 1 + AAACCAACAGGGATTGCCTTAGTAGCGGCGAGTGAAGCGGCAAAAGCTCAAATTTGAAATCTGGCTCTTTCAGAGTCCGA 80

||||||||||||||||||||||||||||||||||||||||||||||||||||||||||||||||||||||||||||||||

Tgt 1 + AAACCAACAGGGATTGCCTTAGTAGCGGCGAGTGAAGCGGCAAAAGCTCAAATTTGAAATCTGGCTCTTTCAGAGTCCGA 80

Qry 81 + GTTGTAATTTGAAGAAGGTATCTTTGGGTCTGGCTCTTGTCTATGTTTCTTGGAACAGAACGTCACAGAGGGTGAGAATC 160

|||||||||||||||||||||||||||||||||| |||||||||||||||||||||||||||||||||||||||||||||

Tgt 81 + GTTGTAATTTGAAGAAGGTATCTTTGGGTCTGGCCCTTGTCTATGTTTCTTGGAACAGAACGTCACAGAGGGTGAGAATC 160

Qry 161 + CCGTGCGATGAGATGATCCAGGCCTATGTAAAGTTCCTTCGAAGAG 206

||||||||||||||||||||||||||||||||||||||||||||||

Tgt 161 + CCGTGCGATGAGATGATCCAGGCCTATGTAAAGTTCCTTCGAAGAG 206

206 cols, 205 ids (99.5%), 0 gaps (0.0%)

#============================================================================================

4.3 ESPI (15 zOTUs)

Full sequences:

(n = sequence reads/2000 sequences after subsampling to even depth)

>Otu27 (n = 212)

AAACCAACAGGGATTGCCTCAGTAACGGCGAGTGAAGCGGCAACAGCTCAAATTTGAAATCTGGCCTCTTTGGGGTCCGA

GTTGTAATTTGTAGAGGATGTTTCGGGTACCGCCTCGGTTTAAATTTCTTGGAACAGAATGTCAGAGAGGGTGAGAATCC

CGTCTTGGACCGGCGGTAGGGGCCCTGTGAAACTCCTTCGACGAGG

>Otu24 (n = 193)

AAACCAACAGGGATTGCCTCAGTAACGGCGAGTGAAGCGGCAACAGCTCAAATTTGAAATCTGGCCTCTTTGGGGTCCGA

GTTGTAATTTGTAGAGGATGTTTCGGGTACCGCCTCGGTTTAAATTTCTTGGAACAGAATGTCAGAGAGGGTGAGAATCC

CGTCTTGGACCGGCGGTAGGGGCCATGTGAAACTCCTTCGACGAGG

>Otu23 (n = 184)

AAACCAACAGGGATTGCCTCAGTAACGGCGAGTGAAGCGGCAACAGCTCAAATTTGAAATCTGGCCTCTTTGGGGTCCGA

GTTGTAATTTGTAGAGGATGTTTCGGGTACCGCCTCGGTTTAAATTTCTTGGAACAGAATGTCAGAGAGGGTGAGAATCC

CGTCTTGGACCGGCGGTAGGGGCCATGTGAAACTCCTTCGACGAGT

>Otu38 (n = 181)

AAACCAACAGGGATTGCCTCAGTAACGGCGAGTGAAGCGGCAACAGCTCAAATTTGAAATCTGGCCTCTTTGGGGTCCGA

GTTGTAATTTGTAGAGGATGTTTCGGGTACCGCCTCGGTTTAAATTTCTTGGAACAGAATGTCAGAGAGGGTGAGAATCC

CGTCTTGGACCGGCGGTAGGGGCCCTGTGGAACTCCTTCGACGGGG

>Otu30 (n = 176)

AAACCAACAGGGATTGCCTCAGTAACGGCGAGTGAAGCGGCAACAGCTCAAATTTGAAATCTGGCCTCTTTGGGGTCCGA

GTTGTAATTTGTAGAGGATGTTTCGGGTACCGCCTCGGTTTAAATTTCTTGGAACAGAATGTCAGAGAGGGTGAGAATCC

CGTCTTGGACCGGCGGTAGGGGCCCTGTGAAACTCCTTCGACGAGT

>Otu28 (n = 165)

AAACCAACAGGGATTGCCTCAGTAACGGCGAGTGAAGCGGCAACAGCTCAAATTTGAAATCTGGCCTCTTTGGGGTCCGA

GTTGTAATTTGTAGAGGATGTTTCGGGTACCGCCTCGGTTTAAATTTCTTGGAACAGAATGTCAGAGAGGGTGAGAATCC

CGTCTTGGACCGGCGGTAGGGGCCATGTGAAACTCCTTCGACGGGG

>Otu33 (n = 147)

AAACCAACAGGGATTGCCTCAGTAACGGCGAGTGAAGCGGCAACAGCTCAAATTTGAAATCTGGCCTCTTTGGGGTCCGA

GTTGTAATTTGTAGAGGATGTTTCGGGTACCGCCTCGGTTTAAATTTCTTGGAACAGAATGTCAGAGAGGGTGAGAATCC

CGTCTTGGACCGGCGGTAGGGGCCATGTGGAACTCCTTCGACGGGG

>Otu32 (n = 141)

AAACCAACAGGGATTGCCTCAGTAACGGCGAGTGAAGCGGCAACAGCTCAAATTTGAAATCTGGCCTCTTTGGGGTCCGA

GTTGTAATTTGTAGAGGATGTTTCGGGTACCGCCTCGGTTTAAATTTCTTGGAACAGAATGTCAGAGAGGGTGAGAATCC

CGTCTTGGACCGGCGGTAGGGGCCATGTGGAACTCCTTCGACGAGT

>Otu36 (n = 120)

AAACCAACAGGGATTGCCTCAGTAACGGCGAGTGAAGCGGCAACAGCTCAAATTTGAAATCTGGCCTCTTTGGGGTCCGA

GTTGTAATTTGTAGAGGATGTTTCGGGTACCGCCTCGGTTTAAATTTCTTGGAACAGAATGTCAGAGAGGGTGAGAATCC

CGTCTTGGACCGGCGGTAGGGGCCCTGTGAAACTCCTTCGACGGGG

>Otu63 (n = 97)

AAACCAACAGGGATTGCCTCAGTAACGGCGAGTGAAGCGGCAACAGCTCAAATTTGAAATCTGGCCTCTTTGGGGTCCGA

GTTGTAATTTGTAGAGGATGTTTCGGGTACCGCCTCGGTTTAAATTTCTTGGAACAGAATGTCAGAGAGGGTGAGAATCC

CGTCTTGGACCGGCGGTAGGGGCCCTGTGGAACTCCTTCGGCGGGG

>Otu29 (n = 91)

AAACCAACAGGGATTGCCTCAGTAACGGCGAGTGAAGCGGCAACAGCTCAAATTTGAAATCTGGCCTCTTTGGGGTCCGA

GTTGTAATTTGTAGAGGATGTTTCGGGTACCGCCTCGGTTTAAATTTCTTGGAACAGAATGTCAGAGAGGGTGAGAATCC

CGTCTTGGACCGGCGGTAGGGCCCATGTGAAACTCCTTCGACGAGG

>Otu41 (n = 85)

AAACCAACAGGGATTGCCTCAGTAACGGCGAGTGAAGCGGCAACAGCTCAAATTTGAAATCTGGCCTCTTTGGGGTCCGA

GTTGTAATTTGTAGAGGATGTTTCGGGTACCGCCTCGGTTTAAATTTCTTGGAACAGAATGTCAGAGAGGGTGAGAATCC

CGTCTTGGACCGGCGGTAGGGCCCCTGTGAAACTCCTTCGACGAGT

>Otu35 (n = 74)

AAACCAACAGGGATTGCCTCAGTAACGGCGAGTGAAGCGGCAACAGCTCAAATTTGAAATCTGGCCTCTTTGGGGTCCGA

GTTGTAATTTGTAGAGGATGTTTCGGGTACCGCCTCGGTTTAAATTTCTTGGAACAGAATGTCAGAGAGGGTGAGAATCC

CGTCTTGGACCGGCGGTAGGGCCCATGTGAAACTCCTTCGACGAGT

>Otu88 (n = 68)

AAACCAACAGGGATTGCCTCAGTAACGGCGAGTGAAGCGGCAACAGCTCAAATTTGAAATCTGGCCTCTTTGGGGTCCGA

GTTGTAATTTGTAGAGGATGTTTCGGGTACCGCCTCGGTTTAAATTTCTTGGAACAGAATGTCAGAGAGGGTGAGAATCC

CGTCTTGGACCGGCGGTAGGGGCCCTGTGGAAATCCTTCGGCGGGG

>Otu44 (n = 67)

AAACCAACAGGGATTGCCTCAGTAACGGCGAGTGAAGCGGCAACAGCTCAAATTTGAAATCTGGCCTCTTTGGGGTCCGA

GTTGTAATTTGTAGAGGATGTTTCGGGTACCGCCTCGGTTTAAATTTCTTGGAACAGAATGTCAGAGAGGGTGAGAATCC

CGTCTTGGACCGGCGGTAGGGGCCCTGTGAAACTCCTTCGACGGGT

---------------------------

Pairwise global alignments:

Query >Otu27

%Id TLen Target

100% 206 Otu30

100% 206 Otu36

100% 206 Otu24

99% 206 Otu29

99% 206 Otu41

99% 206 Otu44

99% 206 Otu38

99% 206 Otu23

99% 206 Otu28

99% 206 Otu35

99% 206 Otu33

99% 206 Otu32

99% 206 Otu63

98% 206 Otu88

Query 206nt >Otu27

Target 206nt >Otu30

Qry 1 + AAACCAACAGGGATTGCCTCAGTAACGGCGAGTGAAGCGGCAACAGCTCAAATTTGAAATCTGGCCTCTTTGGGGTCCGA 80

||||||||||||||||||||||||||||||||||||||||||||||||||||||||||||||||||||||||||||||||

Tgt 1 + AAACCAACAGGGATTGCCTCAGTAACGGCGAGTGAAGCGGCAACAGCTCAAATTTGAAATCTGGCCTCTTTGGGGTCCGA 80

Qry 81 + GTTGTAATTTGTAGAGGATGTTTCGGGTACCGCCTCGGTTTAAATTTCTTGGAACAGAATGTCAGAGAGGGTGAGAATCC 160

||||||||||||||||||||||||||||||||||||||||||||||||||||||||||||||||||||||||||||||||

Tgt 81 + GTTGTAATTTGTAGAGGATGTTTCGGGTACCGCCTCGGTTTAAATTTCTTGGAACAGAATGTCAGAGAGGGTGAGAATCC 160

Qry 161 + CGTCTTGGACCGGCGGTAGGGGCCCTGTGAAACTCCTTCGACGAGG 206

|||||||||||||||||||||||||||||||||||||||||||||

Tgt 161 + CGTCTTGGACCGGCGGTAGGGGCCCTGTGAAACTCCTTCGACGAGT 206

206 cols, 205 ids (99.5%), 0 gaps (0.0%)

Query 206nt >Otu27

Target 206nt >Otu36

Qry 1 + AAACCAACAGGGATTGCCTCAGTAACGGCGAGTGAAGCGGCAACAGCTCAAATTTGAAATCTGGCCTCTTTGGGGTCCGA 80

||||||||||||||||||||||||||||||||||||||||||||||||||||||||||||||||||||||||||||||||

Tgt 1 + AAACCAACAGGGATTGCCTCAGTAACGGCGAGTGAAGCGGCAACAGCTCAAATTTGAAATCTGGCCTCTTTGGGGTCCGA 80

Qry 81 + GTTGTAATTTGTAGAGGATGTTTCGGGTACCGCCTCGGTTTAAATTTCTTGGAACAGAATGTCAGAGAGGGTGAGAATCC 160

||||||||||||||||||||||||||||||||||||||||||||||||||||||||||||||||||||||||||||||||

Tgt 81 + GTTGTAATTTGTAGAGGATGTTTCGGGTACCGCCTCGGTTTAAATTTCTTGGAACAGAATGTCAGAGAGGGTGAGAATCC 160

Qry 161 + CGTCTTGGACCGGCGGTAGGGGCCCTGTGAAACTCCTTCGACGAGG 206

||||||||||||||||||||||||||||||||||||||||||| ||

Tgt 161 + CGTCTTGGACCGGCGGTAGGGGCCCTGTGAAACTCCTTCGACGGGG 206

206 cols, 205 ids (99.5%), 0 gaps (0.0%)

Query 206nt >Otu27

Target 206nt >Otu24

Qry 1 + AAACCAACAGGGATTGCCTCAGTAACGGCGAGTGAAGCGGCAACAGCTCAAATTTGAAATCTGGCCTCTTTGGGGTCCGA 80

||||||||||||||||||||||||||||||||||||||||||||||||||||||||||||||||||||||||||||||||

Tgt 1 + AAACCAACAGGGATTGCCTCAGTAACGGCGAGTGAAGCGGCAACAGCTCAAATTTGAAATCTGGCCTCTTTGGGGTCCGA 80

Qry 81 + GTTGTAATTTGTAGAGGATGTTTCGGGTACCGCCTCGGTTTAAATTTCTTGGAACAGAATGTCAGAGAGGGTGAGAATCC 160

||||||||||||||||||||||||||||||||||||||||||||||||||||||||||||||||||||||||||||||||

Tgt 81 + GTTGTAATTTGTAGAGGATGTTTCGGGTACCGCCTCGGTTTAAATTTCTTGGAACAGAATGTCAGAGAGGGTGAGAATCC 160

Qry 161 + CGTCTTGGACCGGCGGTAGGGGCCCTGTGAAACTCCTTCGACGAGG 206

|||||||||||||||||||||||| |||||||||||||||||||||

Tgt 161 + CGTCTTGGACCGGCGGTAGGGGCCATGTGAAACTCCTTCGACGAGG 206

206 cols, 205 ids (99.5%), 0 gaps (0.0%)

Query 206nt >Otu27

Target 206nt >Otu29

Qry 1 + AAACCAACAGGGATTGCCTCAGTAACGGCGAGTGAAGCGGCAACAGCTCAAATTTGAAATCTGGCCTCTTTGGGGTCCGA 80

||||||||||||||||||||||||||||||||||||||||||||||||||||||||||||||||||||||||||||||||

Tgt 1 + AAACCAACAGGGATTGCCTCAGTAACGGCGAGTGAAGCGGCAACAGCTCAAATTTGAAATCTGGCCTCTTTGGGGTCCGA 80

Qry 81 + GTTGTAATTTGTAGAGGATGTTTCGGGTACCGCCTCGGTTTAAATTTCTTGGAACAGAATGTCAGAGAGGGTGAGAATCC 160

||||||||||||||||||||||||||||||||||||||||||||||||||||||||||||||||||||||||||||||||

Tgt 81 + GTTGTAATTTGTAGAGGATGTTTCGGGTACCGCCTCGGTTTAAATTTCTTGGAACAGAATGTCAGAGAGGGTGAGAATCC 160

Qry 161 + CGTCTTGGACCGGCGGTAGGGGCCCTGTGAAACTCCTTCGACGAGG 206

||||||||||||||||||||| || |||||||||||||||||||||

Tgt 161 + CGTCTTGGACCGGCGGTAGGGCCCATGTGAAACTCCTTCGACGAGG 206

206 cols, 204 ids (99.0%), 0 gaps (0.0%)

Query 206nt >Otu27

Target 206nt >Otu41

Qry 1 + AAACCAACAGGGATTGCCTCAGTAACGGCGAGTGAAGCGGCAACAGCTCAAATTTGAAATCTGGCCTCTTTGGGGTCCGA 80

||||||||||||||||||||||||||||||||||||||||||||||||||||||||||||||||||||||||||||||||

Tgt 1 + AAACCAACAGGGATTGCCTCAGTAACGGCGAGTGAAGCGGCAACAGCTCAAATTTGAAATCTGGCCTCTTTGGGGTCCGA 80

Qry 81 + GTTGTAATTTGTAGAGGATGTTTCGGGTACCGCCTCGGTTTAAATTTCTTGGAACAGAATGTCAGAGAGGGTGAGAATCC 160

||||||||||||||||||||||||||||||||||||||||||||||||||||||||||||||||||||||||||||||||

Tgt 81 + GTTGTAATTTGTAGAGGATGTTTCGGGTACCGCCTCGGTTTAAATTTCTTGGAACAGAATGTCAGAGAGGGTGAGAATCC 160

Qry 161 + CGTCTTGGACCGGCGGTAGGGGCCCTGTGAAACTCCTTCGACGAGG 206

||||||||||||||||||||| |||||||||||||||||||||||

Tgt 161 + CGTCTTGGACCGGCGGTAGGGCCCCTGTGAAACTCCTTCGACGAGT 206

206 cols, 204 ids (99.0%), 0 gaps (0.0%)

Query 206nt >Otu27

Target 206nt >Otu44

Qry 1 + AAACCAACAGGGATTGCCTCAGTAACGGCGAGTGAAGCGGCAACAGCTCAAATTTGAAATCTGGCCTCTTTGGGGTCCGA 80

||||||||||||||||||||||||||||||||||||||||||||||||||||||||||||||||||||||||||||||||

Tgt 1 + AAACCAACAGGGATTGCCTCAGTAACGGCGAGTGAAGCGGCAACAGCTCAAATTTGAAATCTGGCCTCTTTGGGGTCCGA 80

Qry 81 + GTTGTAATTTGTAGAGGATGTTTCGGGTACCGCCTCGGTTTAAATTTCTTGGAACAGAATGTCAGAGAGGGTGAGAATCC 160

||||||||||||||||||||||||||||||||||||||||||||||||||||||||||||||||||||||||||||||||

Tgt 81 + GTTGTAATTTGTAGAGGATGTTTCGGGTACCGCCTCGGTTTAAATTTCTTGGAACAGAATGTCAGAGAGGGTGAGAATCC 160

Qry 161 + CGTCTTGGACCGGCGGTAGGGGCCCTGTGAAACTCCTTCGACGAGG 206

||||||||||||||||||||||||||||||||||||||||||| |

Tgt 161 + CGTCTTGGACCGGCGGTAGGGGCCCTGTGAAACTCCTTCGACGGGT 206

206 cols, 204 ids (99.0%), 0 gaps (0.0%)

Query 206nt >Otu27

Target 206nt >Otu38

Qry 1 + AAACCAACAGGGATTGCCTCAGTAACGGCGAGTGAAGCGGCAACAGCTCAAATTTGAAATCTGGCCTCTTTGGGGTCCGA 80

||||||||||||||||||||||||||||||||||||||||||||||||||||||||||||||||||||||||||||||||

Tgt 1 + AAACCAACAGGGATTGCCTCAGTAACGGCGAGTGAAGCGGCAACAGCTCAAATTTGAAATCTGGCCTCTTTGGGGTCCGA 80

Qry 81 + GTTGTAATTTGTAGAGGATGTTTCGGGTACCGCCTCGGTTTAAATTTCTTGGAACAGAATGTCAGAGAGGGTGAGAATCC 160

||||||||||||||||||||||||||||||||||||||||||||||||||||||||||||||||||||||||||||||||

Tgt 81 + GTTGTAATTTGTAGAGGATGTTTCGGGTACCGCCTCGGTTTAAATTTCTTGGAACAGAATGTCAGAGAGGGTGAGAATCC 160

Qry 161 + CGTCTTGGACCGGCGGTAGGGGCCCTGTGAAACTCCTTCGACGAGG 206

||||||||||||||||||||||||||||| ||||||||||||| ||

Tgt 161 + CGTCTTGGACCGGCGGTAGGGGCCCTGTGGAACTCCTTCGACGGGG 206

206 cols, 204 ids (99.0%), 0 gaps (0.0%)

Query 206nt >Otu27

Target 206nt >Otu23

Qry 1 + AAACCAACAGGGATTGCCTCAGTAACGGCGAGTGAAGCGGCAACAGCTCAAATTTGAAATCTGGCCTCTTTGGGGTCCGA 80

||||||||||||||||||||||||||||||||||||||||||||||||||||||||||||||||||||||||||||||||

Tgt 1 + AAACCAACAGGGATTGCCTCAGTAACGGCGAGTGAAGCGGCAACAGCTCAAATTTGAAATCTGGCCTCTTTGGGGTCCGA 80

Qry 81 + GTTGTAATTTGTAGAGGATGTTTCGGGTACCGCCTCGGTTTAAATTTCTTGGAACAGAATGTCAGAGAGGGTGAGAATCC 160

||||||||||||||||||||||||||||||||||||||||||||||||||||||||||||||||||||||||||||||||

Tgt 81 + GTTGTAATTTGTAGAGGATGTTTCGGGTACCGCCTCGGTTTAAATTTCTTGGAACAGAATGTCAGAGAGGGTGAGAATCC 160

Qry 161 + CGTCTTGGACCGGCGGTAGGGGCCCTGTGAAACTCCTTCGACGAGG 206

|||||||||||||||||||||||| ||||||||||||||||||||

Tgt 161 + CGTCTTGGACCGGCGGTAGGGGCCATGTGAAACTCCTTCGACGAGT 206

206 cols, 204 ids (99.0%), 0 gaps (0.0%)

Query 206nt >Otu27

Target 206nt >Otu28

Qry 1 + AAACCAACAGGGATTGCCTCAGTAACGGCGAGTGAAGCGGCAACAGCTCAAATTTGAAATCTGGCCTCTTTGGGGTCCGA 80

||||||||||||||||||||||||||||||||||||||||||||||||||||||||||||||||||||||||||||||||

Tgt 1 + AAACCAACAGGGATTGCCTCAGTAACGGCGAGTGAAGCGGCAACAGCTCAAATTTGAAATCTGGCCTCTTTGGGGTCCGA 80

Qry 81 + GTTGTAATTTGTAGAGGATGTTTCGGGTACCGCCTCGGTTTAAATTTCTTGGAACAGAATGTCAGAGAGGGTGAGAATCC 160

||||||||||||||||||||||||||||||||||||||||||||||||||||||||||||||||||||||||||||||||

Tgt 81 + GTTGTAATTTGTAGAGGATGTTTCGGGTACCGCCTCGGTTTAAATTTCTTGGAACAGAATGTCAGAGAGGGTGAGAATCC 160

Qry 161 + CGTCTTGGACCGGCGGTAGGGGCCCTGTGAAACTCCTTCGACGAGG 206

|||||||||||||||||||||||| |||||||||||||||||| ||

Tgt 161 + CGTCTTGGACCGGCGGTAGGGGCCATGTGAAACTCCTTCGACGGGG 206

206 cols, 204 ids (99.0%), 0 gaps (0.0%)

Query 206nt >Otu27

Target 206nt >Otu35

Qry 1 + AAACCAACAGGGATTGCCTCAGTAACGGCGAGTGAAGCGGCAACAGCTCAAATTTGAAATCTGGCCTCTTTGGGGTCCGA 80

||||||||||||||||||||||||||||||||||||||||||||||||||||||||||||||||||||||||||||||||

Tgt 1 + AAACCAACAGGGATTGCCTCAGTAACGGCGAGTGAAGCGGCAACAGCTCAAATTTGAAATCTGGCCTCTTTGGGGTCCGA 80

Qry 81 + GTTGTAATTTGTAGAGGATGTTTCGGGTACCGCCTCGGTTTAAATTTCTTGGAACAGAATGTCAGAGAGGGTGAGAATCC 160

||||||||||||||||||||||||||||||||||||||||||||||||||||||||||||||||||||||||||||||||

Tgt 81 + GTTGTAATTTGTAGAGGATGTTTCGGGTACCGCCTCGGTTTAAATTTCTTGGAACAGAATGTCAGAGAGGGTGAGAATCC 160

Qry 161 + CGTCTTGGACCGGCGGTAGGGGCCCTGTGAAACTCCTTCGACGAGG 206

||||||||||||||||||||| || ||||||||||||||||||||

Tgt 161 + CGTCTTGGACCGGCGGTAGGGCCCATGTGAAACTCCTTCGACGAGT 206

206 cols, 203 ids (98.5%), 0 gaps (0.0%)

Query 206nt >Otu27

Target 206nt >Otu33

Qry 1 + AAACCAACAGGGATTGCCTCAGTAACGGCGAGTGAAGCGGCAACAGCTCAAATTTGAAATCTGGCCTCTTTGGGGTCCGA 80

||||||||||||||||||||||||||||||||||||||||||||||||||||||||||||||||||||||||||||||||

Tgt 1 + AAACCAACAGGGATTGCCTCAGTAACGGCGAGTGAAGCGGCAACAGCTCAAATTTGAAATCTGGCCTCTTTGGGGTCCGA 80

Qry 81 + GTTGTAATTTGTAGAGGATGTTTCGGGTACCGCCTCGGTTTAAATTTCTTGGAACAGAATGTCAGAGAGGGTGAGAATCC 160

||||||||||||||||||||||||||||||||||||||||||||||||||||||||||||||||||||||||||||||||

Tgt 81 + GTTGTAATTTGTAGAGGATGTTTCGGGTACCGCCTCGGTTTAAATTTCTTGGAACAGAATGTCAGAGAGGGTGAGAATCC 160

Qry 161 + CGTCTTGGACCGGCGGTAGGGGCCCTGTGAAACTCCTTCGACGAGG 206

|||||||||||||||||||||||| |||| ||||||||||||| ||

Tgt 161 + CGTCTTGGACCGGCGGTAGGGGCCATGTGGAACTCCTTCGACGGGG 206

206 cols, 203 ids (98.5%), 0 gaps (0.0%)

Query 206nt >Otu27

Target 206nt >Otu32

Qry 1 + AAACCAACAGGGATTGCCTCAGTAACGGCGAGTGAAGCGGCAACAGCTCAAATTTGAAATCTGGCCTCTTTGGGGTCCGA 80

||||||||||||||||||||||||||||||||||||||||||||||||||||||||||||||||||||||||||||||||

Tgt 1 + AAACCAACAGGGATTGCCTCAGTAACGGCGAGTGAAGCGGCAACAGCTCAAATTTGAAATCTGGCCTCTTTGGGGTCCGA 80

Qry 81 + GTTGTAATTTGTAGAGGATGTTTCGGGTACCGCCTCGGTTTAAATTTCTTGGAACAGAATGTCAGAGAGGGTGAGAATCC 160

||||||||||||||||||||||||||||||||||||||||||||||||||||||||||||||||||||||||||||||||

Tgt 81 + GTTGTAATTTGTAGAGGATGTTTCGGGTACCGCCTCGGTTTAAATTTCTTGGAACAGAATGTCAGAGAGGGTGAGAATCC 160

Qry 161 + CGTCTTGGACCGGCGGTAGGGGCCCTGTGAAACTCCTTCGACGAGG 206

|||||||||||||||||||||||| |||| |||||||||||||||

Tgt 161 + CGTCTTGGACCGGCGGTAGGGGCCATGTGGAACTCCTTCGACGAGT 206

206 cols, 203 ids (98.5%), 0 gaps (0.0%)

Query 206nt >Otu27

Target 206nt >Otu63

Qry 1 + AAACCAACAGGGATTGCCTCAGTAACGGCGAGTGAAGCGGCAACAGCTCAAATTTGAAATCTGGCCTCTTTGGGGTCCGA 80

||||||||||||||||||||||||||||||||||||||||||||||||||||||||||||||||||||||||||||||||

Tgt 1 + AAACCAACAGGGATTGCCTCAGTAACGGCGAGTGAAGCGGCAACAGCTCAAATTTGAAATCTGGCCTCTTTGGGGTCCGA 80

Qry 81 + GTTGTAATTTGTAGAGGATGTTTCGGGTACCGCCTCGGTTTAAATTTCTTGGAACAGAATGTCAGAGAGGGTGAGAATCC 160

||||||||||||||||||||||||||||||||||||||||||||||||||||||||||||||||||||||||||||||||

Tgt 81 + GTTGTAATTTGTAGAGGATGTTTCGGGTACCGCCTCGGTTTAAATTTCTTGGAACAGAATGTCAGAGAGGGTGAGAATCC 160

Qry 161 + CGTCTTGGACCGGCGGTAGGGGCCCTGTGAAACTCCTTCGACGAGG 206

||||||||||||||||||||||||||||| |||||||||| || ||

Tgt 161 + CGTCTTGGACCGGCGGTAGGGGCCCTGTGGAACTCCTTCGGCGGGG 206

206 cols, 203 ids (98.5%), 0 gaps (0.0%)

Query 206nt >Otu27

Target 206nt >Otu88

Qry 1 + AAACCAACAGGGATTGCCTCAGTAACGGCGAGTGAAGCGGCAACAGCTCAAATTTGAAATCTGGCCTCTTTGGGGTCCGA 80

||||||||||||||||||||||||||||||||||||||||||||||||||||||||||||||||||||||||||||||||

Tgt 1 + AAACCAACAGGGATTGCCTCAGTAACGGCGAGTGAAGCGGCAACAGCTCAAATTTGAAATCTGGCCTCTTTGGGGTCCGA 80

Qry 81 + GTTGTAATTTGTAGAGGATGTTTCGGGTACCGCCTCGGTTTAAATTTCTTGGAACAGAATGTCAGAGAGGGTGAGAATCC 160

||||||||||||||||||||||||||||||||||||||||||||||||||||||||||||||||||||||||||||||||

Tgt 81 + GTTGTAATTTGTAGAGGATGTTTCGGGTACCGCCTCGGTTTAAATTTCTTGGAACAGAATGTCAGAGAGGGTGAGAATCC 160

Qry 161 + CGTCTTGGACCGGCGGTAGGGGCCCTGTGAAACTCCTTCGACGAGG 206

||||||||||||||||||||||||||||| || ||||||| || ||

Tgt 161 + CGTCTTGGACCGGCGGTAGGGGCCCTGTGGAAATCCTTCGGCGGGG 206

206 cols, 202 ids (98.1%), 0 gaps (0.0%)

Query >Otu24

%Id TLen Target

100% 206 Otu29

100% 206 Otu28

100% 206 Otu23

99% 206 Otu36

99% 206 Otu35

99% 206 Otu32

99% 206 Otu30

99% 206 Otu33

99% 206 Otu38

99% 206 Otu44

99% 206 Otu41

98% 206 Otu63

98% 206 Otu88

Query 206nt >Otu24

Target 206nt >Otu29

Qry 1 + AAACCAACAGGGATTGCCTCAGTAACGGCGAGTGAAGCGGCAACAGCTCAAATTTGAAATCTGGCCTCTTTGGGGTCCGA 80

||||||||||||||||||||||||||||||||||||||||||||||||||||||||||||||||||||||||||||||||

Tgt 1 + AAACCAACAGGGATTGCCTCAGTAACGGCGAGTGAAGCGGCAACAGCTCAAATTTGAAATCTGGCCTCTTTGGGGTCCGA 80

Qry 81 + GTTGTAATTTGTAGAGGATGTTTCGGGTACCGCCTCGGTTTAAATTTCTTGGAACAGAATGTCAGAGAGGGTGAGAATCC 160

||||||||||||||||||||||||||||||||||||||||||||||||||||||||||||||||||||||||||||||||

Tgt 81 + GTTGTAATTTGTAGAGGATGTTTCGGGTACCGCCTCGGTTTAAATTTCTTGGAACAGAATGTCAGAGAGGGTGAGAATCC 160

Qry 161 + CGTCTTGGACCGGCGGTAGGGGCCATGTGAAACTCCTTCGACGAGG 206

||||||||||||||||||||| ||||||||||||||||||||||||

Tgt 161 + CGTCTTGGACCGGCGGTAGGGCCCATGTGAAACTCCTTCGACGAGG 206

206 cols, 205 ids (99.5%), 0 gaps (0.0%)

Query 206nt >Otu24

Target 206nt >Otu28

Qry 1 + AAACCAACAGGGATTGCCTCAGTAACGGCGAGTGAAGCGGCAACAGCTCAAATTTGAAATCTGGCCTCTTTGGGGTCCGA 80

||||||||||||||||||||||||||||||||||||||||||||||||||||||||||||||||||||||||||||||||

Tgt 1 + AAACCAACAGGGATTGCCTCAGTAACGGCGAGTGAAGCGGCAACAGCTCAAATTTGAAATCTGGCCTCTTTGGGGTCCGA 80

Qry 81 + GTTGTAATTTGTAGAGGATGTTTCGGGTACCGCCTCGGTTTAAATTTCTTGGAACAGAATGTCAGAGAGGGTGAGAATCC 160

||||||||||||||||||||||||||||||||||||||||||||||||||||||||||||||||||||||||||||||||

Tgt 81 + GTTGTAATTTGTAGAGGATGTTTCGGGTACCGCCTCGGTTTAAATTTCTTGGAACAGAATGTCAGAGAGGGTGAGAATCC 160

Qry 161 + CGTCTTGGACCGGCGGTAGGGGCCATGTGAAACTCCTTCGACGAGG 206

||||||||||||||||||||||||||||||||||||||||||| ||

Tgt 161 + CGTCTTGGACCGGCGGTAGGGGCCATGTGAAACTCCTTCGACGGGG 206

206 cols, 205 ids (99.5%), 0 gaps (0.0%)

Query 206nt >Otu24

Target 206nt >Otu23

Qry 1 + AAACCAACAGGGATTGCCTCAGTAACGGCGAGTGAAGCGGCAACAGCTCAAATTTGAAATCTGGCCTCTTTGGGGTCCGA 80

||||||||||||||||||||||||||||||||||||||||||||||||||||||||||||||||||||||||||||||||

Tgt 1 + AAACCAACAGGGATTGCCTCAGTAACGGCGAGTGAAGCGGCAACAGCTCAAATTTGAAATCTGGCCTCTTTGGGGTCCGA 80

Qry 81 + GTTGTAATTTGTAGAGGATGTTTCGGGTACCGCCTCGGTTTAAATTTCTTGGAACAGAATGTCAGAGAGGGTGAGAATCC 160

||||||||||||||||||||||||||||||||||||||||||||||||||||||||||||||||||||||||||||||||

Tgt 81 + GTTGTAATTTGTAGAGGATGTTTCGGGTACCGCCTCGGTTTAAATTTCTTGGAACAGAATGTCAGAGAGGGTGAGAATCC 160

Qry 161 + CGTCTTGGACCGGCGGTAGGGGCCATGTGAAACTCCTTCGACGAGG 206

|||||||||||||||||||||||||||||||||||||||||||||

Tgt 161 + CGTCTTGGACCGGCGGTAGGGGCCATGTGAAACTCCTTCGACGAGT 206

206 cols, 205 ids (99.5%), 0 gaps (0.0%)

Query 206nt >Otu24

Target 206nt >Otu36

Qry 1 + AAACCAACAGGGATTGCCTCAGTAACGGCGAGTGAAGCGGCAACAGCTCAAATTTGAAATCTGGCCTCTTTGGGGTCCGA 80

||||||||||||||||||||||||||||||||||||||||||||||||||||||||||||||||||||||||||||||||

Tgt 1 + AAACCAACAGGGATTGCCTCAGTAACGGCGAGTGAAGCGGCAACAGCTCAAATTTGAAATCTGGCCTCTTTGGGGTCCGA 80

Qry 81 + GTTGTAATTTGTAGAGGATGTTTCGGGTACCGCCTCGGTTTAAATTTCTTGGAACAGAATGTCAGAGAGGGTGAGAATCC 160

||||||||||||||||||||||||||||||||||||||||||||||||||||||||||||||||||||||||||||||||

Tgt 81 + GTTGTAATTTGTAGAGGATGTTTCGGGTACCGCCTCGGTTTAAATTTCTTGGAACAGAATGTCAGAGAGGGTGAGAATCC 160

Qry 161 + CGTCTTGGACCGGCGGTAGGGGCCATGTGAAACTCCTTCGACGAGG 206

|||||||||||||||||||||||| |||||||||||||||||| ||

Tgt 161 + CGTCTTGGACCGGCGGTAGGGGCCCTGTGAAACTCCTTCGACGGGG 206

206 cols, 204 ids (99.0%), 0 gaps (0.0%)

Query 206nt >Otu24

Target 206nt >Otu35

Qry 1 + AAACCAACAGGGATTGCCTCAGTAACGGCGAGTGAAGCGGCAACAGCTCAAATTTGAAATCTGGCCTCTTTGGGGTCCGA 80

||||||||||||||||||||||||||||||||||||||||||||||||||||||||||||||||||||||||||||||||

Tgt 1 + AAACCAACAGGGATTGCCTCAGTAACGGCGAGTGAAGCGGCAACAGCTCAAATTTGAAATCTGGCCTCTTTGGGGTCCGA 80

Qry 81 + GTTGTAATTTGTAGAGGATGTTTCGGGTACCGCCTCGGTTTAAATTTCTTGGAACAGAATGTCAGAGAGGGTGAGAATCC 160

||||||||||||||||||||||||||||||||||||||||||||||||||||||||||||||||||||||||||||||||

Tgt 81 + GTTGTAATTTGTAGAGGATGTTTCGGGTACCGCCTCGGTTTAAATTTCTTGGAACAGAATGTCAGAGAGGGTGAGAATCC 160

Qry 161 + CGTCTTGGACCGGCGGTAGGGGCCATGTGAAACTCCTTCGACGAGG 206

||||||||||||||||||||| |||||||||||||||||||||||

Tgt 161 + CGTCTTGGACCGGCGGTAGGGCCCATGTGAAACTCCTTCGACGAGT 206

206 cols, 204 ids (99.0%), 0 gaps (0.0%)

Query 206nt >Otu24

Target 206nt >Otu32

Qry 1 + AAACCAACAGGGATTGCCTCAGTAACGGCGAGTGAAGCGGCAACAGCTCAAATTTGAAATCTGGCCTCTTTGGGGTCCGA 80

||||||||||||||||||||||||||||||||||||||||||||||||||||||||||||||||||||||||||||||||

Tgt 1 + AAACCAACAGGGATTGCCTCAGTAACGGCGAGTGAAGCGGCAACAGCTCAAATTTGAAATCTGGCCTCTTTGGGGTCCGA 80

Qry 81 + GTTGTAATTTGTAGAGGATGTTTCGGGTACCGCCTCGGTTTAAATTTCTTGGAACAGAATGTCAGAGAGGGTGAGAATCC 160

||||||||||||||||||||||||||||||||||||||||||||||||||||||||||||||||||||||||||||||||

Tgt 81 + GTTGTAATTTGTAGAGGATGTTTCGGGTACCGCCTCGGTTTAAATTTCTTGGAACAGAATGTCAGAGAGGGTGAGAATCC 160

Qry 161 + CGTCTTGGACCGGCGGTAGGGGCCATGTGAAACTCCTTCGACGAGG 206

||||||||||||||||||||||||||||| |||||||||||||||

Tgt 161 + CGTCTTGGACCGGCGGTAGGGGCCATGTGGAACTCCTTCGACGAGT 206

206 cols, 204 ids (99.0%), 0 gaps (0.0%)

Query 206nt >Otu24

Target 206nt >Otu30

Qry 1 + AAACCAACAGGGATTGCCTCAGTAACGGCGAGTGAAGCGGCAACAGCTCAAATTTGAAATCTGGCCTCTTTGGGGTCCGA 80

||||||||||||||||||||||||||||||||||||||||||||||||||||||||||||||||||||||||||||||||

Tgt 1 + AAACCAACAGGGATTGCCTCAGTAACGGCGAGTGAAGCGGCAACAGCTCAAATTTGAAATCTGGCCTCTTTGGGGTCCGA 80

Qry 81 + GTTGTAATTTGTAGAGGATGTTTCGGGTACCGCCTCGGTTTAAATTTCTTGGAACAGAATGTCAGAGAGGGTGAGAATCC 160

||||||||||||||||||||||||||||||||||||||||||||||||||||||||||||||||||||||||||||||||

Tgt 81 + GTTGTAATTTGTAGAGGATGTTTCGGGTACCGCCTCGGTTTAAATTTCTTGGAACAGAATGTCAGAGAGGGTGAGAATCC 160

Qry 161 + CGTCTTGGACCGGCGGTAGGGGCCATGTGAAACTCCTTCGACGAGG 206

|||||||||||||||||||||||| ||||||||||||||||||||

Tgt 161 + CGTCTTGGACCGGCGGTAGGGGCCCTGTGAAACTCCTTCGACGAGT 206

206 cols, 204 ids (99.0%), 0 gaps (0.0%)

Query 206nt >Otu24

Target 206nt >Otu33

Qry 1 + AAACCAACAGGGATTGCCTCAGTAACGGCGAGTGAAGCGGCAACAGCTCAAATTTGAAATCTGGCCTCTTTGGGGTCCGA 80

||||||||||||||||||||||||||||||||||||||||||||||||||||||||||||||||||||||||||||||||

Tgt 1 + AAACCAACAGGGATTGCCTCAGTAACGGCGAGTGAAGCGGCAACAGCTCAAATTTGAAATCTGGCCTCTTTGGGGTCCGA 80

Qry 81 + GTTGTAATTTGTAGAGGATGTTTCGGGTACCGCCTCGGTTTAAATTTCTTGGAACAGAATGTCAGAGAGGGTGAGAATCC 160

||||||||||||||||||||||||||||||||||||||||||||||||||||||||||||||||||||||||||||||||

Tgt 81 + GTTGTAATTTGTAGAGGATGTTTCGGGTACCGCCTCGGTTTAAATTTCTTGGAACAGAATGTCAGAGAGGGTGAGAATCC 160

Qry 161 + CGTCTTGGACCGGCGGTAGGGGCCATGTGAAACTCCTTCGACGAGG 206

||||||||||||||||||||||||||||| ||||||||||||| ||

Tgt 161 + CGTCTTGGACCGGCGGTAGGGGCCATGTGGAACTCCTTCGACGGGG 206

206 cols, 204 ids (99.0%), 0 gaps (0.0%)

Query 206nt >Otu24

Target 206nt >Otu38

Qry 1 + AAACCAACAGGGATTGCCTCAGTAACGGCGAGTGAAGCGGCAACAGCTCAAATTTGAAATCTGGCCTCTTTGGGGTCCGA 80

||||||||||||||||||||||||||||||||||||||||||||||||||||||||||||||||||||||||||||||||

Tgt 1 + AAACCAACAGGGATTGCCTCAGTAACGGCGAGTGAAGCGGCAACAGCTCAAATTTGAAATCTGGCCTCTTTGGGGTCCGA 80

Qry 81 + GTTGTAATTTGTAGAGGATGTTTCGGGTACCGCCTCGGTTTAAATTTCTTGGAACAGAATGTCAGAGAGGGTGAGAATCC 160

||||||||||||||||||||||||||||||||||||||||||||||||||||||||||||||||||||||||||||||||

Tgt 81 + GTTGTAATTTGTAGAGGATGTTTCGGGTACCGCCTCGGTTTAAATTTCTTGGAACAGAATGTCAGAGAGGGTGAGAATCC 160

Qry 161 + CGTCTTGGACCGGCGGTAGGGGCCATGTGAAACTCCTTCGACGAGG 206

|||||||||||||||||||||||| |||| ||||||||||||| ||

Tgt 161 + CGTCTTGGACCGGCGGTAGGGGCCCTGTGGAACTCCTTCGACGGGG 206

206 cols, 203 ids (98.5%), 0 gaps (0.0%)

Query 206nt >Otu24

Target 206nt >Otu44

Qry 1 + AAACCAACAGGGATTGCCTCAGTAACGGCGAGTGAAGCGGCAACAGCTCAAATTTGAAATCTGGCCTCTTTGGGGTCCGA 80

||||||||||||||||||||||||||||||||||||||||||||||||||||||||||||||||||||||||||||||||

Tgt 1 + AAACCAACAGGGATTGCCTCAGTAACGGCGAGTGAAGCGGCAACAGCTCAAATTTGAAATCTGGCCTCTTTGGGGTCCGA 80

Qry 81 + GTTGTAATTTGTAGAGGATGTTTCGGGTACCGCCTCGGTTTAAATTTCTTGGAACAGAATGTCAGAGAGGGTGAGAATCC 160

||||||||||||||||||||||||||||||||||||||||||||||||||||||||||||||||||||||||||||||||

Tgt 81 + GTTGTAATTTGTAGAGGATGTTTCGGGTACCGCCTCGGTTTAAATTTCTTGGAACAGAATGTCAGAGAGGGTGAGAATCC 160

Qry 161 + CGTCTTGGACCGGCGGTAGGGGCCATGTGAAACTCCTTCGACGAGG 206

|||||||||||||||||||||||| |||||||||||||||||| |

Tgt 161 + CGTCTTGGACCGGCGGTAGGGGCCCTGTGAAACTCCTTCGACGGGT 206

206 cols, 203 ids (98.5%), 0 gaps (0.0%)

Query 206nt >Otu24

Target 206nt >Otu41

Qry 1 + AAACCAACAGGGATTGCCTCAGTAACGGCGAGTGAAGCGGCAACAGCTCAAATTTGAAATCTGGCCTCTTTGGGGTCCGA 80

||||||||||||||||||||||||||||||||||||||||||||||||||||||||||||||||||||||||||||||||

Tgt 1 + AAACCAACAGGGATTGCCTCAGTAACGGCGAGTGAAGCGGCAACAGCTCAAATTTGAAATCTGGCCTCTTTGGGGTCCGA 80

Qry 81 + GTTGTAATTTGTAGAGGATGTTTCGGGTACCGCCTCGGTTTAAATTTCTTGGAACAGAATGTCAGAGAGGGTGAGAATCC 160

||||||||||||||||||||||||||||||||||||||||||||||||||||||||||||||||||||||||||||||||

Tgt 81 + GTTGTAATTTGTAGAGGATGTTTCGGGTACCGCCTCGGTTTAAATTTCTTGGAACAGAATGTCAGAGAGGGTGAGAATCC 160

Qry 161 + CGTCTTGGACCGGCGGTAGGGGCCATGTGAAACTCCTTCGACGAGG 206

||||||||||||||||||||| || ||||||||||||||||||||

Tgt 161 + CGTCTTGGACCGGCGGTAGGGCCCCTGTGAAACTCCTTCGACGAGT 206

206 cols, 203 ids (98.5%), 0 gaps (0.0%)

Query 206nt >Otu24

Target 206nt >Otu63

Qry 1 + AAACCAACAGGGATTGCCTCAGTAACGGCGAGTGAAGCGGCAACAGCTCAAATTTGAAATCTGGCCTCTTTGGGGTCCGA 80

||||||||||||||||||||||||||||||||||||||||||||||||||||||||||||||||||||||||||||||||

Tgt 1 + AAACCAACAGGGATTGCCTCAGTAACGGCGAGTGAAGCGGCAACAGCTCAAATTTGAAATCTGGCCTCTTTGGGGTCCGA 80

Qry 81 + GTTGTAATTTGTAGAGGATGTTTCGGGTACCGCCTCGGTTTAAATTTCTTGGAACAGAATGTCAGAGAGGGTGAGAATCC 160

||||||||||||||||||||||||||||||||||||||||||||||||||||||||||||||||||||||||||||||||

Tgt 81 + GTTGTAATTTGTAGAGGATGTTTCGGGTACCGCCTCGGTTTAAATTTCTTGGAACAGAATGTCAGAGAGGGTGAGAATCC 160

Qry 161 + CGTCTTGGACCGGCGGTAGGGGCCATGTGAAACTCCTTCGACGAGG 206

|||||||||||||||||||||||| |||| |||||||||| || ||

Tgt 161 + CGTCTTGGACCGGCGGTAGGGGCCCTGTGGAACTCCTTCGGCGGGG 206

206 cols, 202 ids (98.1%), 0 gaps (0.0%)

Query 206nt >Otu24

Target 206nt >Otu88

Qry 1 + AAACCAACAGGGATTGCCTCAGTAACGGCGAGTGAAGCGGCAACAGCTCAAATTTGAAATCTGGCCTCTTTGGGGTCCGA 80

||||||||||||||||||||||||||||||||||||||||||||||||||||||||||||||||||||||||||||||||

Tgt 1 + AAACCAACAGGGATTGCCTCAGTAACGGCGAGTGAAGCGGCAACAGCTCAAATTTGAAATCTGGCCTCTTTGGGGTCCGA 80

Qry 81 + GTTGTAATTTGTAGAGGATGTTTCGGGTACCGCCTCGGTTTAAATTTCTTGGAACAGAATGTCAGAGAGGGTGAGAATCC 160

||||||||||||||||||||||||||||||||||||||||||||||||||||||||||||||||||||||||||||||||

Tgt 81 + GTTGTAATTTGTAGAGGATGTTTCGGGTACCGCCTCGGTTTAAATTTCTTGGAACAGAATGTCAGAGAGGGTGAGAATCC 160

Qry 161 + CGTCTTGGACCGGCGGTAGGGGCCATGTGAAACTCCTTCGACGAGG 206

|||||||||||||||||||||||| |||| || ||||||| || ||

Tgt 161 + CGTCTTGGACCGGCGGTAGGGGCCCTGTGGAAATCCTTCGGCGGGG 206

206 cols, 201 ids (97.6%), 0 gaps (0.0%)

Query >Otu23

%Id TLen Target

100% 206 Otu35

100% 206 Otu32

100% 206 Otu30

99% 206 Otu41

99% 206 Otu29

99% 206 Otu28

99% 206 Otu44

99% 206 Otu33

99% 206 Otu36

98% 206 Otu38

98% 206 Otu63

97% 206 Otu88

Query 206nt >Otu23

Target 206nt >Otu35

Qry 1 + AAACCAACAGGGATTGCCTCAGTAACGGCGAGTGAAGCGGCAACAGCTCAAATTTGAAATCTGGCCTCTTTGGGGTCCGA 80

||||||||||||||||||||||||||||||||||||||||||||||||||||||||||||||||||||||||||||||||

Tgt 1 + AAACCAACAGGGATTGCCTCAGTAACGGCGAGTGAAGCGGCAACAGCTCAAATTTGAAATCTGGCCTCTTTGGGGTCCGA 80

Qry 81 + GTTGTAATTTGTAGAGGATGTTTCGGGTACCGCCTCGGTTTAAATTTCTTGGAACAGAATGTCAGAGAGGGTGAGAATCC 160

||||||||||||||||||||||||||||||||||||||||||||||||||||||||||||||||||||||||||||||||

Tgt 81 + GTTGTAATTTGTAGAGGATGTTTCGGGTACCGCCTCGGTTTAAATTTCTTGGAACAGAATGTCAGAGAGGGTGAGAATCC 160

Qry 161 + CGTCTTGGACCGGCGGTAGGGGCCATGTGAAACTCCTTCGACGAGT 206

||||||||||||||||||||| ||||||||||||||||||||||||

Tgt 161 + CGTCTTGGACCGGCGGTAGGGCCCATGTGAAACTCCTTCGACGAGT 206

206 cols, 205 ids (99.5%), 0 gaps (0.0%)

Query 206nt >Otu23

Target 206nt >Otu32

Qry 1 + AAACCAACAGGGATTGCCTCAGTAACGGCGAGTGAAGCGGCAACAGCTCAAATTTGAAATCTGGCCTCTTTGGGGTCCGA 80

||||||||||||||||||||||||||||||||||||||||||||||||||||||||||||||||||||||||||||||||

Tgt 1 + AAACCAACAGGGATTGCCTCAGTAACGGCGAGTGAAGCGGCAACAGCTCAAATTTGAAATCTGGCCTCTTTGGGGTCCGA 80

Qry 81 + GTTGTAATTTGTAGAGGATGTTTCGGGTACCGCCTCGGTTTAAATTTCTTGGAACAGAATGTCAGAGAGGGTGAGAATCC 160

||||||||||||||||||||||||||||||||||||||||||||||||||||||||||||||||||||||||||||||||

Tgt 81 + GTTGTAATTTGTAGAGGATGTTTCGGGTACCGCCTCGGTTTAAATTTCTTGGAACAGAATGTCAGAGAGGGTGAGAATCC 160

Qry 161 + CGTCTTGGACCGGCGGTAGGGGCCATGTGAAACTCCTTCGACGAGT 206

||||||||||||||||||||||||||||| ||||||||||||||||

Tgt 161 + CGTCTTGGACCGGCGGTAGGGGCCATGTGGAACTCCTTCGACGAGT 206

206 cols, 205 ids (99.5%), 0 gaps (0.0%)

Query 206nt >Otu23

Target 206nt >Otu30

Qry 1 + AAACCAACAGGGATTGCCTCAGTAACGGCGAGTGAAGCGGCAACAGCTCAAATTTGAAATCTGGCCTCTTTGGGGTCCGA 80

||||||||||||||||||||||||||||||||||||||||||||||||||||||||||||||||||||||||||||||||

Tgt 1 + AAACCAACAGGGATTGCCTCAGTAACGGCGAGTGAAGCGGCAACAGCTCAAATTTGAAATCTGGCCTCTTTGGGGTCCGA 80

Qry 81 + GTTGTAATTTGTAGAGGATGTTTCGGGTACCGCCTCGGTTTAAATTTCTTGGAACAGAATGTCAGAGAGGGTGAGAATCC 160

||||||||||||||||||||||||||||||||||||||||||||||||||||||||||||||||||||||||||||||||

Tgt 81 + GTTGTAATTTGTAGAGGATGTTTCGGGTACCGCCTCGGTTTAAATTTCTTGGAACAGAATGTCAGAGAGGGTGAGAATCC 160

Qry 161 + CGTCTTGGACCGGCGGTAGGGGCCATGTGAAACTCCTTCGACGAGT 206

|||||||||||||||||||||||| |||||||||||||||||||||

Tgt 161 + CGTCTTGGACCGGCGGTAGGGGCCCTGTGAAACTCCTTCGACGAGT 206

206 cols, 205 ids (99.5%), 0 gaps (0.0%)

Query 206nt >Otu23

Target 206nt >Otu41

Qry 1 + AAACCAACAGGGATTGCCTCAGTAACGGCGAGTGAAGCGGCAACAGCTCAAATTTGAAATCTGGCCTCTTTGGGGTCCGA 80

||||||||||||||||||||||||||||||||||||||||||||||||||||||||||||||||||||||||||||||||

Tgt 1 + AAACCAACAGGGATTGCCTCAGTAACGGCGAGTGAAGCGGCAACAGCTCAAATTTGAAATCTGGCCTCTTTGGGGTCCGA 80

Qry 81 + GTTGTAATTTGTAGAGGATGTTTCGGGTACCGCCTCGGTTTAAATTTCTTGGAACAGAATGTCAGAGAGGGTGAGAATCC 160

||||||||||||||||||||||||||||||||||||||||||||||||||||||||||||||||||||||||||||||||

Tgt 81 + GTTGTAATTTGTAGAGGATGTTTCGGGTACCGCCTCGGTTTAAATTTCTTGGAACAGAATGTCAGAGAGGGTGAGAATCC 160

Qry 161 + CGTCTTGGACCGGCGGTAGGGGCCATGTGAAACTCCTTCGACGAGT 206

||||||||||||||||||||| || |||||||||||||||||||||

Tgt 161 + CGTCTTGGACCGGCGGTAGGGCCCCTGTGAAACTCCTTCGACGAGT 206

206 cols, 204 ids (99.0%), 0 gaps (0.0%)

Query 206nt >Otu23

Target 206nt >Otu29

Qry 1 + AAACCAACAGGGATTGCCTCAGTAACGGCGAGTGAAGCGGCAACAGCTCAAATTTGAAATCTGGCCTCTTTGGGGTCCGA 80

||||||||||||||||||||||||||||||||||||||||||||||||||||||||||||||||||||||||||||||||

Tgt 1 + AAACCAACAGGGATTGCCTCAGTAACGGCGAGTGAAGCGGCAACAGCTCAAATTTGAAATCTGGCCTCTTTGGGGTCCGA 80

Qry 81 + GTTGTAATTTGTAGAGGATGTTTCGGGTACCGCCTCGGTTTAAATTTCTTGGAACAGAATGTCAGAGAGGGTGAGAATCC 160

||||||||||||||||||||||||||||||||||||||||||||||||||||||||||||||||||||||||||||||||

Tgt 81 + GTTGTAATTTGTAGAGGATGTTTCGGGTACCGCCTCGGTTTAAATTTCTTGGAACAGAATGTCAGAGAGGGTGAGAATCC 160

Qry 161 + CGTCTTGGACCGGCGGTAGGGGCCATGTGAAACTCCTTCGACGAGT 206

||||||||||||||||||||| |||||||||||||||||||||||

Tgt 161 + CGTCTTGGACCGGCGGTAGGGCCCATGTGAAACTCCTTCGACGAGG 206

206 cols, 204 ids (99.0%), 0 gaps (0.0%)

Query 206nt >Otu23

Target 206nt >Otu28

Qry 1 + AAACCAACAGGGATTGCCTCAGTAACGGCGAGTGAAGCGGCAACAGCTCAAATTTGAAATCTGGCCTCTTTGGGGTCCGA 80

||||||||||||||||||||||||||||||||||||||||||||||||||||||||||||||||||||||||||||||||

Tgt 1 + AAACCAACAGGGATTGCCTCAGTAACGGCGAGTGAAGCGGCAACAGCTCAAATTTGAAATCTGGCCTCTTTGGGGTCCGA 80

Qry 81 + GTTGTAATTTGTAGAGGATGTTTCGGGTACCGCCTCGGTTTAAATTTCTTGGAACAGAATGTCAGAGAGGGTGAGAATCC 160

||||||||||||||||||||||||||||||||||||||||||||||||||||||||||||||||||||||||||||||||

Tgt 81 + GTTGTAATTTGTAGAGGATGTTTCGGGTACCGCCTCGGTTTAAATTTCTTGGAACAGAATGTCAGAGAGGGTGAGAATCC 160

Qry 161 + CGTCTTGGACCGGCGGTAGGGGCCATGTGAAACTCCTTCGACGAGT 206

||||||||||||||||||||||||||||||||||||||||||| |

Tgt 161 + CGTCTTGGACCGGCGGTAGGGGCCATGTGAAACTCCTTCGACGGGG 206

206 cols, 204 ids (99.0%), 0 gaps (0.0%)

Query 206nt >Otu23

Target 206nt >Otu44

Qry 1 + AAACCAACAGGGATTGCCTCAGTAACGGCGAGTGAAGCGGCAACAGCTCAAATTTGAAATCTGGCCTCTTTGGGGTCCGA 80

||||||||||||||||||||||||||||||||||||||||||||||||||||||||||||||||||||||||||||||||

Tgt 1 + AAACCAACAGGGATTGCCTCAGTAACGGCGAGTGAAGCGGCAACAGCTCAAATTTGAAATCTGGCCTCTTTGGGGTCCGA 80

Qry 81 + GTTGTAATTTGTAGAGGATGTTTCGGGTACCGCCTCGGTTTAAATTTCTTGGAACAGAATGTCAGAGAGGGTGAGAATCC 160

||||||||||||||||||||||||||||||||||||||||||||||||||||||||||||||||||||||||||||||||

Tgt 81 + GTTGTAATTTGTAGAGGATGTTTCGGGTACCGCCTCGGTTTAAATTTCTTGGAACAGAATGTCAGAGAGGGTGAGAATCC 160

Qry 161 + CGTCTTGGACCGGCGGTAGGGGCCATGTGAAACTCCTTCGACGAGT 206

|||||||||||||||||||||||| |||||||||||||||||| ||

Tgt 161 + CGTCTTGGACCGGCGGTAGGGGCCCTGTGAAACTCCTTCGACGGGT 206

206 cols, 204 ids (99.0%), 0 gaps (0.0%)

Query 206nt >Otu23

Target 206nt >Otu33

Qry 1 + AAACCAACAGGGATTGCCTCAGTAACGGCGAGTGAAGCGGCAACAGCTCAAATTTGAAATCTGGCCTCTTTGGGGTCCGA 80

||||||||||||||||||||||||||||||||||||||||||||||||||||||||||||||||||||||||||||||||

Tgt 1 + AAACCAACAGGGATTGCCTCAGTAACGGCGAGTGAAGCGGCAACAGCTCAAATTTGAAATCTGGCCTCTTTGGGGTCCGA 80

Qry 81 + GTTGTAATTTGTAGAGGATGTTTCGGGTACCGCCTCGGTTTAAATTTCTTGGAACAGAATGTCAGAGAGGGTGAGAATCC 160

||||||||||||||||||||||||||||||||||||||||||||||||||||||||||||||||||||||||||||||||

Tgt 81 + GTTGTAATTTGTAGAGGATGTTTCGGGTACCGCCTCGGTTTAAATTTCTTGGAACAGAATGTCAGAGAGGGTGAGAATCC 160

Qry 161 + CGTCTTGGACCGGCGGTAGGGGCCATGTGAAACTCCTTCGACGAGT 206

||||||||||||||||||||||||||||| ||||||||||||| |

Tgt 161 + CGTCTTGGACCGGCGGTAGGGGCCATGTGGAACTCCTTCGACGGGG 206

206 cols, 203 ids (98.5%), 0 gaps (0.0%)

Query 206nt >Otu23

Target 206nt >Otu36

Qry 1 + AAACCAACAGGGATTGCCTCAGTAACGGCGAGTGAAGCGGCAACAGCTCAAATTTGAAATCTGGCCTCTTTGGGGTCCGA 80

||||||||||||||||||||||||||||||||||||||||||||||||||||||||||||||||||||||||||||||||

Tgt 1 + AAACCAACAGGGATTGCCTCAGTAACGGCGAGTGAAGCGGCAACAGCTCAAATTTGAAATCTGGCCTCTTTGGGGTCCGA 80

Qry 81 + GTTGTAATTTGTAGAGGATGTTTCGGGTACCGCCTCGGTTTAAATTTCTTGGAACAGAATGTCAGAGAGGGTGAGAATCC 160

||||||||||||||||||||||||||||||||||||||||||||||||||||||||||||||||||||||||||||||||

Tgt 81 + GTTGTAATTTGTAGAGGATGTTTCGGGTACCGCCTCGGTTTAAATTTCTTGGAACAGAATGTCAGAGAGGGTGAGAATCC 160

Qry 161 + CGTCTTGGACCGGCGGTAGGGGCCATGTGAAACTCCTTCGACGAGT 206

|||||||||||||||||||||||| |||||||||||||||||| |

Tgt 161 + CGTCTTGGACCGGCGGTAGGGGCCCTGTGAAACTCCTTCGACGGGG 206

206 cols, 203 ids (98.5%), 0 gaps (0.0%)

Query 206nt >Otu23

Target 206nt >Otu38

Qry 1 + AAACCAACAGGGATTGCCTCAGTAACGGCGAGTGAAGCGGCAACAGCTCAAATTTGAAATCTGGCCTCTTTGGGGTCCGA 80

||||||||||||||||||||||||||||||||||||||||||||||||||||||||||||||||||||||||||||||||

Tgt 1 + AAACCAACAGGGATTGCCTCAGTAACGGCGAGTGAAGCGGCAACAGCTCAAATTTGAAATCTGGCCTCTTTGGGGTCCGA 80

Qry 81 + GTTGTAATTTGTAGAGGATGTTTCGGGTACCGCCTCGGTTTAAATTTCTTGGAACAGAATGTCAGAGAGGGTGAGAATCC 160

||||||||||||||||||||||||||||||||||||||||||||||||||||||||||||||||||||||||||||||||

Tgt 81 + GTTGTAATTTGTAGAGGATGTTTCGGGTACCGCCTCGGTTTAAATTTCTTGGAACAGAATGTCAGAGAGGGTGAGAATCC 160

Qry 161 + CGTCTTGGACCGGCGGTAGGGGCCATGTGAAACTCCTTCGACGAGT 206

|||||||||||||||||||||||| |||| ||||||||||||| |

Tgt 161 + CGTCTTGGACCGGCGGTAGGGGCCCTGTGGAACTCCTTCGACGGGG 206

206 cols, 202 ids (98.1%), 0 gaps (0.0%)

Query 206nt >Otu23

Target 206nt >Otu63

Qry 1 + AAACCAACAGGGATTGCCTCAGTAACGGCGAGTGAAGCGGCAACAGCTCAAATTTGAAATCTGGCCTCTTTGGGGTCCGA 80

||||||||||||||||||||||||||||||||||||||||||||||||||||||||||||||||||||||||||||||||

Tgt 1 + AAACCAACAGGGATTGCCTCAGTAACGGCGAGTGAAGCGGCAACAGCTCAAATTTGAAATCTGGCCTCTTTGGGGTCCGA 80

Qry 81 + GTTGTAATTTGTAGAGGATGTTTCGGGTACCGCCTCGGTTTAAATTTCTTGGAACAGAATGTCAGAGAGGGTGAGAATCC 160

||||||||||||||||||||||||||||||||||||||||||||||||||||||||||||||||||||||||||||||||

Tgt 81 + GTTGTAATTTGTAGAGGATGTTTCGGGTACCGCCTCGGTTTAAATTTCTTGGAACAGAATGTCAGAGAGGGTGAGAATCC 160

Qry 161 + CGTCTTGGACCGGCGGTAGGGGCCATGTGAAACTCCTTCGACGAGT 206

|||||||||||||||||||||||| |||| |||||||||| || |

Tgt 161 + CGTCTTGGACCGGCGGTAGGGGCCCTGTGGAACTCCTTCGGCGGGG 206

206 cols, 201 ids (97.6%), 0 gaps (0.0%)

Query 206nt >Otu23

Target 206nt >Otu88

Qry 1 + AAACCAACAGGGATTGCCTCAGTAACGGCGAGTGAAGCGGCAACAGCTCAAATTTGAAATCTGGCCTCTTTGGGGTCCGA 80

||||||||||||||||||||||||||||||||||||||||||||||||||||||||||||||||||||||||||||||||

Tgt 1 + AAACCAACAGGGATTGCCTCAGTAACGGCGAGTGAAGCGGCAACAGCTCAAATTTGAAATCTGGCCTCTTTGGGGTCCGA 80

Qry 81 + GTTGTAATTTGTAGAGGATGTTTCGGGTACCGCCTCGGTTTAAATTTCTTGGAACAGAATGTCAGAGAGGGTGAGAATCC 160

||||||||||||||||||||||||||||||||||||||||||||||||||||||||||||||||||||||||||||||||

Tgt 81 + GTTGTAATTTGTAGAGGATGTTTCGGGTACCGCCTCGGTTTAAATTTCTTGGAACAGAATGTCAGAGAGGGTGAGAATCC 160

Qry 161 + CGTCTTGGACCGGCGGTAGGGGCCATGTGAAACTCCTTCGACGAGT 206

|||||||||||||||||||||||| |||| || ||||||| || |

Tgt 161 + CGTCTTGGACCGGCGGTAGGGGCCCTGTGGAAATCCTTCGGCGGGG 206

206 cols, 200 ids (97.1%), 0 gaps (0.0%)

Query >Otu38

%Id TLen Target

100% 206 Otu36

100% 206 Otu63

100% 206 Otu33

99% 206 Otu44

99% 206 Otu28

99% 206 Otu88

99% 206 Otu32

99% 206 Otu30

98% 206 Otu29

98% 206 Otu41

98% 206 Otu35

Query 206nt >Otu38

Target 206nt >Otu36

Qry 1 + AAACCAACAGGGATTGCCTCAGTAACGGCGAGTGAAGCGGCAACAGCTCAAATTTGAAATCTGGCCTCTTTGGGGTCCGA 80

||||||||||||||||||||||||||||||||||||||||||||||||||||||||||||||||||||||||||||||||

Tgt 1 + AAACCAACAGGGATTGCCTCAGTAACGGCGAGTGAAGCGGCAACAGCTCAAATTTGAAATCTGGCCTCTTTGGGGTCCGA 80

Qry 81 + GTTGTAATTTGTAGAGGATGTTTCGGGTACCGCCTCGGTTTAAATTTCTTGGAACAGAATGTCAGAGAGGGTGAGAATCC 160

||||||||||||||||||||||||||||||||||||||||||||||||||||||||||||||||||||||||||||||||

Tgt 81 + GTTGTAATTTGTAGAGGATGTTTCGGGTACCGCCTCGGTTTAAATTTCTTGGAACAGAATGTCAGAGAGGGTGAGAATCC 160

Qry 161 + CGTCTTGGACCGGCGGTAGGGGCCCTGTGGAACTCCTTCGACGGGG 206

||||||||||||||||||||||||||||| ||||||||||||||||

Tgt 161 + CGTCTTGGACCGGCGGTAGGGGCCCTGTGAAACTCCTTCGACGGGG 206

206 cols, 205 ids (99.5%), 0 gaps (0.0%)

Query 206nt >Otu38

Target 206nt >Otu63

Qry 1 + AAACCAACAGGGATTGCCTCAGTAACGGCGAGTGAAGCGGCAACAGCTCAAATTTGAAATCTGGCCTCTTTGGGGTCCGA 80

||||||||||||||||||||||||||||||||||||||||||||||||||||||||||||||||||||||||||||||||

Tgt 1 + AAACCAACAGGGATTGCCTCAGTAACGGCGAGTGAAGCGGCAACAGCTCAAATTTGAAATCTGGCCTCTTTGGGGTCCGA 80

Qry 81 + GTTGTAATTTGTAGAGGATGTTTCGGGTACCGCCTCGGTTTAAATTTCTTGGAACAGAATGTCAGAGAGGGTGAGAATCC 160

||||||||||||||||||||||||||||||||||||||||||||||||||||||||||||||||||||||||||||||||

Tgt 81 + GTTGTAATTTGTAGAGGATGTTTCGGGTACCGCCTCGGTTTAAATTTCTTGGAACAGAATGTCAGAGAGGGTGAGAATCC 160

Qry 161 + CGTCTTGGACCGGCGGTAGGGGCCCTGTGGAACTCCTTCGACGGGG 206

|||||||||||||||||||||||||||||||||||||||| |||||

Tgt 161 + CGTCTTGGACCGGCGGTAGGGGCCCTGTGGAACTCCTTCGGCGGGG 206

206 cols, 205 ids (99.5%), 0 gaps (0.0%)

Query 206nt >Otu38

Target 206nt >Otu33

Qry 1 + AAACCAACAGGGATTGCCTCAGTAACGGCGAGTGAAGCGGCAACAGCTCAAATTTGAAATCTGGCCTCTTTGGGGTCCGA 80

||||||||||||||||||||||||||||||||||||||||||||||||||||||||||||||||||||||||||||||||

Tgt 1 + AAACCAACAGGGATTGCCTCAGTAACGGCGAGTGAAGCGGCAACAGCTCAAATTTGAAATCTGGCCTCTTTGGGGTCCGA 80

Qry 81 + GTTGTAATTTGTAGAGGATGTTTCGGGTACCGCCTCGGTTTAAATTTCTTGGAACAGAATGTCAGAGAGGGTGAGAATCC 160

||||||||||||||||||||||||||||||||||||||||||||||||||||||||||||||||||||||||||||||||

Tgt 81 + GTTGTAATTTGTAGAGGATGTTTCGGGTACCGCCTCGGTTTAAATTTCTTGGAACAGAATGTCAGAGAGGGTGAGAATCC 160

Qry 161 + CGTCTTGGACCGGCGGTAGGGGCCCTGTGGAACTCCTTCGACGGGG 206

|||||||||||||||||||||||| |||||||||||||||||||||

Tgt 161 + CGTCTTGGACCGGCGGTAGGGGCCATGTGGAACTCCTTCGACGGGG 206

206 cols, 205 ids (99.5%), 0 gaps (0.0%)

Query 206nt >Otu38

Target 206nt >Otu44

Qry 1 + AAACCAACAGGGATTGCCTCAGTAACGGCGAGTGAAGCGGCAACAGCTCAAATTTGAAATCTGGCCTCTTTGGGGTCCGA 80

||||||||||||||||||||||||||||||||||||||||||||||||||||||||||||||||||||||||||||||||

Tgt 1 + AAACCAACAGGGATTGCCTCAGTAACGGCGAGTGAAGCGGCAACAGCTCAAATTTGAAATCTGGCCTCTTTGGGGTCCGA 80

Qry 81 + GTTGTAATTTGTAGAGGATGTTTCGGGTACCGCCTCGGTTTAAATTTCTTGGAACAGAATGTCAGAGAGGGTGAGAATCC 160

||||||||||||||||||||||||||||||||||||||||||||||||||||||||||||||||||||||||||||||||

Tgt 81 + GTTGTAATTTGTAGAGGATGTTTCGGGTACCGCCTCGGTTTAAATTTCTTGGAACAGAATGTCAGAGAGGGTGAGAATCC 160

Qry 161 + CGTCTTGGACCGGCGGTAGGGGCCCTGTGGAACTCCTTCGACGGGG 206

||||||||||||||||||||||||||||| |||||||||||||||

Tgt 161 + CGTCTTGGACCGGCGGTAGGGGCCCTGTGAAACTCCTTCGACGGGT 206

206 cols, 204 ids (99.0%), 0 gaps (0.0%)

Query 206nt >Otu38

Target 206nt >Otu28

Qry 1 + AAACCAACAGGGATTGCCTCAGTAACGGCGAGTGAAGCGGCAACAGCTCAAATTTGAAATCTGGCCTCTTTGGGGTCCGA 80

||||||||||||||||||||||||||||||||||||||||||||||||||||||||||||||||||||||||||||||||

Tgt 1 + AAACCAACAGGGATTGCCTCAGTAACGGCGAGTGAAGCGGCAACAGCTCAAATTTGAAATCTGGCCTCTTTGGGGTCCGA 80

Qry 81 + GTTGTAATTTGTAGAGGATGTTTCGGGTACCGCCTCGGTTTAAATTTCTTGGAACAGAATGTCAGAGAGGGTGAGAATCC 160

||||||||||||||||||||||||||||||||||||||||||||||||||||||||||||||||||||||||||||||||

Tgt 81 + GTTGTAATTTGTAGAGGATGTTTCGGGTACCGCCTCGGTTTAAATTTCTTGGAACAGAATGTCAGAGAGGGTGAGAATCC 160

Qry 161 + CGTCTTGGACCGGCGGTAGGGGCCCTGTGGAACTCCTTCGACGGGG 206

|||||||||||||||||||||||| |||| ||||||||||||||||

Tgt 161 + CGTCTTGGACCGGCGGTAGGGGCCATGTGAAACTCCTTCGACGGGG 206

206 cols, 204 ids (99.0%), 0 gaps (0.0%)

Query 206nt >Otu38

Target 206nt >Otu88

Qry 1 + AAACCAACAGGGATTGCCTCAGTAACGGCGAGTGAAGCGGCAACAGCTCAAATTTGAAATCTGGCCTCTTTGGGGTCCGA 80

||||||||||||||||||||||||||||||||||||||||||||||||||||||||||||||||||||||||||||||||

Tgt 1 + AAACCAACAGGGATTGCCTCAGTAACGGCGAGTGAAGCGGCAACAGCTCAAATTTGAAATCTGGCCTCTTTGGGGTCCGA 80

Qry 81 + GTTGTAATTTGTAGAGGATGTTTCGGGTACCGCCTCGGTTTAAATTTCTTGGAACAGAATGTCAGAGAGGGTGAGAATCC 160

||||||||||||||||||||||||||||||||||||||||||||||||||||||||||||||||||||||||||||||||

Tgt 81 + GTTGTAATTTGTAGAGGATGTTTCGGGTACCGCCTCGGTTTAAATTTCTTGGAACAGAATGTCAGAGAGGGTGAGAATCC 160

Qry 161 + CGTCTTGGACCGGCGGTAGGGGCCCTGTGGAACTCCTTCGACGGGG 206

|||||||||||||||||||||||||||||||| ||||||| |||||

Tgt 161 + CGTCTTGGACCGGCGGTAGGGGCCCTGTGGAAATCCTTCGGCGGGG 206

206 cols, 204 ids (99.0%), 0 gaps (0.0%)

Query 206nt >Otu38

Target 206nt >Otu32

Qry 1 + AAACCAACAGGGATTGCCTCAGTAACGGCGAGTGAAGCGGCAACAGCTCAAATTTGAAATCTGGCCTCTTTGGGGTCCGA 80

||||||||||||||||||||||||||||||||||||||||||||||||||||||||||||||||||||||||||||||||

Tgt 1 + AAACCAACAGGGATTGCCTCAGTAACGGCGAGTGAAGCGGCAACAGCTCAAATTTGAAATCTGGCCTCTTTGGGGTCCGA 80

Qry 81 + GTTGTAATTTGTAGAGGATGTTTCGGGTACCGCCTCGGTTTAAATTTCTTGGAACAGAATGTCAGAGAGGGTGAGAATCC 160

||||||||||||||||||||||||||||||||||||||||||||||||||||||||||||||||||||||||||||||||

Tgt 81 + GTTGTAATTTGTAGAGGATGTTTCGGGTACCGCCTCGGTTTAAATTTCTTGGAACAGAATGTCAGAGAGGGTGAGAATCC 160

Qry 161 + CGTCTTGGACCGGCGGTAGGGGCCCTGTGGAACTCCTTCGACGGGG 206

|||||||||||||||||||||||| |||||||||||||||||| |

Tgt 161 + CGTCTTGGACCGGCGGTAGGGGCCATGTGGAACTCCTTCGACGAGT 206

206 cols, 203 ids (98.5%), 0 gaps (0.0%)

Query 206nt >Otu38

Target 206nt >Otu30

Qry 1 + AAACCAACAGGGATTGCCTCAGTAACGGCGAGTGAAGCGGCAACAGCTCAAATTTGAAATCTGGCCTCTTTGGGGTCCGA 80

||||||||||||||||||||||||||||||||||||||||||||||||||||||||||||||||||||||||||||||||

Tgt 1 + AAACCAACAGGGATTGCCTCAGTAACGGCGAGTGAAGCGGCAACAGCTCAAATTTGAAATCTGGCCTCTTTGGGGTCCGA 80

Qry 81 + GTTGTAATTTGTAGAGGATGTTTCGGGTACCGCCTCGGTTTAAATTTCTTGGAACAGAATGTCAGAGAGGGTGAGAATCC 160

||||||||||||||||||||||||||||||||||||||||||||||||||||||||||||||||||||||||||||||||

Tgt 81 + GTTGTAATTTGTAGAGGATGTTTCGGGTACCGCCTCGGTTTAAATTTCTTGGAACAGAATGTCAGAGAGGGTGAGAATCC 160

Qry 161 + CGTCTTGGACCGGCGGTAGGGGCCCTGTGGAACTCCTTCGACGGGG 206

||||||||||||||||||||||||||||| ||||||||||||| |

Tgt 161 + CGTCTTGGACCGGCGGTAGGGGCCCTGTGAAACTCCTTCGACGAGT 206

206 cols, 203 ids (98.5%), 0 gaps (0.0%)

Query 206nt >Otu38

Target 206nt >Otu29

Qry 1 + AAACCAACAGGGATTGCCTCAGTAACGGCGAGTGAAGCGGCAACAGCTCAAATTTGAAATCTGGCCTCTTTGGGGTCCGA 80

||||||||||||||||||||||||||||||||||||||||||||||||||||||||||||||||||||||||||||||||

Tgt 1 + AAACCAACAGGGATTGCCTCAGTAACGGCGAGTGAAGCGGCAACAGCTCAAATTTGAAATCTGGCCTCTTTGGGGTCCGA 80

Qry 81 + GTTGTAATTTGTAGAGGATGTTTCGGGTACCGCCTCGGTTTAAATTTCTTGGAACAGAATGTCAGAGAGGGTGAGAATCC 160

||||||||||||||||||||||||||||||||||||||||||||||||||||||||||||||||||||||||||||||||

Tgt 81 + GTTGTAATTTGTAGAGGATGTTTCGGGTACCGCCTCGGTTTAAATTTCTTGGAACAGAATGTCAGAGAGGGTGAGAATCC 160

Qry 161 + CGTCTTGGACCGGCGGTAGGGGCCCTGTGGAACTCCTTCGACGGGG 206

||||||||||||||||||||| || |||| ||||||||||||| ||

Tgt 161 + CGTCTTGGACCGGCGGTAGGGCCCATGTGAAACTCCTTCGACGAGG 206

206 cols, 202 ids (98.1%), 0 gaps (0.0%)

Query 206nt >Otu38

Target 206nt >Otu41

Qry 1 + AAACCAACAGGGATTGCCTCAGTAACGGCGAGTGAAGCGGCAACAGCTCAAATTTGAAATCTGGCCTCTTTGGGGTCCGA 80

||||||||||||||||||||||||||||||||||||||||||||||||||||||||||||||||||||||||||||||||

Tgt 1 + AAACCAACAGGGATTGCCTCAGTAACGGCGAGTGAAGCGGCAACAGCTCAAATTTGAAATCTGGCCTCTTTGGGGTCCGA 80

Qry 81 + GTTGTAATTTGTAGAGGATGTTTCGGGTACCGCCTCGGTTTAAATTTCTTGGAACAGAATGTCAGAGAGGGTGAGAATCC 160

||||||||||||||||||||||||||||||||||||||||||||||||||||||||||||||||||||||||||||||||

Tgt 81 + GTTGTAATTTGTAGAGGATGTTTCGGGTACCGCCTCGGTTTAAATTTCTTGGAACAGAATGTCAGAGAGGGTGAGAATCC 160

Qry 161 + CGTCTTGGACCGGCGGTAGGGGCCCTGTGGAACTCCTTCGACGGGG 206

||||||||||||||||||||| ||||||| ||||||||||||| |

Tgt 161 + CGTCTTGGACCGGCGGTAGGGCCCCTGTGAAACTCCTTCGACGAGT 206

206 cols, 202 ids (98.1%), 0 gaps (0.0%)

Query 206nt >Otu38

Target 206nt >Otu35

Qry 1 + AAACCAACAGGGATTGCCTCAGTAACGGCGAGTGAAGCGGCAACAGCTCAAATTTGAAATCTGGCCTCTTTGGGGTCCGA 80

||||||||||||||||||||||||||||||||||||||||||||||||||||||||||||||||||||||||||||||||

Tgt 1 + AAACCAACAGGGATTGCCTCAGTAACGGCGAGTGAAGCGGCAACAGCTCAAATTTGAAATCTGGCCTCTTTGGGGTCCGA 80

Qry 81 + GTTGTAATTTGTAGAGGATGTTTCGGGTACCGCCTCGGTTTAAATTTCTTGGAACAGAATGTCAGAGAGGGTGAGAATCC 160

||||||||||||||||||||||||||||||||||||||||||||||||||||||||||||||||||||||||||||||||

Tgt 81 + GTTGTAATTTGTAGAGGATGTTTCGGGTACCGCCTCGGTTTAAATTTCTTGGAACAGAATGTCAGAGAGGGTGAGAATCC 160

Qry 161 + CGTCTTGGACCGGCGGTAGGGGCCCTGTGGAACTCCTTCGACGGGG 206

||||||||||||||||||||| || |||| ||||||||||||| |

Tgt 161 + CGTCTTGGACCGGCGGTAGGGCCCATGTGAAACTCCTTCGACGAGT 206

206 cols, 201 ids (97.6%), 0 gaps (0.0%)

Query >Otu30

%Id TLen Target

100% 206 Otu44

100% 206 Otu41

99% 206 Otu35

99% 206 Otu36

99% 206 Otu32

99% 206 Otu28

99% 206 Otu29

98% 206 Otu33

98% 206 Otu63

98% 206 Otu88

Query 206nt >Otu30

Target 206nt >Otu44

Qry 1 + AAACCAACAGGGATTGCCTCAGTAACGGCGAGTGAAGCGGCAACAGCTCAAATTTGAAATCTGGCCTCTTTGGGGTCCGA 80

||||||||||||||||||||||||||||||||||||||||||||||||||||||||||||||||||||||||||||||||

Tgt 1 + AAACCAACAGGGATTGCCTCAGTAACGGCGAGTGAAGCGGCAACAGCTCAAATTTGAAATCTGGCCTCTTTGGGGTCCGA 80

Qry 81 + GTTGTAATTTGTAGAGGATGTTTCGGGTACCGCCTCGGTTTAAATTTCTTGGAACAGAATGTCAGAGAGGGTGAGAATCC 160

||||||||||||||||||||||||||||||||||||||||||||||||||||||||||||||||||||||||||||||||

Tgt 81 + GTTGTAATTTGTAGAGGATGTTTCGGGTACCGCCTCGGTTTAAATTTCTTGGAACAGAATGTCAGAGAGGGTGAGAATCC 160

Qry 161 + CGTCTTGGACCGGCGGTAGGGGCCCTGTGAAACTCCTTCGACGAGT 206

||||||||||||||||||||||||||||||||||||||||||| ||

Tgt 161 + CGTCTTGGACCGGCGGTAGGGGCCCTGTGAAACTCCTTCGACGGGT 206

206 cols, 205 ids (99.5%), 0 gaps (0.0%)

Query 206nt >Otu30

Target 206nt >Otu41

Qry 1 + AAACCAACAGGGATTGCCTCAGTAACGGCGAGTGAAGCGGCAACAGCTCAAATTTGAAATCTGGCCTCTTTGGGGTCCGA 80

||||||||||||||||||||||||||||||||||||||||||||||||||||||||||||||||||||||||||||||||

Tgt 1 + AAACCAACAGGGATTGCCTCAGTAACGGCGAGTGAAGCGGCAACAGCTCAAATTTGAAATCTGGCCTCTTTGGGGTCCGA 80

Qry 81 + GTTGTAATTTGTAGAGGATGTTTCGGGTACCGCCTCGGTTTAAATTTCTTGGAACAGAATGTCAGAGAGGGTGAGAATCC 160

||||||||||||||||||||||||||||||||||||||||||||||||||||||||||||||||||||||||||||||||

Tgt 81 + GTTGTAATTTGTAGAGGATGTTTCGGGTACCGCCTCGGTTTAAATTTCTTGGAACAGAATGTCAGAGAGGGTGAGAATCC 160

Qry 161 + CGTCTTGGACCGGCGGTAGGGGCCCTGTGAAACTCCTTCGACGAGT 206

||||||||||||||||||||| ||||||||||||||||||||||||

Tgt 161 + CGTCTTGGACCGGCGGTAGGGCCCCTGTGAAACTCCTTCGACGAGT 206

206 cols, 205 ids (99.5%), 0 gaps (0.0%)

Query 206nt >Otu30

Target 206nt >Otu35

Qry 1 + AAACCAACAGGGATTGCCTCAGTAACGGCGAGTGAAGCGGCAACAGCTCAAATTTGAAATCTGGCCTCTTTGGGGTCCGA 80

||||||||||||||||||||||||||||||||||||||||||||||||||||||||||||||||||||||||||||||||

Tgt 1 + AAACCAACAGGGATTGCCTCAGTAACGGCGAGTGAAGCGGCAACAGCTCAAATTTGAAATCTGGCCTCTTTGGGGTCCGA 80

Qry 81 + GTTGTAATTTGTAGAGGATGTTTCGGGTACCGCCTCGGTTTAAATTTCTTGGAACAGAATGTCAGAGAGGGTGAGAATCC 160

||||||||||||||||||||||||||||||||||||||||||||||||||||||||||||||||||||||||||||||||

Tgt 81 + GTTGTAATTTGTAGAGGATGTTTCGGGTACCGCCTCGGTTTAAATTTCTTGGAACAGAATGTCAGAGAGGGTGAGAATCC 160

Qry 161 + CGTCTTGGACCGGCGGTAGGGGCCCTGTGAAACTCCTTCGACGAGT 206

||||||||||||||||||||| || |||||||||||||||||||||

Tgt 161 + CGTCTTGGACCGGCGGTAGGGCCCATGTGAAACTCCTTCGACGAGT 206

206 cols, 204 ids (99.0%), 0 gaps (0.0%)

Query 206nt >Otu30

Target 206nt >Otu36

Qry 1 + AAACCAACAGGGATTGCCTCAGTAACGGCGAGTGAAGCGGCAACAGCTCAAATTTGAAATCTGGCCTCTTTGGGGTCCGA 80

||||||||||||||||||||||||||||||||||||||||||||||||||||||||||||||||||||||||||||||||

Tgt 1 + AAACCAACAGGGATTGCCTCAGTAACGGCGAGTGAAGCGGCAACAGCTCAAATTTGAAATCTGGCCTCTTTGGGGTCCGA 80

Qry 81 + GTTGTAATTTGTAGAGGATGTTTCGGGTACCGCCTCGGTTTAAATTTCTTGGAACAGAATGTCAGAGAGGGTGAGAATCC 160

||||||||||||||||||||||||||||||||||||||||||||||||||||||||||||||||||||||||||||||||

Tgt 81 + GTTGTAATTTGTAGAGGATGTTTCGGGTACCGCCTCGGTTTAAATTTCTTGGAACAGAATGTCAGAGAGGGTGAGAATCC 160

Qry 161 + CGTCTTGGACCGGCGGTAGGGGCCCTGTGAAACTCCTTCGACGAGT 206

||||||||||||||||||||||||||||||||||||||||||| |

Tgt 161 + CGTCTTGGACCGGCGGTAGGGGCCCTGTGAAACTCCTTCGACGGGG 206

206 cols, 204 ids (99.0%), 0 gaps (0.0%)

Query 206nt >Otu30

Target 206nt >Otu32

Qry 1 + AAACCAACAGGGATTGCCTCAGTAACGGCGAGTGAAGCGGCAACAGCTCAAATTTGAAATCTGGCCTCTTTGGGGTCCGA 80

||||||||||||||||||||||||||||||||||||||||||||||||||||||||||||||||||||||||||||||||

Tgt 1 + AAACCAACAGGGATTGCCTCAGTAACGGCGAGTGAAGCGGCAACAGCTCAAATTTGAAATCTGGCCTCTTTGGGGTCCGA 80

Qry 81 + GTTGTAATTTGTAGAGGATGTTTCGGGTACCGCCTCGGTTTAAATTTCTTGGAACAGAATGTCAGAGAGGGTGAGAATCC 160

||||||||||||||||||||||||||||||||||||||||||||||||||||||||||||||||||||||||||||||||

Tgt 81 + GTTGTAATTTGTAGAGGATGTTTCGGGTACCGCCTCGGTTTAAATTTCTTGGAACAGAATGTCAGAGAGGGTGAGAATCC 160

Qry 161 + CGTCTTGGACCGGCGGTAGGGGCCCTGTGAAACTCCTTCGACGAGT 206

|||||||||||||||||||||||| |||| ||||||||||||||||

Tgt 161 + CGTCTTGGACCGGCGGTAGGGGCCATGTGGAACTCCTTCGACGAGT 206

206 cols, 204 ids (99.0%), 0 gaps (0.0%)

Query 206nt >Otu30

Target 206nt >Otu28

Qry 1 + AAACCAACAGGGATTGCCTCAGTAACGGCGAGTGAAGCGGCAACAGCTCAAATTTGAAATCTGGCCTCTTTGGGGTCCGA 80

||||||||||||||||||||||||||||||||||||||||||||||||||||||||||||||||||||||||||||||||

Tgt 1 + AAACCAACAGGGATTGCCTCAGTAACGGCGAGTGAAGCGGCAACAGCTCAAATTTGAAATCTGGCCTCTTTGGGGTCCGA 80

Qry 81 + GTTGTAATTTGTAGAGGATGTTTCGGGTACCGCCTCGGTTTAAATTTCTTGGAACAGAATGTCAGAGAGGGTGAGAATCC 160

||||||||||||||||||||||||||||||||||||||||||||||||||||||||||||||||||||||||||||||||

Tgt 81 + GTTGTAATTTGTAGAGGATGTTTCGGGTACCGCCTCGGTTTAAATTTCTTGGAACAGAATGTCAGAGAGGGTGAGAATCC 160

Qry 161 + CGTCTTGGACCGGCGGTAGGGGCCCTGTGAAACTCCTTCGACGAGT 206

|||||||||||||||||||||||| |||||||||||||||||| |

Tgt 161 + CGTCTTGGACCGGCGGTAGGGGCCATGTGAAACTCCTTCGACGGGG 206

206 cols, 203 ids (98.5%), 0 gaps (0.0%)

Query 206nt >Otu30

Target 206nt >Otu29

Qry 1 + AAACCAACAGGGATTGCCTCAGTAACGGCGAGTGAAGCGGCAACAGCTCAAATTTGAAATCTGGCCTCTTTGGGGTCCGA 80

||||||||||||||||||||||||||||||||||||||||||||||||||||||||||||||||||||||||||||||||

Tgt 1 + AAACCAACAGGGATTGCCTCAGTAACGGCGAGTGAAGCGGCAACAGCTCAAATTTGAAATCTGGCCTCTTTGGGGTCCGA 80

Qry 81 + GTTGTAATTTGTAGAGGATGTTTCGGGTACCGCCTCGGTTTAAATTTCTTGGAACAGAATGTCAGAGAGGGTGAGAATCC 160

||||||||||||||||||||||||||||||||||||||||||||||||||||||||||||||||||||||||||||||||

Tgt 81 + GTTGTAATTTGTAGAGGATGTTTCGGGTACCGCCTCGGTTTAAATTTCTTGGAACAGAATGTCAGAGAGGGTGAGAATCC 160

Qry 161 + CGTCTTGGACCGGCGGTAGGGGCCCTGTGAAACTCCTTCGACGAGT 206

||||||||||||||||||||| || ||||||||||||||||||||

Tgt 161 + CGTCTTGGACCGGCGGTAGGGCCCATGTGAAACTCCTTCGACGAGG 206

206 cols, 203 ids (98.5%), 0 gaps (0.0%)

Query 206nt >Otu30

Target 206nt >Otu33

Qry 1 + AAACCAACAGGGATTGCCTCAGTAACGGCGAGTGAAGCGGCAACAGCTCAAATTTGAAATCTGGCCTCTTTGGGGTCCGA 80

||||||||||||||||||||||||||||||||||||||||||||||||||||||||||||||||||||||||||||||||

Tgt 1 + AAACCAACAGGGATTGCCTCAGTAACGGCGAGTGAAGCGGCAACAGCTCAAATTTGAAATCTGGCCTCTTTGGGGTCCGA 80

Qry 81 + GTTGTAATTTGTAGAGGATGTTTCGGGTACCGCCTCGGTTTAAATTTCTTGGAACAGAATGTCAGAGAGGGTGAGAATCC 160

||||||||||||||||||||||||||||||||||||||||||||||||||||||||||||||||||||||||||||||||

Tgt 81 + GTTGTAATTTGTAGAGGATGTTTCGGGTACCGCCTCGGTTTAAATTTCTTGGAACAGAATGTCAGAGAGGGTGAGAATCC 160

Qry 161 + CGTCTTGGACCGGCGGTAGGGGCCCTGTGAAACTCCTTCGACGAGT 206

|||||||||||||||||||||||| |||| ||||||||||||| |

Tgt 161 + CGTCTTGGACCGGCGGTAGGGGCCATGTGGAACTCCTTCGACGGGG 206

206 cols, 202 ids (98.1%), 0 gaps (0.0%)

Query 206nt >Otu30

Target 206nt >Otu63

Qry 1 + AAACCAACAGGGATTGCCTCAGTAACGGCGAGTGAAGCGGCAACAGCTCAAATTTGAAATCTGGCCTCTTTGGGGTCCGA 80

||||||||||||||||||||||||||||||||||||||||||||||||||||||||||||||||||||||||||||||||

Tgt 1 + AAACCAACAGGGATTGCCTCAGTAACGGCGAGTGAAGCGGCAACAGCTCAAATTTGAAATCTGGCCTCTTTGGGGTCCGA 80

Qry 81 + GTTGTAATTTGTAGAGGATGTTTCGGGTACCGCCTCGGTTTAAATTTCTTGGAACAGAATGTCAGAGAGGGTGAGAATCC 160

||||||||||||||||||||||||||||||||||||||||||||||||||||||||||||||||||||||||||||||||

Tgt 81 + GTTGTAATTTGTAGAGGATGTTTCGGGTACCGCCTCGGTTTAAATTTCTTGGAACAGAATGTCAGAGAGGGTGAGAATCC 160

Qry 161 + CGTCTTGGACCGGCGGTAGGGGCCCTGTGAAACTCCTTCGACGAGT 206

||||||||||||||||||||||||||||| |||||||||| || |

Tgt 161 + CGTCTTGGACCGGCGGTAGGGGCCCTGTGGAACTCCTTCGGCGGGG 206

206 cols, 202 ids (98.1%), 0 gaps (0.0%)

Query 206nt >Otu30

Target 206nt >Otu88

Qry 1 + AAACCAACAGGGATTGCCTCAGTAACGGCGAGTGAAGCGGCAACAGCTCAAATTTGAAATCTGGCCTCTTTGGGGTCCGA 80

||||||||||||||||||||||||||||||||||||||||||||||||||||||||||||||||||||||||||||||||

Tgt 1 + AAACCAACAGGGATTGCCTCAGTAACGGCGAGTGAAGCGGCAACAGCTCAAATTTGAAATCTGGCCTCTTTGGGGTCCGA 80

Qry 81 + GTTGTAATTTGTAGAGGATGTTTCGGGTACCGCCTCGGTTTAAATTTCTTGGAACAGAATGTCAGAGAGGGTGAGAATCC 160

||||||||||||||||||||||||||||||||||||||||||||||||||||||||||||||||||||||||||||||||

Tgt 81 + GTTGTAATTTGTAGAGGATGTTTCGGGTACCGCCTCGGTTTAAATTTCTTGGAACAGAATGTCAGAGAGGGTGAGAATCC 160

Qry 161 + CGTCTTGGACCGGCGGTAGGGGCCCTGTGAAACTCCTTCGACGAGT 206

||||||||||||||||||||||||||||| || ||||||| || |

Tgt 161 + CGTCTTGGACCGGCGGTAGGGGCCCTGTGGAAATCCTTCGGCGGGG 206

206 cols, 201 ids (97.6%), 0 gaps (0.0%)

Query >Otu28

%Id TLen Target

100% 206 Otu36

100% 206 Otu33

99% 206 Otu29

99% 206 Otu44

99% 206 Otu35

99% 206 Otu32

99% 206 Otu63

98% 206 Otu88

98% 206 Otu41

Query 206nt >Otu28

Target 206nt >Otu36

Qry 1 + AAACCAACAGGGATTGCCTCAGTAACGGCGAGTGAAGCGGCAACAGCTCAAATTTGAAATCTGGCCTCTTTGGGGTCCGA 80

||||||||||||||||||||||||||||||||||||||||||||||||||||||||||||||||||||||||||||||||

Tgt 1 + AAACCAACAGGGATTGCCTCAGTAACGGCGAGTGAAGCGGCAACAGCTCAAATTTGAAATCTGGCCTCTTTGGGGTCCGA 80

Qry 81 + GTTGTAATTTGTAGAGGATGTTTCGGGTACCGCCTCGGTTTAAATTTCTTGGAACAGAATGTCAGAGAGGGTGAGAATCC 160

||||||||||||||||||||||||||||||||||||||||||||||||||||||||||||||||||||||||||||||||

Tgt 81 + GTTGTAATTTGTAGAGGATGTTTCGGGTACCGCCTCGGTTTAAATTTCTTGGAACAGAATGTCAGAGAGGGTGAGAATCC 160

Qry 161 + CGTCTTGGACCGGCGGTAGGGGCCATGTGAAACTCCTTCGACGGGG 206

|||||||||||||||||||||||| |||||||||||||||||||||

Tgt 161 + CGTCTTGGACCGGCGGTAGGGGCCCTGTGAAACTCCTTCGACGGGG 206

206 cols, 205 ids (99.5%), 0 gaps (0.0%)

Query 206nt >Otu28

Target 206nt >Otu33

Qry 1 + AAACCAACAGGGATTGCCTCAGTAACGGCGAGTGAAGCGGCAACAGCTCAAATTTGAAATCTGGCCTCTTTGGGGTCCGA 80

||||||||||||||||||||||||||||||||||||||||||||||||||||||||||||||||||||||||||||||||

Tgt 1 + AAACCAACAGGGATTGCCTCAGTAACGGCGAGTGAAGCGGCAACAGCTCAAATTTGAAATCTGGCCTCTTTGGGGTCCGA 80

Qry 81 + GTTGTAATTTGTAGAGGATGTTTCGGGTACCGCCTCGGTTTAAATTTCTTGGAACAGAATGTCAGAGAGGGTGAGAATCC 160

||||||||||||||||||||||||||||||||||||||||||||||||||||||||||||||||||||||||||||||||

Tgt 81 + GTTGTAATTTGTAGAGGATGTTTCGGGTACCGCCTCGGTTTAAATTTCTTGGAACAGAATGTCAGAGAGGGTGAGAATCC 160

Qry 161 + CGTCTTGGACCGGCGGTAGGGGCCATGTGAAACTCCTTCGACGGGG 206

||||||||||||||||||||||||||||| ||||||||||||||||

Tgt 161 + CGTCTTGGACCGGCGGTAGGGGCCATGTGGAACTCCTTCGACGGGG 206

206 cols, 205 ids (99.5%), 0 gaps (0.0%)

Query 206nt >Otu28

Target 206nt >Otu29

Qry 1 + AAACCAACAGGGATTGCCTCAGTAACGGCGAGTGAAGCGGCAACAGCTCAAATTTGAAATCTGGCCTCTTTGGGGTCCGA 80

||||||||||||||||||||||||||||||||||||||||||||||||||||||||||||||||||||||||||||||||

Tgt 1 + AAACCAACAGGGATTGCCTCAGTAACGGCGAGTGAAGCGGCAACAGCTCAAATTTGAAATCTGGCCTCTTTGGGGTCCGA 80

Qry 81 + GTTGTAATTTGTAGAGGATGTTTCGGGTACCGCCTCGGTTTAAATTTCTTGGAACAGAATGTCAGAGAGGGTGAGAATCC 160

||||||||||||||||||||||||||||||||||||||||||||||||||||||||||||||||||||||||||||||||

Tgt 81 + GTTGTAATTTGTAGAGGATGTTTCGGGTACCGCCTCGGTTTAAATTTCTTGGAACAGAATGTCAGAGAGGGTGAGAATCC 160

Qry 161 + CGTCTTGGACCGGCGGTAGGGGCCATGTGAAACTCCTTCGACGGGG 206

||||||||||||||||||||| ||||||||||||||||||||| ||

Tgt 161 + CGTCTTGGACCGGCGGTAGGGCCCATGTGAAACTCCTTCGACGAGG 206

206 cols, 204 ids (99.0%), 0 gaps (0.0%)

Query 206nt >Otu28

Target 206nt >Otu44

Qry 1 + AAACCAACAGGGATTGCCTCAGTAACGGCGAGTGAAGCGGCAACAGCTCAAATTTGAAATCTGGCCTCTTTGGGGTCCGA 80

||||||||||||||||||||||||||||||||||||||||||||||||||||||||||||||||||||||||||||||||

Tgt 1 + AAACCAACAGGGATTGCCTCAGTAACGGCGAGTGAAGCGGCAACAGCTCAAATTTGAAATCTGGCCTCTTTGGGGTCCGA 80

Qry 81 + GTTGTAATTTGTAGAGGATGTTTCGGGTACCGCCTCGGTTTAAATTTCTTGGAACAGAATGTCAGAGAGGGTGAGAATCC 160

||||||||||||||||||||||||||||||||||||||||||||||||||||||||||||||||||||||||||||||||

Tgt 81 + GTTGTAATTTGTAGAGGATGTTTCGGGTACCGCCTCGGTTTAAATTTCTTGGAACAGAATGTCAGAGAGGGTGAGAATCC 160

Qry 161 + CGTCTTGGACCGGCGGTAGGGGCCATGTGAAACTCCTTCGACGGGG 206

|||||||||||||||||||||||| ||||||||||||||||||||

Tgt 161 + CGTCTTGGACCGGCGGTAGGGGCCCTGTGAAACTCCTTCGACGGGT 206

206 cols, 204 ids (99.0%), 0 gaps (0.0%)

Query 206nt >Otu28

Target 206nt >Otu35

Qry 1 + AAACCAACAGGGATTGCCTCAGTAACGGCGAGTGAAGCGGCAACAGCTCAAATTTGAAATCTGGCCTCTTTGGGGTCCGA 80

||||||||||||||||||||||||||||||||||||||||||||||||||||||||||||||||||||||||||||||||

Tgt 1 + AAACCAACAGGGATTGCCTCAGTAACGGCGAGTGAAGCGGCAACAGCTCAAATTTGAAATCTGGCCTCTTTGGGGTCCGA 80

Qry 81 + GTTGTAATTTGTAGAGGATGTTTCGGGTACCGCCTCGGTTTAAATTTCTTGGAACAGAATGTCAGAGAGGGTGAGAATCC 160

||||||||||||||||||||||||||||||||||||||||||||||||||||||||||||||||||||||||||||||||

Tgt 81 + GTTGTAATTTGTAGAGGATGTTTCGGGTACCGCCTCGGTTTAAATTTCTTGGAACAGAATGTCAGAGAGGGTGAGAATCC 160

Qry 161 + CGTCTTGGACCGGCGGTAGGGGCCATGTGAAACTCCTTCGACGGGG 206

||||||||||||||||||||| ||||||||||||||||||||| |

Tgt 161 + CGTCTTGGACCGGCGGTAGGGCCCATGTGAAACTCCTTCGACGAGT 206

206 cols, 203 ids (98.5%), 0 gaps (0.0%)

Query 206nt >Otu28

Target 206nt >Otu32

Qry 1 + AAACCAACAGGGATTGCCTCAGTAACGGCGAGTGAAGCGGCAACAGCTCAAATTTGAAATCTGGCCTCTTTGGGGTCCGA 80

||||||||||||||||||||||||||||||||||||||||||||||||||||||||||||||||||||||||||||||||

Tgt 1 + AAACCAACAGGGATTGCCTCAGTAACGGCGAGTGAAGCGGCAACAGCTCAAATTTGAAATCTGGCCTCTTTGGGGTCCGA 80

Qry 81 + GTTGTAATTTGTAGAGGATGTTTCGGGTACCGCCTCGGTTTAAATTTCTTGGAACAGAATGTCAGAGAGGGTGAGAATCC 160

||||||||||||||||||||||||||||||||||||||||||||||||||||||||||||||||||||||||||||||||

Tgt 81 + GTTGTAATTTGTAGAGGATGTTTCGGGTACCGCCTCGGTTTAAATTTCTTGGAACAGAATGTCAGAGAGGGTGAGAATCC 160

Qry 161 + CGTCTTGGACCGGCGGTAGGGGCCATGTGAAACTCCTTCGACGGGG 206

||||||||||||||||||||||||||||| ||||||||||||| |

Tgt 161 + CGTCTTGGACCGGCGGTAGGGGCCATGTGGAACTCCTTCGACGAGT 206

206 cols, 203 ids (98.5%), 0 gaps (0.0%)

Query 206nt >Otu28

Target 206nt >Otu63

Qry 1 + AAACCAACAGGGATTGCCTCAGTAACGGCGAGTGAAGCGGCAACAGCTCAAATTTGAAATCTGGCCTCTTTGGGGTCCGA 80

||||||||||||||||||||||||||||||||||||||||||||||||||||||||||||||||||||||||||||||||

Tgt 1 + AAACCAACAGGGATTGCCTCAGTAACGGCGAGTGAAGCGGCAACAGCTCAAATTTGAAATCTGGCCTCTTTGGGGTCCGA 80

Qry 81 + GTTGTAATTTGTAGAGGATGTTTCGGGTACCGCCTCGGTTTAAATTTCTTGGAACAGAATGTCAGAGAGGGTGAGAATCC 160

||||||||||||||||||||||||||||||||||||||||||||||||||||||||||||||||||||||||||||||||

Tgt 81 + GTTGTAATTTGTAGAGGATGTTTCGGGTACCGCCTCGGTTTAAATTTCTTGGAACAGAATGTCAGAGAGGGTGAGAATCC 160

Qry 161 + CGTCTTGGACCGGCGGTAGGGGCCATGTGAAACTCCTTCGACGGGG 206

|||||||||||||||||||||||| |||| |||||||||| |||||

Tgt 161 + CGTCTTGGACCGGCGGTAGGGGCCCTGTGGAACTCCTTCGGCGGGG 206

206 cols, 203 ids (98.5%), 0 gaps (0.0%)

Query 206nt >Otu28

Target 206nt >Otu88

Qry 1 + AAACCAACAGGGATTGCCTCAGTAACGGCGAGTGAAGCGGCAACAGCTCAAATTTGAAATCTGGCCTCTTTGGGGTCCGA 80

||||||||||||||||||||||||||||||||||||||||||||||||||||||||||||||||||||||||||||||||

Tgt 1 + AAACCAACAGGGATTGCCTCAGTAACGGCGAGTGAAGCGGCAACAGCTCAAATTTGAAATCTGGCCTCTTTGGGGTCCGA 80

Qry 81 + GTTGTAATTTGTAGAGGATGTTTCGGGTACCGCCTCGGTTTAAATTTCTTGGAACAGAATGTCAGAGAGGGTGAGAATCC 160

||||||||||||||||||||||||||||||||||||||||||||||||||||||||||||||||||||||||||||||||

Tgt 81 + GTTGTAATTTGTAGAGGATGTTTCGGGTACCGCCTCGGTTTAAATTTCTTGGAACAGAATGTCAGAGAGGGTGAGAATCC 160

Qry 161 + CGTCTTGGACCGGCGGTAGGGGCCATGTGAAACTCCTTCGACGGGG 206

|||||||||||||||||||||||| |||| || ||||||| |||||

Tgt 161 + CGTCTTGGACCGGCGGTAGGGGCCCTGTGGAAATCCTTCGGCGGGG 206

206 cols, 202 ids (98.1%), 0 gaps (0.0%)

Query 206nt >Otu28

Target 206nt >Otu41

Qry 1 + AAACCAACAGGGATTGCCTCAGTAACGGCGAGTGAAGCGGCAACAGCTCAAATTTGAAATCTGGCCTCTTTGGGGTCCGA 80

||||||||||||||||||||||||||||||||||||||||||||||||||||||||||||||||||||||||||||||||

Tgt 1 + AAACCAACAGGGATTGCCTCAGTAACGGCGAGTGAAGCGGCAACAGCTCAAATTTGAAATCTGGCCTCTTTGGGGTCCGA 80

Qry 81 + GTTGTAATTTGTAGAGGATGTTTCGGGTACCGCCTCGGTTTAAATTTCTTGGAACAGAATGTCAGAGAGGGTGAGAATCC 160

||||||||||||||||||||||||||||||||||||||||||||||||||||||||||||||||||||||||||||||||

Tgt 81 + GTTGTAATTTGTAGAGGATGTTTCGGGTACCGCCTCGGTTTAAATTTCTTGGAACAGAATGTCAGAGAGGGTGAGAATCC 160

Qry 161 + CGTCTTGGACCGGCGGTAGGGGCCATGTGAAACTCCTTCGACGGGG 206

||||||||||||||||||||| || |||||||||||||||||| |

Tgt 161 + CGTCTTGGACCGGCGGTAGGGCCCCTGTGAAACTCCTTCGACGAGT 206

206 cols, 202 ids (98.1%), 0 gaps (0.0%)

Query >Otu33

%Id TLen Target

99% 206 Otu63

99% 206 Otu36

99% 206 Otu32

99% 206 Otu88

99% 206 Otu44

99% 206 Otu29

98% 206 Otu35

98% 206 Otu41

Query 206nt >Otu33

Target 206nt >Otu63

Qry 1 + AAACCAACAGGGATTGCCTCAGTAACGGCGAGTGAAGCGGCAACAGCTCAAATTTGAAATCTGGCCTCTTTGGGGTCCGA 80

||||||||||||||||||||||||||||||||||||||||||||||||||||||||||||||||||||||||||||||||

Tgt 1 + AAACCAACAGGGATTGCCTCAGTAACGGCGAGTGAAGCGGCAACAGCTCAAATTTGAAATCTGGCCTCTTTGGGGTCCGA 80

Qry 81 + GTTGTAATTTGTAGAGGATGTTTCGGGTACCGCCTCGGTTTAAATTTCTTGGAACAGAATGTCAGAGAGGGTGAGAATCC 160

||||||||||||||||||||||||||||||||||||||||||||||||||||||||||||||||||||||||||||||||

Tgt 81 + GTTGTAATTTGTAGAGGATGTTTCGGGTACCGCCTCGGTTTAAATTTCTTGGAACAGAATGTCAGAGAGGGTGAGAATCC 160

Qry 161 + CGTCTTGGACCGGCGGTAGGGGCCATGTGGAACTCCTTCGACGGGG 206

|||||||||||||||||||||||| ||||||||||||||| |||||

Tgt 161 + CGTCTTGGACCGGCGGTAGGGGCCCTGTGGAACTCCTTCGGCGGGG 206

206 cols, 204 ids (99.0%), 0 gaps (0.0%)

Query 206nt >Otu33

Target 206nt >Otu36

Qry 1 + AAACCAACAGGGATTGCCTCAGTAACGGCGAGTGAAGCGGCAACAGCTCAAATTTGAAATCTGGCCTCTTTGGGGTCCGA 80

||||||||||||||||||||||||||||||||||||||||||||||||||||||||||||||||||||||||||||||||

Tgt 1 + AAACCAACAGGGATTGCCTCAGTAACGGCGAGTGAAGCGGCAACAGCTCAAATTTGAAATCTGGCCTCTTTGGGGTCCGA 80

Qry 81 + GTTGTAATTTGTAGAGGATGTTTCGGGTACCGCCTCGGTTTAAATTTCTTGGAACAGAATGTCAGAGAGGGTGAGAATCC 160

||||||||||||||||||||||||||||||||||||||||||||||||||||||||||||||||||||||||||||||||

Tgt 81 + GTTGTAATTTGTAGAGGATGTTTCGGGTACCGCCTCGGTTTAAATTTCTTGGAACAGAATGTCAGAGAGGGTGAGAATCC 160

Qry 161 + CGTCTTGGACCGGCGGTAGGGGCCATGTGGAACTCCTTCGACGGGG 206

|||||||||||||||||||||||| |||| ||||||||||||||||

Tgt 161 + CGTCTTGGACCGGCGGTAGGGGCCCTGTGAAACTCCTTCGACGGGG 206

206 cols, 204 ids (99.0%), 0 gaps (0.0%)

Query 206nt >Otu33

Target 206nt >Otu32

Qry 1 + AAACCAACAGGGATTGCCTCAGTAACGGCGAGTGAAGCGGCAACAGCTCAAATTTGAAATCTGGCCTCTTTGGGGTCCGA 80

||||||||||||||||||||||||||||||||||||||||||||||||||||||||||||||||||||||||||||||||

Tgt 1 + AAACCAACAGGGATTGCCTCAGTAACGGCGAGTGAAGCGGCAACAGCTCAAATTTGAAATCTGGCCTCTTTGGGGTCCGA 80

Qry 81 + GTTGTAATTTGTAGAGGATGTTTCGGGTACCGCCTCGGTTTAAATTTCTTGGAACAGAATGTCAGAGAGGGTGAGAATCC 160

||||||||||||||||||||||||||||||||||||||||||||||||||||||||||||||||||||||||||||||||

Tgt 81 + GTTGTAATTTGTAGAGGATGTTTCGGGTACCGCCTCGGTTTAAATTTCTTGGAACAGAATGTCAGAGAGGGTGAGAATCC 160

Qry 161 + CGTCTTGGACCGGCGGTAGGGGCCATGTGGAACTCCTTCGACGGGG 206

||||||||||||||||||||||||||||||||||||||||||| |

Tgt 161 + CGTCTTGGACCGGCGGTAGGGGCCATGTGGAACTCCTTCGACGAGT 206

206 cols, 204 ids (99.0%), 0 gaps (0.0%)

Query 206nt >Otu33

Target 206nt >Otu88

Qry 1 + AAACCAACAGGGATTGCCTCAGTAACGGCGAGTGAAGCGGCAACAGCTCAAATTTGAAATCTGGCCTCTTTGGGGTCCGA 80

||||||||||||||||||||||||||||||||||||||||||||||||||||||||||||||||||||||||||||||||

Tgt 1 + AAACCAACAGGGATTGCCTCAGTAACGGCGAGTGAAGCGGCAACAGCTCAAATTTGAAATCTGGCCTCTTTGGGGTCCGA 80

Qry 81 + GTTGTAATTTGTAGAGGATGTTTCGGGTACCGCCTCGGTTTAAATTTCTTGGAACAGAATGTCAGAGAGGGTGAGAATCC 160

||||||||||||||||||||||||||||||||||||||||||||||||||||||||||||||||||||||||||||||||

Tgt 81 + GTTGTAATTTGTAGAGGATGTTTCGGGTACCGCCTCGGTTTAAATTTCTTGGAACAGAATGTCAGAGAGGGTGAGAATCC 160

Qry 161 + CGTCTTGGACCGGCGGTAGGGGCCATGTGGAACTCCTTCGACGGGG 206

|||||||||||||||||||||||| ||||||| ||||||| |||||

Tgt 161 + CGTCTTGGACCGGCGGTAGGGGCCCTGTGGAAATCCTTCGGCGGGG 206

206 cols, 203 ids (98.5%), 0 gaps (0.0%)

Query 206nt >Otu33

Target 206nt >Otu44

Qry 1 + AAACCAACAGGGATTGCCTCAGTAACGGCGAGTGAAGCGGCAACAGCTCAAATTTGAAATCTGGCCTCTTTGGGGTCCGA 80

||||||||||||||||||||||||||||||||||||||||||||||||||||||||||||||||||||||||||||||||

Tgt 1 + AAACCAACAGGGATTGCCTCAGTAACGGCGAGTGAAGCGGCAACAGCTCAAATTTGAAATCTGGCCTCTTTGGGGTCCGA 80

Qry 81 + GTTGTAATTTGTAGAGGATGTTTCGGGTACCGCCTCGGTTTAAATTTCTTGGAACAGAATGTCAGAGAGGGTGAGAATCC 160

||||||||||||||||||||||||||||||||||||||||||||||||||||||||||||||||||||||||||||||||

Tgt 81 + GTTGTAATTTGTAGAGGATGTTTCGGGTACCGCCTCGGTTTAAATTTCTTGGAACAGAATGTCAGAGAGGGTGAGAATCC 160

Qry 161 + CGTCTTGGACCGGCGGTAGGGGCCATGTGGAACTCCTTCGACGGGG 206

|||||||||||||||||||||||| |||| |||||||||||||||

Tgt 161 + CGTCTTGGACCGGCGGTAGGGGCCCTGTGAAACTCCTTCGACGGGT 206

206 cols, 203 ids (98.5%), 0 gaps (0.0%)

Query 206nt >Otu33

Target 206nt >Otu29

Qry 1 + AAACCAACAGGGATTGCCTCAGTAACGGCGAGTGAAGCGGCAACAGCTCAAATTTGAAATCTGGCCTCTTTGGGGTCCGA 80

||||||||||||||||||||||||||||||||||||||||||||||||||||||||||||||||||||||||||||||||

Tgt 1 + AAACCAACAGGGATTGCCTCAGTAACGGCGAGTGAAGCGGCAACAGCTCAAATTTGAAATCTGGCCTCTTTGGGGTCCGA 80

Qry 81 + GTTGTAATTTGTAGAGGATGTTTCGGGTACCGCCTCGGTTTAAATTTCTTGGAACAGAATGTCAGAGAGGGTGAGAATCC 160

||||||||||||||||||||||||||||||||||||||||||||||||||||||||||||||||||||||||||||||||

Tgt 81 + GTTGTAATTTGTAGAGGATGTTTCGGGTACCGCCTCGGTTTAAATTTCTTGGAACAGAATGTCAGAGAGGGTGAGAATCC 160

Qry 161 + CGTCTTGGACCGGCGGTAGGGGCCATGTGGAACTCCTTCGACGGGG 206

||||||||||||||||||||| ||||||| ||||||||||||| ||

Tgt 161 + CGTCTTGGACCGGCGGTAGGGCCCATGTGAAACTCCTTCGACGAGG 206

206 cols, 203 ids (98.5%), 0 gaps (0.0%)

Query 206nt >Otu33

Target 206nt >Otu35

Qry 1 + AAACCAACAGGGATTGCCTCAGTAACGGCGAGTGAAGCGGCAACAGCTCAAATTTGAAATCTGGCCTCTTTGGGGTCCGA 80

||||||||||||||||||||||||||||||||||||||||||||||||||||||||||||||||||||||||||||||||

Tgt 1 + AAACCAACAGGGATTGCCTCAGTAACGGCGAGTGAAGCGGCAACAGCTCAAATTTGAAATCTGGCCTCTTTGGGGTCCGA 80

Qry 81 + GTTGTAATTTGTAGAGGATGTTTCGGGTACCGCCTCGGTTTAAATTTCTTGGAACAGAATGTCAGAGAGGGTGAGAATCC 160

||||||||||||||||||||||||||||||||||||||||||||||||||||||||||||||||||||||||||||||||

Tgt 81 + GTTGTAATTTGTAGAGGATGTTTCGGGTACCGCCTCGGTTTAAATTTCTTGGAACAGAATGTCAGAGAGGGTGAGAATCC 160

Qry 161 + CGTCTTGGACCGGCGGTAGGGGCCATGTGGAACTCCTTCGACGGGG 206

||||||||||||||||||||| ||||||| ||||||||||||| |

Tgt 161 + CGTCTTGGACCGGCGGTAGGGCCCATGTGAAACTCCTTCGACGAGT 206

206 cols, 202 ids (98.1%), 0 gaps (0.0%)

Query 206nt >Otu33

Target 206nt >Otu41

Qry 1 + AAACCAACAGGGATTGCCTCAGTAACGGCGAGTGAAGCGGCAACAGCTCAAATTTGAAATCTGGCCTCTTTGGGGTCCGA 80

||||||||||||||||||||||||||||||||||||||||||||||||||||||||||||||||||||||||||||||||

Tgt 1 + AAACCAACAGGGATTGCCTCAGTAACGGCGAGTGAAGCGGCAACAGCTCAAATTTGAAATCTGGCCTCTTTGGGGTCCGA 80

Qry 81 + GTTGTAATTTGTAGAGGATGTTTCGGGTACCGCCTCGGTTTAAATTTCTTGGAACAGAATGTCAGAGAGGGTGAGAATCC 160

||||||||||||||||||||||||||||||||||||||||||||||||||||||||||||||||||||||||||||||||

Tgt 81 + GTTGTAATTTGTAGAGGATGTTTCGGGTACCGCCTCGGTTTAAATTTCTTGGAACAGAATGTCAGAGAGGGTGAGAATCC 160

Qry 161 + CGTCTTGGACCGGCGGTAGGGGCCATGTGGAACTCCTTCGACGGGG 206

||||||||||||||||||||| || |||| ||||||||||||| |

Tgt 161 + CGTCTTGGACCGGCGGTAGGGCCCCTGTGAAACTCCTTCGACGAGT 206

206 cols, 201 ids (97.6%), 0 gaps (0.0%)

Query >Otu32

%Id TLen Target

99% 206 Otu35

99% 206 Otu41

99% 206 Otu44

99% 206 Otu29

98% 206 Otu36

98% 206 Otu63

98% 206 Otu88

Query 206nt >Otu32

Target 206nt >Otu35

Qry 1 + AAACCAACAGGGATTGCCTCAGTAACGGCGAGTGAAGCGGCAACAGCTCAAATTTGAAATCTGGCCTCTTTGGGGTCCGA 80

||||||||||||||||||||||||||||||||||||||||||||||||||||||||||||||||||||||||||||||||

Tgt 1 + AAACCAACAGGGATTGCCTCAGTAACGGCGAGTGAAGCGGCAACAGCTCAAATTTGAAATCTGGCCTCTTTGGGGTCCGA 80

Qry 81 + GTTGTAATTTGTAGAGGATGTTTCGGGTACCGCCTCGGTTTAAATTTCTTGGAACAGAATGTCAGAGAGGGTGAGAATCC 160

||||||||||||||||||||||||||||||||||||||||||||||||||||||||||||||||||||||||||||||||

Tgt 81 + GTTGTAATTTGTAGAGGATGTTTCGGGTACCGCCTCGGTTTAAATTTCTTGGAACAGAATGTCAGAGAGGGTGAGAATCC 160

Qry 161 + CGTCTTGGACCGGCGGTAGGGGCCATGTGGAACTCCTTCGACGAGT 206

||||||||||||||||||||| ||||||| ||||||||||||||||

Tgt 161 + CGTCTTGGACCGGCGGTAGGGCCCATGTGAAACTCCTTCGACGAGT 206

206 cols, 204 ids (99.0%), 0 gaps (0.0%)

Query 206nt >Otu32

Target 206nt >Otu41

Qry 1 + AAACCAACAGGGATTGCCTCAGTAACGGCGAGTGAAGCGGCAACAGCTCAAATTTGAAATCTGGCCTCTTTGGGGTCCGA 80

||||||||||||||||||||||||||||||||||||||||||||||||||||||||||||||||||||||||||||||||

Tgt 1 + AAACCAACAGGGATTGCCTCAGTAACGGCGAGTGAAGCGGCAACAGCTCAAATTTGAAATCTGGCCTCTTTGGGGTCCGA 80

Qry 81 + GTTGTAATTTGTAGAGGATGTTTCGGGTACCGCCTCGGTTTAAATTTCTTGGAACAGAATGTCAGAGAGGGTGAGAATCC 160

||||||||||||||||||||||||||||||||||||||||||||||||||||||||||||||||||||||||||||||||

Tgt 81 + GTTGTAATTTGTAGAGGATGTTTCGGGTACCGCCTCGGTTTAAATTTCTTGGAACAGAATGTCAGAGAGGGTGAGAATCC 160

Qry 161 + CGTCTTGGACCGGCGGTAGGGGCCATGTGGAACTCCTTCGACGAGT 206

||||||||||||||||||||| || |||| ||||||||||||||||

Tgt 161 + CGTCTTGGACCGGCGGTAGGGCCCCTGTGAAACTCCTTCGACGAGT 206

206 cols, 203 ids (98.5%), 0 gaps (0.0%)

Query 206nt >Otu32

Target 206nt >Otu44

Qry 1 + AAACCAACAGGGATTGCCTCAGTAACGGCGAGTGAAGCGGCAACAGCTCAAATTTGAAATCTGGCCTCTTTGGGGTCCGA 80

||||||||||||||||||||||||||||||||||||||||||||||||||||||||||||||||||||||||||||||||

Tgt 1 + AAACCAACAGGGATTGCCTCAGTAACGGCGAGTGAAGCGGCAACAGCTCAAATTTGAAATCTGGCCTCTTTGGGGTCCGA 80

Qry 81 + GTTGTAATTTGTAGAGGATGTTTCGGGTACCGCCTCGGTTTAAATTTCTTGGAACAGAATGTCAGAGAGGGTGAGAATCC 160

||||||||||||||||||||||||||||||||||||||||||||||||||||||||||||||||||||||||||||||||

Tgt 81 + GTTGTAATTTGTAGAGGATGTTTCGGGTACCGCCTCGGTTTAAATTTCTTGGAACAGAATGTCAGAGAGGGTGAGAATCC 160

Qry 161 + CGTCTTGGACCGGCGGTAGGGGCCATGTGGAACTCCTTCGACGAGT 206

|||||||||||||||||||||||| |||| ||||||||||||| ||

Tgt 161 + CGTCTTGGACCGGCGGTAGGGGCCCTGTGAAACTCCTTCGACGGGT 206

206 cols, 203 ids (98.5%), 0 gaps (0.0%)

Query 206nt >Otu32

Target 206nt >Otu29

Qry 1 + AAACCAACAGGGATTGCCTCAGTAACGGCGAGTGAAGCGGCAACAGCTCAAATTTGAAATCTGGCCTCTTTGGGGTCCGA 80

||||||||||||||||||||||||||||||||||||||||||||||||||||||||||||||||||||||||||||||||

Tgt 1 + AAACCAACAGGGATTGCCTCAGTAACGGCGAGTGAAGCGGCAACAGCTCAAATTTGAAATCTGGCCTCTTTGGGGTCCGA 80

Qry 81 + GTTGTAATTTGTAGAGGATGTTTCGGGTACCGCCTCGGTTTAAATTTCTTGGAACAGAATGTCAGAGAGGGTGAGAATCC 160

||||||||||||||||||||||||||||||||||||||||||||||||||||||||||||||||||||||||||||||||

Tgt 81 + GTTGTAATTTGTAGAGGATGTTTCGGGTACCGCCTCGGTTTAAATTTCTTGGAACAGAATGTCAGAGAGGGTGAGAATCC 160

Qry 161 + CGTCTTGGACCGGCGGTAGGGGCCATGTGGAACTCCTTCGACGAGT 206

||||||||||||||||||||| ||||||| |||||||||||||||

Tgt 161 + CGTCTTGGACCGGCGGTAGGGCCCATGTGAAACTCCTTCGACGAGG 206

206 cols, 203 ids (98.5%), 0 gaps (0.0%)

Query 206nt >Otu32

Target 206nt >Otu36

Qry 1 + AAACCAACAGGGATTGCCTCAGTAACGGCGAGTGAAGCGGCAACAGCTCAAATTTGAAATCTGGCCTCTTTGGGGTCCGA 80

||||||||||||||||||||||||||||||||||||||||||||||||||||||||||||||||||||||||||||||||

Tgt 1 + AAACCAACAGGGATTGCCTCAGTAACGGCGAGTGAAGCGGCAACAGCTCAAATTTGAAATCTGGCCTCTTTGGGGTCCGA 80

Qry 81 + GTTGTAATTTGTAGAGGATGTTTCGGGTACCGCCTCGGTTTAAATTTCTTGGAACAGAATGTCAGAGAGGGTGAGAATCC 160

||||||||||||||||||||||||||||||||||||||||||||||||||||||||||||||||||||||||||||||||

Tgt 81 + GTTGTAATTTGTAGAGGATGTTTCGGGTACCGCCTCGGTTTAAATTTCTTGGAACAGAATGTCAGAGAGGGTGAGAATCC 160

Qry 161 + CGTCTTGGACCGGCGGTAGGGGCCATGTGGAACTCCTTCGACGAGT 206

|||||||||||||||||||||||| |||| ||||||||||||| |

Tgt 161 + CGTCTTGGACCGGCGGTAGGGGCCCTGTGAAACTCCTTCGACGGGG 206

206 cols, 202 ids (98.1%), 0 gaps (0.0%)

Query 206nt >Otu32

Target 206nt >Otu63

Qry 1 + AAACCAACAGGGATTGCCTCAGTAACGGCGAGTGAAGCGGCAACAGCTCAAATTTGAAATCTGGCCTCTTTGGGGTCCGA 80

||||||||||||||||||||||||||||||||||||||||||||||||||||||||||||||||||||||||||||||||

Tgt 1 + AAACCAACAGGGATTGCCTCAGTAACGGCGAGTGAAGCGGCAACAGCTCAAATTTGAAATCTGGCCTCTTTGGGGTCCGA 80

Qry 81 + GTTGTAATTTGTAGAGGATGTTTCGGGTACCGCCTCGGTTTAAATTTCTTGGAACAGAATGTCAGAGAGGGTGAGAATCC 160

||||||||||||||||||||||||||||||||||||||||||||||||||||||||||||||||||||||||||||||||

Tgt 81 + GTTGTAATTTGTAGAGGATGTTTCGGGTACCGCCTCGGTTTAAATTTCTTGGAACAGAATGTCAGAGAGGGTGAGAATCC 160

Qry 161 + CGTCTTGGACCGGCGGTAGGGGCCATGTGGAACTCCTTCGACGAGT 206

|||||||||||||||||||||||| ||||||||||||||| || |

Tgt 161 + CGTCTTGGACCGGCGGTAGGGGCCCTGTGGAACTCCTTCGGCGGGG 206

206 cols, 202 ids (98.1%), 0 gaps (0.0%)

Query 206nt >Otu32

Target 206nt >Otu88

Qry 1 + AAACCAACAGGGATTGCCTCAGTAACGGCGAGTGAAGCGGCAACAGCTCAAATTTGAAATCTGGCCTCTTTGGGGTCCGA 80

||||||||||||||||||||||||||||||||||||||||||||||||||||||||||||||||||||||||||||||||

Tgt 1 + AAACCAACAGGGATTGCCTCAGTAACGGCGAGTGAAGCGGCAACAGCTCAAATTTGAAATCTGGCCTCTTTGGGGTCCGA 80

Qry 81 + GTTGTAATTTGTAGAGGATGTTTCGGGTACCGCCTCGGTTTAAATTTCTTGGAACAGAATGTCAGAGAGGGTGAGAATCC 160

||||||||||||||||||||||||||||||||||||||||||||||||||||||||||||||||||||||||||||||||

Tgt 81 + GTTGTAATTTGTAGAGGATGTTTCGGGTACCGCCTCGGTTTAAATTTCTTGGAACAGAATGTCAGAGAGGGTGAGAATCC 160

Qry 161 + CGTCTTGGACCGGCGGTAGGGGCCATGTGGAACTCCTTCGACGAGT 206

|||||||||||||||||||||||| ||||||| ||||||| || |

Tgt 161 + CGTCTTGGACCGGCGGTAGGGGCCCTGTGGAAATCCTTCGGCGGGG 206

206 cols, 201 ids (97.6%), 0 gaps (0.0%)

Query >Otu36

%Id TLen Target

100% 206 Otu44

99% 206 Otu63

99% 206 Otu88

99% 206 Otu29

99% 206 Otu41

98% 206 Otu35

Query 206nt >Otu36

Target 206nt >Otu44

Qry 1 + AAACCAACAGGGATTGCCTCAGTAACGGCGAGTGAAGCGGCAACAGCTCAAATTTGAAATCTGGCCTCTTTGGGGTCCGA 80

||||||||||||||||||||||||||||||||||||||||||||||||||||||||||||||||||||||||||||||||

Tgt 1 + AAACCAACAGGGATTGCCTCAGTAACGGCGAGTGAAGCGGCAACAGCTCAAATTTGAAATCTGGCCTCTTTGGGGTCCGA 80

Qry 81 + GTTGTAATTTGTAGAGGATGTTTCGGGTACCGCCTCGGTTTAAATTTCTTGGAACAGAATGTCAGAGAGGGTGAGAATCC 160

||||||||||||||||||||||||||||||||||||||||||||||||||||||||||||||||||||||||||||||||

Tgt 81 + GTTGTAATTTGTAGAGGATGTTTCGGGTACCGCCTCGGTTTAAATTTCTTGGAACAGAATGTCAGAGAGGGTGAGAATCC 160

Qry 161 + CGTCTTGGACCGGCGGTAGGGGCCCTGTGAAACTCCTTCGACGGGG 206

|||||||||||||||||||||||||||||||||||||||||||||

Tgt 161 + CGTCTTGGACCGGCGGTAGGGGCCCTGTGAAACTCCTTCGACGGGT 206

206 cols, 205 ids (99.5%), 0 gaps (0.0%)

Query 206nt >Otu36

Target 206nt >Otu63

Qry 1 + AAACCAACAGGGATTGCCTCAGTAACGGCGAGTGAAGCGGCAACAGCTCAAATTTGAAATCTGGCCTCTTTGGGGTCCGA 80

||||||||||||||||||||||||||||||||||||||||||||||||||||||||||||||||||||||||||||||||

Tgt 1 + AAACCAACAGGGATTGCCTCAGTAACGGCGAGTGAAGCGGCAACAGCTCAAATTTGAAATCTGGCCTCTTTGGGGTCCGA 80

Qry 81 + GTTGTAATTTGTAGAGGATGTTTCGGGTACCGCCTCGGTTTAAATTTCTTGGAACAGAATGTCAGAGAGGGTGAGAATCC 160

||||||||||||||||||||||||||||||||||||||||||||||||||||||||||||||||||||||||||||||||

Tgt 81 + GTTGTAATTTGTAGAGGATGTTTCGGGTACCGCCTCGGTTTAAATTTCTTGGAACAGAATGTCAGAGAGGGTGAGAATCC 160

Qry 161 + CGTCTTGGACCGGCGGTAGGGGCCCTGTGAAACTCCTTCGACGGGG 206

||||||||||||||||||||||||||||| |||||||||| |||||

Tgt 161 + CGTCTTGGACCGGCGGTAGGGGCCCTGTGGAACTCCTTCGGCGGGG 206

206 cols, 204 ids (99.0%), 0 gaps (0.0%)

Query 206nt >Otu36

Target 206nt >Otu88

Qry 1 + AAACCAACAGGGATTGCCTCAGTAACGGCGAGTGAAGCGGCAACAGCTCAAATTTGAAATCTGGCCTCTTTGGGGTCCGA 80

||||||||||||||||||||||||||||||||||||||||||||||||||||||||||||||||||||||||||||||||

Tgt 1 + AAACCAACAGGGATTGCCTCAGTAACGGCGAGTGAAGCGGCAACAGCTCAAATTTGAAATCTGGCCTCTTTGGGGTCCGA 80

Qry 81 + GTTGTAATTTGTAGAGGATGTTTCGGGTACCGCCTCGGTTTAAATTTCTTGGAACAGAATGTCAGAGAGGGTGAGAATCC 160

||||||||||||||||||||||||||||||||||||||||||||||||||||||||||||||||||||||||||||||||

Tgt 81 + GTTGTAATTTGTAGAGGATGTTTCGGGTACCGCCTCGGTTTAAATTTCTTGGAACAGAATGTCAGAGAGGGTGAGAATCC 160

Qry 161 + CGTCTTGGACCGGCGGTAGGGGCCCTGTGAAACTCCTTCGACGGGG 206

||||||||||||||||||||||||||||| || ||||||| |||||

Tgt 161 + CGTCTTGGACCGGCGGTAGGGGCCCTGTGGAAATCCTTCGGCGGGG 206

206 cols, 203 ids (98.5%), 0 gaps (0.0%)

Query 206nt >Otu36

Target 206nt >Otu29

Qry 1 + AAACCAACAGGGATTGCCTCAGTAACGGCGAGTGAAGCGGCAACAGCTCAAATTTGAAATCTGGCCTCTTTGGGGTCCGA 80

||||||||||||||||||||||||||||||||||||||||||||||||||||||||||||||||||||||||||||||||

Tgt 1 + AAACCAACAGGGATTGCCTCAGTAACGGCGAGTGAAGCGGCAACAGCTCAAATTTGAAATCTGGCCTCTTTGGGGTCCGA 80

Qry 81 + GTTGTAATTTGTAGAGGATGTTTCGGGTACCGCCTCGGTTTAAATTTCTTGGAACAGAATGTCAGAGAGGGTGAGAATCC 160

||||||||||||||||||||||||||||||||||||||||||||||||||||||||||||||||||||||||||||||||

Tgt 81 + GTTGTAATTTGTAGAGGATGTTTCGGGTACCGCCTCGGTTTAAATTTCTTGGAACAGAATGTCAGAGAGGGTGAGAATCC 160

Qry 161 + CGTCTTGGACCGGCGGTAGGGGCCCTGTGAAACTCCTTCGACGGGG 206

||||||||||||||||||||| || |||||||||||||||||| ||

Tgt 161 + CGTCTTGGACCGGCGGTAGGGCCCATGTGAAACTCCTTCGACGAGG 206

206 cols, 203 ids (98.5%), 0 gaps (0.0%)

Query 206nt >Otu36

Target 206nt >Otu41

Qry 1 + AAACCAACAGGGATTGCCTCAGTAACGGCGAGTGAAGCGGCAACAGCTCAAATTTGAAATCTGGCCTCTTTGGGGTCCGA 80

||||||||||||||||||||||||||||||||||||||||||||||||||||||||||||||||||||||||||||||||

Tgt 1 + AAACCAACAGGGATTGCCTCAGTAACGGCGAGTGAAGCGGCAACAGCTCAAATTTGAAATCTGGCCTCTTTGGGGTCCGA 80

Qry 81 + GTTGTAATTTGTAGAGGATGTTTCGGGTACCGCCTCGGTTTAAATTTCTTGGAACAGAATGTCAGAGAGGGTGAGAATCC 160

||||||||||||||||||||||||||||||||||||||||||||||||||||||||||||||||||||||||||||||||

Tgt 81 + GTTGTAATTTGTAGAGGATGTTTCGGGTACCGCCTCGGTTTAAATTTCTTGGAACAGAATGTCAGAGAGGGTGAGAATCC 160

Qry 161 + CGTCTTGGACCGGCGGTAGGGGCCCTGTGAAACTCCTTCGACGGGG 206

||||||||||||||||||||| ||||||||||||||||||||| |

Tgt 161 + CGTCTTGGACCGGCGGTAGGGCCCCTGTGAAACTCCTTCGACGAGT 206

206 cols, 203 ids (98.5%), 0 gaps (0.0%)

Query 206nt >Otu36

Target 206nt >Otu35

Qry 1 + AAACCAACAGGGATTGCCTCAGTAACGGCGAGTGAAGCGGCAACAGCTCAAATTTGAAATCTGGCCTCTTTGGGGTCCGA 80

||||||||||||||||||||||||||||||||||||||||||||||||||||||||||||||||||||||||||||||||

Tgt 1 + AAACCAACAGGGATTGCCTCAGTAACGGCGAGTGAAGCGGCAACAGCTCAAATTTGAAATCTGGCCTCTTTGGGGTCCGA 80

Qry 81 + GTTGTAATTTGTAGAGGATGTTTCGGGTACCGCCTCGGTTTAAATTTCTTGGAACAGAATGTCAGAGAGGGTGAGAATCC 160

||||||||||||||||||||||||||||||||||||||||||||||||||||||||||||||||||||||||||||||||

Tgt 81 + GTTGTAATTTGTAGAGGATGTTTCGGGTACCGCCTCGGTTTAAATTTCTTGGAACAGAATGTCAGAGAGGGTGAGAATCC 160

Qry 161 + CGTCTTGGACCGGCGGTAGGGGCCCTGTGAAACTCCTTCGACGGGG 206

||||||||||||||||||||| || |||||||||||||||||| |

Tgt 161 + CGTCTTGGACCGGCGGTAGGGCCCATGTGAAACTCCTTCGACGAGT 206

206 cols, 202 ids (98.1%), 0 gaps (0.0%)

Query >Otu63

%Id TLen Target

100% 206 Otu88

99% 206 Otu44

98% 206 Otu29

98% 206 Otu41

97% 206 Otu35

Query 206nt >Otu63

Target 206nt >Otu88

Qry 1 + AAACCAACAGGGATTGCCTCAGTAACGGCGAGTGAAGCGGCAACAGCTCAAATTTGAAATCTGGCCTCTTTGGGGTCCGA 80

||||||||||||||||||||||||||||||||||||||||||||||||||||||||||||||||||||||||||||||||

Tgt 1 + AAACCAACAGGGATTGCCTCAGTAACGGCGAGTGAAGCGGCAACAGCTCAAATTTGAAATCTGGCCTCTTTGGGGTCCGA 80

Qry 81 + GTTGTAATTTGTAGAGGATGTTTCGGGTACCGCCTCGGTTTAAATTTCTTGGAACAGAATGTCAGAGAGGGTGAGAATCC 160

||||||||||||||||||||||||||||||||||||||||||||||||||||||||||||||||||||||||||||||||

Tgt 81 + GTTGTAATTTGTAGAGGATGTTTCGGGTACCGCCTCGGTTTAAATTTCTTGGAACAGAATGTCAGAGAGGGTGAGAATCC 160

Qry 161 + CGTCTTGGACCGGCGGTAGGGGCCCTGTGGAACTCCTTCGGCGGGG 206

|||||||||||||||||||||||||||||||| |||||||||||||

Tgt 161 + CGTCTTGGACCGGCGGTAGGGGCCCTGTGGAAATCCTTCGGCGGGG 206

206 cols, 205 ids (99.5%), 0 gaps (0.0%)

Query 206nt >Otu63

Target 206nt >Otu44

Qry 1 + AAACCAACAGGGATTGCCTCAGTAACGGCGAGTGAAGCGGCAACAGCTCAAATTTGAAATCTGGCCTCTTTGGGGTCCGA 80

||||||||||||||||||||||||||||||||||||||||||||||||||||||||||||||||||||||||||||||||

Tgt 1 + AAACCAACAGGGATTGCCTCAGTAACGGCGAGTGAAGCGGCAACAGCTCAAATTTGAAATCTGGCCTCTTTGGGGTCCGA 80

Qry 81 + GTTGTAATTTGTAGAGGATGTTTCGGGTACCGCCTCGGTTTAAATTTCTTGGAACAGAATGTCAGAGAGGGTGAGAATCC 160

||||||||||||||||||||||||||||||||||||||||||||||||||||||||||||||||||||||||||||||||

Tgt 81 + GTTGTAATTTGTAGAGGATGTTTCGGGTACCGCCTCGGTTTAAATTTCTTGGAACAGAATGTCAGAGAGGGTGAGAATCC 160

Qry 161 + CGTCTTGGACCGGCGGTAGGGGCCCTGTGGAACTCCTTCGGCGGGG 206

||||||||||||||||||||||||||||| |||||||||| ||||

Tgt 161 + CGTCTTGGACCGGCGGTAGGGGCCCTGTGAAACTCCTTCGACGGGT 206

206 cols, 203 ids (98.5%), 0 gaps (0.0%)

Query 206nt >Otu63

Target 206nt >Otu29

Qry 1 + AAACCAACAGGGATTGCCTCAGTAACGGCGAGTGAAGCGGCAACAGCTCAAATTTGAAATCTGGCCTCTTTGGGGTCCGA 80

||||||||||||||||||||||||||||||||||||||||||||||||||||||||||||||||||||||||||||||||

Tgt 1 + AAACCAACAGGGATTGCCTCAGTAACGGCGAGTGAAGCGGCAACAGCTCAAATTTGAAATCTGGCCTCTTTGGGGTCCGA 80

Qry 81 + GTTGTAATTTGTAGAGGATGTTTCGGGTACCGCCTCGGTTTAAATTTCTTGGAACAGAATGTCAGAGAGGGTGAGAATCC 160

||||||||||||||||||||||||||||||||||||||||||||||||||||||||||||||||||||||||||||||||

Tgt 81 + GTTGTAATTTGTAGAGGATGTTTCGGGTACCGCCTCGGTTTAAATTTCTTGGAACAGAATGTCAGAGAGGGTGAGAATCC 160

Qry 161 + CGTCTTGGACCGGCGGTAGGGGCCCTGTGGAACTCCTTCGGCGGGG 206

||||||||||||||||||||| || |||| |||||||||| || ||

Tgt 161 + CGTCTTGGACCGGCGGTAGGGCCCATGTGAAACTCCTTCGACGAGG 206

206 cols, 201 ids (97.6%), 0 gaps (0.0%)

Query 206nt >Otu63

Target 206nt >Otu41

Qry 1 + AAACCAACAGGGATTGCCTCAGTAACGGCGAGTGAAGCGGCAACAGCTCAAATTTGAAATCTGGCCTCTTTGGGGTCCGA 80

||||||||||||||||||||||||||||||||||||||||||||||||||||||||||||||||||||||||||||||||

Tgt 1 + AAACCAACAGGGATTGCCTCAGTAACGGCGAGTGAAGCGGCAACAGCTCAAATTTGAAATCTGGCCTCTTTGGGGTCCGA 80

Qry 81 + GTTGTAATTTGTAGAGGATGTTTCGGGTACCGCCTCGGTTTAAATTTCTTGGAACAGAATGTCAGAGAGGGTGAGAATCC 160

||||||||||||||||||||||||||||||||||||||||||||||||||||||||||||||||||||||||||||||||

Tgt 81 + GTTGTAATTTGTAGAGGATGTTTCGGGTACCGCCTCGGTTTAAATTTCTTGGAACAGAATGTCAGAGAGGGTGAGAATCC 160

Qry 161 + CGTCTTGGACCGGCGGTAGGGGCCCTGTGGAACTCCTTCGGCGGGG 206

||||||||||||||||||||| ||||||| |||||||||| || |

Tgt 161 + CGTCTTGGACCGGCGGTAGGGCCCCTGTGAAACTCCTTCGACGAGT 206

206 cols, 201 ids (97.6%), 0 gaps (0.0%)

Query 206nt >Otu63

Target 206nt >Otu35

Qry 1 + AAACCAACAGGGATTGCCTCAGTAACGGCGAGTGAAGCGGCAACAGCTCAAATTTGAAATCTGGCCTCTTTGGGGTCCGA 80

||||||||||||||||||||||||||||||||||||||||||||||||||||||||||||||||||||||||||||||||

Tgt 1 + AAACCAACAGGGATTGCCTCAGTAACGGCGAGTGAAGCGGCAACAGCTCAAATTTGAAATCTGGCCTCTTTGGGGTCCGA 80

Qry 81 + GTTGTAATTTGTAGAGGATGTTTCGGGTACCGCCTCGGTTTAAATTTCTTGGAACAGAATGTCAGAGAGGGTGAGAATCC 160

||||||||||||||||||||||||||||||||||||||||||||||||||||||||||||||||||||||||||||||||

Tgt 81 + GTTGTAATTTGTAGAGGATGTTTCGGGTACCGCCTCGGTTTAAATTTCTTGGAACAGAATGTCAGAGAGGGTGAGAATCC 160

Qry 161 + CGTCTTGGACCGGCGGTAGGGGCCCTGTGGAACTCCTTCGGCGGGG 206

||||||||||||||||||||| || |||| |||||||||| || |

Tgt 161 + CGTCTTGGACCGGCGGTAGGGCCCATGTGAAACTCCTTCGACGAGT 206

206 cols, 200 ids (97.1%), 0 gaps (0.0%)

Query >Otu29

%Id TLen Target

100% 206 Otu35

99% 206 Otu41

98% 206 Otu44

97% 206 Otu88

Query 206nt >Otu29

Target 206nt >Otu35

Qry 1 + AAACCAACAGGGATTGCCTCAGTAACGGCGAGTGAAGCGGCAACAGCTCAAATTTGAAATCTGGCCTCTTTGGGGTCCGA 80

||||||||||||||||||||||||||||||||||||||||||||||||||||||||||||||||||||||||||||||||

Tgt 1 + AAACCAACAGGGATTGCCTCAGTAACGGCGAGTGAAGCGGCAACAGCTCAAATTTGAAATCTGGCCTCTTTGGGGTCCGA 80

Qry 81 + GTTGTAATTTGTAGAGGATGTTTCGGGTACCGCCTCGGTTTAAATTTCTTGGAACAGAATGTCAGAGAGGGTGAGAATCC 160

||||||||||||||||||||||||||||||||||||||||||||||||||||||||||||||||||||||||||||||||

Tgt 81 + GTTGTAATTTGTAGAGGATGTTTCGGGTACCGCCTCGGTTTAAATTTCTTGGAACAGAATGTCAGAGAGGGTGAGAATCC 160

Qry 161 + CGTCTTGGACCGGCGGTAGGGCCCATGTGAAACTCCTTCGACGAGG 206

|||||||||||||||||||||||||||||||||||||||||||||

Tgt 161 + CGTCTTGGACCGGCGGTAGGGCCCATGTGAAACTCCTTCGACGAGT 206

206 cols, 205 ids (99.5%), 0 gaps (0.0%)

Query 206nt >Otu29

Target 206nt >Otu41

Qry 1 + AAACCAACAGGGATTGCCTCAGTAACGGCGAGTGAAGCGGCAACAGCTCAAATTTGAAATCTGGCCTCTTTGGGGTCCGA 80

||||||||||||||||||||||||||||||||||||||||||||||||||||||||||||||||||||||||||||||||

Tgt 1 + AAACCAACAGGGATTGCCTCAGTAACGGCGAGTGAAGCGGCAACAGCTCAAATTTGAAATCTGGCCTCTTTGGGGTCCGA 80

Qry 81 + GTTGTAATTTGTAGAGGATGTTTCGGGTACCGCCTCGGTTTAAATTTCTTGGAACAGAATGTCAGAGAGGGTGAGAATCC 160

||||||||||||||||||||||||||||||||||||||||||||||||||||||||||||||||||||||||||||||||

Tgt 81 + GTTGTAATTTGTAGAGGATGTTTCGGGTACCGCCTCGGTTTAAATTTCTTGGAACAGAATGTCAGAGAGGGTGAGAATCC 160

Qry 161 + CGTCTTGGACCGGCGGTAGGGCCCATGTGAAACTCCTTCGACGAGG 206

|||||||||||||||||||||||| ||||||||||||||||||||

Tgt 161 + CGTCTTGGACCGGCGGTAGGGCCCCTGTGAAACTCCTTCGACGAGT 206

206 cols, 204 ids (99.0%), 0 gaps (0.0%)

Query 206nt >Otu29

Target 206nt >Otu44

Qry 1 + AAACCAACAGGGATTGCCTCAGTAACGGCGAGTGAAGCGGCAACAGCTCAAATTTGAAATCTGGCCTCTTTGGGGTCCGA 80

||||||||||||||||||||||||||||||||||||||||||||||||||||||||||||||||||||||||||||||||

Tgt 1 + AAACCAACAGGGATTGCCTCAGTAACGGCGAGTGAAGCGGCAACAGCTCAAATTTGAAATCTGGCCTCTTTGGGGTCCGA 80

Qry 81 + GTTGTAATTTGTAGAGGATGTTTCGGGTACCGCCTCGGTTTAAATTTCTTGGAACAGAATGTCAGAGAGGGTGAGAATCC 160

||||||||||||||||||||||||||||||||||||||||||||||||||||||||||||||||||||||||||||||||

Tgt 81 + GTTGTAATTTGTAGAGGATGTTTCGGGTACCGCCTCGGTTTAAATTTCTTGGAACAGAATGTCAGAGAGGGTGAGAATCC 160

Qry 161 + CGTCTTGGACCGGCGGTAGGGCCCATGTGAAACTCCTTCGACGAGG 206

||||||||||||||||||||| || |||||||||||||||||| |

Tgt 161 + CGTCTTGGACCGGCGGTAGGGGCCCTGTGAAACTCCTTCGACGGGT 206

206 cols, 202 ids (98.1%), 0 gaps (0.0%)

Query 206nt >Otu29

Target 206nt >Otu88

Qry 1 + AAACCAACAGGGATTGCCTCAGTAACGGCGAGTGAAGCGGCAACAGCTCAAATTTGAAATCTGGCCTCTTTGGGGTCCGA 80

||||||||||||||||||||||||||||||||||||||||||||||||||||||||||||||||||||||||||||||||

Tgt 1 + AAACCAACAGGGATTGCCTCAGTAACGGCGAGTGAAGCGGCAACAGCTCAAATTTGAAATCTGGCCTCTTTGGGGTCCGA 80

Qry 81 + GTTGTAATTTGTAGAGGATGTTTCGGGTACCGCCTCGGTTTAAATTTCTTGGAACAGAATGTCAGAGAGGGTGAGAATCC 160

||||||||||||||||||||||||||||||||||||||||||||||||||||||||||||||||||||||||||||||||

Tgt 81 + GTTGTAATTTGTAGAGGATGTTTCGGGTACCGCCTCGGTTTAAATTTCTTGGAACAGAATGTCAGAGAGGGTGAGAATCC 160

Qry 161 + CGTCTTGGACCGGCGGTAGGGCCCATGTGAAACTCCTTCGACGAGG 206

||||||||||||||||||||| || |||| || ||||||| || ||

Tgt 161 + CGTCTTGGACCGGCGGTAGGGGCCCTGTGGAAATCCTTCGGCGGGG 206

206 cols, 200 ids (97.1%), 0 gaps (0.0%)

Query >Otu41

%Id TLen Target

100% 206 Otu35

99% 206 Otu44

97% 206 Otu88

Query 206nt >Otu41

Target 206nt >Otu35

Qry 1 + AAACCAACAGGGATTGCCTCAGTAACGGCGAGTGAAGCGGCAACAGCTCAAATTTGAAATCTGGCCTCTTTGGGGTCCGA 80

||||||||||||||||||||||||||||||||||||||||||||||||||||||||||||||||||||||||||||||||

Tgt 1 + AAACCAACAGGGATTGCCTCAGTAACGGCGAGTGAAGCGGCAACAGCTCAAATTTGAAATCTGGCCTCTTTGGGGTCCGA 80

Qry 81 + GTTGTAATTTGTAGAGGATGTTTCGGGTACCGCCTCGGTTTAAATTTCTTGGAACAGAATGTCAGAGAGGGTGAGAATCC 160

||||||||||||||||||||||||||||||||||||||||||||||||||||||||||||||||||||||||||||||||

Tgt 81 + GTTGTAATTTGTAGAGGATGTTTCGGGTACCGCCTCGGTTTAAATTTCTTGGAACAGAATGTCAGAGAGGGTGAGAATCC 160

Qry 161 + CGTCTTGGACCGGCGGTAGGGCCCCTGTGAAACTCCTTCGACGAGT 206

|||||||||||||||||||||||| |||||||||||||||||||||

Tgt 161 + CGTCTTGGACCGGCGGTAGGGCCCATGTGAAACTCCTTCGACGAGT 206

206 cols, 205 ids (99.5%), 0 gaps (0.0%)

Query 206nt >Otu41

Target 206nt >Otu44

Qry 1 + AAACCAACAGGGATTGCCTCAGTAACGGCGAGTGAAGCGGCAACAGCTCAAATTTGAAATCTGGCCTCTTTGGGGTCCGA 80

||||||||||||||||||||||||||||||||||||||||||||||||||||||||||||||||||||||||||||||||

Tgt 1 + AAACCAACAGGGATTGCCTCAGTAACGGCGAGTGAAGCGGCAACAGCTCAAATTTGAAATCTGGCCTCTTTGGGGTCCGA 80

Qry 81 + GTTGTAATTTGTAGAGGATGTTTCGGGTACCGCCTCGGTTTAAATTTCTTGGAACAGAATGTCAGAGAGGGTGAGAATCC 160

||||||||||||||||||||||||||||||||||||||||||||||||||||||||||||||||||||||||||||||||

Tgt 81 + GTTGTAATTTGTAGAGGATGTTTCGGGTACCGCCTCGGTTTAAATTTCTTGGAACAGAATGTCAGAGAGGGTGAGAATCC 160

Qry 161 + CGTCTTGGACCGGCGGTAGGGCCCCTGTGAAACTCCTTCGACGAGT 206

||||||||||||||||||||| ||||||||||||||||||||| ||

Tgt 161 + CGTCTTGGACCGGCGGTAGGGGCCCTGTGAAACTCCTTCGACGGGT 206

206 cols, 204 ids (99.0%), 0 gaps (0.0%)

Query 206nt >Otu41

Target 206nt >Otu88

Qry 1 + AAACCAACAGGGATTGCCTCAGTAACGGCGAGTGAAGCGGCAACAGCTCAAATTTGAAATCTGGCCTCTTTGGGGTCCGA 80

||||||||||||||||||||||||||||||||||||||||||||||||||||||||||||||||||||||||||||||||

Tgt 1 + AAACCAACAGGGATTGCCTCAGTAACGGCGAGTGAAGCGGCAACAGCTCAAATTTGAAATCTGGCCTCTTTGGGGTCCGA 80

Qry 81 + GTTGTAATTTGTAGAGGATGTTTCGGGTACCGCCTCGGTTTAAATTTCTTGGAACAGAATGTCAGAGAGGGTGAGAATCC 160

||||||||||||||||||||||||||||||||||||||||||||||||||||||||||||||||||||||||||||||||

Tgt 81 + GTTGTAATTTGTAGAGGATGTTTCGGGTACCGCCTCGGTTTAAATTTCTTGGAACAGAATGTCAGAGAGGGTGAGAATCC 160

Qry 161 + CGTCTTGGACCGGCGGTAGGGCCCCTGTGAAACTCCTTCGACGAGT 206

||||||||||||||||||||| ||||||| || ||||||| || |

Tgt 161 + CGTCTTGGACCGGCGGTAGGGGCCCTGTGGAAATCCTTCGGCGGGG 206

206 cols, 200 ids (97.1%), 0 gaps (0.0%)

Query >Otu35

%Id TLen Target

99% 206 Otu44

97% 206 Otu88

Query 206nt >Otu35

Target 206nt >Otu44

Qry 1 + AAACCAACAGGGATTGCCTCAGTAACGGCGAGTGAAGCGGCAACAGCTCAAATTTGAAATCTGGCCTCTTTGGGGTCCGA 80

||||||||||||||||||||||||||||||||||||||||||||||||||||||||||||||||||||||||||||||||

Tgt 1 + AAACCAACAGGGATTGCCTCAGTAACGGCGAGTGAAGCGGCAACAGCTCAAATTTGAAATCTGGCCTCTTTGGGGTCCGA 80

Qry 81 + GTTGTAATTTGTAGAGGATGTTTCGGGTACCGCCTCGGTTTAAATTTCTTGGAACAGAATGTCAGAGAGGGTGAGAATCC 160

||||||||||||||||||||||||||||||||||||||||||||||||||||||||||||||||||||||||||||||||

Tgt 81 + GTTGTAATTTGTAGAGGATGTTTCGGGTACCGCCTCGGTTTAAATTTCTTGGAACAGAATGTCAGAGAGGGTGAGAATCC 160

Qry 161 + CGTCTTGGACCGGCGGTAGGGCCCATGTGAAACTCCTTCGACGAGT 206

||||||||||||||||||||| || |||||||||||||||||| ||

Tgt 161 + CGTCTTGGACCGGCGGTAGGGGCCCTGTGAAACTCCTTCGACGGGT 206

206 cols, 203 ids (98.5%), 0 gaps (0.0%)

Query 206nt >Otu35

Target 206nt >Otu88

Qry 1 + AAACCAACAGGGATTGCCTCAGTAACGGCGAGTGAAGCGGCAACAGCTCAAATTTGAAATCTGGCCTCTTTGGGGTCCGA 80

||||||||||||||||||||||||||||||||||||||||||||||||||||||||||||||||||||||||||||||||

Tgt 1 + AAACCAACAGGGATTGCCTCAGTAACGGCGAGTGAAGCGGCAACAGCTCAAATTTGAAATCTGGCCTCTTTGGGGTCCGA 80

Qry 81 + GTTGTAATTTGTAGAGGATGTTTCGGGTACCGCCTCGGTTTAAATTTCTTGGAACAGAATGTCAGAGAGGGTGAGAATCC 160

||||||||||||||||||||||||||||||||||||||||||||||||||||||||||||||||||||||||||||||||

Tgt 81 + GTTGTAATTTGTAGAGGATGTTTCGGGTACCGCCTCGGTTTAAATTTCTTGGAACAGAATGTCAGAGAGGGTGAGAATCC 160

Qry 161 + CGTCTTGGACCGGCGGTAGGGCCCATGTGAAACTCCTTCGACGAGT 206

||||||||||||||||||||| || |||| || ||||||| || |

Tgt 161 + CGTCTTGGACCGGCGGTAGGGGCCCTGTGGAAATCCTTCGGCGGGG 206

206 cols, 199 ids (96.6%), 0 gaps (0.0%)

Query >Otu88

%Id TLen Target

98% 206 Otu44

Query 206nt >Otu88

Target 206nt >Otu44

Qry 1 + AAACCAACAGGGATTGCCTCAGTAACGGCGAGTGAAGCGGCAACAGCTCAAATTTGAAATCTGGCCTCTTTGGGGTCCGA 80

||||||||||||||||||||||||||||||||||||||||||||||||||||||||||||||||||||||||||||||||

Tgt 1 + AAACCAACAGGGATTGCCTCAGTAACGGCGAGTGAAGCGGCAACAGCTCAAATTTGAAATCTGGCCTCTTTGGGGTCCGA 80

Qry 81 + GTTGTAATTTGTAGAGGATGTTTCGGGTACCGCCTCGGTTTAAATTTCTTGGAACAGAATGTCAGAGAGGGTGAGAATCC 160

||||||||||||||||||||||||||||||||||||||||||||||||||||||||||||||||||||||||||||||||

Tgt 81 + GTTGTAATTTGTAGAGGATGTTTCGGGTACCGCCTCGGTTTAAATTTCTTGGAACAGAATGTCAGAGAGGGTGAGAATCC 160

Qry 161 + CGTCTTGGACCGGCGGTAGGGGCCCTGTGGAAATCCTTCGGCGGGG 206

||||||||||||||||||||||||||||| || ||||||| ||||

Tgt 161 + CGTCTTGGACCGGCGGTAGGGGCCCTGTGAAACTCCTTCGACGGGT 206

206 cols, 202 ids (98.1%), 0 gaps (0.0%)

#============================================================================================

#============================================================================================
