# Supplementary material for: Characterizing the Human Mycobiota: A Comparison of Small Subunit rRNA, ITS1, ITS2, and Large Subunit rRNA Genomic Targets
Source: Front Microbiol. 2018 Sep 19;9:2208. doi: 10.3389/fmicb.2018.02208 (PMC6157398; doi:10.3389/fmicb.2018.02208)
Supplement: Supplementary file 4 [file Image_1.pdf]

Mock_community_Full_taxonomy	Source	PCR	~Amplicon length (bp)	UNITE_Classification	RDP_Classification	Rel. %
d:Fungi,p:Ascomycota,c:Dothideomycetes,o:Pleosporales,f:Pleosporaceae,g:Alternaria,s:Alternaria_alternata	Wild type (LabPLUS)	++	350	d:Fungi_Unclassified	c:Dothideomycetes,s:Alternaria_alternata	2.0%
d:Fungi,p:Ascomycota,c:Dothideomycetes,o:Capnodiales,f:Davidiellaceae,g:Cladosporium,s:Cladosporium_sphaeospermum	Wild type (LabPLUS)	++	380	p:Ascomycota_Unclassified	c:Dothideomycetes,s:Cladosporium_halotolerans	1.2%
d:Fungi,p:Ascomycota,c:Eurotiomycetes,o:Eurotiales,f:Trichocomaceae,g:Aspergillus,s:Aspergillus_brasiliensis	ATCC® 16404™	++	350	p:Ascomycota_Unclassified*	c:Eurotiomycetes,g:Aspergillus*	9%* (incl. A. niger)
d:Fungi,p:Ascomycota,c:Eurotiomycetes,o:Eurotiales,f:Trichocomaceae,g:Aspergillus,s:Aspergillus_flavus	Wild type (LabPLUS)	+++	380	c:Eurotiomycetes,g:Aspergillus	c:Eurotiomycetes,s:Aspergillus_flavus	3.6%
d:Fungi,p:Ascomycota,c:Eurotiomycetes,o:Eurotiales,f:Trichocomaceae,g:Aspergillus,s:Aspergillus_fumigatus	Wild type (LabPLUS)	+++	380	c:Eurotiomycetes,s:Aspergillus_fumigatus	c:Eurotiomycetes,s:Aspergillus_fumigatus	5.8%
d:Fungi,p:Ascomycota,c:Eurotiomycetes,o:Eurotiales,f:Trichocomaceae,g:Aspergillus,s:Aspergillus_niger	ATCC® 9642™	+++	380	p:Ascomycota_Unclassified*	c:Eurotiomycetes,g:Aspergillus*	9%* (incl. A. brasiliensis)
d:Fungi,p:Ascomycota,c:Eurotiomycetes,o:Chaetothyriales,f:Herpotrichiellaceae,g:Exophiala,s:Exophiala_spinifera	ATCC® 14488™	+++	400	c:Eurotiomycetes,s:Exophiala_exophialae	c:Eurotiomycetes,s:Exophiala_spinifera	1.8%
d:Fungi,p:Ascomycota,c:Eurotiomycetes,o:Eurotiales,f:Trichocomaceae,g:Penicillium,s:Penicillium_chrysogenum	ATCC® 10002™	+++	400	c:Eurotiomycetes,g:Penicillium	c:Eurotiomycetes,g:Eupenicillium	2.3%
d:Fungi,p:Ascomycota,c:Eurotiomycetes,o:Onygenales,f:Arthrodermataceae,g:Trichophyton,s:Trichophyton_mentagrophytes	ATCC® 9533™	+++	500	c:Eurotiomycetes,s:Trichophyton_mentagrophytes	c:Eurotiomycetes,g:Arthroderma	4.1%
d:Fungi,p:Ascomycota,c:Saccharomycetes,o:Saccharomycetales,f:Saccharomycetales_fam_Incertae_sedis,g:Candida,s:Candida_albicans	ATCC® 10231™	+++	300	c:Saccharomycetes,g:Candida	c:Saccharomycetes,g:Candida	13.8%
d:Fungi,p:Ascomycota,c:Saccharomycetes,o:Saccharomycetales,f:Saccharomycetales_fam_Incertae_sedis,g:Candida,s:Candida_glabrata	ATCC® 90030™	+++	600	c:Saccharomycetes,o:Saccharomycetales_Unclassified	c:Saccharomycetes,s:Candida_glabrata	1.6%
d:Fungi,p:Ascomycota,c:Saccharomycetes,o:Saccharomycetales,f:Saccharomycetales_fam_Incertae_sedis,g:Candida,s:Candida_parapsilosis	ATCC® 22019™	+++	300	c:Saccharomycetes,s:Candida_parapsilosis	c:Saccharomycetes,s:Candida_parapsilosis	8.5%
d:Fungi,p:Ascomycota,c:Saccharomycetes,o:Saccharomycetales,f:Saccharomycetales_fam_Incertae_sedis,g:Candida,s:Candida_tropicalis	ATCC® 13802™	++	350	c:Saccharomycetes,s:Candida_tropicalis	c:Saccharomycetes,s:Candida_tropicalis	0.8%
d:Fungi,p:Ascomycota,c:Saccharomycetes,o:Saccharomycetales,f:Saccharomycetales_fam_Incertae_sedis,g:Yarrowia,s:Yarrowia_lipolytica	ATCC® 9773™	+	250	c:Saccharomycetes,s:Yarrowia_lipolytica	d:Fungi_unclassified	0.0%
d:Fungi,p:Ascomycota,c:Saccharomycetes,o:Saccharomycetales,f:Saccharomycetaceae,g:Saccharomyces,s:Saccharomyces_cerevisiae	Wild type (UoA)	+++	520	c:Saccharomycetes,g:Saccharomyces	c:Saccharomycetes,s:Saccharomyces_cerevisiae	3.1%
d:Fungi,p:Ascomycota,c:Sordariomycetes,o:Hypocreales,f:Nectriaceae,g:Fusarium,s:Fusarium_solani	Wild type (LabPLUS)	+++	380	d:Fungi_Unclassified	c:Sordariomycetes,f:Nectriaceae_Unclassified	4.8%
d:Fungi,p:Basidiomycota,c:Tremellomycetes,o:Tremellales,f:Tremellales_fam_Incertae_sedis,g:Cryptococcus,s:Cryptococcus_neoformans	ATCC® 32045™	+++	350	c:Tremellomycetes,s:Cryptococcus_neoformans	c:Tremellomycetes,g:Filobasidiella	4.6%
d:Fungi,p:Basidiomycota,c:Tremellomycetes,o:Trichosporonales,f:Trichosporonaceae,g:Trichosporon,s:Trichosporon_dermatis	ATCC® 204094™	+++	300	c:Tremellomycetes,g:Trichosporon	c:Tremellomycetes,s:Trichosporon_dermatis	33.0%
d:Fungi,p:Basidiomycota,c:Exobasidiomycetes,o:Malasseziales,f:Malasseziaceae,g:Malassezia,s:Malassezia_furfur	NZRM 3493	-		c:Exobasidiomycetes,s:Malassezia_furfur	c:Exobasidiomycetes,s:Malassezia_furfur	0.0%
d:Fungi,p:Basidiomycota,c:Exobasidiomycetes,o:Malasseziales,f:Malasseziaceae,g:Malassezia,s:Malassezia_globosa	ATCC® MYA4612™	+	450	c:Exobasidiomycetes,g:Malassezia	c:Exobasidiomycetes,s:Malassezia_globosa	< 0.1%
d:Fungi,p:Basidiomycota,c:Exobasidiomycetes,o:Malasseziales,f:Malasseziaceae,g:Malassezia,s:Malassezia_pachydermatis	Wild type (LabPLUS)	-		c:Exobasidiomycetes,g:Malassezia	c:Exobasidiomycetes,s:Malassezia_pachydermatis	0.0%

+++ Strong visible band
++ Moderate visible band
+ Weak visible band
- No visible band

Correct species (incl. teleomorph)
Correct genus (incl. teleomorph)
Correct class, incorrect or unclassified to genus
Not classified to class
Incorrect class

Over-represented taxa
Under-represented taxa

Image 1. Human mycobiota mock community: ITS1. Full taxonomy and isolate source for the 21 species that comprised the mock community, together with details of PCR band intensity (visualised via an agarose gel) **(PCR)**, approximate amplicon length (bp) including primers and adapters **(Amplicon length (bp))**, taxonomic classifications of sequences based on reference databases **(*Reference*_Classification)**, and the relative abundance of sequences for each taxon when sequenced as an equal parts mixed community **(Rel. %)**. ATCC = American Type Culture Collection. NZRM = New Zealand Reference Culture Collection. UoA = University of Auckland. **A. brasiliensis* and *A. niger* shared the same ZOTU for each of ITS1, ITS2, and LSU (i.e. had identical sequences).

Mock_community_Full_taxonomy	Source	PCR	~Amplicon length (bp)	UNITE_Classification	RDP_Classification	Rel. %
d:Fungi,p:Ascomycota,c:Dothideomycetes,o:Pleosporales,f:Pleosporaceae,g:Alternaria,s:Alternaria_alternata	Wild type (LabPLUS)	++	450	c:Dothideomycetes,g:Alternaria	c:Dothideomycetes,g:Alternaria	1.0%
d:Fungi,p:Ascomycota,c:Dothideomycetes,o:Capnodiales,f:Davidiellaceae,g:Cladosporium,s:Cladosporium_sphaeospermum	Wild type (LabPLUS)	++	480	c:Dothideomycetes,o:Capnodiales_Unclassified	c:Dothideomycetes,g:Cladosporium	1.0%
d:Fungi,p:Ascomycota,c:Eurotiomycetes,o:Eurotiales,f:Trichocomaceae,g:Aspergillus,s:Aspergillus_brasiliensis	ATCC® 16404™	++	420	c:Eurotiomycetes,s:Aspergillus_brasiliensis*	c:Eurotiomycetes,g:Aspergillus*	7.9%* (incl. A. niger)
d:Fungi,p:Ascomycota,c:Eurotiomycetes,o:Eurotiales,f:Trichocomaceae,g:Aspergillus,s:Aspergillus_flavus	Wild type (LabPLUS)	+++	450	c:Eurotiomycetes,s:Aspergillus_flavus	c:Eurotiomycetes,g:Petromyces	2.7%
d:Fungi,p:Ascomycota,c:Eurotiomycetes,o:Eurotiales,f:Trichocomaceae,g:Aspergillus,s:Aspergillus_fumigatus	Wild type (LabPLUS)	+++	450	c:Eurotiomycetes,s:Aspergillus_lentulus	c:Eurotiomycetes,g:Neosartorya	4.1%
d:Fungi,p:Ascomycota,c:Eurotiomycetes,o:Eurotiales,f:Trichocomaceae,g:Aspergillus,s:Aspergillus_niger	ATCC® 9642™	+++	450	c:Eurotiomycetes,s:Aspergillus_brasiliensis*	c:Eurotiomycetes,g:Aspergillus*	7.9%* (incl. A. brasiliensis)
d:Fungi,p:Ascomycota,c:Eurotiomycetes,o:Chaetothyriales,f:Herpotrichiellaceae,g:Exophiala,s:Exophiala_spinifera	ATCC® 14488™	+++	450	c:Eurotiomycetes,g:Exophiala	c:Eurotiomycetes,s:Exophiala_spinifera	1.2%
d:Fungi,p:Ascomycota,c:Eurotiomycetes,o:Eurotiales,f:Trichocomaceae,g:Penicillium,s:Penicillium_chrysogenum	ATCC® 10002™	+++	400	c:Eurotiomycetes,o:Eurotiales_Unclassified	c:Eurotiomycetes,g:Penicillium	1.9%
d:Fungi,p:Ascomycota,c:Eurotiomycetes,o:Onygenales,f:Arthrodermataceae,g:Trichophyton,s:Trichophyton_mentagrophytes	ATCC® 9533™	+++	450	c:Eurotiomycetes,g:Trichophyton	c:Eurotiomycetes,s:Arthroderma_vanbreuseghemii	3.6%
d:Fungi,p:Ascomycota,c:Saccharomycetes,o:Saccharomycetales,f:Saccharomycetales_fam_Incertae_sedis,g:Candida,s:Candida_albicans	ATCC® 10231™	+++	350	c:Saccharomycetes,s:Candida_albicans	c:Saccharomycetes,g:Candida	9.7%
d:Fungi,p:Ascomycota,c:Saccharomycetes,o:Saccharomycetales,f:Saccharomycetales_fam_Incertae_sedis,g:Candida,s:Candida_glabrata	ATCC® 90030™	+++	520	c:Saccharomycetes,g:Candida	c:Saccharomycetes,s:Candida_glabrata	4.7%
d:Fungi,p:Ascomycota,c:Saccharomycetes,o:Saccharomycetales,f:Saccharomycetales_fam_Incertae_sedis,g:Candida,s:Candida_parapsilosis	ATCC® 22019™	+++	320	c:Saccharomycetes,g:Candida	c:Saccharomycetes,g:Candida	5.1%
d:Fungi,p:Ascomycota,c:Saccharomycetes,o:Saccharomycetales,f:Saccharomycetales_fam_Incertae_sedis,g:Candida,s:Candida_tropicalis	ATCC® 13802™	++	450	c:Saccharomycetes,g:Candida	c:Saccharomycetes,s:Candida_tropicalis	0.4%
d:Fungi,p:Ascomycota,c:Saccharomycetes,o:Saccharomycetales,f:Saccharomycetales_fam_Incertae_sedis,g:Yarrowia,s:Yarrowia_lipolytica	ATCC® 9773™	+++	300	c:Saccharomycetes,s:Yarrowia_lipolytica	p:Ascomycota_Unclassified	19.9%
d:Fungi,p:Ascomycota,c:Saccharomycetes,o:Saccharomycetales,f:Saccharomycetaceae,g:Saccharomyces,s:Saccharomyces_cerevisiae	Wild type (UoA)	+++	480	c:Saccharomycetes,g:Saccharomyces	c:Saccharomycetes,s:Saccharomyces_kudriavzevii	6.1%
d:Fungi,p:Ascomycota,c:Sordariomycetes,o:Hypocreales,f:Nectriaceae,g:Fusarium,s:Fusarium_solani	Wild type (LabPLUS)	+++	400	c:Sordariomycetes,g:Fusarium	c:Sordariomycetes,f:Nectriaceae_Unclassified	1.8%
d:Fungi,p:Basidiomycota,c:Tremellomycetes,o:Tremellales,f:Tremellales_fam_Incertae_sedis,g:Cryptococcus,s:Cryptococcus_neoformans	ATCC® 32045™	+++	500	c:Tremellomycetes,g:Cryptococcus	c:Tremellomycetes,g:Filobasidiella	5.2%
d:Fungi,p:Basidiomycota,c:Tremellomycetes,o:Trichosporonales,f:Trichosporonaceae,g:Trichosporon,s:Trichosporon_dermatis	ATCC® 204094™	+++	400	c:Tremellomycetes,g:Trichosporon	c:Tremellomycetes,s:Trichosporon_dermatis	21.5%
d:Fungi,p:Basidiomycota,c:Exobasidiomycetes,o:Malasseziales,f:Malasseziaceae,g:Malassezia,s:Malassezia_furfur	NZRM 3493	++	650	c:Exobasidiomycetes,s:Malassezia_furfur	c:Exobasidiomycetes,s:Malassezia_furfur	0.1%
d:Fungi,p:Basidiomycota,c:Exobasidiomycetes,o:Malasseziales,f:Malasseziaceae,g:Malassezia,s:Malassezia_globosa	ATCC® MYA4612™	+++	600	c:Exobasidiomycetes,g:Malassezia	c:Exobasidiomycetes,s:Malassezia_globosa	1.6%
d:Fungi,p:Basidiomycota,c:Exobasidiomycetes,o:Malasseziales,f:Malasseziaceae,g:Malassezia,s:Malassezia_pachydermatis	Wild type (LabPLUS)	+++	620	c:Exobasidiomycetes,s:Malassezia_pachydermatis	c:Exobasidiomycetes,s:Malassezia_pachydermatis	0.3%

+++ Strong visible band
++ Moderate visible band
+ Weak visible band
- No visible band

Correct species (incl. teleomorph)
Correct genus (incl. teleomorph)
Correct class, incorrect or unclassified to genus
Not classified to class
Incorrect class

Over-represented taxa
Under-represented taxa

Supplementary material 1B. Human mycobiota mock community: ITS2. Full taxonomy and isolate source for the 21 species that comprised the mock community, together with details of PCR band intensity (visualised via an agarose gel) **(PCR)**, approximate amplicon length (bp) including primers and adapters **(Amplicon length (bp))**, taxonomic classifications of sequences based on reference databases **(*Reference*_Classification)**, and the relative abundance of sequences for each taxon when sequenced as an equal parts mixed community **(Rel. %)**. ATCC = American Type Culture Collection. NZRM = New Zealand Reference Culture Collection. UoA = University of Auckland. *A. brasiliensis* and *A. niger* shared the same ZOTU for each of ITS1, ITS2, and LSU (i.e. had identical sequences).

Mock_community_Full_taxonomy	Source	PCR	~Amplicon length (bp)	SILVA_Classification	Rel. %
d:Fungi,p:Ascomycota,c:Dothideomycetes,o:Pleosporales,f:Pleosporaceae,g:Alternaria,s:Alternaria_alternata	Wild type (LabPLUS)	+	650	c:Dothideomycetes_Unclassified	
d:Fungi,p:Ascomycota,c:Dothideomycetes,o:Capnodiales,f:Davidiellaceae,g:Cladosporium,s:Cladosporium_sphaeospermum	Wild type (LabPLUS)	+	650	p:Ascomycota_Unclassified	
d:Fungi,p:Ascomycota,c:Eurotiomycetes,o:Eurotiales,f:Trichocomaceae,g:Aspergillus,s:Aspergillus_brasiliensis	ATCC® 16404™	+	600	Mixed classification; split across numerous Zotus**	
d:Fungi,p:Ascomycota,c:Eurotiomycetes,o:Eurotiales,f:Trichocomaceae,g:Aspergillus,s:Aspergillus_flavus	Wild type (LabPLUS)	+	650	c:Eurotiomycetes,s:Aspergillus_sojae	
d:Fungi,p:Ascomycota,c:Eurotiomycetes,o:Eurotiales,f:Trichocomaceae,g:Aspergillus,s:Aspergillus_fumigatus	Wild type (LabPLUS)	+	650	c:Eurotiomycetes_Unclassified	
d:Fungi,p:Ascomycota,c:Eurotiomycetes,o:Eurotiales,f:Trichocomaceae,g:Aspergillus,s:Aspergillus_niger	ATCC® 9642™	++	650	c:Eurotiomycetes,g:Aspergillus	
d:Fungi,p:Ascomycota,c:Eurotiomycetes,o:Chaetothyriales,f:Herpotrichiellaceae,g:Exophiala,s:Exophiala_spinifera	ATCC® 14488™	+	650	c:Eurotiomycetes_Unclassified	
d:Fungi,p:Ascomycota,c:Eurotiomycetes,o:Eurotiales,f:Trichocomaceae,g:Penicillium,s:Penicillium_chrysogenum	ATCC® 10002™	+	650	c:Eurotiomycetes,s:Penicillium_camemberti	
d:Fungi,p:Ascomycota,c:Eurotiomycetes,o:Onygenales,f:Arthrodermataceae,g:Trichophyton,s:Trichophyton_mentagrophytes	ATCC® 9533™	++	650	c:Eurotiomycetes,g:Trichophyton	
d:Fungi,p:Ascomycota,c:Saccharomycetes,o:Saccharomycetales,f:Saccharomycetales_fam_Incertae_sedis,g:Candida,s:Candida_albicans	ATCC® 10231™	++	650	c:Saccharomycetes_Unclassified*	
d:Fungi,p:Ascomycota,c:Saccharomycetes,o:Saccharomycetales,f:Saccharomycetales_fam_Incertae_sedis,g:Candida,s:Candida_glabrata	ATCC® 90030™	+++	650	c:Saccharomycetes_Unclassified	
d:Fungi,p:Ascomycota,c:Saccharomycetes,o:Saccharomycetales,f:Saccharomycetales_fam_Incertae_sedis,g:Candida,s:Candida_parapsilosis	ATCC® 22019™	+++	650	c:Saccharomycetes_Unclassified*	
d:Fungi,p:Ascomycota,c:Saccharomycetes,o:Saccharomycetales,f:Saccharomycetales_fam_Incertae_sedis,g:Candida,s:Candida_tropicalis	ATCC® 13802™	+++	650	c:Saccharomycetes_Unclassified	
d:Fungi,p:Ascomycota,c:Saccharomycetes,o:Saccharomycetales,f:Saccharomycetales_fam_Incertae_sedis,g:Yarrowia,s:Yarrowia_lipolytica	ATCC® 9773™	+++	580	c:Saccharomycetes,s:Yarrowia_lipolytica	
d:Fungi,p:Ascomycota,c:Saccharomycetes,o:Saccharomycetales,f:Saccharomycetaceae,g:Saccharomyces,s:Saccharomyces_cerevisiae	Wild type (UoA)	+++	580	c:Saccharomycetes,s:Saccharomyces_cerevisiae	
d:Fungi,p:Ascomycota,c:Sordariomycetes,o:Hypocreales,f:Nectriaceae,g:Fusarium,s:Fusarium_solani	Wild type (LabPLUS)	++	650	d:Eukaryota_Unclassified	
d:Fungi,p:Basidiomycota,c:Tremellomycetes,o:Tremellales,f:Tremellales_fam_Incertae_sedis,g:Cryptococcus,s:Cryptococcus_neoformans	ATCC® 32045™	+++	650	c:Tremellomycetes,s:Cryptococcus_gattii	
d:Fungi,p:Basidiomycota,c:Tremellomycetes,o:Trichosporonales,f:Trichosporonaceae,g:Trichosporon,s:Trichosporon_dermatis	ATCC® 204094™	+++	600	c:Tremellomycetes,g:Trichosporon	
d:Fungi,p:Basidiomycota,c:Exobasidiomycetes,o:Malasseziales,f:Malasseziaceae,g:Malassezia,s:Malassezia_furfur	NZRM 3493	+	650	c:Exobasidiomycetes_Unclassified	
d:Fungi,p:Basidiomycota,c:Exobasidiomycetes,o:Malasseziales,f:Malasseziaceae,g:Malassezia,s:Malassezia_globosa	ATCC® MYA4612™	++	650	c:Exobasidiomycetes,s:Malassezia_globosa	
d:Fungi,p:Basidiomycota,c:Exobasidiomycetes,o:Malasseziales,f:Malasseziaceae,g:Malassezia,s:Malassezia_pachydermatis	Wild type (LabPLUS)	+	650	c:Exobasidiomycetes,s:Malassezia_pachydermatis	

+++	Strong visible band
++	Moderate visible band
+	Weak visible band
-	No visible band

Colour key
Correct species (incl. teleomorph)
Correct genus (incl. teleomorph)
Correct class, incorrect or unclassified to genus
Not classified to class
Incorrect class

Supplementary material 1C. Human mycobiota mock community: SSU. Full taxonomy and isolate source for the 21 species that comprised the mock community, together with details of PCR band intensity (visualised via an agarose gel) **(PCR)**, approximate amplicon length (bp) including primers and adapters **(Amplicon length (bp))**, taxonomic classifications of sequences based on reference databases **(*Reference*_Classification)**, and the relative abundance of sequences for each taxon when sequenced as an equal parts mixed community **(Rel. %)** (not available for SSU data). ATCC = American Type Culture Collection. NZRM = New Zealand Reference Culture Collection. UoA = University of Auckland. *_C. albicans_ and _C. parapsilosis_ shared the same ZOTU for SSU. **_A. brasiliensis_ was split across numerous ZOTUs for SSU sequences and had mixed classification.

Mock_community_Full_taxonomy	Source	PCR	~Amplicon length (bp)	SILVA_Classification	RDP_Classification	Rel. %
d:Fungi,p:Ascomycota,c:Dothideomycetes,o:Pleosporales,f:Pleosporaceae,g:Alternaria,s:Alternaria_alternata	Wild type (LabPLUS)	++	700	c:Dothideomycetes_Unclassified	c:Dothideomycetes,g:Alternaria	0.7%
d:Fungi,p:Ascomycota,c:Dothideomycetes,o:Capnodiales,f:Davidiellaceae,g:Cladosporium,s:Cladosporium_sphaeospermum	Wild type (LabPLUS)	++	700	c:Dothideomycetes_Unclassified	c:Dothideomycetes,g:Cladosporium_complex	0.8%
d:Fungi,p:Ascomycota,c:Eurotiomycetes,o:Eurotiales,f:Trichocomaceae,g:Aspergillus,s:Aspergillus_brasiliensis	ATCC® 16404™	+	700	c:Eurotiomycetes,s:Aspergillus_niger*	c:Agaricomycetes,g:Poria*	7.5%* (incl. A. niger)
d:Fungi,p:Ascomycota,c:Eurotiomycetes,o:Eurotiales,f:Trichocomaceae,g:Aspergillus,s:Aspergillus_flavus	Wild type (LabPLUS)	+++	700	c:Eurotiomycetes,s:Aspergillus_oryzae	c:Eurotiomycetes,f:Trichocomaceae_Unclassified	2.7%
d:Fungi,p:Ascomycota,c:Eurotiomycetes,o:Eurotiales,f:Trichocomaceae,g:Aspergillus,s:Aspergillus_fumigatus	Wild type (LabPLUS)	+++	700	c:Eurotiomycetes,s:Aspergillus_fumigatus	c:Eurotiomycetes,g:Aspergillus	4.6%
d:Fungi,p:Ascomycota,c:Eurotiomycetes,o:Eurotiales,f:Trichocomaceae,g:Aspergillus,s:Aspergillus_niger	ATCC® 9642™	+++	700	c:Eurotiomycetes,s:Aspergillus_niger*	c:Agaricomycetes,g:Poria*	7.5%* (incl. A. brasiliensis)
d:Fungi,p:Ascomycota,c:Eurotiomycetes,o:Chaetothyriales,f:Herpotrichiellaceae,g:Exophiala,s:Exophiala_spinifera	ATCC® 14488™	++	700	p:Ascomycota_Unclassified	c:Eurotiomycetes,f:Herpotrichiellaceae	0.6%
d:Fungi,p:Ascomycota,c:Eurotiomycetes,o:Eurotiales,f:Trichocomaceae,g:Penicillium,s:Penicillium_chrysogenum	ATCC® 10002™	++	750	c:Eurotiomycetes_Unclassified	c:Eurotiomycetes,g:Penicillium	1.9%
d:Fungi,p:Ascomycota,c:Eurotiomycetes,o:Onygenales,f:Arthrodermataceae,g:Trichophyton,s:Trichophyton_mentagrophytes	ATCC® 9533™	+++	700	c:Eurotiomycetes,s:Trichophyton_tonsurans	c:Eurotiomycetes,g:Arthroderma	2.7%
d:Fungi,p:Ascomycota,c:Saccharomycetes,o:Saccharomycetales,f:Saccharomycetales_fam_Incertae_sedis,g:Candida,s:Candida_albicans	ATCC® 10231™	+++	650	c:Saccharomycetes,s:Candida_albicans	c:Saccharomycetes,f:Saccharomycetaceae_Unclassified	9.3%
d:Fungi,p:Ascomycota,c:Saccharomycetes,o:Saccharomycetales,f:Saccharomycetales_fam_Incertae_sedis,g:Candida,s:Candida_glabrata	ATCC® 90030™	+++	700	c:Saccharomycetes,s:Candida_glabrata	c:Saccharomycetes,g:Nakaseomyces	4.3%
d:Fungi,p:Ascomycota,c:Saccharomycetes,o:Saccharomycetales,f:Saccharomycetales_fam_Incertae_sedis,g:Candida,s:Candida_parapsilosis	ATCC® 22019™	+++	650	c:Saccharomycetes,s:Candida_parapsilosis	c:Saccharomycetes,g:Vanderwaltozyma	4.1%
d:Fungi,p:Ascomycota,c:Saccharomycetes,o:Saccharomycetales,f:Saccharomycetales_fam_Incertae_sedis,g:Candida,s:Candida_tropicalis	ATCC® 13802™	+	700	c:Saccharomycetes_Unclassified	c:Saccharomycetes,g:Lodderomyces	0.5%
d:Fungi,p:Ascomycota,c:Saccharomycetes,o:Saccharomycetales,f:Saccharomycetales_fam_Incertae_sedis,g:Yarrowia,s:Yarrowia_lipolytica	ATCC® 9773™	+++	650	c:Saccharomycetes,s:Yarrowia_lipolytica	c:Saccharomycetes,g:Yarrowia	16.9%
d:Fungi,p:Ascomycota,c:Saccharomycetes,o:Saccharomycetales,f:Saccharomycetaceae,g:Saccharomyces,s:Saccharomyces_cerevisiae	Wild type (UoA)	+++	650	c:Hexapoda,s:Polistes_fuscatus	c:Saccharomycetes,g:Saccharomyces	10.3%
d:Fungi,p:Ascomycota,c:Sordariomycetes,o:Hypocreales,f:Nectriaceae,g:Fusarium,s:Fusarium_solani	Wild type (LabPLUS)	+++	700	c:Sordariomycetes_Unclassified	c:Sordariomycetes,g:Nectria	3.7%
d:Fungi,p:Basidiomycota,c:Tremellomycetes,o:Tremellales,f:Tremellales_fam_Incertae_sedis,g:Cryptococcus,s:Cryptococcus_neoformans	ATCC® 32045™	+++	750	c:Tremellomycetes,s:Cryptococcus_gattii	c:Tremellomycetes,g:Cryptococcus	4.7%
d:Fungi,p:Basidiomycota,c:Tremellomycetes,o:Trichosporonales,f:Trichosporonaceae,g:Trichosporon,s:Trichosporon_dermatis	ATCC® 204094™	+++	700	c:Tremellomycetes_Unclassified	c:Tremellomycetes,g:Asterotremella	17.9%
d:Fungi,p:Basidiomycota,c:Exobasidiomycetes,o:Malasseziales,f:Malasseziaceae,g:Malassezia,s:Malassezia_furfur	NZRM 3493	++	750	c:Exobasidiomycetes,s:Malassezia	c:Exobasidiomycetes,g:Malassezia	0.6%
d:Fungi,p:Basidiomycota,c:Exobasidiomycetes,o:Malasseziales,f:Malasseziaceae,g:Malassezia,s:Malassezia_globosa	ATCC® MYA4612™	+++	750	c:Exobasidiomycetes,s:Malassezia_globosa	c:Exobasidiomycetes,g:Malassezia	5.3%
d:Fungi,p:Basidiomycota,c:Exobasidiomycetes,o:Malasseziales,f:Malasseziaceae,g:Malassezia,s:Malassezia_pachydermatis	Wild type (LabPLUS)	++	750	c:Exobasidiomycetes,s:Malassezia	c:Exobasidiomycetes,g:Malassezia	1.2%

+++ Strong visible band
++ Moderate visible band
+ Weak visible band
- No visible band

Correct species (incl. teleomorph)
Correct genus (incl. teleomorph)
Correct class, incorrect or unclassified to genus
Not classified to class
Incorrect class

Over-represented taxa
Under-represented taxa

Supplementary material 1D. Human mycobiota mock community: LSU. Full taxonomy and isolate source for the 21 species that comprised the mock community, together with details of PCR band intensity (visualised via an agarose gel) **(PCR)**, approximate amplicon length (bp) including primers and adapters **(Amplicon length (bp))**, taxonomic classifications of sequences based on reference databases **(*Reference*_Classification)**, and the relative abundance of sequences for each taxon when sequenced as an equal parts mixed community **(Rel. %)**. ATCC = American Type Culture Collection. NZRM = New Zealand Reference Culture Collection. UoA = University of Auckland. **A. brasiliensis* and *A. niger* shared the same ZOTU for each of ITS1, ITS2, and LSU (i.e. had identical sequences).
