# Supplementary material for: Characterizing the Human Mycobiota: A Comparison of Small Subunit rRNA, ITS1, ITS2, and Large Subunit rRNA Genomic Targets
Source: Front Microbiol. 2018 Sep 19;9:2208. doi: 10.3389/fmicb.2018.02208 (PMC6157398; doi:10.3389/fmicb.2018.02208)
Supplement: Supplementary file 5 [file Image_2.pdf]

(**A**)

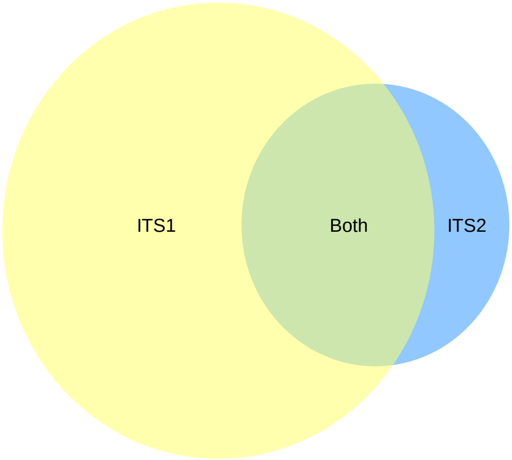

ITS1_only	shared	ITS2_only
p:Ascomycota,f:Mycosphaerellaceae_Unclassified	**d:Fungi_Unclassified**	p:Ascomycota,g:Toxicocladosporium
p:Ascomycota,f:Teratosphaeriaceae_Unclassified	**p:Ascomycota_Unclassified**	p:Ascomycota,g:Graphiopsis
p:Ascomycota,g:Devriesia	**p:Ascomycota,o:Capnodiales_Unclassified**	p:Ascomycota,g:Peltaster
p:Ascomycota,g:Microcyclospora	**p:Ascomycota,g:Cladosporium**	**p:Ascomycota,o:Pleosporales_Unclassified**
p:Ascomycota,g:Paraphoma	**p:Ascomycota,g:Davidiella**	p:Ascomycota,f:Tubeufiaceae_Unclassified
p:Ascomycota,f:Montagnulaceae_Unclassified	p:Ascomycota,g:Mycosphaerella	p:Ascomycota,g:Petromyces
p:Ascomycota,g:Preussia	**p:Ascomycota,g:Kabatiella**	p:Ascomycota,f:Orbiliaceae_Unclassified
p:Ascomycota,c:Eurotiomycetes_Unclassified	p:Ascomycota,f:Didymellaceae_Unclassified	p:Ascomycota,g:Clavispora
p:Ascomycota,f:Herpotrichiellaceae_Unclassified	p:Ascomycota,g:Leptosphaerulina	p:Ascomycota,g:Microascus
p:Ascomycota,g:Phialophora	p:Ascomycota,g:Cladophialophora	p:Basidiomycota,g:Hemimycena
p:Ascomycota,f:Trichocomaceae_Unclassified	**p:Ascomycota,g:Exophiala**	p:Basidiomycota,g:Hyphodontia
p:Ascomycota,g:Arthroderma/Trichophyton	**p:Ascomycota,g:Aspergillus**	p:Basidiomycota,f:Peniophoraceae_Unclassified
p:Ascomycota,g:Erysiphe	**p:Ascomycota,g:Penicillium/Eupenicillium**	p:Basidiomycota,c:Microbotryomycetes_Unclassified
p:Ascomycota,g:Dokmaia	p:Ascomycota,g:Pichia	
p:Ascomycota,g:Knufia	**p:Ascomycota,g:Debaryomyces**	
p:Ascomycota,o:Saccharomycetales_Unclassified	**p:Ascomycota,g:Candida**	
p:Ascomycota,g:Meyerozyma	**p:Ascomycota,g:Thielaviopsis**	
p:Ascomycota,g:Lodderomyces	p:Basidiomycota_Unclassified	
p:Ascomycota,c:Sordariomycetes_Unclassified	p:Basidiomycota,c:Agaricomycetes_Unclassified	
p:Ascomycota,o:Hypocreales_Unclassified	p:Basidiomycota,g:Tricholoma	
p:Ascomycota,f:Hypocreales_Incertae_sedis_Unclassified	p:Basidiomycota,g:Rhizoctonia/Ceratobasidium	
p:Ascomycota,f:Nectriaceae_Unclassified	p:Basidiomycota,g:Trametes	
p:Ascomycota,g:Gibberella	**p:Basidiomycota,g:Rhodotorula/Rhodosporidium**	
p:Ascomycota,g:Plectosphaerella	p:Basidiomycota,g:Mrakia	
p:Ascomycota,g:Pestalotiopsis	**p:Basidiomycota,g:Filobasidium**	
p:Basidiomycota,o:Agaricales_Unclassified	p:Basidiomycota,o:Tremellales_Unclassified	
p:Basidiomycota,g:Agaricus	**p:Basidiomycota,g:Cryptococcus**	
p:Basidiomycota,g:Pleurotus	**p:Basidiomycota,g:Filobasidiella**	
p:Basidiomycota,g:Scleroderma	**p:Basidiomycota,g:Trichosporon**	
p:Basidiomycota,g:Suillus	**p:Basidiomycota,g:Malassezia**	
p:Basidiomycota,o:Polyporales_Unclassified	**p:Basidiomycota,g:Wallemia**	
p:Basidiomycota,g:Ganoderma		
p:Basidiomycota,g:Phlebia		
p:Basidiomycota,g:Junghuhnia		
p:Basidiomycota,g:Peniophora		
p:Basidiomycota,g:Leucosporidium		
p:Basidiomycota,g:Sporidiobolus		
p:Basidiomycota,g:Sporobolomyces		
p:Basidiomycota,g:Cystofilobasidium		
p:Basidiomycota,g:Guehomyces		

(**B**)

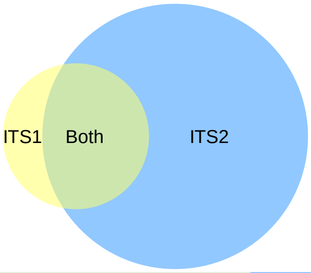

ITS1_only	shared	ITS2_only
p:Ascomycota,g:Hypocrea	d:Fungi_Unclassified	**p:Ascomycota,o:Pleosporales_Unclassified**
p:Basidiomycota,g:Psathyrella	**p:Ascomycota,g:Davidiella**	**p:Ascomycota,g:Leptosphaerulina**
**p:Basidiomycota,g:Rigidoporus**	**p:Ascomycota,g:Thermomyces**	p:Ascomycota,g:Dictyosporium
	**p:Ascomycota,g:Aspergillus/Eurotium**	p:Ascomycota,f:Trichocomaceae_Unclassified
	p:Ascomycota,g:Debaryomyces	**p:Ascomycota,g:Eupenicillium**
	p:Ascomycota,g:Gibellulopsis	p:Ascomycota,g:Paecilomyces
	p:Basidiomycota_Unclassified	p:Ascomycota,g:Candida
	**p:Basidiomycota,g:Clitopilus**	p:Ascomycota,o:Hypocreales_Unclassified
	**p:Basidiomycota,g:Cystofilobasidium**	p:Ascomycota,g:Khuskia/Nigrospora
		p:Basidiomycota,c:Agaricomycetes_Unclassified
		p:Basidiomycota,o:Agaricales_Unclassified
		p:Basidiomycota,g:Mycena
		**p:Basidiomycota,g:Rhodotorula**

Image 2. ITS1 and ITS2 community coverage for sinonasal and mouse fecal samples. Lists of unique taxonomic assignments and Venn diagrams representing the number of each taxa identified by ITS1 only (yellow), ITS2 only (blue), or both (green), for (**A**) human sinonasal, and (**B**) mouse fecal samples, with taxonomic assignments based on RDP ITS reference database. The 20 (sinonasal) and 10 (mouse fecal) most abundant taxa for each are given in bold.
